# RUNDC3A regulates SNAP25-mediated chemotherapy resistance by binding AKT in gastric neuroendocrine carcinoma (GNEC)

**DOI:** 10.1038/s41420-022-01084-4

**Published:** 2022-06-25

**Authors:** Pengchen Chen, Wei Wang, Sin Wa Wong, Junnan Li, Qiushaung Wu, Shu-Dong Zhang, Yao Lin, Hang Fai Kwok

**Affiliations:** 1 Cancer Centre, Faculty of Health Sciences, University of Macau, Avenida da Universidade, Taipa, Macau SAR China; 2 MoE Frontiers Science Center for Precision Oncology, University of Macau, Avenida de Universidade, Taipa, Macau SAR China; 3 Centre for Reproductive Medicine, Dongguan Maternal and Child Health Care Hospital, Southern Medical University, Guangzhou, China; 4 Northern Ireland Centre for Stratified Medicine, Ulster University, Londonderry, Northern Ireland UK; 5 Central Laboratory of the Second Affiliated Hospital of Fujian Traditional Chinese Medical University, Collaborative Innovation Center for Rehabilitation Technology, Fujian University of Traditional Chinese Medicine, Fuzhou, China

**Keywords:** Gastric cancer, Neuroendocrine cancer

## Abstract

Gastric neuroendocrine carcinoma (GNEC) is a common type of neuroendocrine carcinoma (NEC) with a poor prognosis and limited therapeutic options. The underlying mechanisms of chemoresistance in patients with GNEC and those with NEC are largely unknown, and thus, reliable biomarkers and therapeutic targets that could improve treatment outcomes in patients with NECs are lacking. The aim of this study was to identify specific targets and investigate their roles in GNEC progression and treatment resistance. Differentially expressed genes (DEGs) were identified in GNEC specimens and were further analysed by focusing on their roles in chemoresistance. Gene Ontology (GO) and pathway enrichment analyses of GNEC DEGs revealed that synapse-related function was the most prominent cellular function perturbed in GNEC. SNAP25 was identified as the target gene involved in most of the enriched pathways. In vitro and in vivo experiments showed that SNAP25 plays a role in proliferation and chemoresistance in GNEC cell lines. AKT has been identified as a downstream target, and SNAP25 binds to AKT protein and promotes AKT protein half-life. Further analysis of other types of NEC as well as small cell lung cancer, which resembles NEC on a molecular level, has identified RUNDC3A as an upstream molecule that regulates SNAP25 expression and the associated phenotypes that could enhance chemoresistance in NECs. Our results show that SNAP25 expression in GNEC is mediated by RUNDC3A and promotes GNEC progression and chemoresistance via posttranslational modification of AKT. Thus, our results suggest that the RUNDC3A/SNAP25/Akt axis could be a potential therapeutic target in GNEC.

## Introduction

Gastric neuroendocrine carcinoma (GNEC) is a specific type of cancer arising from enterochromaffin-like cells of the stomach [1] and is a subtype of gastroenteropancreatic neuroendocrine tumours (GEP-NETs), which arise from the gastrointestinal tract and pancreas [2]. Although GNEC is a rare form of cancer and accounts for only approximately 4.1% of all neuroendocrine tumours [3], its incidence has been increasing in most countries over the past 50 years [1]. According to a previous analysis of clinical data, GNEC is different from gastric adenocarcinoma (GAC) in many ways, including in malignancy, prognosis and treatment response [4–6]. Our previous studies using clinical specimens and cell lines in vitro also indicated that chemotherapeutic agents that have been used to treat GAC (platinum-based) may not be similarly efficacious for GNEC, which has resulted in a poorer prognosis in these patients [7, 8]. In addition, it has been shown that cytotoxic chemotherapy-based and somatostatin analogue-based therapies for GEP-NETs in an adjuvant setting conferred no benefit in terms of recurrence-free survival and overall survival (OS) [9]. Similarly, in a study that focused on patients with colorectal NEC, it was shown that neither 5-FU-based chemotherapy nor the combination of etoposide and platinum regimens improved survival in patients with stage I-III disease and that only stage IV patients could benefit from these chemotherapeutic agents [10]. Together, these results suggest that NETs and GNEC may develop resistance to chemotherapeutic agents during their progression.

Interestingly, an ex vivo study of small intestinal NEC showed that individual tumour samples have varying levels of sensitivity to different types of chemotherapeutic agents, which suggests that patients with small intestinal NECs who are treated with chemotherapy may exhibit a differential response. Therefore, the identification of biomarkers that could help optimise patient selection may be crucial for achieving better treatment outcomes [11].

Resistance to chemotherapy is one of the most common reasons for tumour progression in NEC patients; however, only a few published studies have investigated the mechanisms of chemotherapy resistance in NETs. It has been reported that pancreatic NET patients with retinoblastoma 1 (RB1) deletion and Kirsten rat sarcoma virus (KRAS) mutations exhibited a better treatment effect with platinum-based chemotherapy. As these mutations are relatively rare events in patients with this type of cancer, most of these patients do not respond well to platinum-based therapy [12]. Another study reported that both RB and p16 were predictive biomarkers of the response to the combination of etoposide and platinum, which suggests that cell cycle disruption in NECs may lead to differential sensitivity of the tumours towards these chemotherapeutic agents [13]. As NECs are highly heterogeneous, the underlying mechanisms of chemoresistance in NEC patients are largely unknown, which has resulted in a lack of reliable biomarkers and therapeutic targets that could direct treatment decisions and improve treatment outcomes in patients with NECs.

## Results

### Functional enrichment pattern of DEGs in GNEC

**Fig. 1:**
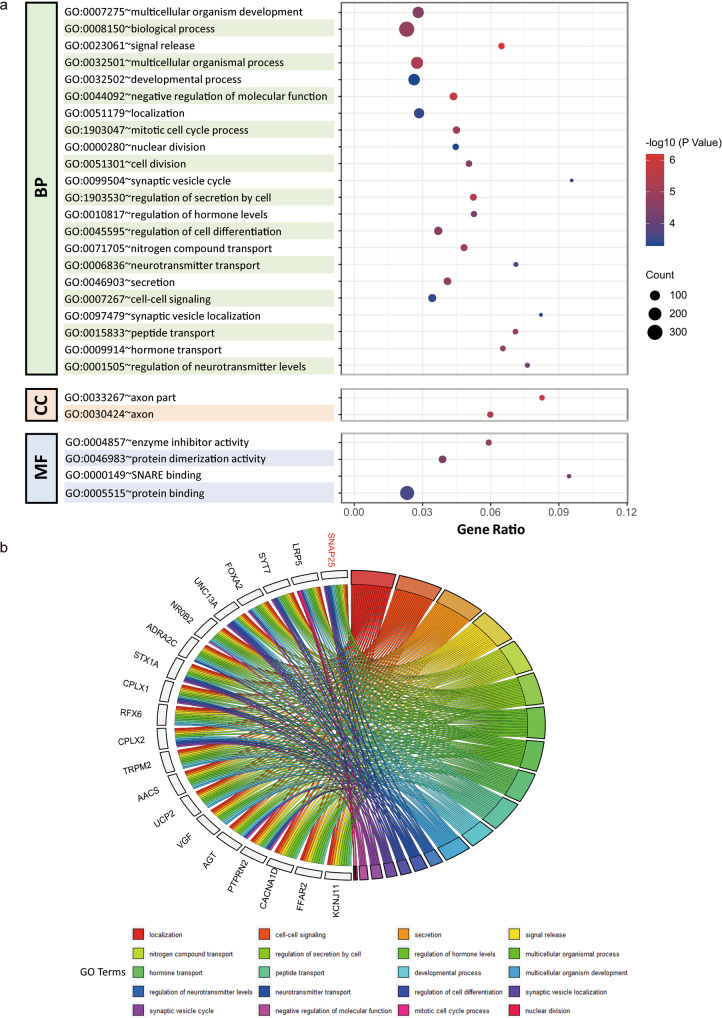
Functional enrichment pattern of DEGs in GNEC patient samples. **a** Gene Ontology functional enrichment analysis of DEGs in GNEC. In all, 28 GO terms were found to be significantly enriched. **b** Twenty enriched GO Terms in the category of Biological Process (except that with the highest hierarchy GO:0008150) were processed using a GOChord plot. Common genes (found in at least ten GO terms) are shown here on the left side of the circular plot, while enriched GO terms are shown on the right side.

**Fig. 2:**
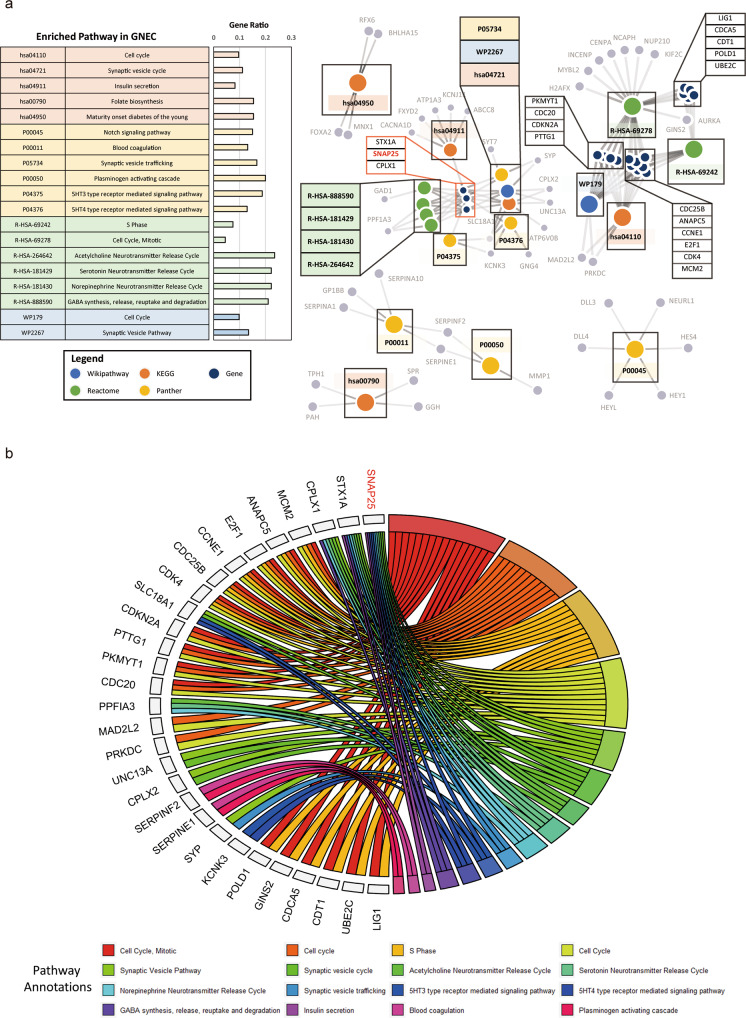
Pathway Enrichment Pattern of DEGs in GNEC patient samples. **a** Overrepresentative analyses revealed 19 pathways that were significantly enriched in GNEC. **b** Sixteen enriched pathway annotations were processed using a GOChord plot. Common genes (found in at least two pathway annotations) are listed on the left side, and enriched pathway annotations are listed on the right side.

**Fig. 3:**
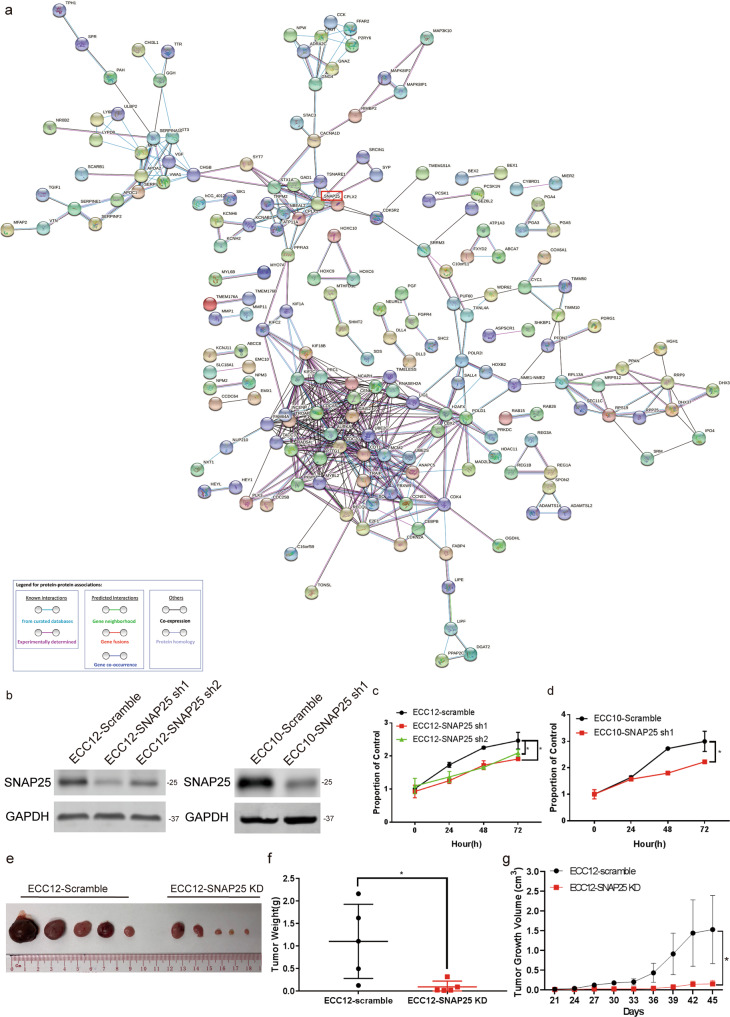
PPI network analysis of GNEC DEGs and the effect of SNAP25 on GNEC cell proliferation. **a** PPI analysis of GNEC DEGs. **b** SNAP25 knockdown effect in GNEC cell lines. **c**, **d** The changes in proliferation in SNAP25 knockdown GNEC cell lines. **e**–**g** The growth rate of SNAP25 knockdown GNEC cells in a xenograft model. Plots indicate the average ± s.d. Significance test is indicated *(*p* < 0.05).

Based on transcriptome profiling generated in our previous study [8], we identified 459 differentially expressed genes (DEGs) (with *p* value < 0.05 and fold change > = 4) by comparing the GNEC gene expression profile with the gene expression profile of nontumor specimens (Supplementary Table 1). Through GO (Gene Ontology) functional enrichment analysis using DAVID (Supplementary Tables 2–4), we found that synapsis-, transport- and secretion-related biological processes were among the top significantly enriched GO functional terms (Fig. 1a and Supplementary Table 5), while SNAP25 was found to be involved in most of these processes (Fig. 1b). Similarly, when pathway enrichment analysis was performed using four different databases (KEGG, WikiPathway, Reactome and Panther), we found that synapsis- or cell cycle-related pathways were significantly enriched in the DEGs identified in GNEC patient samples (Fig. 2a and Supplementary Table 6) and that SNAP25 was the most involved entry (Fig. 2b). Moreover, our protein-protein interaction (PPI) network analysis revealed that SNAP25 was indeed at the centre of the interaction network (Fig. 3a). Altogether, our results indicate that the dysregulation of synapse-related functions may be characteristic of GNEC, while SNAP25 may act as a hub within all enriched pathways.

### SNAP25 mediated cell proliferation and multiple drug resistance in GNEC cells

**Fig. 4:**
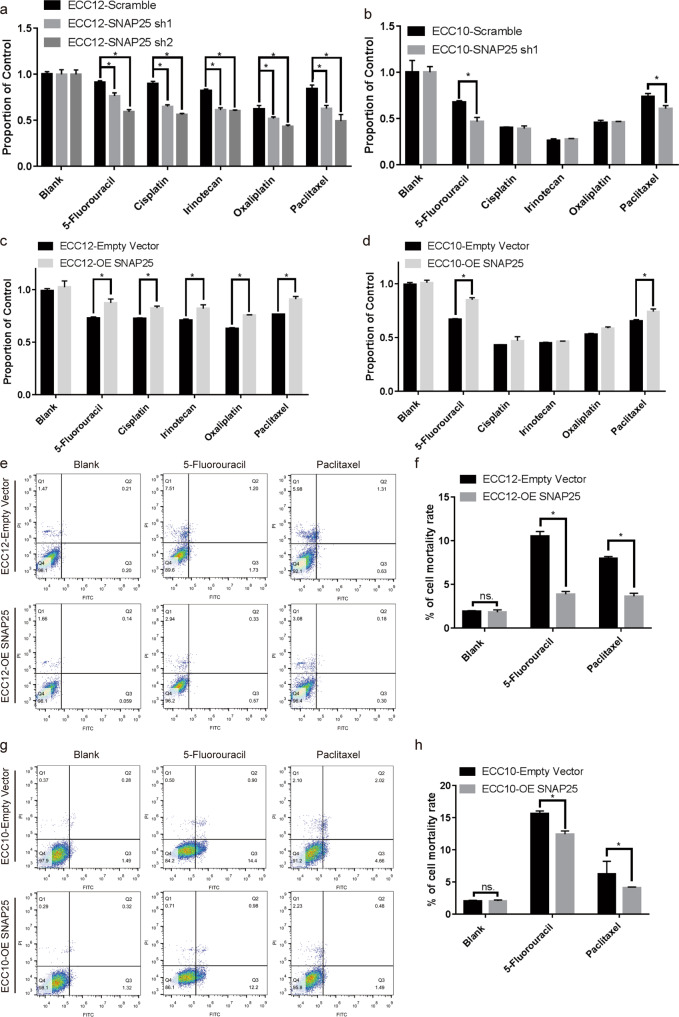
SNAP25 affects resistance to multiple drugs in GNEC cells. **a**–**d** Cell viability determination after treatment with multiple chemotherapy drugs in SNAP25 knockdown and overexpression GNEC cell lines. **e**–**h** Cell apoptosis detection after 5-fluorouracil and paclitaxel treatment in SNAP25 knockdown and overexpression GNEC cell lines. Plots indicate the average ± s.d. Significance test is indicated *(*p* < 0.05).

To understand the role of SNAP25 in GNEC, in vitro and in vivo experiments were performed. We first constructed stable SNAP25 knockdown cell lines using the two GNEC cell lines ECC10 and ECC12 (Fig. 3b). SNAP25 knockdown reduced cell proliferation of GNEC cell lines (Fig. 3c, d). Compared with the control group in the GNEC xenograft model, we found that SNAP25 knockdown significantly reduced tumour cell growth in vivo (Fig. 3e–g). To explore the role of SNAP25 in chemoresistance, we treated SNAP25 knockdown and SNAP25-overexpressing GNEC cell lines with chemotherapeutic agents commonly used in gastric cancer. We found that knockdown of SNAP25 expression sensitised GNEC cell lines to multiple chemotherapeutic agents, while SNAP25 overexpression in GNEC cell lines enhanced resistance to multiple chemotherapeutic agents (Fig. 4a–d). Further flow cytometric analysis showed that significantly fewer apoptotic cells were observed in ECC10 and ECC12 cells overexpressing SNAP25 after treatment with 5-fluorouracil and paclitaxel (Fig. 4e–h).

### AKT protein stability was mediated by SNAP25 via a direct binding effect

**Fig. 5:**
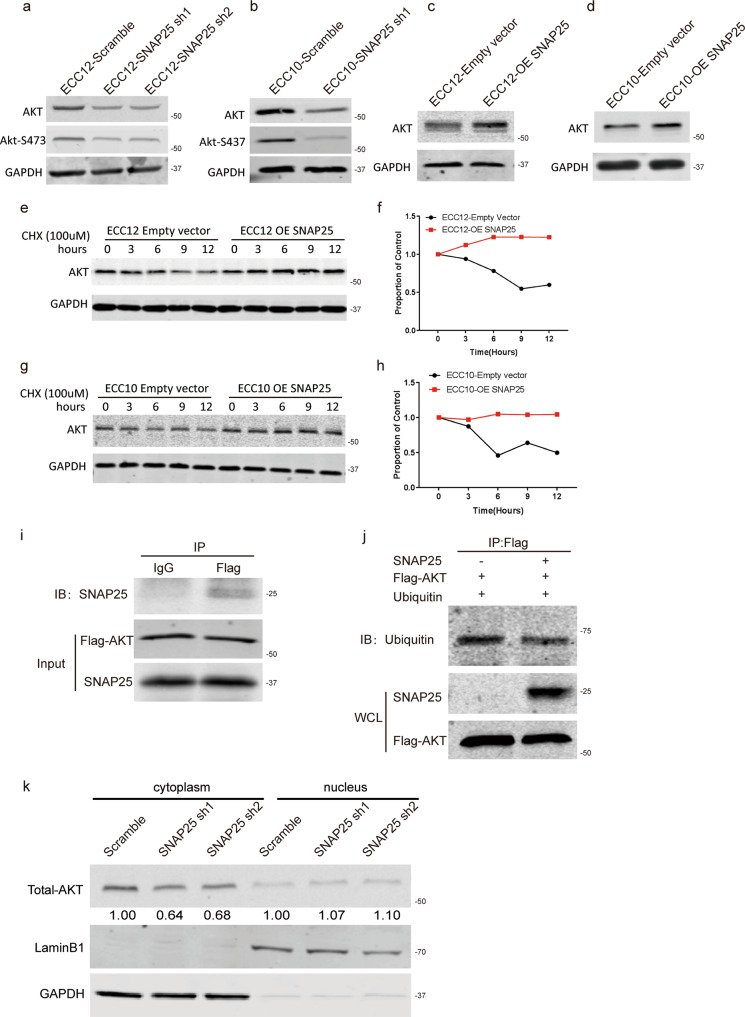
AKT protein stability is mediated by SNAP25 via a direct binding effect. **a**–**d** Changes in AKT protein levels were mediated by SNAP25 knockdown and overexpression in GNEC cell lines. **e**–**h** The half-life of AKT protein was mediated by SNAP25 overexpression in GNEC cells. **i** The binding effect between SNAP25 and AKT. **j** The AKT ubiquitination status was changed with or without SNAP25 expression. **k** The AKT protein level in the cytoplasm was changed upon SNAP25 disruption.

To investigate the downstream target of SNAP25, we tested the change in expression of different proteins from important oncogenic pathways and found that SNAP25 knockdown downregulated AKT protein levels, while SNAP25 overexpression upregulated AKT protein levels in the two GNEC cell lines (Fig. 5a–d). By RT–PCR, we found that modulating the expression of SNAP25 (Supplementary Fig. 1c and d) did not change the AKT mRNA expression level. To understand the mechanism by which AKT is regulated by SNAP25, we treated SNAP25 knockdown cells with MG132 and cycloheximide and found that SNAP25 regulates AKT expression at the posttranslational level (Supplementary Fig. 1e and Fig. 5e–h). Next, we explored the mechanism by which SNAP25 stabilises AKT protein. A co-IP experiment was performed, and SNAP25 was detected in the AKT pull-down sample (Fig. 5i), while AKT ubiquitination was reduced in SNAP25-overexpressing cells. This suggests that SNAP25 may interact with AKT and enhance its stability by reducing AKT monoubiquitination, which could protect AKT protein downstream of proteasome degradation (Fig. 5j). Subsequent cytoplasm and nuclear separation studies indicated that the change in AKT monoubiquitination by SNAP25 disruption could decrease AKT degradation in the cytoplasm but not in the nucleus (Fig. 5k). Importantly, we found that reduced proliferation induced by SNAP25 knockdown can be rescued by AKT overexpression (Supplementary Fig. 1a, 1b), which indicates that SNAP25 may enhance GNEC cell proliferation by stabilising AKT protein.

### Transcriptome pattern comparison among GNEC, PNET, SCLC and SINEC

**Fig. 6:**
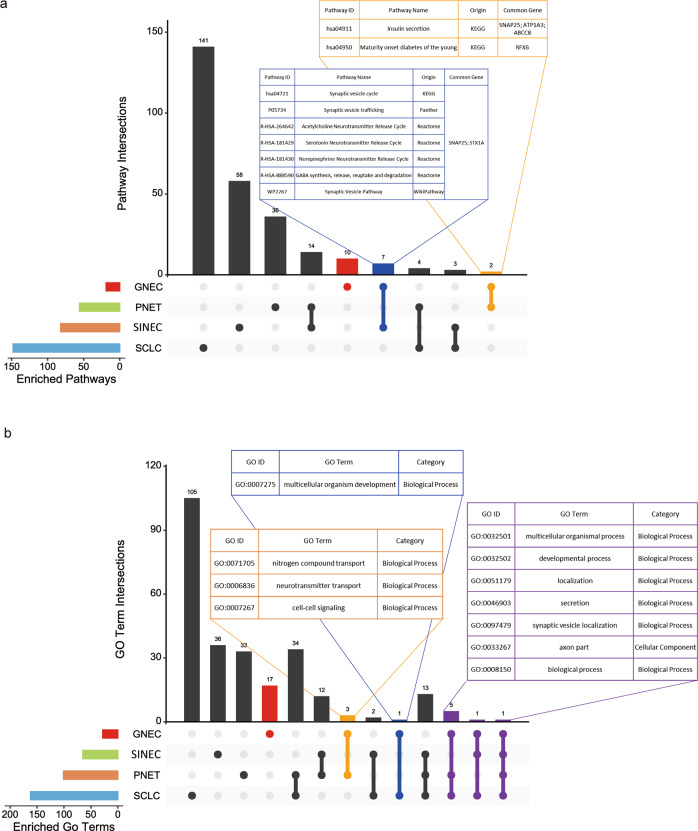
Functional enrichment pattern comparison among GNEC, PNET, SCLC and SINEC. **a** The enriched pathways from overrepresentative analyses across different cancers were processed using UpSetR plot. The connected dots indicate which cancers were assessed. The pathways found in GNEC are highlighted. **b** The enriched GO terms from overrepresentative analyses across different cancers were processed using UpSetR plot. The connected dots indicate which cancers were assessed. The GO terms found in GNEC are highlighted.

Since a similar chemotherapy resistance phenomenon has been observed in multiple NECs and related tumour types, we sought to understand whether SNAP25 plays a similar role in other NECs (including PNET, SINEC and SCLC) and tumours with similar molecular signatures. We first extracted DEGs (*p* value < 0.05, fold change > = 4) from the PNET (GSE43795), SCLC (GSE60052) and SINEC (E-GEOD-6272) databases (Supplementary Tables 7–9). Then, the DEGs identified in these four NECs were subjected to functional enrichment analysis using different pathway enrichment databases. We found that in GNEC and SINEC, seven common pathways were enriched (Fig. 6a). These seven common pathways were mostly related to the synaptic vesicle cycle and neurotransmitter cycle, which are synaptic cellular activities. Notably, SNAP25 and STX1A are common genes in these pathways. Furthermore, when focusing on the network of enriched pathways among SCLC, PNET and SINEC, we found that SNAP25 was at the centre and connects to most of the synapse-related pathways (Supplementary Fig. 2).

**Fig. 7:**
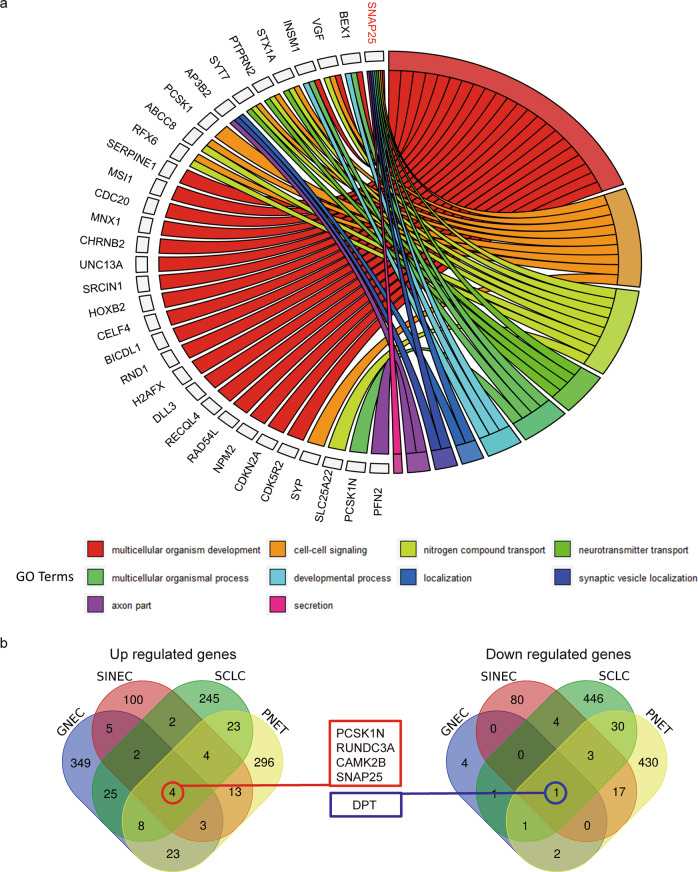
Pathway enrichment pattern of DEGs in multiple NECs. **a** Ten enriched GO terms (except that with the highest hierarchy GO:0008150) found in GNEC and at least one in another cancer type were processed using a GOChord plot. Common genes are listed on the left side, and enriched GO terms are listed on the right side. **b** The Venn diagrams show the number of coregulated genes among multiple NECs.

In addition, we observed that the DEGs were mostly genes that function in various transport- and synapse-related biological processes (Fig. 6b). Particularly, five biological processes, including synaptic vesicle localisation, were commonly enriched in SCLC, PNET and GNEC (Fig. 6b). The GO cellular component term axon part was commonly enriched in GNEC, SINEC and SCLC (Fig. 6b). Furthermore, we summarised the top ten enriched GO items (Supplementary Table 10). SNAP25 was also involved in the biological process of multicellular organism development, which co-occurred in both GNEC and SCLC enrichment results. Notably, SNAP25 participated in the most common enriched GO items (Fig. 7a). PPI network analyses revealed that SNAP25 was located in the hub of interaction networks and exhibited a close relationship with other DEGs in SCLC, PNET and SINEC (Supplementary Fig. 3–5). Taken together, our extended GO and pathway enrichment analyses indicate that the synapsis-related pathways were typical, significant pathways among NECs and that SNAP25 appears to be the most critical gene for synapsis-related cellular functions in multiple NECs.

When the DEGs were further compared across four cancer types, we found five coregulated DEGs (upregulated DEGs: PCSK1N, CAMK2B, RUNDC3A and SNAP25; downregulated DEG: DPT) among four NECs (Fig. 7b).

### RUNDC3A affected GNEC proliferation by regulating SNAP25 expression

**Fig. 8:**
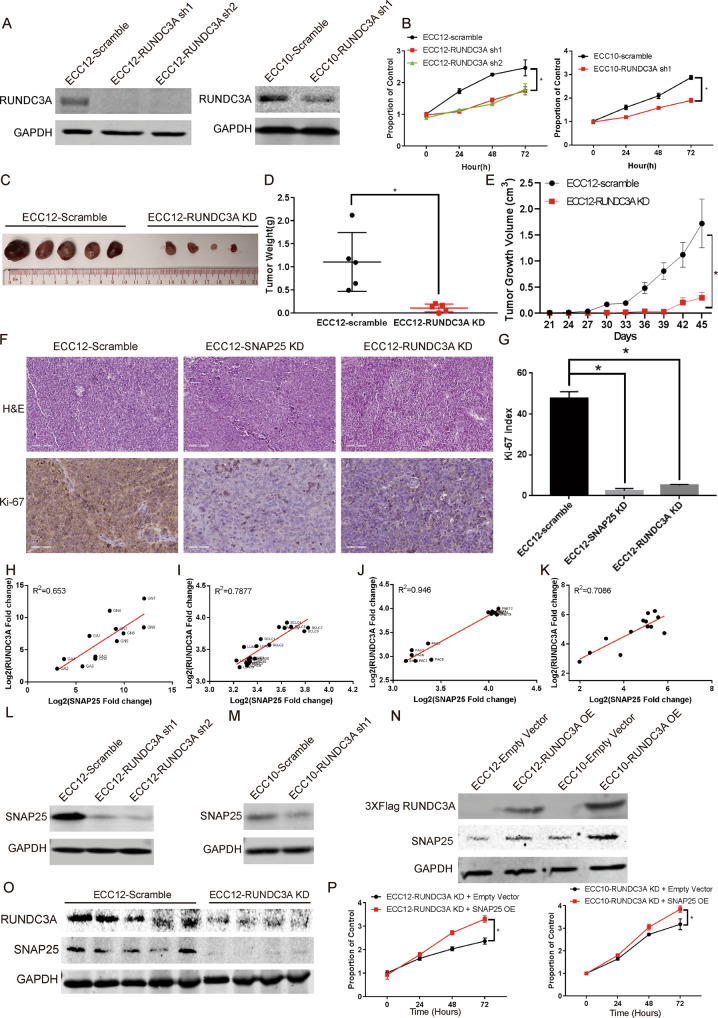
RUNDC3A affects GNEC proliferation by regulating SNAP25 expression. **A** Effect of RUNDC3A knockdown in GNEC cell lines. **B** The changes in GNEC cell proliferation after RUNDC3A knockdown. **C**–**E** The growth rate of GNEC cells in a xenograft model after RUNDC3A knockdown. **F**, **G** The Ki-67 expression level in SNAP25- and RUNDC3A-knockdown xenograft models. **H** The correlation of SNAP25 and RUNDC3A expression in GNEC and GAC patient samples. **I** The correlation of SNAP25 and RUNDC3A expression in SCLC and LUAD patient samples. **J** The correlation of SNAP25 and RUNDC3A expression in PNET and PAC patient samples. **K** The correlation of SNAP25 and RUNDC3A expression in SINEC patient samples. **L**–**N** SNAP25 expression was mediated by RUNDC3A expression in GNEC cells. **O** The correlation of SNAP25 and RUNDC3A expression in the RUNDC3A-knockdown xenograft model. **P** The SNAP25-mediated proliferation rate decrease could be rescued by RUNDC3A overexpression in GNEC cells. Plots indicate the average ± s.d. Significance test is indicated *(*p* < 0.05).

After we identified the coregulated DEGs among NECs, we focused on the three upregulated targets (PCSK1N, CAMK2B and RUNDC3A) other than SNAP25. Based on in vitro experiments, we found that knockdown of CAMK2B and PCSK1N did not affect the proliferation ability of the GNEC cell line ECC12 (Supplementary Fig. 6). However, in both GNEC cell lines ECC12 and ECC10, the proliferation ability was attenuated by RUNDC3A knockdown (Fig. 8A, B). Compared with the control group, RUNDC3A knockdown also significantly reduced the tumour growth rate as well as Ki67 staining in the GNEC xenograft model (Fig. 8C–G).

Based on the similar effect of SNAP25 and RUNDC3A knockdown on GNEC proliferation, we tested for any interactions between SNAP25 and RUNDC3A. We selected another five pairs of GNEC patient samples and four pairs of GAC patient samples for SNAP25 and RUNDC3A mRNA detection. Based on the comprehensive analysis of transcriptome data showing gene expression fold changes in these nine pairs of gastric cancer patient samples and three pairs of GNEC patient samples, we found an obvious correlation between SNAP25 and RUNDC3A expression in these gastric cancer samples (correlation coefficient = 0.653, *p* value = 0.0015) (Fig. 8H). Interestingly, using lung, pancreas and SINEC datasets for a similar correlation analysis, we also found a significant correlation between SNAP25 and RUNDC3A expression in these datasets (Fig. 8I–K). Moreover, based on the correlation analysis from GEPIA and the LinkedOmics platform, we demonstrated a significant coexpression relationship for these two genes in stomach adenocarcinoma (STAD), lung adenocarcinoma (LUAD) and pancreatic adenocarcinoma (PAC) patient samples (Supplementary Fig. 7a, b). Overall, these results indicate that SNAP25 and RUNDC3A expression was coregulated in various cancer types.

To verify this in silico analysis, we performed in vitro experiments to test whether the expression of SNAP25 is modulated by RUNDC3A or vice versa. We observed that RUNDC3A knockdown reduced SNAP25 expression levels in GNEC cells (Fig. 8L, M) and that SNAP25 expression was enhanced by RUNDC3A overexpression (Fig. 8N); however, the opposite was not observed (Supplementary Fig. 7c, d), which suggests that RUNDC3A may be an upstream regulator of SNAP25 in GNEC. Additionally, the regulation of SNAP25 by RUNDC3A was maintained in the in vivo xenograft model samples (Fig. 8O, Supplementary Fig. 7e). We further identified that SNAP25 overexpression rescued the reduced cell proliferation in RUNDC3A knockdown GNEC cell lines (Fig. 8p). Therefore, our results indicate that RUNDC3A may promote cell proliferation via SNAP25 in GNEC cells.

## Discussion

According to the prognosis of patients with GNEC treated with different treatment regimens, the median survival of patients who underwent radical resection, palliative resection, and chemotherapy alone was 48 months, 20 months, and 12 months, respectively [14], which suggests that chemotherapy is not effective in treating GNEC and that treatment resistance will likely develop. Therefore, understanding the mechanism of chemoresistance in GNEC is important. We reported here that synapse-related pathways were the most significant cellular function in GNEC cells and for the first time that SNAP25 modulates cell proliferation and chemoresistance in GNEC cells via AKT. SNAP25 is listed as a critical carcinogenic target and is potentially involved in the tumorigenesis process caused by Alibel’s disease [15]. The expression level of SNAP25 in diffuse large B-cell lymphoma was correlated with patient survival and prognosis [16]. SNAP25 was also found to be involved in the lysosomal autophagy pathway, which could in turn promote epithelial tumours [17].

In this study, we also identified that RUNDC3A regulates SNAP25 expression in GNEC cells, while SNAP25 stabilises AKT protein by binding and monoubiquitination regulation. Other studies have shown that the continuous activation of the AKT-related signalling pathway leads to enhanced chemoresistance in multiple cancer cells; for instance, activation of the AKT signalling pathway enhances platinum drug resistance in metastatic urothelial carcinoma and ovarian cancer [18–21] as well as in breast cancer [22]. Gastric cancer studies have shown that AKT pathway inactivation sensitizes cancer cells to chemotherapeutic agents [23–25]. We have demonstrated similar results in the current report, which shows that RUNDC3A/SNAP25 mediates chemoresistance by controlling AKT protein stability.

We have also shown that synapse-related pathways were the most common dysregulated pathways among GNEC, SCLC, PNET and SINEC. Synaptic activity is an essential part of cancer neurobiology, and synaptic function is associated with tumour progression. Synapse-related pathways are dysregulated in both primary and metastatic brain tumours [26–28], and the dysregulation of synapse-related pathways enhances tumour growth and confers treatment resistance [29, 30]. Moreover, in an ependymoma-related treatment resistance study, it was found that genes involved in synaptic vesicle-related pathways were enriched [31]. Indeed, other members of the SNARE family, including syntaxin-6 and VAMP-4, colocalize with a P-type ATPase and induce the secretory vesicular transport of cisplatin, which in turn promotes cell growth in ovarian cancer [32]. In summary, our report has demonstrated a novel observation that overexpression of RUNDC3A and SNAP25 modulates AKT protein stability to enhance tumour growth and chemoresistance in NECs, which suggests that this novel RUNDC3A/SNAP25/AKT axis may be a potential therapeutic target in patients with NECs.

## Materials and methods

### Patient sample collection

The resected carcinoma and paired adjacent tissue specimens of patients were stored in liquid nitrogen, and a fraction of each was sent for pathology inspection to confirm the diagnosis. The study was approved by the ethics committee of the Second Affiliated Hospital of Fujian Traditional Chinese Medical University (Fujian, China, SPHFJP-S2021124-01). Human tissue specimens collected for this study were from residual tissue in blocks generated for gastrectomy processing. All patients signed informed consent agreements for further examinations and investigations but were not involved in the design, conduct, reporting, or dissemination plans of our research.

### Cell culture

The GNEC cell lines ECC10 and ECC12 were purchased from RIKEN BRC CELL BANK (Japan). The GAC cell line AGS and the kidney epithelial cell line HEK293T were purchased from ATCC (CRL-1739). All cell lines were tested for mycoplasma contamination before use. The GNEC, GAC and HEK293T cell lines were cultured in RPMI 1640 supplemented with 10% foetal bovine serum (Gibco). Cells were cultured at 37 °C in an incubator with 5% CO2.

### Animals

Six-week-old female NOD SCID mice (20 ± 2 g) were provided by the Animal Facility of the Faculty of Health Sciences, University of Macau and were maintained on the premises under standard housing conditions. All mice were housed at an ambient temperature of 25 ± 2 °C and a relative humidity of e55 ± 5% under 12-hr light/dark cycles with free access to water and food for one week before the experiment. The mice were randomly assigned to the control group and the experimental group before the experiment under the condition of determining the approximate body weight (*n* = 5 mice in each group). All animal procedures were approved by the Animal Research Ethics Committee of the University of Macau. Tissue samples were fixed in 4% neutral-buffered formalin, embedded in paraffin, sectioned at a thickness of 4 μm, and stained with haematoxylin and eosin (H&E). For immunohistochemistry, formalin-fixed, paraffin-embedded sections were immunostained with an UltraSensitiveTM SP (Mouse/Rabbit) IHC Kit. Slides were stained using an anti-Ki-67 (1:500, Proteintech) antibody.

### Analysis of multiple datasets for gene expression correlation and related prognosis analysis

The SCLC (GSE60052) [33] and PNET (GSE43795) [34] datasets downloaded from the Gene Expression Omnibus (GEO), and the SINEC (E-GEOD-6272) datasets [35] downloaded from Array Express were used for the analysis. R scripting was used to extract the expression values of probesets from genes of interest. Gene expression correlation analysis was performed in GEPIA (http://gepia.cancer-pku.cn) and LinkedOmics (http://www.linkedomics.org/login.php). The associations between the expression levels of genes were analysed by Pearson’s correlation test. Gene ontology (GO) enrichment analyses were performed using DAVID bioinformatic resources 6.8 (https://david.ncifcrf.gov/) and the web tool REVIGO (http://revigo.irb.hr/). Pathway enrichment analyses against multiple pathway databases were performed using the web tool WebGestalt (http://www.webgestalt.org/). Protein–protein interaction functional enrichment analyses were performed using the STRING database v11.0 (http://www.string-db.org). The R packages UpSetR and GOplot were utilised for the visualisation of gene enrichment results.

### mRNA extraction and quantitative reverse transcription PCR (qRT–PCR)

Total RNA was extracted from cultured cells using an RNeasy Plus Universal Mini Kit (Qiagen), and the 260 nm/280 nm absorbance ratio was determined using a NanoDrop® ND-1000 spectrophotometer. The cDNA samples were prepared using a High-Capacity cDNA Reverse Transcription Kit (Applied Biosystems). Real-time PCR was performed using Taqman Fast Advanced Master Mix (Applied Biosystems). All reactions were run in triplicate and all experiments were repeated for three times. After the reactions were completed, fixed threshold settings were employed for all cycle threshold (*C*_T_) data determinations. The comparison of each condition and control reaction was performed using the comparative *C*_T_ method. The total mRNA levels were normalised to glyceraldehyde 3-phosphate dehydrogenase (GAPDH). The primers for GAPDH, SNAP25, RUNDC3A, and AKT were as follows: 5′-ACATCGCTCAGACACCATG-3′ (GAPDH, sense), 5′-TGTAGTTGAGGTCAATGAAGGG-3′ (GAPDH, anti-sense); 5′-TCCGCAGGGTAACAAATGAT-3′ (SNAP25, sense), 5′-TGGCCTCATCAATTCTGGTT-3′ (SNAP25, anti-sense); 5′-GCTGTGGAGCGTAAGAACCT-3′ (RUNDC3A, sense), 5′-CTGAACCAGCTCACTGGACC-3′ (RUNDC3A, anti-sense); 5′-CAGGATGTGGACCAACGTGA-3′ (AKT, sense), 5′-AAGGTGCGTTCGATGACAGT-3′ AKT, anti-sense).

### Establishment of stable cell lines

Lentiviral constructs of SNAP25- and RUNDC3A-related overexpression and knockdown plasmids and their corresponding empty vectors were purchased from Obio Technology Corp., Ltd. (Shanghai). Lentiviral packaging plasmid pCMV-dR8.2 DVPR (#8455), pCMV-VSV-G (#8454) and 3x Flag AKT expression plasmids (#9021) were purchased from Addgene. All the plasmids were amplified and extracted using gastric cancer cell lines seeded in 6-well plates at a concentration of 8 × 10^5^ cells per well before lentivirus infection. When the confluence reached approximately 50%, the cell lines were infected with the lentiviruses. After 48 h of infection, the cells were selected with puromycin (Gibco).

### Western blot and IHC

Protein extraction was performed using a Bio-Rad western blot system. The membranes were blocked with 5% fat-free dried milk in TBS at RT for 1 h and incubated at 4 °C overnight with anti-SNAP25 (1:2000, Proteintech 14903-1-AP), anti-RUNDC3A (1:1000, Proteintech 20531-1-AP), anti-GAPDH (1:5000, CST 5174 T), anti-AKT (1:1000, CST 4685 S), anti-p-AKT s437 (1:1000, CST 4060 T), anti-CDK2 (1:1000, CST 18048 T), anti-CDK4 (1:1000, CST 12790 T), anti-CDK6 (1:1000, CST 13331 T), anti-cyclinD1 (1:1000, CST 55506 T), and anti-cyclinD3 (1:1000, CST 2936 T) antibodies. The cycloheximide (100 μM) used in the protein half-life study was purchased from Sigma–Aldrich. The protein signals were exposed and quantified using an Odyssey Imaging System (LI-COR). All experiments were repeated three times.

### Cell proliferation detection

According to the specific growth characteristics of each cell line, ECC10 and ECC12 cells were seeded at a density of 1 × 10^4^ cells/well in 96-well plates, while AGS cells were seeded at a density of 10^3^ cells/well in 96-well plates. Cells were exposed to 5-fluorouracil (Selleck), irinotecan (Selleck), paclitaxel (Selleck), cisplatin (Sigma–Aldrich) and oxaliplatin (Sigma–Aldrich). Cell viability was detected by MTT assay (Invitrogen).

### Apoptosis assay by flow cytometry

Fluorescein isothiocyanate (FITC)-Annexin V and propidium iodide (PI) double-staining was performed with a Dead Cell Apoptosis Kit (Invitrogen). The assay was conducted according to the manufacturer’s protocol.

### Statistical analysis

Data are presented as the mean ± SEM (standard error of the mean) of at least three independent preparations. All statistical analyses were performed using SPSS 19.0. Each experiment was conducted in triplicate, and the data are presented as the mean ± SEM unless otherwise stated. The variance between the groups was statistically compared. A two-tailed t test was used to compare the mean values, and *p < 0.05 indicated significant differences.

## Supplementary information

Supplementary Figures

Supplementary Tables

Original Data File

## Data Availability

The datasets generated and analysed in the current study are available in GEO [Accession number: GSE60052 and GSE43795] and Array Express [Accession number: E-GEOD-6272].
