# Supplementary material for: RUNDC3A regulates SNAP25-mediated chemotherapy resistance by binding AKT in gastric neuroendocrine carcinoma (GNEC)
Source: Cell Death Discov. 2022 Jun 25;8:296. doi: 10.1038/s41420-022-01084-4 (PMC9233710; doi:10.1038/s41420-022-01084-4)
Supplement: Supplementary file 1 — Supplementary Figures [file 41420_2022_1084_MOESM1_ESM.docx]

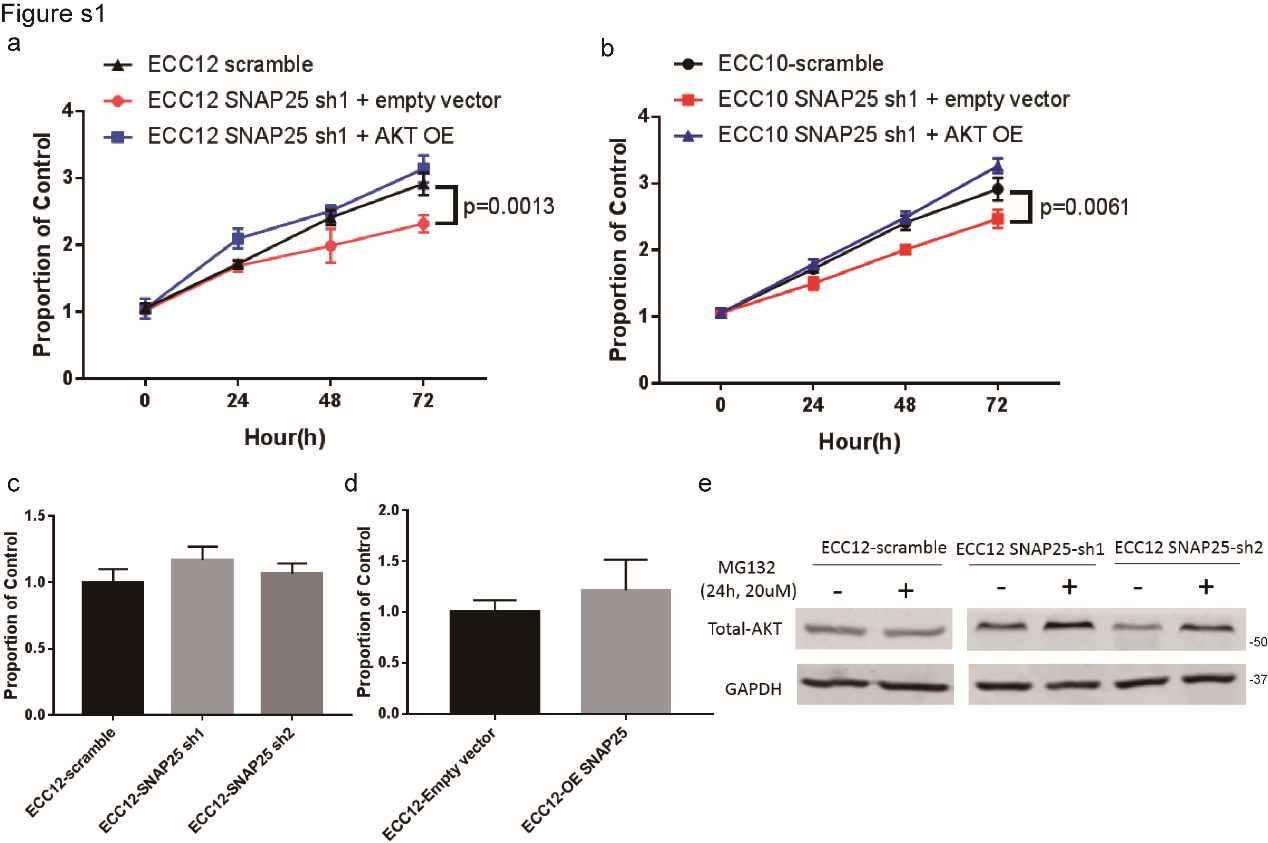


Supplementary Figure 1. (a, b) The proliferation detection in SNAP25 knockdown GNEC cell lines with AKT rescue. (c, d) AKT mRNA level detection in SNAP25 knockdown GNEC cell lines. (e) AKT protein change in SNAP25 knockdown cell lines with /without proteasome inhibitor MG132 treatment.

Figure s2


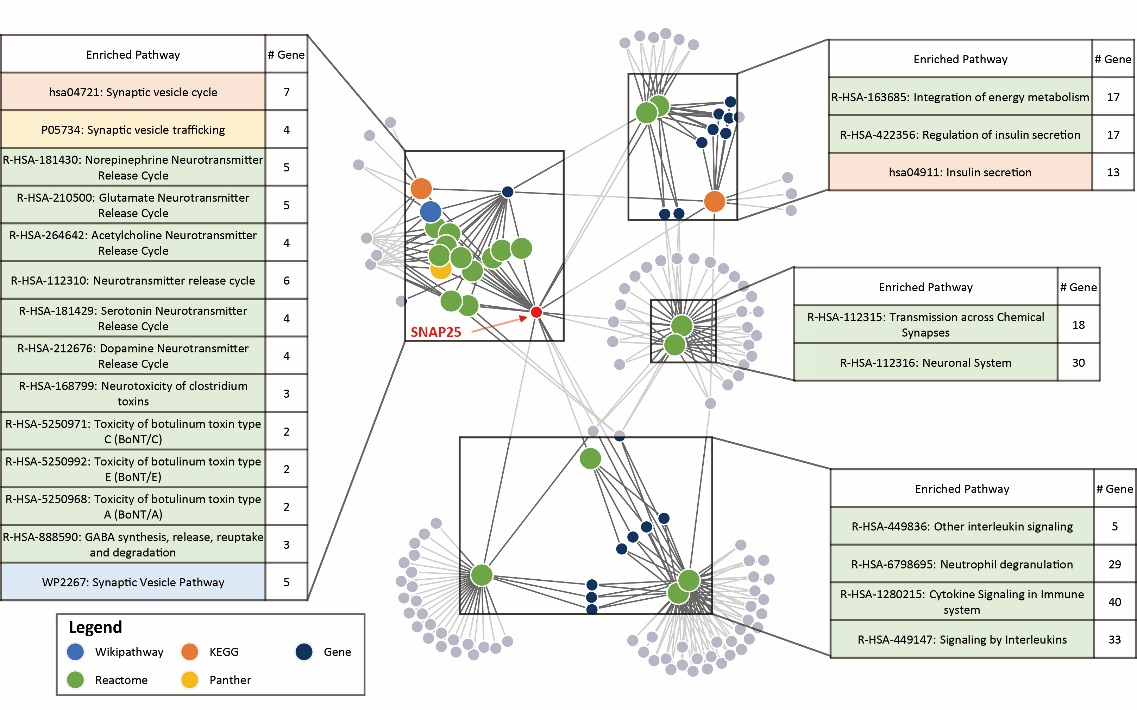


Supplementary Figure 2. Pathways significantly enriched in PNET, SINEC, and SCLC in association of gene SNAP25.

Figure s3


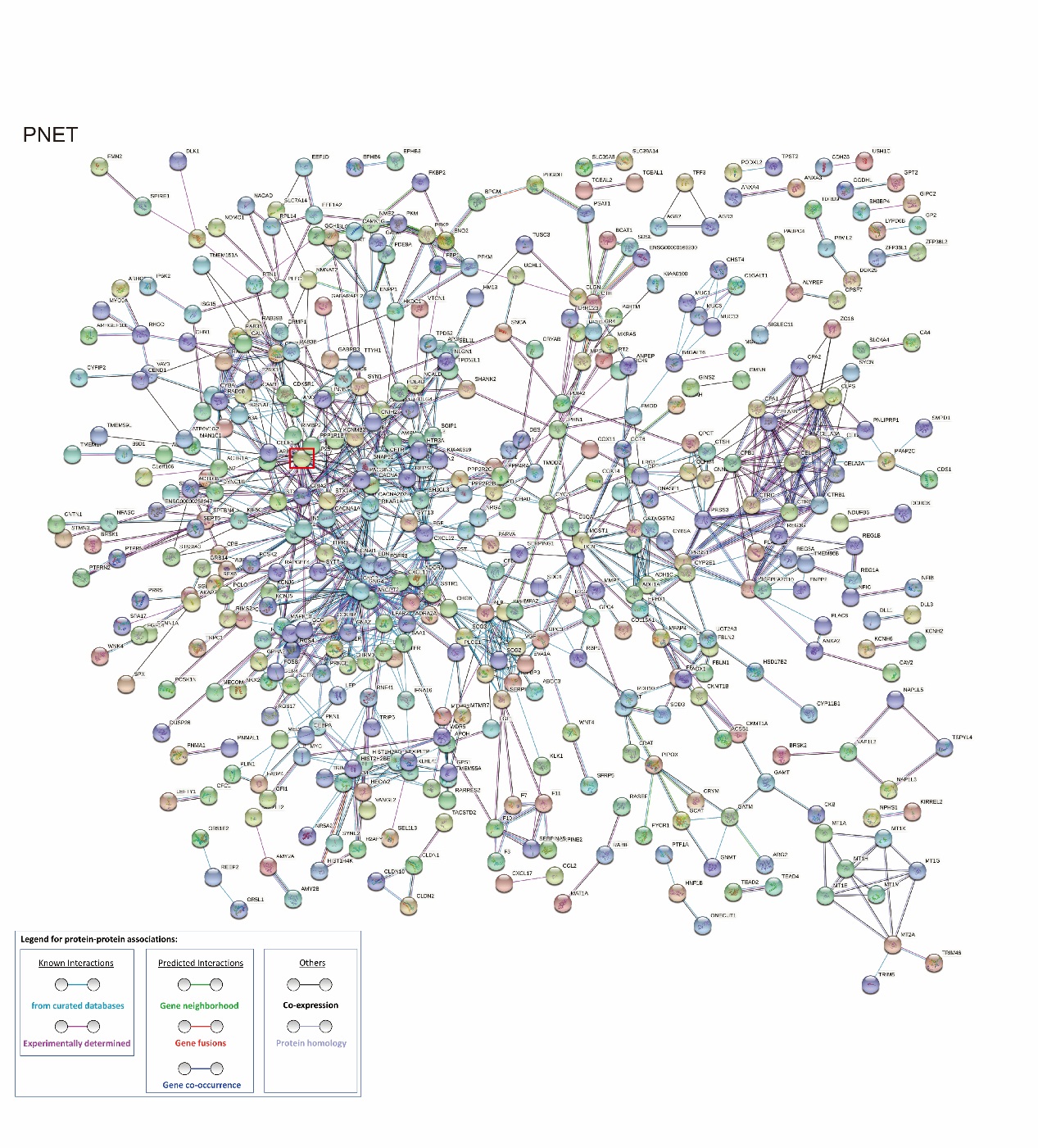


Supplementary Figure 3. PNET DEGs related PPI analysis

Figure s4


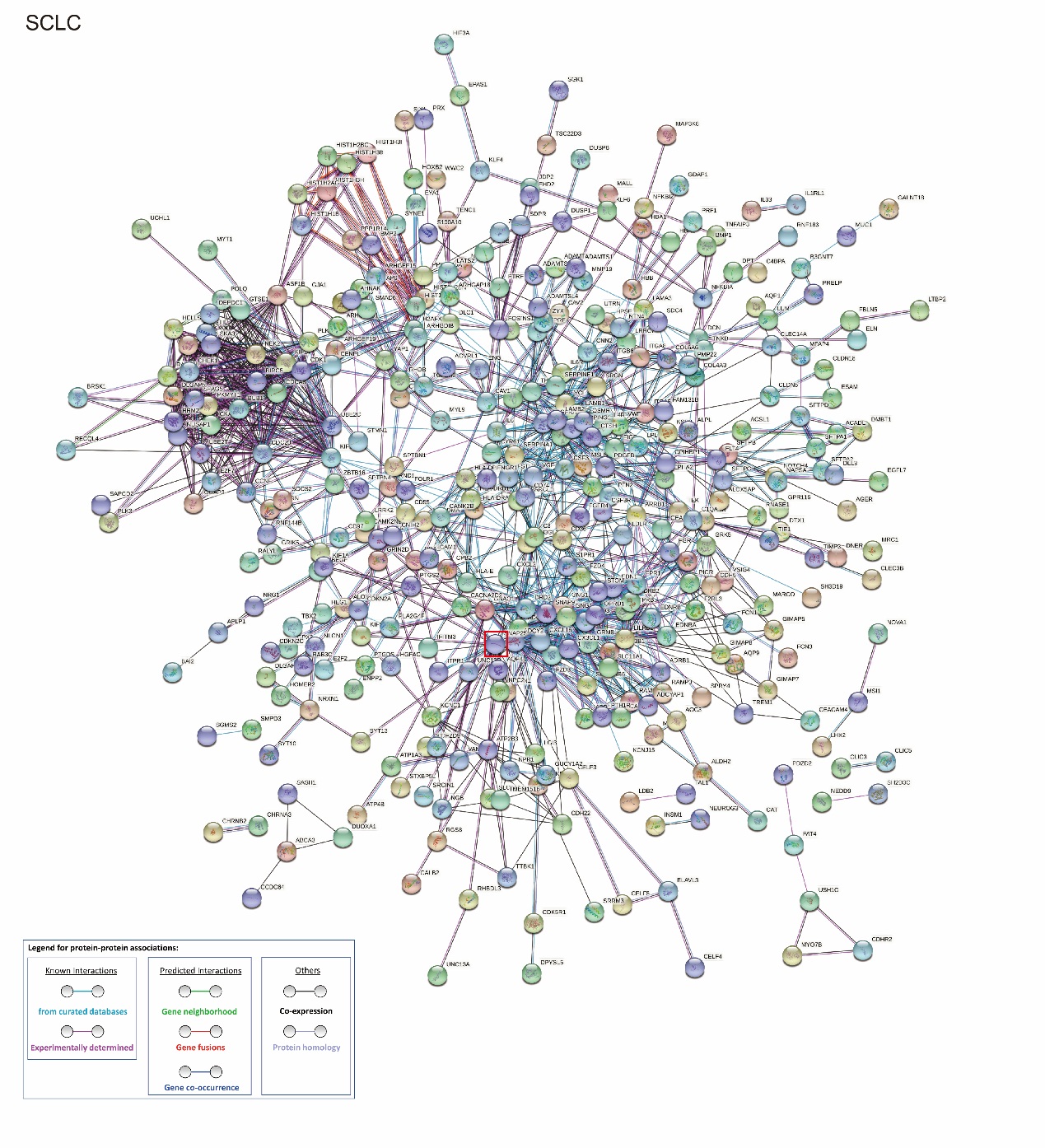


Supplementary Figure 4. SCLC DEGs related PPI analysis

Figure s5


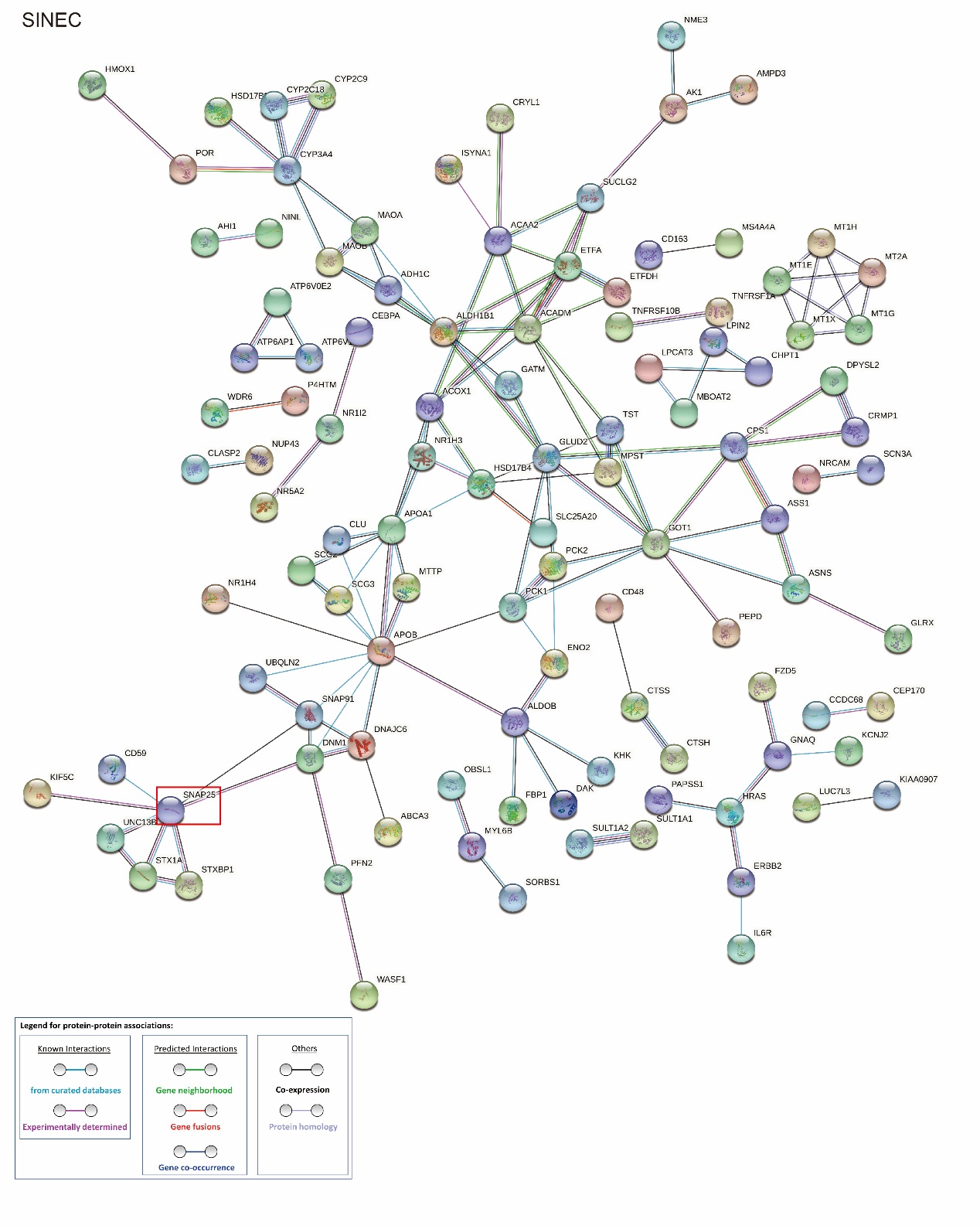


Supplementary Figure 5. SINEC DEGs related PPI analysis

Figure s6


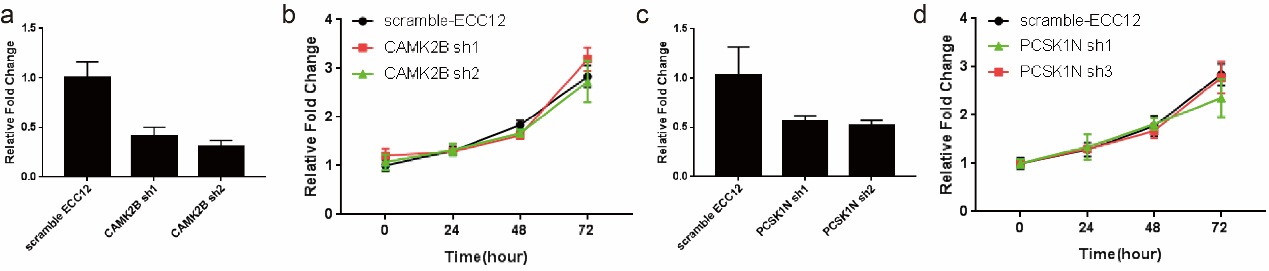


Supplementary Figure 6. The knockdown effect of CAMK2B and PCSK1N in GNEC cell line ECC12. (a, c) The target mRNA level in stable knockdown CAMK2B and PCSK1N cell lines. (b, d) The proliferation effect in stable knockdown CAMK2B and PCSK1N cell lines.


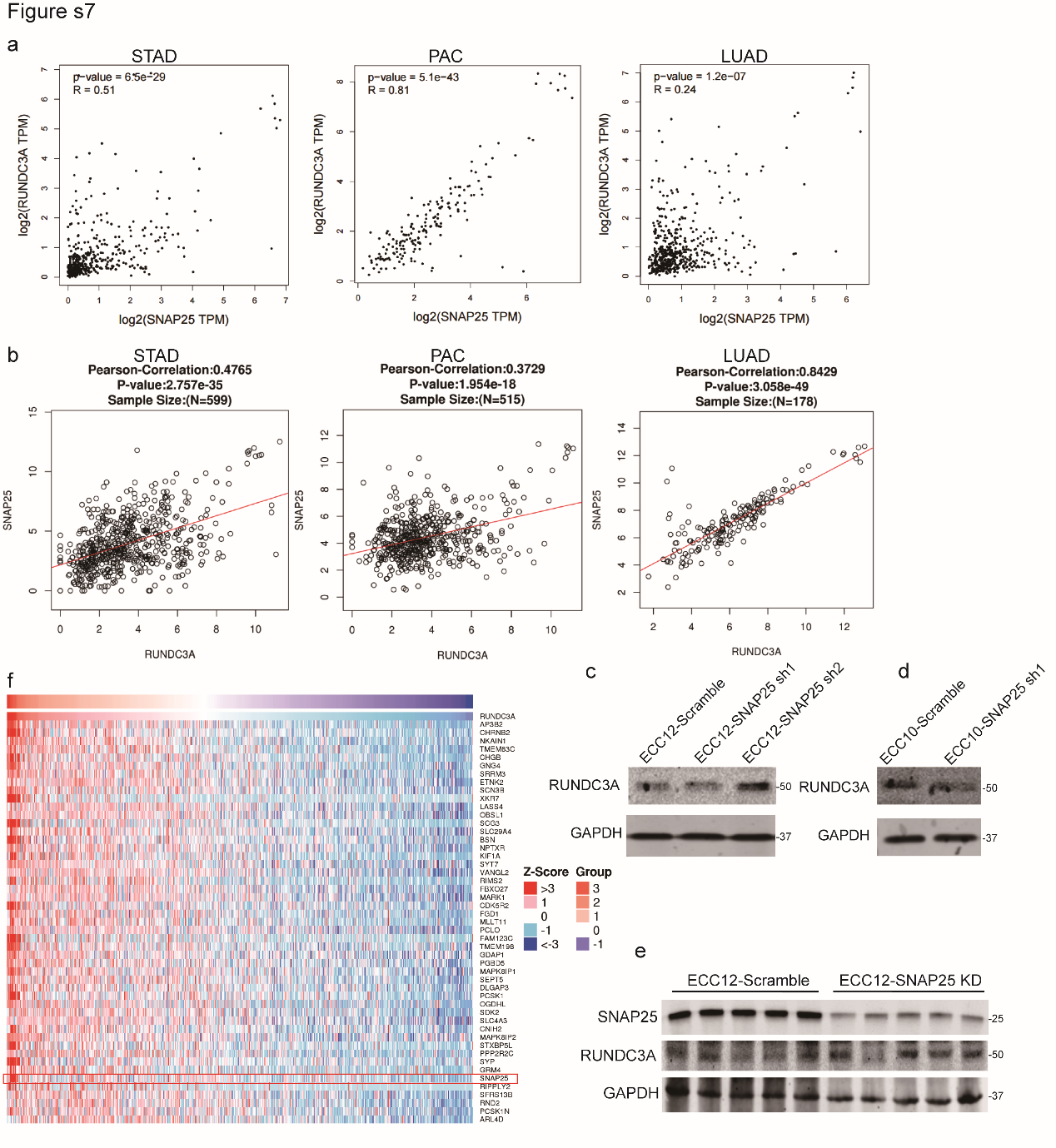


Supplementary Figure 7. (a, b) The expression correlation between SNAP25 and RUNDC3A in STAD, PAC and LUAD, the data used in (a) were the corresponding TCGA samples archived in GEPIA, and in (b) were TCGA data archived in LinkedOmics. (c-e) RUNDC3A expression change in SNAP25 knockdown GNEC cell lines and tumor sample of xenograft model. (f) The RUNDC3A and SNAP25 expression levels from 599 gastric patients were downloaded from TCGA project archived in LinkedOmics.
