# Supplementary material for: RUNDC3A regulates SNAP25-mediated chemotherapy resistance by binding AKT in gastric neuroendocrine carcinoma (GNEC)
Source: Cell Death Discov. 2022 Jun 25;8:296. doi: 10.1038/s41420-022-01084-4 (PMC9233710; doi:10.1038/s41420-022-01084-4)
Supplement: Supplementary file 2 — Supplementary Tables [file 41420_2022_1084_MOESM2_ESM.docx]

| **Table S1: Differentially Expressed Genes in GNEC** | | | |
| --- | --- | --- | --- |
|  |  |  |  |
| **Name** | **ENSEMBL_ID** | **Gene Symbol** | **Expression** |
| transmembrane protein 176A(TMEM176A) | ENSG00000002933 | TMEM176A | UP |
| ATP binding cassette subfamily C member 8(ABCC8) | ENSG00000006071 | ABCC8 | UP |
| mitogen-activated protein kinase 8 interacting protein 2(MAPK8IP2) | ENSG00000008735 | MAPK8IP2 | UP |
| NME1-NME2 readthrough(NME1-NME2) | ENSG00000011052 | NME1-NME2 | UP |
| synaptotagmin 7(SYT7) | ENSG00000011347 | SYT7 | UP |
| solute carrier family 25 member 39(SLC25A39) | ENSG00000013306 | SLC25A39 | UP |
| transforming acidic coiled-coil containing protein 3(TACC3) | ENSG00000013810 | TACC3 | UP |
| zinc finger CCCH-type containing 3(ZC3H3) | ENSG00000014164 | ZC3H3 | UP |
| dipeptidase 1 (renal)(DPEP1) | ENSG00000015413 | DPEP1 | UP |
| pleckstrin homology domain containing B1(PLEKHB1) | ENSG00000021300 | PLEKHB1 | UP |
| RTEL1-TNFRSF6B readthrough (NMD candidate)(RTEL1-TNFRSF6B) | ENSG00000026036 | RTEL1-TNFRSF6B | UP |
| myocilin (MYOC) | ENSG00000034971 | MYOC | DOWN |
| solute carrier family 18 member A1(SLC18A1) | ENSG00000036565 | SLC18A1 | UP |
| forkhead box P3(FOXP3) | ENSG00000049768 | FOXP3 | UP |
| potassium voltage-gated channel subfamily H member 2(KCNH2) | ENSG00000055118 | KCNH2 | UP |
| RIMS binding protein 2(RIMBP2) | ENSG00000060709 | RIMBP2 | UP |
| diacylglycerol O-acyltransferase 2(DGAT2) | ENSG00000062282 | DGAT2 | UP |
| DNA polymerase delta 1, catalytic subunit(POLD1) | ENSG00000062822 | POLD1 | UP |
| ATP binding cassette subfamily A member 7(ABCA7) | ENSG00000064687 | ABCA7 | UP |
| ATPase phospholipid transporting 11A(ATP11A) | ENSG00000068650 | ATP11A | UP |
| microRNA 8085(MIR8085) | ENSG00000069399 | MIR8085 | UP |
| potassium voltage-gated channel subfamily A regulatory beta subunit 2(KCNAB2) | ENSG00000069424 | KCNAB2 | UP |
| ZXD family zinc finger C(ZXDC) | ENSG00000070476 | ZXDC | UP |
| microRNA 939(MIR939) | ENSG00000071894 | MIR939 | UP |
| cytochrome b reductase 1 (CYBRD1) | ENSG00000071967 | CYBRD1 | DOWN |
| scavenger receptor class B member 1(SCARB1) | ENSG00000073060 | SCARB1 | UP |
| minichromosome maintenance complex component 2(MCM2) | ENSG00000073111 | MCM2 | UP |
| WD repeat domain 62(WDR62) | ENSG00000075702 | WDR62 | UP |
| lipase E, hormone sensitive type(LIPE) | ENSG00000079435 | LIPE | UP |
| platelet activating factor acetylhydrolase 1b catalytic subunit 3(PAFAH1B3) | ENSG00000079462 | PAFAH1B3 | UP |
| acetoacetyl-CoA synthetase(AACS) | ENSG00000081760 | AACS | UP |
| RAD54-like (S. cerevisiae)(RAD54L) | ENSG00000085999 | RAD54L | UP |
| aurora kinase A(AURKA) | ENSG00000087586 | AURKA | UP |
| sulfotransferase family 2B member 1(SULT2B1) | ENSG00000088002 | SULT2B1 | UP |
| p53 and DNA damage regulated 1(PDRG1) | ENSG00000088356 | PDRG1 | UP |
| tescalcin(TESC) | ENSG00000088992 | TESC | UP |
| anaphase promoting complex subunit 5(ANAPC5) | ENSG00000089053 | ANAPC5 | UP |
| chromogranin B(CHGB) | ENSG00000089199 | CHGB | UP |
| GRAM domain containing 1A(GRAMD1A) | ENSG00000089351 | GRAMD1A | UP |
| delta like canonical Notch ligand 3(DLL3) | ENSG00000090932 | DLL3 | UP |
| progastricsin (PGC) | ENSG00000096088 | PGC | DOWN |
| matrix metallopeptidase 11(MMP11) | ENSG00000099953 | MMP11 | UP |
| MYB proto-oncogene like 2(MYBL2) | ENSG00000101057 | MYBL2 | UP |
| spalt like transcription factor 4(SALL4) | ENSG00000101115 | SALL4 | UP |
| cell division cycle 25B(CDC25B) | ENSG00000101224 | CDC25B | UP |
| tribbles pseudokinase 3(TRIB3) | ENSG00000101255 | TRIB3 | UP |
| E2F transcription factor 1(E2F1) | ENSG00000101412 | E2F1 | UP |
| cystatin C(CST3) | ENSG00000101439 | CST3 | UP |
| WAP four-disulfide core domain 2(WFDC2) | ENSG00000101443 | WFDC2 | UP |
| CUGBP, Elav-like family member 4(CELF4) | ENSG00000101489 | CELF4 | UP |
| synaptophysin(SYP) | ENSG00000102003 | SYP | UP |
| proprotein convertase subtilisin/kexin type 1 inhibitor(PCSK1N) | ENSG00000102109 | PCSK1N | UP |
| par-6 family cell polarity regulator alpha(PARD6A) | ENSG00000102981 | PARD6A | UP |
| adaptor related protein complex 3 beta 2 subunit(AP3B2) | ENSG00000103723 | AP3B2 | UP |
| Bcl2 modifying factor(BMF) | ENSG00000104081 | BMF | UP |
| pyrroline-5-carboxylate reductase-like(PYCRL) | ENSG00000104524 | PYCRL | UP |
| RELB proto-oncogene, NF-kB subunit(RELB) | ENSG00000104856 | RELB | UP |
| ribonuclease H2 subunit A(RNASEH2A) | ENSG00000104889 | RNASEH2A | UP |
| cyclin E1(CCNE1) | ENSG00000105173 | CCNE1 | UP |
| translocase of inner mitochondrial membrane 50(TIMM50) | ENSG00000105197 | TIMM50 | UP |
| RNA polymerase II subunit I(POLR2I) | ENSG00000105258 | POLR2I | UP |
| solute carrier family 1 member 5(SLC1A5) | ENSG00000105281 | SLC1A5 | UP |
| microRNA 3191(MIR3191) | ENSG00000105327 | MIR3191 | UP |
| ribosomal protein S19(RPS19) | ENSG00000105372 | RPS19 | UP |
| ATPase Na+/K+ transporting subunit alpha 3(ATP1A3) | ENSG00000105409 | ATP1A3 | UP |
| DNA ligase 1(LIG1) | ENSG00000105486 | LIG1 | UP |
| MIER family member 2(MIER2) | ENSG00000105556 | MIER2 | UP |
| CREB regulated transcription coactivator 1(CRTC1) | ENSG00000105662 | CRTC1 | UP |
| syntaxin 1A(STX1A) | ENSG00000106089 | STX1A | UP |
| nudix hydrolase 1(NUDT1) | ENSG00000106268 | NUDT1 | UP |
| serpin family E member 1(SERPINE1) | ENSG00000106366 | SERPINE1 | UP |
| transmembrane protein 176B(TMEM176B) | ENSG00000106565 | TMEM176B | UP |
| SH2 domain containing adaptor protein B(SHB) | ENSG00000107338 | SHB | UP |
| nucleophosmin/nucleoplasmin 3(NPM3) | ENSG00000107833 | NPM3 | UP |
| neuralized E3 ubiquitin protein ligase 1(NEURL1) | ENSG00000107954 | NEURL1 | UP |
| ubiquitin conjugating enzyme E2 S(UBE2S) | ENSG00000108106 | UBE2S | UP |
| DnaJ heat shock protein family (Hsp40) member C12(DNAJC12) | ENSG00000108176 | DNAJC12 | UP |
| RUN domain containing 3A(RUNDC3A) | ENSG00000108309 | RUNDC3A | UP |
| vitronectin(VTN) | ENSG00000109072 | VTN | UP |
| coiled-coil domain containing 86(CCDC86) | ENSG00000110104 | CCDC86 | UP |
| polypeptide N-acetylgalactosaminyltransferase 18(GALNT18) | ENSG00000110328 | GALNT18 | UP |
| timeless circadian clock(TIMELESS) | ENSG00000111602 | TIMELESS | UP |
| cytochrome c oxidase subunit 6A1(COX6A1) | ENSG00000111775 | COX6A1 | UP |
| microRNA 711(MIR711) | ENSG00000114270 | MIR711 | UP |
| podocalyxin like 2(PODXL2) | ENSG00000114631 | PODXL2 | UP |
| ribosomal RNA processing 9, small subunit (SSU) processome component, homolog (yeast)(RRP9) | ENSG00000114767 | RRP9 | UP |
| rhotekin(RTKN) | ENSG00000114993 | RTKN | UP |
| centromere protein A(CENPA) | ENSG00000115163 | CENPA | UP |
| regenerating family member 1 alpha(REG1A) | ENSG00000115386 | REG1A | UP |
| AT-rich interaction domain 3A(ARID3A) | ENSG00000116017 | ARID3A | UP |
| sepiapterin reductase (7,8-dihydrobiopterin:NADP+ oxidoreductase)(SPR) | ENSG00000116096 | SPR | UP |
| spermidine synthase(SRM) | ENSG00000116649 | SRM | UP |
| MAD2 mitotic arrest deficient-like 2 (yeast)(MAD2L2) | ENSG00000116670 | MAD2L2 | UP |
| transmembrane protein 9(TMEM9) | ENSG00000116857 | TMEM9 | UP |
| microfibrillar associated protein 2(MFAP2) | ENSG00000117122 | MFAP2 | UP |
| cell division cycle 20(CDC20) | ENSG00000117399 | CDC20 | UP |
| artemin(ARTN) | ENSG00000117407 | ARTN | UP |
| ATPase H+ transporting V0 subunit b(ATP6V0B) | ENSG00000117410 | ATP6V0B | UP |
| transthyretin(TTR) | ENSG00000118271 | TTR | UP |
| placental growth factor(PGF) | ENSG00000119630 | PGF | UP |
| methylenetetrahydrofolate dehydrogenase (NADP+ dependent) 1-like(MTHFD1L) | ENSG00000120254 | MTHFD1L | UP |
| SMAD family member 9(SMAD9) | ENSG00000120693 | SMAD9 | UP |
| non-SMC condensin I complex subunit H(NCAPH) | ENSG00000121152 | NCAPH | UP |
| mitogen-activated protein kinase 8 interacting protein 1(MAPK8IP1) | ENSG00000121653 | MAPK8IP1 | UP |
| serine peptidase inhibitor, Kazal type 4(SPINK4) | ENSG00000122711 | SPINK4 | UP |
| microRNA 1178(MIR1178) | ENSG00000122966 | MIR1178 | UP |
| ssu-2 homolog (C. elegans)(SSUH2) | ENSG00000125046 | SSUH2 | UP |
| forkhead box A2(FOXA2) | ENSG00000125798 | FOXA2 | UP |
| D-tyrosyl-tRNA deacylase 1(DTD1) | ENSG00000125821 | DTD1 | UP |
| ribosome binding protein 1(RRBP1) | ENSG00000125844 | RRBP1 | UP |
| transmembrane protein 74B(TMEM74B) | ENSG00000125895 | TMEM74B | UP |
| free fatty acid receptor 2(FFAR2) | ENSG00000126262 | FFAR2 | UP |
| dual specificity tyrosine phosphorylation regulated kinase 2(DYRK2) | ENSG00000127334 | DYRK2 | UP |
| protein kinase, membrane associated tyrosine/threonine 1(PKMYT1) | ENSG00000127564 | PKMYT1 | UP |
| G protein subunit alpha z(GNAZ) | ENSG00000128266 | GNAZ | UP |
| VGF nerve growth factor inducible(VGF) | ENSG00000128564 | VGF | UP |
| mitochondrial ribosomal protein S12(MRPS12) | ENSG00000128626 | MRPS12 | UP |
| glutamate decarboxylase 1(GAD1) | ENSG00000128683 | GAD1 | UP |
| delta like canonical Notch ligand 4(DLL4) | ENSG00000128917 | DLL4 | UP |
| ceroid-lipofuscinosis, neuronal 6, late infantile, variant(CLN6) | ENSG00000128973 | CLN6 | UP |
| tryptophan hydroxylase 1(TPH1) | ENSG00000129167 | TPH1 | UP |
| family with sequence similarity 64 member A(FAM64A) | ENSG00000129195 | FAM64A | UP |
| SHC adaptor protein 2(SHC2) | ENSG00000129946 | SHC2 | UP |
| family with sequence similarity 155 member B(FAM155B) | ENSG00000130054 | FAM155B | UP |
| thioesterase superfamily member 6(THEM6) | ENSG00000130193 | THEM6 | UP |
| apolipoprotein C1(APOC1) | ENSG00000130208 | APOC1 | UP |
| kinesin family member 1A(KIF1A) | ENSG00000130294 | KIF1A | UP |
| unc-13 homolog A(UNC13A) | ENSG00000130477 | UNC13A | UP |
| growth differentiation factor 15(GDF15) | ENSG00000130513 | GDF15 | UP |
| microRNA 675(MIR675) | ENSG00000130600 | MIR675 | UP |
| motor neuron and pancreas homeobox 1(MNX1) | ENSG00000130675 | MNX1 | UP |
| transmembrane protein 160(TMEM160) | ENSG00000130748 | TMEM160 | UP |
| mitogen-activated protein kinase kinase kinase 10(MAP3K10) | ENSG00000130758 | MAP3K10 | UP |
| peter pan homolog (Drosophila)(PPAN) | ENSG00000130810 | PPAN | UP |
| UL16 binding protein 2(ULBP2) | ENSG00000131015 | ULBP2 | UP |
| zinc finger protein 428(ZNF428) | ENSG00000131116 | ZNF428 | UP |
| GINS complex subunit 2(GINS2) | ENSG00000131153 | GINS2 | UP |
| proline rich 7, synaptic(PRR7) | ENSG00000131188 | PRR7 | UP |
| nuclear receptor subfamily 0 group B member 2(NR0B2) | ENSG00000131910 | NR0B2 | UP |
| zinc finger SWIM-type containing 4(ZSWIM4) | ENSG00000132003 | ZSWIM4 | UP |
| F-box and WD repeat domain containing 9(FBXW9) | ENSG00000132004 | FBXW9 | UP |
| nucleoporin 210(NUP210) | ENSG00000132182 | NUP210 | UP |
| synaptosome associated protein 25(SNAP25) | ENSG00000132639 | SNAP25 | UP |
| nuclear transport factor 2 like export factor 1(NXT1) | ENSG00000132661 | NXT1 | UP |
| chitinase 3 like 1(CHI3L1) | ENSG00000133048 | CHI3L1 | UP |
| brain expressed X-linked 2(BEX2) | ENSG00000133134 | BEX2 | UP |
| brain expressed X-linked 1(BEX1) | ENSG00000133169 | BEX1 | UP |
| lysine methyltransferase 5C(KMT5C) | ENSG00000133247 | KMT5C | UP |
| diacylglycerol lipase alpha(DAGLA) | ENSG00000134780 | DAGLA | UP |
| translocase of inner mitochondrial membrane 10(TIMM10) | ENSG00000134809 | TIMM10 | UP |
| DExH-box helicase 34(DHX34) | ENSG00000134815 | DHX34 | UP |
| serine dehydratase(SDS) | ENSG00000135094 | SDS | UP |
| musashi RNA binding protein 1(MSI1) | ENSG00000135097 | MSI1 | UP |
| BICD family like cargo adaptor 1(BICDL1) | ENSG00000135127 | BICDL1 | UP |
| cyclin dependent kinase 4(CDK4) | ENSG00000135446 | CDK4 | UP |
| trophinin associated protein(TROAP) | ENSG00000135451 | TROAP | UP |
| empty spiracles homeobox 1(EMX1) | ENSG00000135638 | EMX1 | UP |
| angiotensinogen(AGT) | ENSG00000135744 | AGT | UP |
| uncharacterized LOC101927181(LOC101927181) | ENSG00000136213 | LOC101927181 | UP |
| tweety family member 3(TTYH3) | ENSG00000136295 | TTYH3 | UP |
| immediate early response 3(IER3) | ENSG00000137331 | IER3 | UP |
| myosin VIIA(MYO7A) | ENSG00000137474 | MYO7A | UP |
| gamma-glutamyl hydrolase(GGH) | ENSG00000137563 | GGH | UP |
| FXYD domain containing ion transport regulator 2(FXYD2) | ENSG00000137731 | FXYD2 | UP |
| ADAM metallopeptidase with thrombospondin type 1 motif 14(ADAMTS14) | ENSG00000138316 | ADAMTS14 | UP |
| achaete-scute family bHLH transcription factor 1(ASCL1) | ENSG00000139352 | ASCL1 | UP |
| family with sequence similarity 222 member A(FAM222A) | ENSG00000139438 | FAM222A | UP |
| solute carrier family 7 member 1(SLC7A1) | ENSG00000139514 | SLC7A1 | UP |
| RAB15, member RAS oncogene family(RAB15) | ENSG00000139998 | RAB15 | UP |
| serpin family A member 10(SERPINA10) | ENSG00000140093 | SERPINA10 | UP |
| glucose-6-phosphatase catalytic subunit 3(G6PC3) | ENSG00000141349 | G6PC3 | UP |
| asialoglycoprotein receptor 1(ASGR1) | ENSG00000141505 | ASGR1 | UP |
| thioredoxin like 4A(TXNL4A) | ENSG00000141759 | TXNL4A | UP |
| solute carrier family 39 member 3(SLC39A3) | ENSG00000141873 | SLC39A3 | UP |
| tubulin polyglutamylase complex subunit 1(TPGS1) | ENSG00000141933 | TPGS1 | UP |
| phospholipid phosphatase 2(PLPP2) | ENSG00000141934 | PLPP2 | UP |
| salt inducible kinase 1(SIK1) | ENSG00000142178 | SIK1 | UP |
| transient receptor potential cation channel subfamily M member 2(TRPM2) | ENSG00000142185 | TRPM2 | UP |
|  | ENSG00000142327 |  | UP |
| cytosolic thiouridylase subunit 1(CTU1) | ENSG00000142544 | CTU1 | UP |
| kinesin family member 2C(KIF2C) | ENSG00000142945 | KIF2C | UP |
| transmembrane protein 61(TMEM61) | ENSG00000143001 | TMEM61 | UP |
| dermatopontin (DPT) | ENSG00000143196 | DPT | DOWN |
| prefoldin subunit 2(PFDN2) | ENSG00000143256 | PFDN2 | UP |
| regulator of G-protein signaling 16(RGS16) | ENSG00000143333 | RGS16 | UP |
| dual specificity phosphatase 10(DUSP10) | ENSG00000143507 | DUSP10 | UP |
| von Willebrand factor A domain containing 5B2(VWA5B2) | ENSG00000145198 | VWA5B2 | UP |
| complexin 2(CPLX2) | ENSG00000145920 | CPLX2 | UP |
| sidekick cell adhesion molecule 1(SDK1) | ENSG00000146555 | SDK1 | UP |
| cell division cycle associated 5(CDCA5) | ENSG00000146670 | CDCA5 | UP |
| calcium/calmodulin dependent protein kinase II beta(CAMK2B) | ENSG00000058404 | CAMK2B | UP |
| cyclin dependent kinase inhibitor 2A(CDKN2A) | ENSG00000147889 | CDKN2A | UP |
| chromosome 10 open reading frame 11(C10orf11) | ENSG00000148655 | C10orf11 | UP |
| kelch like family member 35(KLHL35) | ENSG00000149243 | KLHL35 | UP |
| inner centromere protein(INCENP) | ENSG00000149503 | INCENP | UP |
| chromosome 11 open reading frame 53(C11orf53) | ENSG00000150750 | C11orf53 | UP |
| DEAH-box helicase 37(DHX37) | ENSG00000150990 | DHX37 | UP |
| protein tyrosine phosphatase, receptor type N2(PTPRN2) | ENSG00000155093 | PTPRN2 | UP |
| interleukin 34(IL34) | ENSG00000157368 | IL34 | UP |
| calcium voltage-gated channel subunit alpha1 D(CACNA1D) | ENSG00000157388 | CACNA1D | UP |
| tetraspanin 18(TSPAN18) | ENSG00000157570 | TSPAN18 | UP |
| rhophilin Rho GTPase binding protein 1(RHPN1) | ENSG00000158106 | RHPN1 | UP |
| glycerophosphodiester phosphodiesterase domain containing 5(GDPD5) | ENSG00000158555 | GDPD5 | UP |
| nucleophosmin/nucleoplasmin 2(NPM2) | ENSG00000158806 | NPM2 | UP |
| apolipoprotein A2(APOA2) | ENSG00000158874 | APOA2 | UP |
| adhesion G protein-coupled receptor G5(ADGRG5) | ENSG00000159618 | ADGRG5 | UP |
| spondin 2(SPON2) | ENSG00000159674 | SPON2 | UP |
| ATP binding cassette subfamily G member 1(ABCG1) | ENSG00000160179 | ABCG1 | UP |
| SH3KBP1 binding protein 1(SHKBP1) | ENSG00000160410 | SHKBP1 | UP |
| cholinergic receptor nicotinic beta 2 subunit(CHRNB2) | ENSG00000160716 | CHRNB2 | UP |
| neurobeachin like 2(NBEAL2) | ENSG00000160796 | NBEAL2 | UP |
| protein phosphatase 1 regulatory subunit 35(PPP1R35) | ENSG00000160813 | PPP1R35 | UP |
| fibroblast growth factor receptor 4(FGFR4) | ENSG00000160867 | FGFR4 | UP |
| lymphocyte antigen 6 complex, locus E(LY6E) | ENSG00000160932 | LY6E | UP |
| tonsoku-like, DNA repair protein(TONSL) | ENSG00000160949 | TONSL | UP |
| RecQ like helicase 4(RECQL4) | ENSG00000160957 | RECQL4 | UP |
| major facilitator superfamily domain containing 12(MFSD12) | ENSG00000161091 | MFSD12 | UP |
| ER membrane protein complex subunit 10(EMC10) | ENSG00000161671 | EMC10 | UP |
| DBF4 zinc finger B(DBF4B) | ENSG00000161692 | DBF4B | UP |
| general receptor for phosphoinositides 1 associated scaffold protein(GRASP) | ENSG00000161835 | GRASP | UP |
| chromosome 16 open reading frame 59(C16orf59) | ENSG00000162062 | C16orf59 | UP |
| progestin and adipoQ receptor family member 4(PAQR4) | ENSG00000162073 | PAQR4 | UP |
| ClpB homolog, mitochondrial AAA ATPase chaperonin(CLPB) | ENSG00000162129 | CLPB | UP |
| LDL receptor related protein 5(LRP5) | ENSG00000162337 | LRP5 | UP |
| coiled-coil domain containing 74A(CCDC74A) | ENSG00000163040 | CCDC74A | UP |
| NAD(P)HX epimerase(NAXE) | ENSG00000163382 | NAXE | UP |
| histone deacetylase 11(HDAC11) | ENSG00000163517 | HDAC11 | UP |
| neurofascin(NFASC) | ENSG00000163531 | NFASC | UP |
| calcium dependent secretion activator(CADPS) | ENSG00000163618 | CADPS | UP |
| transmembrane protein adipocyte associated 1(TPRA1) | ENSG00000163870 | TPRA1 | UP |
| hes related family bHLH transcription factor with YRPW motif-like(HEYL) | ENSG00000163909 | HEYL | UP |
| melanotransferrin(MELTF) | ENSG00000163975 | MELTF | UP |
| dishevelled binding antagonist of beta catenin 2(DACT2) | ENSG00000164488 | DACT2 | UP |
| pituitary tumor-transforming 1(PTTG1) | ENSG00000164611 | PTTG1 | UP |
| RELT like 2(RELL2) | ENSG00000164620 | RELL2 | UP |
| solute carrier family 29 member 4(SLC29A4) | ENSG00000164638 | SLC29A4 | UP |
| hes related family bHLH transcription factor with YRPW motif 1(HEY1) | ENSG00000164683 | HEY1 | UP |
| collagen triple helix repeat containing 1(CTHRC1) | ENSG00000164932 | CTHRC1 | UP |
| claudin 3(CLDN3) | ENSG00000165215 | CLDN3 | UP |
| aquaporin 3 (Gill blood group)(AQP3) | ENSG00000165272 | AQP3 | UP |
| transmembrane protein 63C(TMEM63C) | ENSG00000165548 | TMEM63C | UP |
| butyrophilin like 9(BTNL9) | ENSG00000165810 | BTNL9 | UP |
| complement C2(C2) | ENSG00000166278 | C2 | UP |
| SEC11 homolog C, signal peptidase complex subunit(SEC11C) | ENSG00000166562 | SEC11C | UP |
| mesoderm posterior bHLH transcription factor 1(MESP1) | ENSG00000166823 | MESP1 | UP |
| chromatin licensing and DNA replication factor 1(CDT1) | ENSG00000167513 | CDT1 | UP |
| transmembrane protein 145(TMEM145) | ENSG00000167619 | TMEM145 | UP |
| kinesin family member C2(KIFC2) | ENSG00000167702 | KIFC2 | UP |
| serpin family F member 2(SERPINF2) | ENSG00000167711 | SERPINF2 | UP |
| chromosome 19 open reading frame 48(C19orf48) | ENSG00000167747 | C19orf48 | UP |
| RAB26, member RAS oncogene family(RAB26) | ENSG00000167964 | RAB26 | UP |
| chromosome 11 open reading frame 84(C11orf84) | ENSG00000168005 | C11orf84 | UP |
| SAC3 domain containing 1(SAC3D1) | ENSG00000168061 | SAC3D1 | UP |
| mitogen-activated protein kinase kinase kinase kinase 2(MAP4K2) | ENSG00000168067 | MAP4K2 | UP |
| ring finger protein 187(RNF187) | ENSG00000168159 | RNF187 | UP |
| G protein subunit gamma 4(GNG4) | ENSG00000168243 | GNG4 | UP |
| 5'-nucleotidase domain containing 2(NT5DC2) | ENSG00000168268 | NT5DC2 | UP |
| complexin 1(CPLX1) | ENSG00000168993 | CPLX1 | UP |
| carnitine palmitoyltransferase 1C(CPT1C) | ENSG00000169169 | CPT1C | UP |
| collagen type XXII alpha 1 chain(COL22A1) | ENSG00000169436 | COL22A1 | UP |
| solute carrier family 38 member 11(SLC38A11) | ENSG00000169507 | SLC38A11 | UP |
| ASPSCR1, UBX domain containing tether for SLC2A4(ASPSCR1) | ENSG00000169696 | ASPSCR1 | UP |
| CKLF like MARVEL transmembrane domain containing 8(CMTM8) | ENSG00000170293 | CMTM8 | UP |
| fatty acid binding protein 4 (FABP4) | ENSG00000170323 | FABP4 | DOWN |
| cystatin SN(CST1) | ENSG00000170373 | CST1 | UP |
| zinc finger protein 296(ZNF296) | ENSG00000170684 | ZNF296 | UP |
| homeobox B9(HOXB9) | ENSG00000170689 | HOXB9 | UP |
| MTSS1, I-BAR domain containing(MTSS1) | ENSG00000170873 | MTSS1 | UP |
| t-SNARE domain containing 1(TSNARE1) | ENSG00000171045 | TSNARE1 | UP |
| potassium two pore domain channel subfamily K member 3(KCNK3) | ENSG00000171303 | KCNK3 | UP |
| cyclin dependent kinase 5 regulatory subunit 2(CDK5R2) | ENSG00000171450 | CDK5R2 | UP |
| pyrimidinergic receptor P2Y6(P2RY6) | ENSG00000171631 | P2RY6 | UP |
| phenylalanine hydroxylase(PAH) | ENSG00000171759 | PAH | UP |
| regenerating family member 3 alpha(REG3A) | ENSG00000172016 | REG3A | UP |
| regenerating family member 1 beta(REG1B) | ENSG00000172023 | REG1B | UP |
| CCAAT/enhancer binding protein beta(CEBPB) | ENSG00000172216 | CEBPB | UP |
| Rho family GTPase 1(RND1) | ENSG00000172602 | RND1 | UP |
| SH3 domain containing ring finger 3(SH3RF3) | ENSG00000172985 | SH3RF3 | UP |
| INSM transcriptional repressor 1(INSM1) | ENSG00000173404 | INSM1 | UP |
| protein phosphatase 1 regulatory inhibitor subunit 14B(PPP1R14B) | ENSG00000173457 | PPP1R14B | UP |
| potassium voltage-gated channel subfamily H member 6(KCNH6) | ENSG00000173826 | KCNH6 | UP |
| polo like kinase 3(PLK3) | ENSG00000173846 | PLK3 | UP |
| chromobox 2(CBX2) | ENSG00000173894 | CBX2 | UP |
| homeobox B2(HOXB2) | ENSG00000173917 | HOXB2 | UP |
| zinc finger HIT-type containing 2(ZNHIT2) | ENSG00000174276 | ZNHIT2 | UP |
| seizure related 6 homolog like 2(SEZ6L2) | ENSG00000174938 | SEZ6L2 | UP |
| aspartate beta-hydroxylase domain containing 1(ASPHD1) | ENSG00000174939 | ASPHD1 | UP |
| ubiquitin conjugating enzyme E2 C(UBE2C) | ENSG00000175063 | UBE2C | UP |
| proprotein convertase subtilisin/kexin type 1(PCSK1) | ENSG00000175426 | PCSK1 | UP |
| DR1 associated protein 1(DRAP1) | ENSG00000175550 | DRAP1 | UP |
| uncoupling protein 2(UCP2) | ENSG00000175567 | UCP2 | UP |
| ETS variant 4(ETV4) | ENSG00000175832 | ETV4 | UP |
| EP300 interacting inhibitor of differentiation 2(EID2) | ENSG00000176396 | EID2 | UP |
| solute carrier organic anion transporter family member 3A1(SLCO3A1) | ENSG00000176463 | SLCO3A1 | UP |
| microRNA 7108(MIR7108) | ENSG00000176619 | MIR7108 | UP |
| MIR7-3 host gene(MIR7-3HG) | ENSG00000176840 | MIR7-3HG | UP |
| PTPRF interacting protein alpha 3(PPFIA3) | ENSG00000177380 | PPFIA3 | UP |
| TGFB induced factor homeobox 1(TGIF1) | ENSG00000177426 | TGIF1 | UP |
| solute carrier family 25 member 22(SLC25A22) | ENSG00000177542 | SLC25A22 | UP |
| serine/arginine repetitive matrix 3(SRRM3) | ENSG00000177679 | SRRM3 | UP |
| SRY-box 12(SOX12) | ENSG00000177732 | SOX12 | UP |
| carbonic anhydrase 8(CA8) | ENSG00000178538 | CA8 | UP |
| poly(ADP-ribose) polymerase family member 10(PARP10) | ENSG00000178685 | PARP10 | UP |
| ribonuclease P/MRP subunit p25(RPP25) | ENSG00000178718 | RPP25 | UP |
| copine 7(CPNE7) | ENSG00000178773 | CPNE7 | UP |
| microRNA 6847(MIR6847) | ENSG00000178896 | MIR6847 | UP |
| hydroxypyruvate isomerase (putative)(HYI) | ENSG00000178922 | HYI | UP |
| exocyst complex component 3 like 1(EXOC3L1) | ENSG00000179044 | EXOC3L1 | UP |
| cytochrome c1(CYC1) | ENSG00000179091 | CYC1 | UP |
| transmembrane protein 151A(TMEM151A) | ENSG00000179292 | TMEM151A | UP |
| von Willebrand factor A domain containing 1(VWA1) | ENSG00000179403 | VWA1 | UP |
| poly(U) binding splicing factor 60(PUF60) | ENSG00000179950 | PUF60 | UP |
| basic helix-loop-helix family member a15(BHLHA15) | ENSG00000180535 | BHLHA15 | UP |
| olfactory receptor family 51 subfamily E member 1(OR51E1) | ENSG00000180785 | OR51E1 | UP |
| homeobox C9(HOXC9) | ENSG00000180806 | HOXC9 | UP |
| homeobox C10(HOXC10) | ENSG00000180818 | HOXC10 | UP |
| microRNA 937(MIR937) | ENSG00000180900 | MIR937 | UP |
| pleckstrin homology like domain family A member 2(PHLDA2) | ENSG00000181649 | PHLDA2 | UP |
| ADP ribosylation factor like GTPase 6 interacting protein 4(ARL6IP4) | ENSG00000182196 | ARL6IP4 | UP |
| serine hydroxymethyltransferase 2(SHMT2) | ENSG00000182199 | SHMT2 | UP |
| F-box and leucine rich repeat protein 6(FBXL6) | ENSG00000182325 | FBXL6 | UP |
| lipase F, gastric type (LIPF) | ENSG00000182333 | LIPF | DOWN |
| EPH receptor B3(EPHB3) | ENSG00000182580 | EPHB3 | UP |
| long intergenic non-protein coding RNA 1006(LINC01006) | ENSG00000182648 | LINC01006 | UP |
| C2 calcium dependent domain containing 4C(C2CD4C) | ENSG00000183186 | C2CD4C | UP |
| poly(rC) binding protein 3(PCBP3) | ENSG00000183570 | PCBP3 | UP |
| DiGeorge syndrome critical region gene 6(DGCR6) | ENSG00000183628 | DGCR6 | UP |
| achaete-scute family bHLH transcription factor 2(ASCL2) | ENSG00000183734 | ASCL2 | UP |
| TRAF interacting protein(TRAIP) | ENSG00000183763 | TRAIP | UP |
| neuropeptide W(NPW) | ENSG00000183971 | NPW | UP |
| adrenoceptor alpha 2C(ADRA2C) | ENSG00000184160 | ADRA2C | UP |
| H1 histone family member X(H1FX) | ENSG00000184897 | H1FX | UP |
| regulatory factor X6(RFX6) | ENSG00000185002 | RFX6 | UP |
| mannosidase endo-alpha like(MANEAL) | ENSG00000185090 | MANEAL | UP |
| suppressor of cytokine signaling 1(SOCS1) | ENSG00000185338 | SOCS1 | UP |
| SH3 and cysteine rich domain 3(STAC3) | ENSG00000185482 | STAC3 | UP |
| preferentially expressed antigen in melanoma(PRAME) | ENSG00000185686 | PRAME | UP |
| solute carrier family 52 member 2(SLC52A2) | ENSG00000185803 | SLC52A2 | UP |
| keratin associated protein 5-5(KRTAP5-5) | ENSG00000185940 | KRTAP5-5 | UP |
| kinesin family member 18B(KIF18B) | ENSG00000186185 | KIF18B | UP |
| carcinoembryonic antigen related cell adhesion molecule 19(CEACAM19) | ENSG00000186567 | CEACAM19 | UP |
| TNF receptor superfamily member 4(TNFRSF4) | ENSG00000186827 | TNFRSF4 | UP |
| cholecystokinin(CCK) | ENSG00000187094 | CCK | UP |
| potassium voltage-gated channel subfamily J member 11(KCNJ11) | ENSG00000187486 | KCNJ11 | UP |
| Ras and Rab interactor like(RINL) | ENSG00000187994 | RINL | UP |
| HEPACAM family member 2(HEPACAM2) | ENSG00000188175 | HEPACAM2 | UP |
| uncharacterized LOC25845(PP7080) | ENSG00000188242 | PP7080 | UP |
| hes family bHLH transcription factor 4(HES4) | ENSG00000188290 | HES4 | UP |
| H2A histone family member X(H2AFX) | ENSG00000188486 | H2AFX | UP |
|  | ENSG00000188573 |  | UP |
| transmembrane protein 198(TMEM198) | ENSG00000188760 | TMEM198 | UP |
| pleckstrin homology and RhoGEF domain containing G4(PLEKHG4) | ENSG00000196155 | PLEKHG4 | UP |
| myosin light chain 6B(MYL6B) | ENSG00000196465 | MYL6B | UP |
| importin 4(IPO4) | ENSG00000196497 | IPO4 | UP |
| matrix metallopeptidase 1(MMP1) | ENSG00000196611 | MMP1 | UP |
| L antigen family member 3(LAGE3) | ENSG00000196976 | LAGE3 | UP |
| MAF bZIP transcription factor G(MAFG) | ENSG00000197063 | MAFG | UP |
| serpin family A member 1(SERPINA1) | ENSG00000197249 | SERPINA1 | UP |
| guanylate cyclase activator 2A(GUCA2A) | ENSG00000197273 | GUCA2A | UP |
| oxoglutarate dehydrogenase-like(OGDHL) | ENSG00000197444 | OGDHL | UP |
| stathmin 3(STMN3) | ENSG00000197457 | STMN3 | UP |
| homeobox C6(HOXC6) | ENSG00000197757 | HOXC6 | UP |
| glycosylphosphatidylinositol anchor attachment 1(GPAA1) | ENSG00000197858 | GPAA1 | UP |
| ADAMTS like 2(ADAMTSL2) | ENSG00000197859 | ADAMTSL2 | UP |
| TEA domain transcription factor 4(TEAD4) | ENSG00000197905 | TEAD4 | UP |
| ribosomal protein S6 kinase like 1(RPS6KL1) | ENSG00000198208 | RPS6KL1 | UP |
| C2 calcium dependent domain containing 4A(C2CD4A) | ENSG00000198535 | C2CD4A | UP |
| ankyrin repeat domain 13B(ANKRD13B) | ENSG00000198720 | ANKRD13B | UP |
| protein phosphatase 1 regulatory inhibitor subunit 14C(PPP1R14C) | ENSG00000198729 | PPP1R14C | UP |
| SPARC related modular calcium binding 1(SMOC1) | ENSG00000198732 | SMOC1 | UP |
| secretory carrier membrane protein 5(SCAMP5) | ENSG00000198794 | SCAMP5 | UP |
| protein regulator of cytokinesis 1(PRC1) | ENSG00000198901 | PRC1 | UP |
| small nucleolar RNA, H/ACA box 52(SNORA52) | ENSG00000199785 | SNORA52 | UP |
| small nucleolar RNA, C/D box 48(SNORD48) | ENSG00000201823 | SNORD48 | UP |
| small nucleolar RNA, C/D box 34(SNORD34) | ENSG00000202503 | SNORD34 | UP |
| glycoprotein Ib platelet beta subunit(GP1BB) | ENSG00000203618 | GP1BB | UP |
| chromosome 1 open reading frame 53(C1orf53) | ENSG00000203724 | C1orf53 | UP |
| SRY-box 18(SOX18) | ENSG00000203883 | SOX18 | UP |
| forkhead box O6(FOXO6) | ENSG00000204060 | FOXO6 | UP |
| Sp5 transcription factor(SP5) | ENSG00000204335 | SP5 | UP |
| adhesion G protein-coupled receptor G1(ADGRG1) | ENSG00000205336 | ADGRG1 | UP |
| C2 calcium dependent domain containing 4B(C2CD4B) | ENSG00000205502 | C2CD4B | UP |
|  | ENSG00000205622 |  | UP |
|  | ENSG00000205664 |  | UP |
| hematological and neurological expressed 1 like(HN1L) | ENSG00000206053 | HN1L | UP |
| small nucleolar RNA, H/ACA box 57(SNORA57) | ENSG00000206597 | SNORA57 | UP |
| small nucleolar RNA, H/ACA box 64(SNORA64) | ENSG00000207405 | SNORA64 | UP |
|  | ENSG00000210196 |  | DOWN |
|  | ENSG00000211892 |  | UP |
|  | ENSG00000211893 |  | UP |
|  | ENSG00000211896 |  | UP |
|  | ENSG00000211897 |  | UP |
| zinc finger protein 580(ZNF580) | ENSG00000213015 | ZNF580 | UP |
| chromosome 8 open reading frame 82(C8orf82) | ENSG00000213563 | C8orf82 | UP |
| StAR related lipid transfer domain containing 10(STARD10) | ENSG00000214530 | STARD10 | UP |
| TNF receptor superfamily member 25(TNFRSF25) | ENSG00000215788 | TNFRSF25 | UP |
| cytoskeleton regulator RNA(CYTOR) | ENSG00000222041 | CYTOR | UP |
| NADH:ubiquinone oxidoreductase complex assembly factor 8(NDUFAF8) | ENSG00000224877 | NDUFAF8 | UP |
|  | ENSG00000225071 |  | UP |
| MELTF antisense RNA 1(MELTF-AS1) | ENSG00000228109 | MELTF-AS1 | UP |
|  | ENSG00000229119 |  | UP |
| pepsinogen A4 (PGA4) | ENSG00000229183 | PGA4 | DOWN |
| prostate cancer susceptibility candidate 2(PRAC2) | ENSG00000229637 | PRAC2 | UP |
| pepsinogen A3 (PGA3) | ENSG00000229859 | PGA3 | DOWN |
| long intergenic non-protein coding RNA 1315(LINC01315) | ENSG00000229891 | LINC01315 | UP |
| HOXB cluster antisense RNA 1(HOXB-AS1) | ENSG00000230148 | HOXB-AS1 | UP |
| long intergenic non-protein coding RNA 1431(LINC01431) | ENSG00000232645 | LINC01431 | UP |
| transmembrane protein 238(TMEM238) | ENSG00000233493 | TMEM238 | UP |
|  | ENSG00000233966 |  | UP |
| uncharacterized LOC105378687(LOC105378687) | ENSG00000234694 | LOC105378687 | UP |
| HGH1 homolog(HGH1) | ENSG00000235173 | HGH1 | UP |
|  | ENSG00000235280 |  | UP |
| PITPNA antisense RNA 1(PITPNA-AS1) | ENSG00000236618 | PITPNA-AS1 | UP |
|  | ENSG00000237988 |  | UP |
| HNF1A antisense RNA 1(HNF1A-AS1) | ENSG00000241388 | HNF1A-AS1 | UP |
| alpha-methylacyl-CoA racemase(AMACR) | ENSG00000242110 | AMACR | UP |
| chromosome 4 open reading frame 48(C4orf48) | ENSG00000243449 | C4orf48 | UP |
| MNX1 antisense RNA 1 (head to head)(MNX1-AS1) | ENSG00000243479 | MNX1-AS1 | UP |
|  | ENSG00000244239 |  | UP |
| long intergenic non-protein coding RNA 1296(LINC01296) | ENSG00000244306 | LINC01296 | UP |
|  | ENSG00000245750 |  | UP |
| microRNA 1204(MIR1204) | ENSG00000249859 | MIR1204 | UP |
| transmembrane protein 158 (gene/pseudogene)(TMEM158) | ENSG00000249992 | TMEM158 | UP |
| HOXC cluster antisense RNA 1(HOXC-AS1) | ENSG00000250451 | HOXC-AS1 | UP |
|  | ENSG00000251357 |  | UP |
|  | ENSG00000253300 |  | UP |
| protein kinase, DNA-activated, catalytic polypeptide(PRKDC) | ENSG00000253729 | PRKDC | UP |
| leucine rich repeat containing 24(LRRC24) | ENSG00000254402 | LRRC24 | UP |
| AGAP2 antisense RNA 1(AGAP2-AS1) | ENSG00000255737 | AGAP2-AS1 | UP |
| PXN antisense RNA 1(PXN-AS1) | ENSG00000255857 | PXN-AS1 | UP |
| hydroxymethylbilane synthase(HMBS) | ENSG00000256269 | HMBS | UP |
| pepsinogen A5 (PGA5) | ENSG00000256713 | PGA5 | DOWN |
| cysteine rich protein 1(CRIP1) | ENSG00000257341 | CRIP1 | UP |
| InaF motif containing 1(INAFM1) | ENSG00000257704 | INAFM1 | UP |
| LY6/PLAUR domain containing 8(LYPD8) | ENSG00000259823 | LYPD8 | UP |
|  | ENSG00000260418 |  | UP |
| scleraxis bHLH transcription factor(SCX) | ENSG00000260428 | SCX | UP |
| microRNA 7112(MIR7112) | ENSG00000261236 | MIR7112 | UP |
| MAFG antisense RNA 1 (head to head)(MAFG-AS1) | ENSG00000265688 | MAFG-AS1 | UP |
| RAET1E antisense RNA 1(RAET1E-AS1) | ENSG00000268592 | RAET1E-AS1 | UP |
|  | ENSG00000269388 |  | UP |
|  | ENSG00000269968 |  | UP |
|  | ENSG00000271781 |  | UP |
|  | ENSG00000272599 |  | UP |
|  | ENSG00000272872 |  | UP |
|  | ENSG00000272933 |  | UP |
| family with sequence similarity 95 member C(FAM95C) | ENSG00000273036 | FAM95C | UP |
|  | ENSG00000273179 |  | UP |
| microRNA 6812(MIR6812) | ENSG00000273555 | MIR6812 | UP |
| coagulation factor VIII-associated 2(F8A2) | ENSG00000274791 | F8A2 | UP |
| serine/threonine-protein kinase SIK1(LOC102724428) | ENSG00000275993 | LOC102724428 | UP |
| ubiquitin like with PHD and ring finger domains 1(UHRF1) | ENSG00000276043 | UHRF1 | UP |
| ORAI calcium release-activated calcium modulator 1(ORAI1) | ENSG00000276045 | ORAI1 | UP |
| SRC kinase signaling inhibitor 1(SRCIN1) | ENSG00000277363 | SRCIN1 | UP |
|  | ENSG00000279140 |  | UP |
|  | ENSG00000279233 |  | UP |
|  | ENSG00000280229 |  | UP |
|  | ENSG00000280649 |  | UP |
|  | ENSG00000283526 |  | UP |

| **Table S2: Gene Ontology-Biological Process (GO-BP) Functional Enrichment Results using DAVID** | | | | | | | | | | | | |
| --- | --- | --- | --- | --- | --- | --- | --- | --- | --- | --- | --- | --- |
|  |  |  |  |  |  |  |  |  |  |  |  |  |
| Category | Term | Count | % | PValue | Genes | List Total | Pop Hits | Pop Total | Fold Enrichment | Bonferroni | Benjamini | FDR |
| GOTERM_BP_ALL | GO:0023061~signal release | 27 | 6.308411 | 6.38E-07 | EXOC3L1, SNAP25, LRP5, C2CD4A, CACNA1D, C2CD4B, C2CD4C, CPLX2, CPLX1, AACS, TRPM2, UCP2, FFAR2, PPFIA3, PTPRN2, UNC13A, KCNJ11, ABCC8, GAD1, CADPS, ADRA2C, NR0B2, SYT7, VGF, RFX6, STX1A, FOXA2 | 349 | 417 | 16792 | 3.115335 | 0.00295 | 0.002954 | 0.002902 |
| GOTERM_BP_ALL | GO:0044092~negative regulation of molecular function | 48 | 11.21495 | 1.80E-06 | SERPINA1, RTKN, PPP1R35, SERPINA10, SERPINE1, LRP5, PKMYT1, SHB, WFDC2, AURKA, CDC20, CST3, VTN, PCSK1N, CST1, SOCS1, DUSP10, PTTG1, HEY1, E2F1, TNFRSF4, SPINK4, PPP1R14B, PTPRN2, PPP1R14C, CDKN2A, UBE2C, TESC, SERPINF2, APOA2, SRCIN1, NR0B2, FOXP3, AGT, MAPK8IP1, PARP10, MAD2L2, HEYL, LOC102724428, FABP4, APOC1, RIMBP2, MAP3K10, DPEP1, SIK1, TRIB3, ANAPC5, FOXA2 | 349 | 1100 | 16792 | 2.099547 | 0.008312 | 0.004173 | 0.0041 |
| GOTERM_BP_ALL | GO:1903530~regulation of secretion by cell | 33 | 7.71028 | 2.93E-06 | SNAP25, LRP5, C2CD4A, CACNA1D, C2CD4B, C2CD4C, CPLX2, CPLX1, AACS, SCAMP5, TRPM2, SOCS1, RAB26, UCP2, FFAR2, TNFRSF4, CHRNB2, UNC13A, KCNJ11, ABCC8, APOA2, CCK, ADRA2C, SRCIN1, NR0B2, FOXP3, SYT7, AGT, RFX6, RAB15, STX1A, CDK5R2, FOXA2 | 349 | 630 | 16792 | 2.520289 | 0.013494 | 0.004529 | 0.004449 |
| GOTERM_BP_ALL | GO:0032940~secretion by cell | 43 | 10.04673 | 4.48E-06 | EXOC3L1, SNAP25, SERPINA1, SERPINE1, LRP5, C2CD4A, CACNA1D, C2CD4B, C2CD4C, CPLX2, CPLX1, AACS, SCAMP5, TRPM2, SOCS1, RAB26, UCP2, FFAR2, TNFRSF4, PPFIA3, CHRNB2, PTPRN2, UNC13A, KCNJ11, ABCC8, GAD1, SERPINF2, CADPS, APOA2, CCK, ADRA2C, SRCIN1, NR0B2, FOXP3, SYT7, AGT, VGF, RFX6, RAB15, CHI3L1, STX1A, CDK5R2, FOXA2 | 349 | 966 | 16792 | 2.141748 | 0.020529 | 0.004596 | 0.004515 |
| GOTERM_BP_ALL | GO:0002790~peptide secretion | 19 | 4.439252 | 4.97E-06 | EXOC3L1, SNAP25, PTPRN2, KCNJ11, ABCC8, LRP5, CACNA1D, ADRA2C, NR0B2, CPLX1, AACS, SYT7, TRPM2, VGF, RFX6, UCP2, FFAR2, STX1A, FOXA2 | 349 | 250 | 16792 | 3.656711 | 0.02273 | 0.004596 | 0.004515 |
| GOTERM_BP_ALL | GO:0044707~single-multicellular organism process | 164 | 38.31776 | 5.96E-06 | SPON2, SCARB1, CRTC1, HDAC11, PLEKHB1, SERPINE1, NUDT1, SCX, STMN3, MSI1, TPGS1, AQP3, SCAMP5, CDC20, RPS19, SOX18, SALL4, PHLDA2, TNFRSF4, MNX1, EPHB3, SLC39A3, CHRNB2, KCNH2, TPRA1, DGAT2, UNC13A, LIG1, SERPINF2, SOX12, MYO7A, MAPK8IP2, SRCIN1, PGC, FOXP3, PGF, TRAIP, LOC102724428, EID2, HOXB9, RFX6, TIMELESS, ORAI1, SIK1, HOXB2, ABCG1, REG3A, TPH1, BEX1, PRKDC, FOXO6, CACNA1D, WDR62, CPLX2, SEZ6L2, NME1-NME2, VTN, TRPM2, ADGRG1, PCSK1N, ADAMTS14, ZNHIT2, SOCS1, HOXC9, HOXC6, CBX2, SMAD9, ABCA7, NBEAL2, PGA3, NR0B2, PGA5, SYT7, PGA4, TMEM198, SDK1, ARTN, CDK4, MAFG, SP5, MESP1, SNAP25, SERPINA1, DAGLA, SERPINA10, CELF4, BICDL1, MYL6B, SHB, HOXC10, AACS, RND1, DUSP10, HEY1, ADAMTSL2, DACT2, FFAR2, DGCR6, TEAD4, TGIF1, EMX1, MYOC, MMP1, GP1BB, TESC, H2AFX, APOA2, KCNAB2, ADRA2C, ETV4, MTSS1, MMP11, VGF, VWA1, CHI3L1, TNFRSF25, PAFAH1B3, CEBPB, LRP5, ATP1A3, RRBP1, GDPD5, ASCL1, CRIP1, ASCL2, RELB, AURKA, DLL3, DLL4, RECQL4, CST3, PARD6A, MTHFD1L, RAB26, UCP2, E2F1, RAD54L, POLR2I, CTHRC1, HES4, CMTM8, LRRC24, NPM2, ZNF580, CDKN2A, IL34, TMEM176B, TMEM176A, STAC3, CCK, AGT, MAD2L2, HEYL, NFASC, FABP4, SMOC1, APOC1, MFAP2, NEURL1, TACC3, INSM1, KCNK3, CDK5R2, FOXA2 | 349 | 5943 | 16792 | 1.327746 | 0.027197 | 0.004596 | 0.004515 |
| GOTERM_BP_ALL | GO:0071705~nitrogen compound transport | 35 | 8.17757 | 8.08E-06 | EXOC3L1, SNAP25, NXT1, ZC3H3, LRP5, CACNA1D, SLC38A11, SLC1A5, SLC7A1, AQP3, CPLX1, AACS, TRPM2, TTR, UCP2, FFAR2, SLC25A22, SLC18A1, CHRNB2, PTPRN2, KCNJ11, NUP210, ABCC8, SLC52A2, CADPS, ABCA7, ADRA2C, NR0B2, SYT7, AGT, VGF, RFX6, SLC29A4, STX1A, FOXA2 | 349 | 725 | 16792 | 2.322774 | 0.036705 | 0.004898 | 0.004813 |
| GOTERM_BP_ALL | GO:1903047~mitotic cell cycle process | 40 | 9.345794 | 8.47E-06 | PRKDC, CDCA5, LRP5, HEPACAM2, DBF4B, WDR62, PKMYT1, CENPA, NCAPH, AURKA, CDC20, PTTG1, E2F1, MYBL2, TXNL4A, SAC3D1, GINS2, CDT1, PLK3, TPRA1, NPM2, LIG1, NUP210, CDKN2A, UBE2C, ARID3A, CDC25B, MAD2L2, KIF18B, CCNE1, UBE2S, PRC1, INCENP, CDK4, TIMELESS, TACC3, FAM64A, ANAPC5, KIF2C, MCM2 | 349 | 890 | 16792 | 2.162455 | 0.03843 | 0.004898 | 0.004813 |
| GOTERM_BP_ALL | GO:0042886~amide transport | 20 | 4.672897 | 1.01E-05 | EXOC3L1, SNAP25, PTPRN2, KCNJ11, ABCC8, LRP5, CACNA1D, ADRA2C, AQP3, NR0B2, CPLX1, AACS, SYT7, TRPM2, VGF, RFX6, UCP2, FFAR2, STX1A, FOXA2 | 349 | 289 | 16792 | 3.329731 | 0.045664 | 0.004982 | 0.004895 |
| GOTERM_BP_ALL | GO:0030072~peptide hormone secretion | 18 | 4.205607 | 1.19E-05 | EXOC3L1, SNAP25, PTPRN2, KCNJ11, ABCC8, LRP5, CACNA1D, ADRA2C, NR0B2, CPLX1, AACS, SYT7, TRPM2, VGF, RFX6, UCP2, STX1A, FOXA2 | 349 | 241 | 16792 | 3.593623 | 0.053741 | 0.004982 | 0.004895 |
| GOTERM_BP_ALL | GO:0007067~mitotic nuclear division | 25 | 5.841121 | 1.20E-05 | CDCA5, LRP5, HEPACAM2, PKMYT1, CENPA, NCAPH, AURKA, CDC20, PTTG1, MYBL2, TXNL4A, SAC3D1, NPM2, UBE2C, CDC25B, MAD2L2, KIF18B, UBE2S, PRC1, INCENP, TIMELESS, TACC3, FAM64A, ANAPC5, KIF2C | 349 | 431 | 16792 | 2.790871 | 0.054063 | 0.004982 | 0.004895 |
| GOTERM_BP_ALL | GO:0015833~peptide transport | 19 | 4.439252 | 1.29E-05 | EXOC3L1, SNAP25, PTPRN2, KCNJ11, ABCC8, LRP5, CACNA1D, ADRA2C, NR0B2, CPLX1, AACS, SYT7, TRPM2, VGF, RFX6, UCP2, FFAR2, STX1A, FOXA2 | 349 | 268 | 16792 | 3.411111 | 0.058037 | 0.004982 | 0.004895 |
| GOTERM_BP_ALL | GO:0044699~single-organism process | 308 | 71.96262 | 1.49E-05 | SPON2, ZNF296, CPNE7, HDAC11, PLEKHB1, SERPINE1, TONSL, NUDT1, SCX, STMN3, TPGS1, AQP3, SCAMP5, LIPF, CDC20, LIPE, RPS19, SOX18, MNX1, EPHB3, IER3, SDS, UNC13A, NUP210, SERPINF2, SOX12, MYO7A, BHLHA15, CPT1C, MAPK8IP2, MAPK8IP1, SULT2B1, EID2, HOXB9, CLDN3, RFX6, ASPHD1, HMBS, ORAI1, SIK1, HOXB2, FAM64A, TRIB3, PLPP2, STX1A, BEX2, TPH1, BEX1, TTYH3, TMEM63C, SHMT2, PRKDC, CDCA5, C2CD4A, C2CD4B, C2CD4C, WDR62, NCAPH, C2, TRPM2, ZNHIT2, ADAMTS14, HOXC9, HOXC6, CDT1, PLK3, PTPRN2, STARD10, ABCA7, NBEAL2, PGA3, NR0B2, PGA5, TMEM198, PGA4, PARP10, ARTN, PAH, FXYD2, PCSK1, MESP1, SERPINA1, GPAA1, MRPS12, SERPINA10, HEPACAM2, BICDL1, MYL6B, SHB, HOXC10, WFDC2, ASGR1, AACS, HEY1, ADAMTSL2, FFAR2, KIF1A, SLC18A1, TEAD4, MAP4K2, ATP6V0B, RPS6KL1, MYOC, GP1BB, TESC, SYP, AP3B2, MTSS1, MMP11, KIFC2, INCENP, SLC29A4, VWA1, KIF2C, PAFAH1B3, TIMM10, GDPD5, ASCL1, CENPA, CRIP1, SLC7A1, ASCL2, RELB, RECQL4, CST3, NPW, MTHFD1L, UCP2, RAD54L, PRAME, DTD1, GINS2, LRRC24, NPM2, ZNF580, CDKN2A, GDF15, IL34, GAD1, TMEM176B, TMEM176A, STAC3, AGT, GUCA2A, MAD2L2, HEYL, FABP4, SLCO3A1, SMOC1, SHKBP1, TACC3, CDK5R2, FOXA2, EXOC3L1, SCARB1, RTKN, CRTC1, GALNT18, REG1B, REG1A, MSI1, OGDHL, COX6A1, IPO4, TMEM145, PTTG1, SALL4, PHLDA2, TXNL4A, TNFRSF4, PPFIA3, SLC39A3, CHRNB2, KCNH2, TPRA1, DGAT2, KCNH6, LIG1, ATP11A, PGC, RUNDC3A, FOXP3, SRCIN1, PGF, TRAIP, CDC25B, LOC102724428, CCNE1, TIMELESS, DPEP1, ANAPC5, TSNARE1, ABCG1, LY6E, GNAZ, REG3A, SHC2, DPT, FOXO6, SLC38A11, CACNA1D, SLC1A5, CPLX2, SEZ6L2, PKMYT1, CPLX1, TIMM50, NME1-NME2, VTN, ADGRG1, PCSK1N, SOCS1, ADGRG5, RHPN1, BMF, CYC1, SAC3D1, UBE2C, SLC52A2, CBX2, ASPSCR1, CYBRD1, GGH, SMAD9, SYT7, SDK1, AMACR, G6PC3, KIF18B, UBE2S, CDK4, MAFG, MAP3K10, SP5, MELTF, FGFR4, ULBP2, RNF187, SNAP25, DAGLA, DYRK2, PYCRL, PODXL2, ZC3H3, CELF4, RND1, DUSP10, SPR, DACT2, MYBL2, DGCR6, CA8, GRASP, TGIF1, PLEKHG4, EMX1, ABCC8, MMP1, H2AFX, APOA2, C10ORF11, KCNAB2, ADRA2C, ETV4, VGF, CHI3L1, TNFRSF25, MCM2, CEBPB, NAXE, UHRF1, RGS16, LRP5, ATP1A3, RRBP1, DBF4B, CLN6, AURKA, DLL3, DLL4, P2RY6, TTR, PARD6A, PUF60, GNG4, RAB26, E2F1, POLR2I, SLC25A22, CTHRC1, HES4, OR51E1, CMTM8, KCNJ11, CADPS, ARID3A, CCK, FAM155B, NFASC, SLC25A39, PRC1, RAB15, TSPAN18, MFAP2, APOC1, NEURL1, INSM1, KCNK3 | 349 | 13377 | 16792 | 1.107819 | 0.066758 | 0.00507 | 0.004981 |
| GOTERM_BP_ALL | GO:0051046~regulation of secretion | 33 | 7.71028 | 1.53E-05 | SNAP25, LRP5, C2CD4A, CACNA1D, C2CD4B, C2CD4C, CPLX2, CPLX1, AACS, SCAMP5, TRPM2, SOCS1, RAB26, UCP2, FFAR2, TNFRSF4, CHRNB2, UNC13A, KCNJ11, ABCC8, APOA2, CCK, ADRA2C, SRCIN1, NR0B2, FOXP3, SYT7, AGT, RFX6, RAB15, STX1A, CDK5R2, FOXA2 | 349 | 683 | 16792 | 2.324718 | 0.068523 | 0.00507 | 0.004981 |
| GOTERM_BP_ALL | GO:0009306~protein secretion | 26 | 6.074766 | 1.67E-05 | SNAP25, LRP5, CACNA1D, CPLX1, AACS, SCAMP5, TRPM2, SOCS1, UCP2, FFAR2, TNFRSF4, PTPRN2, KCNJ11, ABCC8, APOA2, ADRA2C, SRCIN1, NR0B2, FOXP3, SYT7, AGT, VGF, RFX6, CHI3L1, STX1A, FOXA2 | 349 | 469 | 16792 | 2.667335 | 0.074262 | 0.005144 | 0.005054 |
| GOTERM_BP_ALL | GO:0046903~secretion | 45 | 10.51402 | 1.98E-05 | EXOC3L1, SNAP25, SERPINA1, SERPINE1, LRP5, C2CD4A, CACNA1D, C2CD4B, C2CD4C, CPLX2, CPLX1, AACS, SCAMP5, TRPM2, SOCS1, RAB26, UCP2, FFAR2, TNFRSF4, PPFIA3, CHRNB2, PTPRN2, UNC13A, KCNJ11, ABCC8, GAD1, SERPINF2, CADPS, STARD10, APOA2, CCK, ADRA2C, SRCIN1, NR0B2, FOXP3, SYT7, AGT, VGF, RFX6, RAB15, NEURL1, CHI3L1, STX1A, CDK5R2, FOXA2 | 349 | 1098 | 16792 | 1.97191 | 0.087701 | 0.0057 | 0.005601 |
| GOTERM_BP_ALL | GO:0017156~calcium ion regulated exocytosis | 12 | 2.803738 | 2.10E-05 | SCAMP5, SNAP25, UNC13A, CADPS, C2CD4A, C2CD4B, C2CD4C, CPLX2, CPLX1, STX1A, SYT7, CDK5R2 | 349 | 112 | 16792 | 5.155137 | 0.092582 | 0.0057 | 0.005601 |
| GOTERM_BP_ALL | GO:0009914~hormone transport | 20 | 4.672897 | 2.26E-05 | EXOC3L1, SNAP25, PTPRN2, KCNJ11, ABCC8, LRP5, CACNA1D, ADRA2C, NR0B2, CPLX1, AACS, SYT7, TRPM2, TTR, VGF, RFX6, UCP2, FFAR2, STX1A, FOXA2 | 349 | 306 | 16792 | 3.144746 | 0.099457 | 0.0057 | 0.005601 |
| GOTERM_BP_ALL | GO:0000278~mitotic cell cycle | 41 | 9.579439 | 2.44E-05 | PRKDC, CDCA5, LRP5, HEPACAM2, DBF4B, WDR62, PKMYT1, SHB, CENPA, NCAPH, AURKA, CDC20, PTTG1, E2F1, MYBL2, TXNL4A, SAC3D1, GINS2, CDT1, PLK3, TPRA1, NPM2, LIG1, NUP210, CDKN2A, UBE2C, ARID3A, CDC25B, MAD2L2, KIF18B, CCNE1, UBE2S, PRC1, INCENP, CDK4, TIMELESS, TACC3, FAM64A, ANAPC5, KIF2C, MCM2 | 349 | 968 | 16792 | 2.037912 | 0.106902 | 0.0057 | 0.005601 |
| GOTERM_BP_ALL | GO:0045595~regulation of cell differentiation | 57 | 13.31776 | 2.46E-05 | SNAP25, MESP1, CRTC1, PLEKHB1, SHB, AQP3, CDC20, DUSP10, HEY1, RPS19, EPHB3, CHRNB2, EMX1, UNC13A, MYOC, TESC, SERPINF2, BHLHA15, ADRA2C, SRCIN1, FOXP3, LOC102724428, MMP11, TRIB3, SIK1, ABCG1, REG3A, CEBPB, TPH1, LRP5, FOXO6, GDPD5, ASCL1, ASCL2, AURKA, DLL3, NME1-NME2, DLL4, ADGRG1, E2F1, PRAME, CTHRC1, CDKN2A, GDF15, IL34, TMEM176B, TMEM176A, AGT, MAD2L2, HEYL, SDK1, MAFG, SMOC1, NEURL1, MELTF, INSM1, FOXA2 | 349 | 1544 | 16792 | 1.776252 | 0.107752 | 0.0057 | 0.005601 |
| GOTERM_BP_ALL | GO:0051301~cell division | 29 | 6.775701 | 2.68E-05 | CDCA5, HEPACAM2, CENPA, NCAPH, AURKA, CDC20, PARD6A, PTTG1, TXNL4A, SAC3D1, PLK3, TPRA1, LIG1, CDKN2A, UBE2C, PGF, CDC25B, MAD2L2, KIF18B, CCNE1, UBE2S, PRC1, INCENP, CDK4, TIMELESS, TACC3, FAM64A, ANAPC5, KIF2C | 349 | 575 | 16792 | 2.42665 | 0.116829 | 0.005916 | 0.005812 |
| GOTERM_BP_ALL | GO:0043086~negative regulation of catalytic activity | 37 | 8.64486 | 2.94E-05 | SERPINA1, RTKN, PPP1R35, SERPINA10, SERPINE1, LRP5, PKMYT1, SHB, WFDC2, CDC20, CST3, VTN, PCSK1N, CST1, SOCS1, DUSP10, PTTG1, SPINK4, PPP1R14B, PTPRN2, PPP1R14C, CDKN2A, UBE2C, TESC, SERPINF2, APOA2, SRCIN1, AGT, MAPK8IP1, MAD2L2, FABP4, APOC1, RIMBP2, DPEP1, TRIB3, ANAPC5, FOXA2 | 349 | 839 | 16792 | 2.12186 | 0.127082 | 0.006067 | 0.005961 |
| GOTERM_BP_ALL | GO:0007275~multicellular organism development | 137 | 32.00935 | 3.12E-05 | SPON2, CRTC1, HDAC11, PLEKHB1, SERPINE1, NUDT1, SCX, STMN3, MSI1, TPGS1, AQP3, CDC20, RPS19, SOX18, SALL4, PHLDA2, TNFRSF4, MNX1, EPHB3, SLC39A3, CHRNB2, TPRA1, DGAT2, UNC13A, LIG1, SERPINF2, SOX12, MYO7A, MAPK8IP2, SRCIN1, FOXP3, PGF, LOC102724428, EID2, HOXB9, RFX6, TIMELESS, ORAI1, SIK1, HOXB2, REG3A, TPH1, BEX1, PRKDC, FOXO6, WDR62, CPLX2, SEZ6L2, NME1-NME2, VTN, TRPM2, ADGRG1, ZNHIT2, HOXC9, HOXC6, CBX2, SMAD9, NBEAL2, NR0B2, TMEM198, SDK1, ARTN, CDK4, MAFG, SP5, MESP1, SNAP25, DAGLA, CELF4, BICDL1, MYL6B, SHB, HOXC10, AACS, RND1, DUSP10, HEY1, ADAMTSL2, DACT2, DGCR6, TEAD4, TGIF1, EMX1, MYOC, TESC, H2AFX, APOA2, KCNAB2, ADRA2C, ETV4, MTSS1, MMP11, VGF, CHI3L1, TNFRSF25, PAFAH1B3, CEBPB, LRP5, GDPD5, ASCL1, CRIP1, ASCL2, RELB, AURKA, DLL3, DLL4, RECQL4, CST3, PARD6A, MTHFD1L, RAB26, UCP2, E2F1, RAD54L, CTHRC1, HES4, CMTM8, LRRC24, NPM2, CDKN2A, IL34, TMEM176B, TMEM176A, STAC3, CCK, AGT, MAD2L2, HEYL, NFASC, SMOC1, MFAP2, NEURL1, TACC3, INSM1, KCNK3, CDK5R2, FOXA2 | 349 | 4877 | 16792 | 1.35159 | 0.134354 | 0.006067 | 0.005961 |
| GOTERM_BP_ALL | GO:0007269~neurotransmitter secretion | 13 | 3.037383 | 3.28E-05 | SNAP25, PTPRN2, UNC13A, GAD1, CADPS, C2CD4A, C2CD4B, C2CD4C, CPLX2, CPLX1, SYT7, STX1A, PPFIA3 | 349 | 139 | 16792 | 4.499928 | 0.14073 | 0.006067 | 0.005961 |
| GOTERM_BP_ALL | GO:0099643~signal release from synapse | 13 | 3.037383 | 3.28E-05 | SNAP25, PTPRN2, UNC13A, GAD1, CADPS, C2CD4A, C2CD4B, C2CD4C, CPLX2, CPLX1, SYT7, STX1A, PPFIA3 | 349 | 139 | 16792 | 4.499928 | 0.14073 | 0.006067 | 0.005961 |
| GOTERM_BP_ALL | GO:0048731~system development | 123 | 28.73832 | 4.40E-05 | SPON2, CRTC1, HDAC11, SERPINE1, NUDT1, SCX, STMN3, MSI1, AQP3, CDC20, RPS19, SOX18, SALL4, PHLDA2, MNX1, EPHB3, SLC39A3, CHRNB2, DGAT2, UNC13A, LIG1, SERPINF2, SOX12, MYO7A, MAPK8IP2, SRCIN1, FOXP3, PGF, LOC102724428, EID2, HOXB9, RFX6, TIMELESS, ORAI1, SIK1, HOXB2, REG3A, TPH1, BEX1, PRKDC, FOXO6, WDR62, CPLX2, SEZ6L2, NME1-NME2, VTN, TRPM2, ADGRG1, ZNHIT2, HOXC9, HOXC6, SMAD9, NBEAL2, NR0B2, SDK1, ARTN, CDK4, SP5, MESP1, SNAP25, DAGLA, BICDL1, MYL6B, SHB, HOXC10, AACS, RND1, DUSP10, HEY1, ADAMTSL2, DACT2, DGCR6, TEAD4, EMX1, MYOC, TESC, H2AFX, APOA2, KCNAB2, ADRA2C, ETV4, MTSS1, VGF, CHI3L1, PAFAH1B3, CEBPB, LRP5, GDPD5, ASCL1, CRIP1, ASCL2, RELB, AURKA, DLL3, DLL4, CST3, PARD6A, MTHFD1L, RAB26, UCP2, E2F1, CTHRC1, HES4, CMTM8, LRRC24, CDKN2A, IL34, TMEM176B, TMEM176A, STAC3, CCK, AGT, MAD2L2, HEYL, NFASC, SMOC1, MFAP2, NEURL1, TACC3, INSM1, KCNK3, CDK5R2, FOXA2 | 349 | 4291 | 16792 | 1.379188 | 0.184231 | 0.007832 | 0.007694 |
| GOTERM_BP_ALL | GO:0046879~hormone secretion | 19 | 4.439252 | 4.63E-05 | EXOC3L1, SNAP25, PTPRN2, KCNJ11, ABCC8, LRP5, CACNA1D, ADRA2C, NR0B2, CPLX1, AACS, SYT7, TRPM2, VGF, RFX6, UCP2, FFAR2, STX1A, FOXA2 | 349 | 295 | 16792 | 3.098907 | 0.193012 | 0.007942 | 0.007803 |
| GOTERM_BP_ALL | GO:0099531~presynaptic process involved in chemical synaptic transmission | 13 | 3.037383 | 4.97E-05 | SNAP25, PTPRN2, UNC13A, GAD1, CADPS, C2CD4A, C2CD4B, C2CD4C, CPLX2, CPLX1, SYT7, STX1A, PPFIA3 | 349 | 145 | 16792 | 4.313724 | 0.205603 | 0.00822 | 0.008076 |
| GOTERM_BP_ALL | GO:0010817~regulation of hormone levels | 25 | 5.841121 | 5.75E-05 | PCSK1, EXOC3L1, SNAP25, SCARB1, LRP5, CACNA1D, CPLX1, AACS, TRPM2, PCSK1N, TTR, UCP2, FFAR2, PTPRN2, DGAT2, KCNJ11, ABCC8, ADRA2C, NR0B2, SYT7, AGT, VGF, RFX6, STX1A, FOXA2 | 349 | 475 | 16792 | 2.532348 | 0.233605 | 0.009174 | 0.009014 |
| GOTERM_BP_ALL | GO:0001505~regulation of neurotransmitter levels | 15 | 3.504673 | 6.41E-05 | SNAP25, PTPRN2, DAGLA, UNC13A, GAD1, CADPS, C2CD4A, C2CD4B, C2CD4C, CPLX2, CPLX1, SYT7, PAH, STX1A, PPFIA3 | 349 | 197 | 16792 | 3.663549 | 0.256921 | 0.009898 | 0.009725 |
| GOTERM_BP_ALL | GO:0016079~synaptic vesicle exocytosis | 10 | 2.336449 | 7.19E-05 | SNAP25, UNC13A, CADPS, C2CD4A, C2CD4B, C2CD4C, CPLX2, CPLX1, STX1A, SYT7 | 349 | 86 | 16792 | 5.594722 | 0.282995 | 0.010731 | 0.010543 |
| GOTERM_BP_ALL | GO:0050796~regulation of insulin secretion | 14 | 3.271028 | 7.48E-05 | SNAP25, KCNJ11, ABCC8, LRP5, CACNA1D, ADRA2C, NR0B2, AACS, SYT7, TRPM2, RFX6, UCP2, STX1A, FOXA2 | 349 | 175 | 16792 | 3.849169 | 0.292674 | 0.01082 | 0.010631 |
| GOTERM_BP_ALL | GO:0090276~regulation of peptide hormone secretion | 15 | 3.504673 | 8.45E-05 | SNAP25, KCNJ11, ABCC8, LRP5, CACNA1D, ADRA2C, NR0B2, AACS, SYT7, TRPM2, RFX6, UCP2, FFAR2, STX1A, FOXA2 | 349 | 202 | 16792 | 3.572867 | 0.323878 | 0.01186 | 0.011652 |
| GOTERM_BP_ALL | GO:0002791~regulation of peptide secretion | 15 | 3.504673 | 1.10E-04 | SNAP25, KCNJ11, ABCC8, LRP5, CACNA1D, ADRA2C, NR0B2, AACS, SYT7, TRPM2, RFX6, UCP2, FFAR2, STX1A, FOXA2 | 349 | 207 | 16792 | 3.486566 | 0.398323 | 0.014941 | 0.01468 |
| GOTERM_BP_ALL | GO:0048513~animal organ development | 94 | 21.96262 | 1.13E-04 | MESP1, NUDT1, SCX, MYL6B, SHB, AQP3, HOXC10, AACS, HEY1, RPS19, SOX18, SALL4, ADAMTSL2, DACT2, DGCR6, PHLDA2, MNX1, EPHB3, SLC39A3, TEAD4, CHRNB2, EMX1, DGAT2, MYOC, TESC, H2AFX, APOA2, SOX12, MYO7A, KCNAB2, ETV4, FOXP3, MTSS1, PGF, LOC102724428, EID2, HOXB9, VGF, RFX6, TIMELESS, ORAI1, CHI3L1, HOXB2, SIK1, PAFAH1B3, REG3A, CEBPB, TPH1, PRKDC, LRP5, WDR62, GDPD5, ASCL1, SEZ6L2, ASCL2, CRIP1, AURKA, DLL3, RELB, NME1-NME2, DLL4, CST3, VTN, TRPM2, ADGRG1, PARD6A, MTHFD1L, UCP2, E2F1, HOXC9, CTHRC1, CDKN2A, IL34, TMEM176B, TMEM176A, STAC3, SMAD9, NBEAL2, NR0B2, AGT, MAD2L2, HEYL, SDK1, ARTN, CDK4, SMOC1, MFAP2, NEURL1, TACC3, SP5, INSM1, KCNK3, CDK5R2, FOXA2 | 349 | 3129 | 16792 | 1.445437 | 0.408295 | 0.014992 | 0.01473 |
| GOTERM_BP_ALL | GO:0090087~regulation of peptide transport | 15 | 3.504673 | 1.22E-04 | SNAP25, KCNJ11, ABCC8, LRP5, CACNA1D, ADRA2C, NR0B2, AACS, SYT7, TRPM2, RFX6, UCP2, FFAR2, STX1A, FOXA2 | 349 | 209 | 16792 | 3.453202 | 0.430284 | 0.015627 | 0.015354 |
| GOTERM_BP_ALL | GO:0099504~synaptic vesicle cycle | 11 | 2.570093 | 1.39E-04 | SNAP25, UNC13A, CADPS, C2CD4A, C2CD4B, C2CD4C, CPLX2, SLC18A1, CPLX1, STX1A, SYT7 | 349 | 115 | 16792 | 4.602267 | 0.475434 | 0.017436 | 0.017131 |
| GOTERM_BP_ALL | GO:0030154~cell differentiation | 105 | 24.53271 | 2.08E-04 | SPON2, CRTC1, HDAC11, SCX, STMN3, TPGS1, AQP3, CDC20, RPS19, SOX18, MNX1, EPHB3, CHRNB2, UNC13A, SERPINF2, SOX12, MYO7A, BHLHA15, MAPK8IP2, SRCIN1, FOXP3, PGF, CDC25B, LOC102724428, EID2, CLDN3, RFX6, SIK1, TRIB3, ABCG1, REG3A, TPH1, BEX1, PRKDC, FOXO6, WDR62, CPLX2, NME1-NME2, VTN, TRPM2, ADGRG1, ZNHIT2, SOCS1, CBX2, NBEAL2, SDK1, ARTN, MAFG, MELTF, MESP1, SNAP25, DAGLA, CELF4, BICDL1, SHB, HOXC10, AACS, RND1, DUSP10, HEY1, DACT2, FFAR2, MYBL2, EMX1, MYOC, TESC, C10ORF11, KCNAB2, ADRA2C, ETV4, MTSS1, MMP11, CEBPB, LRP5, RRBP1, GDPD5, ASCL1, ASCL2, RELB, AURKA, DLL3, DLL4, CST3, E2F1, PRAME, CTHRC1, HES4, NPM2, CDKN2A, GDF15, IL34, TMEM176B, TMEM176A, STAC3, CCK, AGT, MAD2L2, HEYL, NFASC, FABP4, SMOC1, NEURL1, INSM1, CDK5R2, FOXA2 | 349 | 3652 | 16792 | 1.383361 | 0.619069 | 0.024809 | 0.024375 |
| GOTERM_BP_ALL | GO:0050708~regulation of protein secretion | 21 | 4.906542 | 2.09E-04 | SNAP25, KCNJ11, ABCC8, LRP5, APOA2, CACNA1D, ADRA2C, NR0B2, SRCIN1, FOXP3, AACS, SYT7, SCAMP5, TRPM2, SOCS1, RFX6, UCP2, FFAR2, TNFRSF4, STX1A, FOXA2 | 349 | 392 | 16792 | 2.577569 | 0.620022 | 0.024809 | 0.024375 |
| GOTERM_BP_ALL | GO:0051049~regulation of transport | 60 | 14.01869 | 2.31E-04 | SCARB1, SNAP25, DYRK2, ZC3H3, SERPINE1, TONSL, AACS, SCAMP5, FFAR2, TNFRSF4, KCNH2, CHRNB2, UNC13A, KCNH6, NUP210, ABCC8, TESC, APOA2, KCNAB2, ADRA2C, MAPK8IP2, SRCIN1, FOXP3, LOC102724428, RFX6, ORAI1, TRIB3, SIK1, ABCG1, STX1A, LRP5, C2CD4A, CACNA1D, C2CD4B, C2CD4C, CPLX2, CPLX1, C2, VTN, TRPM2, SOCS1, RAB26, UCP2, E2F1, BMF, PLK3, KCNJ11, CDKN2A, ASPSCR1, ABCA7, CCK, NR0B2, AGT, SYT7, PARP10, RAB15, FXYD2, APOC1, CDK5R2, FOXA2 | 349 | 1796 | 16792 | 1.607392 | 0.657002 | 0.026748 | 0.02628 |
| GOTERM_BP_ALL | GO:0006836~neurotransmitter transport | 14 | 3.271028 | 2.43E-04 | SNAP25, PTPRN2, UNC13A, GAD1, CADPS, C2CD4A, C2CD4B, C2CD4C, CPLX2, CPLX1, SYT7, SLC18A1, STX1A, PPFIA3 | 349 | 197 | 16792 | 3.419313 | 0.675136 | 0.02742 | 0.02694 |
| GOTERM_BP_ALL | GO:0099003~vesicle mediated transport in synapse | 10 | 2.336449 | 2.68E-04 | SNAP25, UNC13A, CADPS, C2CD4A, C2CD4B, C2CD4C, CPLX2, CPLX1, STX1A, SYT7 | 349 | 102 | 16792 | 4.717119 | 0.710758 | 0.029532 | 0.029015 |
| GOTERM_BP_ALL | GO:0030073~insulin secretion | 12 | 2.803738 | 3.14E-04 | TRPM2, PTPRN2, VGF, RFX6, KCNJ11, ABCC8, UCP2, LRP5, NR0B2, CPLX1, AACS, FOXA2 | 349 | 151 | 16792 | 3.823678 | 0.766136 | 0.032324 | 0.031758 |
| GOTERM_BP_ALL | GO:0048489~synaptic vesicle transport | 11 | 2.570093 | 3.14E-04 | SNAP25, UNC13A, CADPS, C2CD4A, C2CD4B, C2CD4C, CPLX2, AP3B2, CPLX1, STX1A, SYT7 | 349 | 127 | 16792 | 4.167407 | 0.766555 | 0.032324 | 0.031758 |
| GOTERM_BP_ALL | GO:0097480~establishment of synaptic vesicle localization | 11 | 2.570093 | 3.14E-04 | SNAP25, UNC13A, CADPS, C2CD4A, C2CD4B, C2CD4C, CPLX2, AP3B2, CPLX1, STX1A, SYT7 | 349 | 127 | 16792 | 4.167407 | 0.766555 | 0.032324 | 0.031758 |
| GOTERM_BP_ALL | GO:1902578~single-organism localization | 103 | 24.06542 | 3.47E-04 | SPON2, SCARB1, EXOC3L1, SERPINE1, TONSL, COX6A1, AQP3, IPO4, SCAMP5, RPS19, TNFRSF4, PPFIA3, SLC39A3, CHRNB2, KCNH2, UNC13A, KCNH6, NUP210, SERPINF2, MYO7A, BHLHA15, ATP11A, MAPK8IP2, SRCIN1, FOXP3, LOC102724428, RFX6, ORAI1, SIK1, ABCG1, STX1A, TTYH3, TMEM63C, C2CD4A, CACNA1D, C2CD4B, SLC38A11, C2CD4C, SLC1A5, CPLX2, CPLX1, TIMM50, C2, TRPM2, SOCS1, BMF, CYC1, PTPRN2, SLC52A2, STARD10, ASPSCR1, ABCA7, NR0B2, SYT7, PARP10, G6PC3, FXYD2, MELTF, SNAP25, SERPINA1, DYRK2, GPAA1, AACS, FFAR2, KIF1A, SLC18A1, ATP6V0B, ABCC8, TESC, APOA2, KCNAB2, ADRA2C, AP3B2, VGF, CHI3L1, SLC29A4, LRP5, ATP1A3, TIMM10, CENPA, SLC7A1, P2RY6, TTR, RAB26, UCP2, E2F1, SLC25A22, CMTM8, KCNJ11, GAD1, CADPS, CCK, AGT, FAM155B, NFASC, SLC25A39, SLCO3A1, RAB15, APOC1, NEURL1, KCNK3, CDK5R2, FOXA2 | 349 | 3614 | 16792 | 1.37128 | 0.799789 | 0.034405 | 0.033803 |
| GOTERM_BP_ALL | GO:0045055~regulated exocytosis | 17 | 3.971963 | 3.49E-04 | SNAP25, SERPINA1, UNC13A, SERPINF2, CADPS, SERPINE1, C2CD4A, C2CD4B, C2CD4C, CPLX2, CPLX1, SYT7, SCAMP5, RAB26, RAB15, CDK5R2, STX1A | 349 | 288 | 16792 | 2.840099 | 0.801566 | 0.034405 | 0.033803 |
| GOTERM_BP_ALL | GO:0007267~cell-cell signaling | 52 | 12.14953 | 3.80E-04 | PCSK1, EXOC3L1, SNAP25, MESP1, CRTC1, CELF4, TPGS1, AACS, CDC20, FFAR2, SLC18A1, PPFIA3, CHRNB2, UNC13A, MYOC, ABCC8, BHLHA15, SYP, ADRA2C, MAPK8IP2, PGF, HOXB9, VGF, RFX6, CCNE1, STX1A, LRP5, C2CD4A, CACNA1D, C2CD4B, C2CD4C, CPLX2, CPLX1, TRPM2, ADGRG1, PARD6A, UCP2, CTHRC1, PTPRN2, KCNJ11, GDF15, GAD1, CADPS, STAC3, NR0B2, AGT, SYT7, TMEM198, MAD2L2, NEURL1, KCNK3, FOXA2 | 349 | 1517 | 16792 | 1.649281 | 0.828205 | 0.03669 | 0.036048 |
| GOTERM_BP_ALL | GO:0044765~single-organism transport | 98 | 22.8972 | 4.14E-04 | SPON2, SCARB1, EXOC3L1, SERPINE1, TONSL, COX6A1, AQP3, IPO4, SCAMP5, RPS19, TNFRSF4, PPFIA3, SLC39A3, CHRNB2, KCNH2, UNC13A, KCNH6, NUP210, SERPINF2, MYO7A, BHLHA15, ATP11A, MAPK8IP2, SRCIN1, FOXP3, LOC102724428, RFX6, ORAI1, SIK1, ABCG1, STX1A, TTYH3, TMEM63C, C2CD4A, CACNA1D, C2CD4B, SLC38A11, C2CD4C, SLC1A5, CPLX2, CPLX1, TIMM50, C2, TRPM2, SOCS1, CYC1, PTPRN2, SLC52A2, STARD10, ASPSCR1, ABCA7, NR0B2, SYT7, PARP10, G6PC3, FXYD2, MELTF, SNAP25, SERPINA1, DYRK2, AACS, FFAR2, KIF1A, SLC18A1, ATP6V0B, ABCC8, TESC, APOA2, KCNAB2, ADRA2C, AP3B2, VGF, CHI3L1, SLC29A4, LRP5, ATP1A3, TIMM10, SLC7A1, P2RY6, TTR, RAB26, UCP2, SLC25A22, KCNJ11, GAD1, CADPS, CCK, AGT, FAM155B, NFASC, SLC25A39, SLCO3A1, RAB15, APOC1, NEURL1, KCNK3, CDK5R2, FOXA2 | 349 | 3415 | 16792 | 1.380741 | 0.8526 | 0.039066 | 0.038382 |
| GOTERM_BP_ALL | GO:0021700~developmental maturation | 15 | 3.504673 | 4.25E-04 | UNC13A, MYOC, SYP, BHLHA15, SEZ6L2, ASCL1, SHB, CDC25B, AURKA, RND1, CDC20, NFASC, SOX18, NEURL1, CDK5R2 | 349 | 236 | 16792 | 3.058132 | 0.860265 | 0.039352 | 0.038663 |
| GOTERM_BP_ALL | GO:0097479~synaptic vesicle localization | 11 | 2.570093 | 4.82E-04 | SNAP25, UNC13A, CADPS, C2CD4A, C2CD4B, C2CD4C, CPLX2, AP3B2, CPLX1, STX1A, SYT7 | 349 | 134 | 16792 | 3.949707 | 0.892865 | 0.043041 | 0.042288 |
| GOTERM_BP_ALL | GO:0044767~single-organism developmental process | 144 | 33.64486 | 4.84E-04 | SPON2, CRTC1, HDAC11, PLEKHB1, SERPINE1, NUDT1, SCX, STMN3, MSI1, TPGS1, AQP3, CDC20, RPS19, SOX18, SALL4, PHLDA2, TNFRSF4, MNX1, EPHB3, SLC39A3, CHRNB2, TPRA1, DGAT2, UNC13A, LIG1, SERPINF2, SOX12, MYO7A, BHLHA15, MAPK8IP2, SRCIN1, FOXP3, PGF, CDC25B, LOC102724428, EID2, CLDN3, HOXB9, RFX6, TIMELESS, ORAI1, SIK1, HOXB2, REG3A, TPH1, BEX1, PRKDC, FOXO6, WDR62, CPLX2, SEZ6L2, NME1-NME2, VTN, TRPM2, ADGRG1, ZNHIT2, HOXC9, HOXC6, CBX2, SMAD9, NBEAL2, NR0B2, TMEM198, SDK1, ARTN, CDK4, MAFG, SP5, MELTF, MESP1, SNAP25, DAGLA, CELF4, BICDL1, MYL6B, SHB, HOXC10, AACS, RND1, DUSP10, HEY1, ADAMTSL2, DACT2, DGCR6, TEAD4, TGIF1, EMX1, MYOC, TESC, H2AFX, APOA2, KCNAB2, SYP, ADRA2C, ETV4, MTSS1, MMP11, VGF, CHI3L1, TNFRSF25, PAFAH1B3, CEBPB, LRP5, GDPD5, ASCL1, CRIP1, ASCL2, RELB, AURKA, DLL3, DLL4, RECQL4, CST3, PARD6A, MTHFD1L, RAB26, UCP2, E2F1, RAD54L, POLR2I, CTHRC1, HES4, CMTM8, LRRC24, NPM2, CDKN2A, GDF15, IL34, TMEM176B, TMEM176A, STAC3, CCK, AGT, MAD2L2, HEYL, NFASC, SMOC1, MFAP2, NEURL1, TACC3, INSM1, KCNK3, CDK5R2, FOXA2 | 349 | 5475 | 16792 | 1.26548 | 0.893399 | 0.043041 | 0.042288 |
| GOTERM_BP_ALL | GO:0000280~nuclear division | 26 | 6.074766 | 5.07E-04 | CDCA5, LRP5, HEPACAM2, PKMYT1, CENPA, NCAPH, AURKA, CDC20, PTTG1, RAD54L, MYBL2, TXNL4A, SAC3D1, NPM2, UBE2C, CDC25B, MAD2L2, KIF18B, UBE2S, PRC1, INCENP, TIMELESS, TACC3, FAM64A, ANAPC5, KIF2C | 349 | 583 | 16792 | 2.145763 | 0.904462 | 0.044295 | 0.04352 |
| GOTERM_BP_ALL | GO:0043433~negative regulation of sequence-specific DNA binding transcription factor activity | 11 | 2.570093 | 5.74E-04 | PARP10, MAD2L2, LOC102724428, HEYL, CDKN2A, MAP3K10, SIK1, TNFRSF4, NR0B2, FOXP3, FOXA2 | 349 | 137 | 16792 | 3.863217 | 0.930048 | 0.049244 | 0.048382 |
| GOTERM_BP_ALL | GO:0007346~regulation of mitotic cell cycle | 23 | 5.373832 | 6.15E-04 | PLK3, TPRA1, NPM2, CDKN2A, PRKDC, UBE2C, CDCA5, LRP5, ARID3A, DBF4B, PKMYT1, ASCL1, SHB, CDC25B, AURKA, MAD2L2, CDC20, LOC102724428, CDK4, E2F1, TACC3, ANAPC5, SIK1 | 349 | 491 | 16792 | 2.253841 | 0.941894 | 0.05172 | 0.050815 |
| GOTERM_BP_ALL | GO:0071827~plasma lipoprotein particle organization | 6 | 1.401869 | 6.30E-04 | SCARB1, APOC1, APOA2, ABCA7, ABCG1, AGT | 349 | 34 | 16792 | 8.490814 | 0.946033 | 0.052115 | 0.051203 |
| GOTERM_BP_ALL | GO:0044763~single-organism cellular process | 281 | 65.65421 | 6.79E-04 | SPON2, CPNE7, HDAC11, PLEKHB1, SERPINE1, TONSL, NUDT1, SCX, STMN3, TPGS1, AQP3, SCAMP5, LIPF, CDC20, LIPE, RPS19, SOX18, MNX1, EPHB3, IER3, SDS, UNC13A, NUP210, SERPINF2, SOX12, MYO7A, BHLHA15, CPT1C, MAPK8IP2, MAPK8IP1, SULT2B1, EID2, HOXB9, CLDN3, RFX6, HMBS, ORAI1, SIK1, FAM64A, TRIB3, PLPP2, STX1A, BEX2, TPH1, BEX1, TTYH3, TMEM63C, SHMT2, PRKDC, CDCA5, C2CD4A, C2CD4B, C2CD4C, WDR62, NCAPH, TRPM2, ZNHIT2, ADAMTS14, CDT1, PLK3, PTPRN2, STARD10, ABCA7, NBEAL2, NR0B2, TMEM198, PARP10, ARTN, PAH, FXYD2, PCSK1, MESP1, SERPINA1, GPAA1, MRPS12, HEPACAM2, BICDL1, MYL6B, SHB, HOXC10, ASGR1, AACS, HEY1, ADAMTSL2, FFAR2, KIF1A, SLC18A1, TEAD4, MAP4K2, ATP6V0B, RPS6KL1, MYOC, GP1BB, TESC, SYP, AP3B2, MTSS1, MMP11, KIFC2, INCENP, SLC29A4, VWA1, KIF2C, TIMM10, GDPD5, ASCL1, CENPA, CRIP1, SLC7A1, ASCL2, RELB, CST3, NPW, MTHFD1L, UCP2, RAD54L, PRAME, DTD1, GINS2, LRRC24, NPM2, ZNF580, CDKN2A, GDF15, IL34, GAD1, TMEM176B, TMEM176A, STAC3, AGT, GUCA2A, MAD2L2, HEYL, FABP4, SMOC1, SHKBP1, TACC3, CDK5R2, FOXA2, EXOC3L1, SCARB1, RTKN, CRTC1, GALNT18, OGDHL, COX6A1, TMEM145, PTTG1, SALL4, PHLDA2, TXNL4A, TNFRSF4, PPFIA3, SLC39A3, CHRNB2, KCNH2, TPRA1, DGAT2, KCNH6, LIG1, ATP11A, RUNDC3A, FOXP3, SRCIN1, PGF, TRAIP, CDC25B, LOC102724428, CCNE1, TIMELESS, DPEP1, ANAPC5, TSNARE1, ABCG1, LY6E, GNAZ, REG3A, SHC2, DPT, FOXO6, SLC38A11, CACNA1D, SLC1A5, CPLX2, SEZ6L2, PKMYT1, CPLX1, TIMM50, NME1-NME2, VTN, ADGRG1, PCSK1N, SOCS1, ADGRG5, RHPN1, BMF, CYC1, SAC3D1, UBE2C, CBX2, CYBRD1, GGH, SMAD9, SYT7, SDK1, AMACR, KIF18B, UBE2S, CDK4, MAFG, MAP3K10, MELTF, FGFR4, ULBP2, SNAP25, DAGLA, DYRK2, PYCRL, PODXL2, ZC3H3, CELF4, RND1, DUSP10, DACT2, MYBL2, CA8, GRASP, PLEKHG4, EMX1, ABCC8, MMP1, H2AFX, APOA2, C10ORF11, KCNAB2, ADRA2C, ETV4, VGF, CHI3L1, TNFRSF25, MCM2, CEBPB, NAXE, UHRF1, RGS16, LRP5, ATP1A3, RRBP1, DBF4B, CLN6, AURKA, DLL3, DLL4, P2RY6, TTR, PARD6A, PUF60, GNG4, RAB26, E2F1, POLR2I, SLC25A22, CTHRC1, HES4, OR51E1, CMTM8, KCNJ11, CADPS, ARID3A, CCK, FAM155B, NFASC, SLC25A39, PRC1, RAB15, TSPAN18, MFAP2, APOC1, NEURL1, INSM1, KCNK3 | 349 | 12256 | 16792 | 1.10315 | 0.956853 | 0.054317 | 0.053367 |
| GOTERM_BP_ALL | GO:0048167~regulation of synaptic plasticity | 11 | 2.570093 | 6.81E-04 | CDC20, SNAP25, UNC13A, VGF, CRTC1, NEURL1, SYP, CPLX2, AGT, SYT7, PPFIA3 | 349 | 140 | 16792 | 3.780434 | 0.957211 | 0.054317 | 0.053367 |
| GOTERM_BP_ALL | GO:0048856~anatomical structure development | 143 | 33.41121 | 7.04E-04 | SPON2, CRTC1, HDAC11, PLEKHB1, SERPINE1, NUDT1, SCX, STMN3, MSI1, TPGS1, AQP3, CDC20, RPS19, SOX18, SALL4, PHLDA2, TNFRSF4, MNX1, EPHB3, SLC39A3, IER3, CHRNB2, TPRA1, DGAT2, UNC13A, LIG1, SERPINF2, SOX12, MYO7A, BHLHA15, MAPK8IP2, SRCIN1, FOXP3, PGF, CDC25B, LOC102724428, EID2, CLDN3, HOXB9, RFX6, TIMELESS, ORAI1, SIK1, HOXB2, REG3A, TPH1, BEX1, PRKDC, FOXO6, WDR62, CPLX2, SEZ6L2, NME1-NME2, VTN, TRPM2, ADGRG1, ZNHIT2, HOXC9, HOXC6, CBX2, SMAD9, NBEAL2, NR0B2, TMEM198, SDK1, ARTN, CDK4, MAFG, SP5, MELTF, MESP1, SNAP25, DAGLA, CELF4, BICDL1, MYL6B, SHB, HOXC10, AACS, RND1, DUSP10, HEY1, ADAMTSL2, DACT2, DGCR6, TEAD4, TGIF1, EMX1, MYOC, TESC, H2AFX, APOA2, KCNAB2, ADRA2C, ETV4, MTSS1, MMP11, VGF, CHI3L1, TNFRSF25, PAFAH1B3, CEBPB, LRP5, GDPD5, ASCL1, CRIP1, ASCL2, RELB, AURKA, DLL3, DLL4, RECQL4, CST3, PARD6A, MTHFD1L, RAB26, UCP2, E2F1, RAD54L, CTHRC1, HES4, CMTM8, LRRC24, NPM2, CDKN2A, GDF15, IL34, TMEM176B, TMEM176A, STAC3, CCK, AGT, MAD2L2, HEYL, NFASC, SMOC1, MFAP2, NEURL1, TACC3, INSM1, KCNK3, CDK5R2, FOXA2 | 349 | 5473 | 16792 | 1.257151 | 0.96157 | 0.055216 | 0.05425 |
| GOTERM_BP_ALL | GO:0051346~negative regulation of hydrolase activity | 20 | 4.672897 | 7.42E-04 | SPINK4, RTKN, PTPRN2, SERPINA1, PPP1R35, SERPINF2, SERPINA10, SERPINE1, APOA2, PKMYT1, WFDC2, AGT, CST3, VTN, PCSK1N, CST1, PTTG1, APOC1, RIMBP2, DPEP1 | 349 | 401 | 16792 | 2.399731 | 0.967854 | 0.05708 | 0.056081 |
| GOTERM_BP_ALL | GO:0006639~acylglycerol metabolic process | 10 | 2.336449 | 7.84E-04 | LIPF, LOC102724428, SCARB1, LIPE, FABP4, DGAT2, DAGLA, APOC1, APOA2, SIK1 | 349 | 118 | 16792 | 4.07751 | 0.973463 | 0.05708 | 0.056081 |
| GOTERM_BP_ALL | GO:0015850~organic hydroxy compound transport | 13 | 3.037383 | 7.84E-04 | CHRNB2, SCARB1, CADPS, APOA2, ABCA7, ADRA2C, AQP3, SYT7, AGT, APOC1, SLC18A1, ABCG1, STX1A | 349 | 195 | 16792 | 3.207641 | 0.973505 | 0.05708 | 0.056081 |
| GOTERM_BP_ALL | GO:0071825~protein-lipid complex subunit organization | 6 | 1.401869 | 8.26E-04 | SCARB1, APOC1, APOA2, ABCA7, ABCG1, AGT | 349 | 36 | 16792 | 8.019102 | 0.978159 | 0.05708 | 0.056081 |
| GOTERM_BP_ALL | GO:0006638~neutral lipid metabolic process | 10 | 2.336449 | 8.33E-04 | LIPF, LOC102724428, SCARB1, LIPE, FABP4, DGAT2, DAGLA, APOC1, APOA2, SIK1 | 349 | 119 | 16792 | 4.043245 | 0.978851 | 0.05708 | 0.056081 |
| GOTERM_BP_ALL | GO:0099537~trans-synaptic signaling | 26 | 6.074766 | 8.39E-04 | SNAP25, CRTC1, CELF4, C2CD4A, C2CD4B, C2CD4C, TPGS1, CPLX2, CPLX1, CDC20, SLC18A1, PPFIA3, CHRNB2, PTPRN2, UNC13A, GAD1, CADPS, STAC3, SYP, MAPK8IP2, SYT7, AGT, VGF, NEURL1, KCNK3, STX1A | 349 | 604 | 16792 | 2.071159 | 0.979412 | 0.05708 | 0.056081 |
| GOTERM_BP_ALL | GO:0007268~chemical synaptic transmission | 26 | 6.074766 | 8.39E-04 | SNAP25, CRTC1, CELF4, C2CD4A, C2CD4B, C2CD4C, TPGS1, CPLX2, CPLX1, CDC20, SLC18A1, PPFIA3, CHRNB2, PTPRN2, UNC13A, GAD1, CADPS, STAC3, SYP, MAPK8IP2, SYT7, AGT, VGF, NEURL1, KCNK3, STX1A | 349 | 604 | 16792 | 2.071159 | 0.979412 | 0.05708 | 0.056081 |
| GOTERM_BP_ALL | GO:0098916~anterograde trans-synaptic signaling | 26 | 6.074766 | 8.39E-04 | SNAP25, CRTC1, CELF4, C2CD4A, C2CD4B, C2CD4C, TPGS1, CPLX2, CPLX1, CDC20, SLC18A1, PPFIA3, CHRNB2, PTPRN2, UNC13A, GAD1, CADPS, STAC3, SYP, MAPK8IP2, SYT7, AGT, VGF, NEURL1, KCNK3, STX1A | 349 | 604 | 16792 | 2.071159 | 0.979412 | 0.05708 | 0.056081 |
| GOTERM_BP_ALL | GO:0099536~synaptic signaling | 26 | 6.074766 | 8.39E-04 | SNAP25, CRTC1, CELF4, C2CD4A, C2CD4B, C2CD4C, TPGS1, CPLX2, CPLX1, CDC20, SLC18A1, PPFIA3, CHRNB2, PTPRN2, UNC13A, GAD1, CADPS, STAC3, SYP, MAPK8IP2, SYT7, AGT, VGF, NEURL1, KCNK3, STX1A | 349 | 604 | 16792 | 2.071159 | 0.979412 | 0.05708 | 0.056081 |
| GOTERM_BP_ALL | GO:0046883~regulation of hormone secretion | 15 | 3.504673 | 8.77E-04 | SNAP25, KCNJ11, ABCC8, LRP5, CACNA1D, ADRA2C, NR0B2, AACS, SYT7, TRPM2, RFX6, UCP2, FFAR2, STX1A, FOXA2 | 349 | 254 | 16792 | 2.841414 | 0.982773 | 0.057993 | 0.056978 |
| GOTERM_BP_ALL | GO:0051648~vesicle localization | 15 | 3.504673 | 8.77E-04 | MAP4K2, SNAP25, SERPINA1, UNC13A, CADPS, C2CD4A, C2CD4B, C2CD4C, MYO7A, TPGS1, CPLX2, AP3B2, CPLX1, SYT7, STX1A | 349 | 254 | 16792 | 2.841414 | 0.982773 | 0.057993 | 0.056978 |
| GOTERM_BP_ALL | GO:0031324~negative regulation of cellular metabolic process | 71 | 16.58879 | 9.01E-04 | MESP1, ZNF296, SERPINA1, SERPINE1, SERPINA10, CELF4, TONSL, SCX, SHB, WFDC2, CDC20, DUSP10, PTTG1, HEY1, SOX18, SALL4, TNFRSF4, TGIF1, SPINK4, DGAT2, TESC, H2AFX, SERPINF2, SRCIN1, FOXP3, MAPK8IP1, LOC102724428, EID2, TIMELESS, DPEP1, TRIB3, SIK1, ANAPC5, CEBPB, UHRF1, PRKDC, PPP1R35, LRP5, PKMYT1, ASCL1, ASCL2, RELB, DLL4, CST3, VTN, CST1, PCSK1N, PARD6A, SOCS1, PCBP3, DHX34, E2F1, PRAME, HOXC6, PLK3, CDKN2A, UBE2C, CBX2, ABCA7, NR0B2, AGT, PARP10, MAD2L2, DRAP1, HEYL, FABP4, APOC1, RIMBP2, MAP3K10, INSM1, FOXA2 | 349 | 2345 | 16792 | 1.456775 | 0.984604 | 0.058757 | 0.057729 |
| GOTERM_BP_ALL | GO:0031100~organ regeneration | 8 | 1.869159 | 9.92E-04 | VTN, CEBPB, CDK4, UCP2, APOA2, NR0B2, PGF, AURKA | 349 | 76 | 16792 | 5.064696 | 0.989885 | 0.06347 | 0.062359 |
| GOTERM_BP_ALL | GO:0048522~positive regulation of cellular process | 126 | 29.43925 | 0.001013 | SCARB1, CRTC1, REG1A, SERPINE1, SCX, SCAMP5, CDC20, RPS19, SOX18, SALL4, ZXDC, TNFRSF4, EPHB3, CHRNB2, KCNH2, DGAT2, UNC13A, SERPINF2, SOX12, BHLHA15, MAPK8IP2, SRCIN1, RUNDC3A, FOXP3, PGF, MAPK8IP1, CDC25B, LOC102724428, HOXB9, RFX6, CCNE1, TIMELESS, SIK1, TRIB3, ANAPC5, STX1A, REG3A, SHC2, TPH1, BEX1, SHMT2, PRKDC, CDCA5, FOXO6, C2, NME1-NME2, VTN, TRPM2, ADGRG1, SOCS1, BMF, PLK3, UBE2C, STARD10, SMAD9, ABCA7, NR0B2, SYT7, TMEM198, UBE2S, CDK4, MAFG, MAP3K10, MELTF, FGFR4, RNF187, MESP1, SNAP25, DYRK2, ZC3H3, CELF4, SHB, HOXC10, AACS, HEY1, FFAR2, MYBL2, MAP4K2, MYOC, MMP1, TESC, H2AFX, APOA2, ADRA2C, ETV4, CHI3L1, TNFRSF25, PFDN2, CEBPB, UHRF1, LRP5, DBF4B, GDPD5, ASCL1, CLN6, RELB, AURKA, DLL4, CST3, P2RY6, UCP2, E2F1, PRAME, POLR2I, CTHRC1, LRRC24, NPM2, KCNJ11, ZNF580, CDKN2A, GDF15, IL34, ARID3A, CCK, GUCA2A, AGT, MAD2L2, HEYL, PRC1, RAB15, SHKBP1, APOC1, NEURL1, INSM1, CDK5R2, FOXA2 | 349 | 4744 | 16792 | 1.277918 | 0.990839 | 0.06347 | 0.062359 |
| GOTERM_BP_ALL | GO:0009892~negative regulation of metabolic process | 75 | 17.52336 | 0.001015 | MESP1, ZNF296, SERPINA1, SERPINE1, SERPINA10, CELF4, TONSL, SCX, SHB, WFDC2, CDC20, DUSP10, PTTG1, HEY1, RPS19, SOX18, SALL4, TNFRSF4, TGIF1, SPINK4, DGAT2, NUP210, TESC, H2AFX, SERPINF2, APOA2, SRCIN1, FOXP3, MAPK8IP1, LOC102724428, EID2, TIMELESS, DPEP1, TRIB3, SIK1, ANAPC5, CEBPB, UHRF1, PRKDC, PPP1R35, LRP5, PKMYT1, ASCL1, ASCL2, RELB, DLL4, CST3, VTN, CST1, PCSK1N, PARD6A, SOCS1, PCBP3, DHX34, E2F1, PRAME, POLR2I, HOXC6, PLK3, CDKN2A, UBE2C, CBX2, ABCA7, NR0B2, AGT, PARP10, MAD2L2, DRAP1, HEYL, FABP4, APOC1, RIMBP2, MAP3K10, INSM1, FOXA2 | 349 | 2522 | 16792 | 1.430847 | 0.990897 | 0.06347 | 0.062359 |
| GOTERM_BP_ALL | GO:0097006~regulation of plasma lipoprotein particle levels | 7 | 1.635514 | 0.001029 | SCARB1, DGAT2, APOC1, APOA2, ABCA7, ABCG1, AGT | 349 | 56 | 16792 | 6.014327 | 0.991495 | 0.063528 | 0.062417 |
| GOTERM_BP_ALL | GO:0032501~multicellular organismal process | 175 | 40.88785 | 0.001172 | SPON2, SCARB1, ZNF296, CRTC1, HDAC11, PLEKHB1, SERPINE1, NUDT1, SCX, STMN3, MSI1, TPGS1, AQP3, SCAMP5, CDC20, PTTG1, RPS19, SOX18, SALL4, PHLDA2, TNFRSF4, MNX1, EPHB3, SLC39A3, CHRNB2, KCNH2, TPRA1, DGAT2, UNC13A, LIG1, SERPINF2, SOX12, MYO7A, MAPK8IP2, SRCIN1, PGC, FOXP3, PGF, TRAIP, CDC25B, LOC102724428, EID2, HOXB9, RFX6, TIMELESS, ORAI1, SIK1, HOXB2, ABCG1, REG3A, TPH1, BEX1, PRKDC, FOXO6, CACNA1D, WDR62, CPLX2, SEZ6L2, NME1-NME2, VTN, TRPM2, ADGRG1, PCSK1N, ADAMTS14, ZNHIT2, SOCS1, HOXC9, HOXC6, CBX2, SMAD9, ABCA7, NBEAL2, PGA3, NR0B2, PGA5, SYT7, PGA4, TMEM198, SDK1, ARTN, CDK4, MAFG, FXYD2, SP5, MESP1, SNAP25, SERPINA1, DAGLA, SERPINA10, CELF4, BICDL1, MYL6B, SHB, HOXC10, WFDC2, AACS, RND1, DUSP10, HEY1, ADAMTSL2, DACT2, FFAR2, DGCR6, TEAD4, TGIF1, EMX1, MYOC, MMP1, GP1BB, TESC, H2AFX, APOA2, KCNAB2, ADRA2C, ETV4, MTSS1, MMP11, VGF, VWA1, CHI3L1, TNFRSF25, PAFAH1B3, CEBPB, RGS16, LRP5, ATP1A3, RRBP1, TIMM10, GDPD5, ASCL1, CRIP1, ASCL2, CLN6, RELB, AURKA, DLL3, DLL4, RECQL4, CST3, CST1, PARD6A, MTHFD1L, RAB26, UCP2, E2F1, RAD54L, POLR2I, CTHRC1, HES4, OR51E1, CMTM8, LRRC24, NPM2, KCNJ11, ZNF580, CDKN2A, IL34, TMEM176B, TMEM176A, STAC3, CCK, AGT, MAD2L2, HEYL, NFASC, FABP4, SMOC1, APOC1, MFAP2, NEURL1, TACC3, INSM1, KCNK3, CDK5R2, FOXA2 | 349 | 7027 | 16792 | 1.198244 | 0.995611 | 0.071388 | 0.070139 |
| GOTERM_BP_ALL | GO:0032502~developmental process | 145 | 33.8785 | 0.001224 | SPON2, CRTC1, HDAC11, PLEKHB1, SERPINE1, NUDT1, SCX, STMN3, MSI1, TPGS1, AQP3, CDC20, RPS19, SOX18, SALL4, PHLDA2, TNFRSF4, MNX1, EPHB3, SLC39A3, IER3, CHRNB2, TPRA1, DGAT2, UNC13A, LIG1, SERPINF2, SOX12, MYO7A, BHLHA15, MAPK8IP2, SRCIN1, FOXP3, PGF, CDC25B, LOC102724428, EID2, CLDN3, HOXB9, RFX6, TIMELESS, ORAI1, SIK1, HOXB2, REG3A, TPH1, BEX1, PRKDC, FOXO6, WDR62, CPLX2, SEZ6L2, NME1-NME2, VTN, TRPM2, ADGRG1, ZNHIT2, HOXC9, HOXC6, CBX2, SMAD9, NBEAL2, NR0B2, TMEM198, SDK1, ARTN, CDK4, MAFG, SP5, MELTF, MESP1, SNAP25, DAGLA, CELF4, BICDL1, MYL6B, SHB, HOXC10, AACS, RND1, DUSP10, HEY1, ADAMTSL2, DACT2, DGCR6, TEAD4, TGIF1, EMX1, MYOC, TESC, H2AFX, APOA2, KCNAB2, SYP, ADRA2C, ETV4, MTSS1, MMP11, VGF, CHI3L1, TNFRSF25, PAFAH1B3, CEBPB, LRP5, GDPD5, ASCL1, CRIP1, ASCL2, RELB, AURKA, DLL3, DLL4, RECQL4, CST3, PARD6A, MTHFD1L, RAB26, UCP2, E2F1, RAD54L, POLR2I, CTHRC1, HES4, CMTM8, LRRC24, NPM2, CDKN2A, GDF15, IL34, TMEM176B, TMEM176A, STAC3, CCK, AGT, MAD2L2, HEYL, NFASC, SMOC1, MFAP2, NEURL1, TACC3, INSM1, KCNK3, CDK5R2, FOXA2 | 349 | 5632 | 16792 | 1.238746 | 0.996554 | 0.0736 | 0.072312 |
| GOTERM_BP_ALL | GO:0046340~diacylglycerol catabolic process | 3 | 0.700935 | 0.001267 | LIPE, DAGLA, APOA2 | 349 | 3 | 16792 | 48.11461 | 0.997176 | 0.074774 | 0.073466 |
| GOTERM_BP_ALL | GO:0010872~regulation of cholesterol esterification | 4 | 0.934579 | 0.001287 | APOC1, APOA2, ABCG1, AGT | 349 | 11 | 16792 | 17.49622 | 0.99742 | 0.074774 | 0.073466 |
| GOTERM_BP_ALL | GO:0048285~organelle fission | 26 | 6.074766 | 0.001292 | CDCA5, LRP5, HEPACAM2, PKMYT1, CENPA, NCAPH, AURKA, CDC20, PTTG1, RAD54L, MYBL2, TXNL4A, SAC3D1, NPM2, UBE2C, CDC25B, MAD2L2, KIF18B, UBE2S, PRC1, INCENP, TIMELESS, TACC3, FAM64A, ANAPC5, KIF2C | 349 | 622 | 16792 | 2.011222 | 0.997486 | 0.074774 | 0.073466 |
| GOTERM_BP_ALL | GO:0006641~triglyceride metabolic process | 9 | 2.102804 | 0.001352 | LIPF, LOC102724428, SCARB1, LIPE, FABP4, DGAT2, APOC1, APOA2, SIK1 | 349 | 103 | 16792 | 4.20419 | 0.998092 | 0.075777 | 0.074451 |
| GOTERM_BP_ALL | GO:0034369~plasma lipoprotein particle remodeling | 5 | 1.168224 | 0.001388 | SCARB1, APOC1, APOA2, ABCG1, AGT | 349 | 24 | 16792 | 10.02388 | 0.998384 | 0.075777 | 0.074451 |
| GOTERM_BP_ALL | GO:0034368~protein-lipid complex remodeling | 5 | 1.168224 | 0.001388 | SCARB1, APOC1, APOA2, ABCG1, AGT | 349 | 24 | 16792 | 10.02388 | 0.998384 | 0.075777 | 0.074451 |
| GOTERM_BP_ALL | GO:0034367~macromolecular complex remodeling | 5 | 1.168224 | 0.001388 | SCARB1, APOC1, APOA2, ABCG1, AGT | 349 | 24 | 16792 | 10.02388 | 0.998384 | 0.075777 | 0.074451 |
| GOTERM_BP_ALL | GO:0050803~regulation of synapse structure or activity | 14 | 3.271028 | 0.001391 | CHRNB2, SNAP25, LRRC24, UNC13A, CRTC1, SYP, CPLX2, SYT7, AGT, CDC20, VGF, NEURL1, PPFIA3, EPHB3 | 349 | 237 | 16792 | 2.842213 | 0.998412 | 0.075777 | 0.074451 |
| GOTERM_BP_ALL | GO:0048518~positive regulation of biological process | 137 | 32.00935 | 0.001436 | SPON2, SCARB1, CRTC1, REG1A, SERPINE1, SCX, AQP3, SCAMP5, CDC20, RPS19, SOX18, SALL4, ZXDC, TNFRSF4, EPHB3, CHRNB2, KCNH2, DGAT2, UNC13A, SERPINF2, SOX12, BHLHA15, MAPK8IP2, SRCIN1, PGC, RUNDC3A, FOXP3, PGF, MAPK8IP1, CDC25B, LOC102724428, HOXB9, RFX6, CCNE1, TIMELESS, ORAI1, SIK1, TRIB3, ANAPC5, RELL2, ABCG1, STX1A, REG3A, SHC2, TPH1, BEX1, SHMT2, PRKDC, CDCA5, FOXO6, CACNA1D, C2, NME1-NME2, VTN, TRPM2, ADGRG1, SOCS1, BMF, PLK3, UBE2C, STARD10, SMAD9, ABCA7, NR0B2, SYT7, TMEM198, ARTN, UBE2S, CDK4, MAFG, MAP3K10, MELTF, FGFR4, RNF187, MESP1, SNAP25, DYRK2, ZC3H3, CELF4, SHB, HOXC10, AACS, HEY1, FFAR2, MYBL2, MAP4K2, MYOC, ABCC8, MMP1, TESC, H2AFX, APOA2, ADRA2C, ETV4, CHI3L1, TNFRSF25, PFDN2, CEBPB, UHRF1, LRP5, DBF4B, GDPD5, ASCL1, CLN6, RELB, AURKA, DLL4, CST3, P2RY6, PARD6A, UCP2, E2F1, PRAME, POLR2I, CTHRC1, LRRC24, NPM2, KCNJ11, ZNF580, CDKN2A, GDF15, IL34, ARID3A, CCK, GUCA2A, AGT, MAD2L2, HEYL, FABP4, PRC1, RAB15, SHKBP1, APOC1, NEURL1, INSM1, CDK5R2, FOXA2 | 349 | 5285 | 16792 | 1.247247 | 0.998708 | 0.077291 | 0.075938 |
| GOTERM_BP_ALL | GO:0048869~cellular developmental process | 108 | 25.23364 | 0.001592 | SPON2, CRTC1, HDAC11, SERPINE1, SCX, STMN3, TPGS1, AQP3, CDC20, RPS19, SOX18, MNX1, EPHB3, SLC39A3, CHRNB2, UNC13A, SERPINF2, SOX12, MYO7A, BHLHA15, MAPK8IP2, SRCIN1, FOXP3, PGF, CDC25B, LOC102724428, EID2, CLDN3, RFX6, SIK1, TRIB3, ABCG1, REG3A, TPH1, BEX1, PRKDC, FOXO6, WDR62, CPLX2, NME1-NME2, VTN, TRPM2, ADGRG1, ZNHIT2, SOCS1, CBX2, NBEAL2, SDK1, ARTN, MAFG, MELTF, MESP1, SNAP25, DAGLA, CELF4, BICDL1, SHB, HOXC10, AACS, RND1, DUSP10, HEY1, DACT2, FFAR2, MYBL2, EMX1, MYOC, TESC, H2AFX, C10ORF11, KCNAB2, ADRA2C, ETV4, MTSS1, MMP11, CEBPB, LRP5, RRBP1, GDPD5, ASCL1, ASCL2, RELB, AURKA, DLL3, DLL4, CST3, E2F1, PRAME, CTHRC1, HES4, NPM2, CDKN2A, GDF15, IL34, TMEM176B, TMEM176A, STAC3, CCK, AGT, MAD2L2, HEYL, NFASC, FABP4, SMOC1, NEURL1, INSM1, CDK5R2, FOXA2 | 349 | 3992 | 16792 | 1.301698 | 0.999374 | 0.084709 | 0.083227 |
| GOTERM_BP_ALL | GO:0008284~positive regulation of cell proliferation | 32 | 7.476636 | 0.001632 | REG3A, SHMT2, PRKDC, REG1A, LRP5, SCX, DBF4B, ASCL1, HOXC10, NME1-NME2, CDC20, DLL4, CST3, ADGRG1, E2F1, PRAME, TNFRSF4, CTHRC1, CHRNB2, ZNF580, IL34, SERPINF2, CCK, FOXP3, PGF, AGT, CDC25B, PRC1, CDK4, INSM1, FGFR4, RNF187 | 349 | 849 | 16792 | 1.813507 | 0.999479 | 0.085826 | 0.084324 |
| GOTERM_BP_ALL | GO:0015748~organophosphate ester transport | 8 | 1.869159 | 0.001666 | SCARB1, G6PC3, APOC1, STARD10, APOA2, ABCA7, ATP11A, ABCG1 | 349 | 83 | 16792 | 4.637553 | 0.999555 | 0.085855 | 0.084353 |
| GOTERM_BP_ALL | GO:0051650~establishment of vesicle localization | 14 | 3.271028 | 0.001669 | MAP4K2, SNAP25, SERPINA1, UNC13A, CADPS, C2CD4A, C2CD4B, C2CD4C, MYO7A, CPLX2, AP3B2, CPLX1, SYT7, STX1A | 349 | 242 | 16792 | 2.78349 | 0.999562 | 0.085855 | 0.084353 |
| GOTERM_BP_ALL | GO:0015914~phospholipid transport | 7 | 1.635514 | 0.001757 | SCARB1, APOC1, STARD10, APOA2, ABCA7, ATP11A, ABCG1 | 349 | 62 | 16792 | 5.432295 | 0.999708 | 0.089366 | 0.087803 |
| GOTERM_BP_ALL | GO:0006887~exocytosis | 19 | 4.439252 | 0.001995 | EXOC3L1, SNAP25, SERPINA1, UNC13A, SERPINF2, CADPS, SERPINE1, C2CD4A, C2CD4B, C2CD4C, CPLX2, SRCIN1, CPLX1, SYT7, SCAMP5, RAB26, RAB15, CDK5R2, STX1A | 349 | 404 | 16792 | 2.262816 | 0.999903 | 0.100394 | 0.098637 |
| GOTERM_BP_ALL | GO:0007399~nervous system development | 65 | 15.18692 | 0.002092 | SPON2, SNAP25, MESP1, DAGLA, CRTC1, HDAC11, BICDL1, STMN3, MSI1, HOXC10, RND1, CDC20, DUSP10, SALL4, MNX1, EPHB3, CHRNB2, EMX1, UNC13A, MYOC, H2AFX, SOX12, MYO7A, ADRA2C, MAPK8IP2, ETV4, SRCIN1, HOXB2, PAFAH1B3, CEBPB, BEX1, PRKDC, FOXO6, WDR62, GDPD5, ASCL1, CPLX2, SEZ6L2, ASCL2, AURKA, DLL3, NME1-NME2, DLL4, CST3, VTN, ADGRG1, ZNHIT2, MTHFD1L, E2F1, CTHRC1, HES4, CMTM8, LRRC24, SMAD9, CCK, HEYL, SDK1, NFASC, ARTN, NEURL1, TACC3, INSM1, KCNK3, CDK5R2, FOXA2 | 349 | 2170 | 16792 | 1.441221 | 0.999938 | 0.104127 | 0.102305 |
| GOTERM_BP_ALL | GO:0050804~modulation of synaptic transmission | 15 | 3.504673 | 0.002193 | CHRNB2, SNAP25, UNC13A, CRTC1, CELF4, SYP, MAPK8IP2, CPLX2, SYT7, AGT, CDC20, VGF, NEURL1, STX1A, PPFIA3 | 349 | 280 | 16792 | 2.577569 | 0.999961 | 0.107985 | 0.106095 |
| GOTERM_BP_ALL | GO:0032879~regulation of localization | 72 | 16.82243 | 0.002326 | SCARB1, SNAP25, DYRK2, ZC3H3, SERPINE1, TONSL, AACS, SCAMP5, RPS19, FFAR2, PHLDA2, TNFRSF4, KCNH2, CHRNB2, UNC13A, KCNH6, MYOC, NUP210, ABCC8, TESC, APOA2, KCNAB2, ADRA2C, MAPK8IP2, SRCIN1, FOXP3, PGF, LOC102724428, RFX6, DPEP1, ORAI1, TRIB3, SIK1, ABCG1, STX1A, CDCA5, LRP5, C2CD4A, CACNA1D, C2CD4B, C2CD4C, CPLX2, CPLX1, C2, DLL4, VTN, TRPM2, P2RY6, ADGRG1, PARD6A, SOCS1, RAB26, UCP2, E2F1, BMF, PLK3, KCNJ11, ZNF580, CDKN2A, ASPSCR1, ABCA7, CCK, NR0B2, AGT, SYT7, PARP10, RAB15, FXYD2, APOC1, INSM1, CDK5R2, FOXA2 | 349 | 2473 | 16792 | 1.40083 | 0.999979 | 0.113357 | 0.111373 |
| GOTERM_BP_ALL | GO:0044770~cell cycle phase transition | 23 | 5.373832 | 0.002442 | PLK3, CDT1, TPRA1, NPM2, CDKN2A, PRKDC, UBE2C, CDCA5, ARID3A, DBF4B, PKMYT1, CDC25B, AURKA, MAD2L2, CDC20, CCNE1, UBE2S, CDK4, E2F1, TIMELESS, TACC3, ANAPC5, MCM2 | 349 | 547 | 16792 | 2.023101 | 0.999988 | 0.117726 | 0.115666 |
| GOTERM_BP_ALL | GO:0065009~regulation of molecular function | 81 | 18.92523 | 0.002493 | SCARB1, SERPINA1, RTKN, CRTC1, SERPINE1, SERPINA10, STMN3, COX6A1, SHB, WFDC2, CDC20, DUSP10, RINL, PTTG1, HEY1, SPR, TNFRSF4, EPHB3, SPINK4, PLEKHG4, MAP4K2, TESC, SERPINF2, APOA2, ADRA2C, MAPK8IP2, RUNDC3A, SRCIN1, FOXP3, MAPK8IP1, CDC25B, LOC102724428, CCNE1, DPEP1, CHI3L1, TRIB3, SIK1, ANAPC5, SHC2, BEX1, UHRF1, PPP1R35, RGS16, LRP5, CACNA1D, DBF4B, PKMYT1, AURKA, CST3, VTN, CST1, PCSK1N, SOCS1, E2F1, CTHRC1, PPP1R14B, PTPRN2, PPP1R14C, NPM2, KCNJ11, CDKN2A, UBE2C, CCK, NR0B2, GUCA2A, AGT, PARP10, MAD2L2, HEYL, FABP4, UBE2S, ARTN, CDK4, FXYD2, NEURL1, APOC1, RIMBP2, MAP3K10, FGFR4, CDK5R2, FOXA2 | 349 | 2863 | 16792 | 1.361259 | 0.99999 | 0.118993 | 0.116911 |
| GOTERM_BP_ALL | GO:0044772~mitotic cell cycle phase transition | 22 | 5.140187 | 0.002527 | PLK3, CDT1, TPRA1, NPM2, CDKN2A, PRKDC, UBE2C, CDCA5, ARID3A, DBF4B, PKMYT1, CDC25B, AURKA, MAD2L2, CDC20, CCNE1, UBE2S, CDK4, E2F1, TACC3, ANAPC5, MCM2 | 349 | 514 | 16792 | 2.05938 | 0.999992 | 0.119366 | 0.117277 |
| GOTERM_BP_ALL | GO:0033700~phospholipid efflux | 4 | 0.934579 | 0.00271 | APOC1, APOA2, ABCA7, ABCG1 | 349 | 14 | 16792 | 13.74703 | 0.999997 | 0.126732 | 0.124514 |
| GOTERM_BP_ALL | GO:0006260~DNA replication | 15 | 3.504673 | 0.003023 | GINS2, RTEL1-TNFRSF6B, CDT1, RNASEH2A, NPM2, LIG1, TONSL, DBF4B, RECQL4, CST3, CCNE1, POLD1, TIMELESS, DTD1, MCM2 | 349 | 290 | 16792 | 2.488687 | 0.999999 | 0.139162 | 0.136727 |
| GOTERM_BP_ALL | GO:0015844~monoamine transport | 7 | 1.635514 | 0.003036 | CHRNB2, CADPS, SLC29A4, ADRA2C, SLC18A1, STX1A, AGT | 349 | 69 | 16792 | 4.881193 | 0.999999 | 0.139162 | 0.136727 |
| GOTERM_BP_ALL | GO:0042127~regulation of cell proliferation | 49 | 11.4486 | 0.003221 | REG1A, SCX, HOXC10, CDC20, SOX18, TNFRSF4, CHRNB2, EMX1, TESC, SERPINF2, ETV4, FOXP3, MTSS1, PGF, CDC25B, EID2, TIMELESS, TNFRSF25, REG3A, CEBPB, SHMT2, PRKDC, LRP5, DPT, DBF4B, ASCL1, ASCL2, NME1-NME2, DLL4, CST3, ADGRG1, E2F1, PRAME, CTHRC1, ZNF580, CDKN2A, IL34, CCK, AGT, PARP10, PRC1, CDK4, MAFG, FXYD2, NEURL1, MELTF, INSM1, FGFR4, RNF187 | 349 | 1552 | 16792 | 1.519083 | 1 | 0.145713 | 0.143163 |
| GOTERM_BP_ALL | GO:0007049~cell cycle | 52 | 12.14953 | 0.003242 | HEPACAM2, SHB, CDC20, PTTG1, MYBL2, TXNL4A, TPRA1, LIG1, NUP210, H2AFX, CDC25B, LOC102724428, CCNE1, INCENP, TIMELESS, SIK1, FAM64A, ANAPC5, KIF2C, MCM2, BEX2, UHRF1, PRKDC, CDCA5, LRP5, DBF4B, WDR62, PKMYT1, GDPD5, ASCL1, CENPA, NCAPH, AURKA, PARD6A, E2F1, RAD54L, SAC3D1, GINS2, CDT1, PLK3, NPM2, CDKN2A, UBE2C, ARID3A, MAD2L2, KIF18B, UBE2S, PRC1, CDK4, TACC3, INSM1, CDK5R2 | 349 | 1672 | 16792 | 1.496387 | 1 | 0.145713 | 0.143163 |
| GOTERM_BP_ALL | GO:0034375~high-density lipoprotein particle remodeling | 4 | 0.934579 | 0.003336 | SCARB1, APOC1, APOA2, ABCG1 | 349 | 15 | 16792 | 12.83056 | 1 | 0.148501 | 0.145902 |
| GOTERM_BP_ALL | GO:0017157~regulation of exocytosis | 11 | 2.570093 | 0.003746 | SCAMP5, RAB26, RAB15, C2CD4A, C2CD4B, C2CD4C, CPLX2, CPLX1, STX1A, SYT7, CDK5R2 | 349 | 176 | 16792 | 3.007163 | 1 | 0.163805 | 0.160938 |
| GOTERM_BP_ALL | GO:0010951~negative regulation of endopeptidase activity | 13 | 3.037383 | 0.003751 | SPINK4, SERPINA1, SERPINF2, SERPINA10, SERPINE1, WFDC2, AGT, CST3, VTN, PCSK1N, CST1, PTTG1, DPEP1 | 349 | 235 | 16792 | 2.661659 | 1 | 0.163805 | 0.160938 |
| GOTERM_BP_ALL | GO:0022008~neurogenesis | 46 | 10.74766 | 0.00402 | SNAP25, MESP1, SPON2, CEBPB, DAGLA, CRTC1, HDAC11, BICDL1, FOXO6, STMN3, WDR62, GDPD5, ASCL1, ASCL2, HOXC10, AURKA, RND1, DLL3, NME1-NME2, CDC20, DLL4, VTN, ADGRG1, ZNHIT2, DUSP10, MNX1, EPHB3, CTHRC1, CHRNB2, EMX1, UNC13A, MYOC, CCK, MYO7A, ADRA2C, MAPK8IP2, ETV4, SRCIN1, HEYL, SDK1, NFASC, ARTN, NEURL1, INSM1, CDK5R2, FOXA2 | 349 | 1447 | 16792 | 1.529559 | 1 | 0.173321 | 0.170288 |
| GOTERM_BP_ALL | GO:0046339~diacylglycerol metabolic process | 4 | 0.934579 | 0.004044 | LIPE, DGAT2, DAGLA, APOA2 | 349 | 16 | 16792 | 12.02865 | 1 | 0.173321 | 0.170288 |
| GOTERM_BP_ALL | GO:0051239~regulation of multicellular organismal process | 75 | 17.52336 | 0.004154 | SPON2, SCARB1, SNAP25, MESP1, CRTC1, SERPINE1, CELF4, SCX, SHB, AQP3, SCAMP5, CDC20, DUSP10, HEY1, RPS19, FFAR2, PHLDA2, EPHB3, KCNH2, CHRNB2, EMX1, UNC13A, TESC, SERPINF2, APOA2, ADRA2C, ETV4, SRCIN1, FOXP3, PGF, TRAIP, CHI3L1, REG3A, CEBPB, TPH1, PRKDC, LRP5, FOXO6, ATP1A3, CACNA1D, GDPD5, ASCL1, ASCL2, AURKA, DLL3, RELB, NME1-NME2, DLL4, CST3, VTN, ADGRG1, PARD6A, SOCS1, CTHRC1, LRRC24, KCNJ11, ZNF580, CDKN2A, IL34, TMEM176B, TMEM176A, ABCA7, CCK, AGT, SYT7, MAD2L2, HEYL, SDK1, MAFG, SMOC1, FXYD2, NEURL1, APOC1, KCNK3, FOXA2 | 349 | 2659 | 16792 | 1.357125 | 1 | 0.176394 | 0.173308 |
| GOTERM_BP_ALL | GO:0030198~extracellular matrix organization | 16 | 3.738318 | 0.004292 | MMP1, SERPINF2, SERPINE1, SCX, DPT, AGT, CST3, MMP11, VTN, ADAMTS14, TTR, MFAP2, ADAMTSL2, VWA1, MELTF, FGFR4 | 349 | 334 | 16792 | 2.304892 | 1 | 0.180369 | 0.177213 |
| GOTERM_BP_ALL | GO:0009893~positive regulation of metabolic process | 84 | 19.62617 | 0.004325 | SCARB1, MESP1, DYRK2, CRTC1, SERPINE1, CELF4, SCX, CDC20, HEY1, RPS19, SOX18, SALL4, ZXDC, MYBL2, TNFRSF4, MAP4K2, DGAT2, TESC, H2AFX, SERPINF2, APOA2, SOX12, BHLHA15, ADRA2C, MAPK8IP2, RUNDC3A, ETV4, SRCIN1, FOXP3, CDC25B, HOXB9, RFX6, CCNE1, TIMELESS, CHI3L1, TNFRSF25, TRIB3, ANAPC5, ABCG1, CEBPB, SHC2, BEX1, UHRF1, PRKDC, LRP5, DBF4B, ASCL1, CLN6, AURKA, RELB, NME1-NME2, DLL4, CST3, VTN, SOCS1, E2F1, POLR2I, PLK3, NPM2, ZNF580, CDKN2A, GDF15, UBE2C, IL34, SMAD9, ARID3A, ABCA7, CCK, NR0B2, GUCA2A, AGT, MAD2L2, HEYL, UBE2S, CDK4, MAFG, NEURL1, APOC1, MAP3K10, MELTF, FGFR4, CDK5R2, RNF187, FOXA2 | 349 | 3052 | 16792 | 1.324255 | 1 | 0.180369 | 0.177213 |
| GOTERM_BP_ALL | GO:0043062~extracellular structure organization | 16 | 3.738318 | 0.00441 | MMP1, SERPINF2, SERPINE1, SCX, DPT, AGT, CST3, MMP11, VTN, ADAMTS14, TTR, MFAP2, ADAMTSL2, VWA1, MELTF, FGFR4 | 349 | 335 | 16792 | 2.298011 | 1 | 0.18226 | 0.179071 |
| GOTERM_BP_ALL | GO:0031325~positive regulation of cellular metabolic process | 79 | 18.45794 | 0.00447 | SCARB1, MESP1, DYRK2, CRTC1, SERPINE1, CELF4, SCX, CDC20, HEY1, SOX18, SALL4, ZXDC, MYBL2, TNFRSF4, MAP4K2, DGAT2, TESC, H2AFX, SERPINF2, APOA2, SOX12, BHLHA15, ADRA2C, MAPK8IP2, RUNDC3A, ETV4, SRCIN1, FOXP3, CDC25B, HOXB9, RFX6, CCNE1, TIMELESS, CHI3L1, TNFRSF25, TRIB3, ANAPC5, CEBPB, SHC2, BEX1, UHRF1, PRKDC, LRP5, DBF4B, ASCL1, CLN6, AURKA, RELB, NME1-NME2, CST3, VTN, SOCS1, E2F1, POLR2I, PLK3, NPM2, CDKN2A, GDF15, UBE2C, IL34, SMAD9, ARID3A, ABCA7, CCK, GUCA2A, AGT, MAD2L2, HEYL, UBE2S, CDK4, MAFG, NEURL1, APOC1, MAP3K10, MELTF, FGFR4, CDK5R2, RNF187, FOXA2 | 349 | 2839 | 16792 | 1.338871 | 1 | 0.183128 | 0.179924 |
| GOTERM_BP_ALL | GO:0017158~regulation of calcium ion-dependent exocytosis | 7 | 1.635514 | 0.0046 | SCAMP5, C2CD4A, C2CD4B, C2CD4C, STX1A, SYT7, CDK5R2 | 349 | 75 | 16792 | 4.490697 | 1 | 0.186796 | 0.183528 |
| GOTERM_BP_ALL | GO:0010866~regulation of triglyceride biosynthetic process | 4 | 0.934579 | 0.004836 | LOC102724428, SCARB1, DGAT2, SIK1 | 349 | 17 | 16792 | 11.32109 | 1 | 0.194645 | 0.191239 |
| GOTERM_BP_ALL | GO:0045787~positive regulation of cell cycle | 16 | 3.738318 | 0.00492 | PLK3, NPM2, CDKN2A, UBE2C, CDCA5, LRP5, ARID3A, DBF4B, GDPD5, ASCL1, SHB, CDC25B, AURKA, CDK4, E2F1, INSM1 | 349 | 339 | 16792 | 2.270896 | 1 | 0.196329 | 0.192893 |
| GOTERM_BP_ALL | GO:1901700~response to oxygen-containing compound | 46 | 10.74766 | 0.005012 | SPON2, SCARB1, CEBPB, PRKDC, SERPINE1, LRP5, ATP1A3, ASCL1, AQP3, AACS, NME1-NME2, CST3, TRPM2, P2RY6, SOCS1, DUSP10, GNG4, UCP2, E2F1, FFAR2, CYC1, TNFRSF4, CHRNB2, PLK3, ATP6V0B, PTPRN2, DGAT2, ZNF580, KCNJ11, ABCC8, TESC, APOA2, GGH, NR0B2, AGT, SDK1, VGF, RFX6, CDK4, NEURL1, TIMELESS, DPEP1, TNFRSF25, TRIB3, FGFR4, FOXA2 | 349 | 1466 | 16792 | 1.509735 | 1 | 0.198295 | 0.194825 |
| GOTERM_BP_ALL | GO:0051179~localization | 146 | 34.11215 | 0.005174 | SPON2, SCARB1, EXOC3L1, CPNE7, SERPINE1, TONSL, COX6A1, TPGS1, AQP3, IPO4, SCAMP5, RINL, RPS19, SOX18, PHLDA2, TNFRSF4, PPFIA3, EPHB3, SLC39A3, CHRNB2, KCNH2, DGAT2, UNC13A, KCNH6, NUP210, SERPINF2, MYO7A, BHLHA15, ATP11A, MAPK8IP2, SRCIN1, MFSD12, FOXP3, PGF, MAPK8IP1, LOC102724428, HOXB9, RFX6, DPEP1, ORAI1, SIK1, TRIB3, TSNARE1, ABCG1, STX1A, TTYH3, TMEM63C, CDCA5, C2CD4A, CACNA1D, C2CD4B, SLC38A11, C2CD4C, SLC1A5, CPLX2, CPLX1, TIMM50, C2, VTN, TRPM2, ADGRG1, SOCS1, BMF, CYC1, PLK3, PTPRN2, SLC52A2, STARD10, ASPSCR1, ABCA7, NR0B2, SYT7, PARP10, G6PC3, ARTN, FXYD2, MELTF, FGFR4, MESP1, SNAP25, SERPINA1, NXT1, DYRK2, PODXL2, GPAA1, ZC3H3, BICDL1, ASGR1, AACS, FFAR2, KIF1A, GRASP, SLC18A1, MAP4K2, ATP6V0B, MYOC, ABCC8, MMP1, TESC, APOA2, KCNAB2, SYP, ADRA2C, AP3B2, VGF, CHI3L1, SLC29A4, KIF2C, TMEM9, LRP5, ATP1A3, RRBP1, TIMM10, ASCL1, CENPA, SLC7A1, AURKA, DLL4, P2RY6, TTR, PARD6A, RAB26, UCP2, E2F1, SLC25A22, CTHRC1, CMTM8, KCNJ11, ZNF580, CDKN2A, GAD1, CADPS, CCK, AGT, FAM155B, NFASC, FABP4, SLC25A39, SLCO3A1, RAB15, APOC1, NEURL1, INSM1, KCNK3, CDK5R2, FOXA2 | 349 | 5872 | 16792 | 1.19631 | 1 | 0.202974 | 0.199422 |
| GOTERM_BP_ALL | GO:0048468~cell development | 58 | 13.5514 | 0.005545 | SPON2, SNAP25, CRTC1, HDAC11, CELF4, BICDL1, STMN3, SHB, TPGS1, RND1, CDC20, DUSP10, HEY1, RPS19, SOX18, DACT2, MNX1, EPHB3, CHRNB2, EMX1, UNC13A, MYOC, MYO7A, BHLHA15, ADRA2C, MAPK8IP2, ETV4, SRCIN1, CDC25B, CLDN3, PRKDC, LRP5, FOXO6, GDPD5, ASCL1, ASCL2, AURKA, DLL3, NME1-NME2, DLL4, CST3, ADGRG1, CTHRC1, NPM2, GDF15, STAC3, NBEAL2, CCK, AGT, MAD2L2, HEYL, SDK1, NFASC, ARTN, NEURL1, MELTF, INSM1, FOXA2 | 349 | 1968 | 16792 | 1.418012 | 1 | 0.214374 | 0.210623 |
| GOTERM_BP_ALL | GO:0010466~negative regulation of peptidase activity | 13 | 3.037383 | 0.005557 | SPINK4, SERPINA1, SERPINF2, SERPINA10, SERPINE1, WFDC2, AGT, CST3, VTN, PCSK1N, CST1, PTTG1, DPEP1 | 349 | 247 | 16792 | 2.532348 | 1 | 0.214374 | 0.210623 |
| GOTERM_BP_ALL | GO:0042493~response to drug | 18 | 4.205607 | 0.00563 | TGIF1, KCNH2, SPINK4, EMX1, KCNJ11, ABCC8, GAD1, APOA2, GGH, ATP1A3, AACS, PGF, CST3, CDK4, DPEP1, RAD54L, SLC18A1, KCNK3 | 349 | 411 | 16792 | 2.107209 | 1 | 0.215392 | 0.211623 |
| GOTERM_BP_ALL | GO:1901990~regulation of mitotic cell cycle phase transition | 15 | 3.504673 | 0.005732 | PLK3, TPRA1, NPM2, CDKN2A, PRKDC, UBE2C, CDCA5, ARID3A, DBF4B, AURKA, MAD2L2, CDC20, CDK4, E2F1, ANAPC5 | 349 | 312 | 16792 | 2.313203 | 1 | 0.216869 | 0.213075 |
| GOTERM_BP_ALL | GO:0022402~cell cycle process | 43 | 10.04673 | 0.005763 | PRKDC, CDCA5, LRP5, HEPACAM2, DBF4B, WDR62, PKMYT1, CENPA, NCAPH, AURKA, CDC20, PTTG1, E2F1, RAD54L, MYBL2, TXNL4A, SAC3D1, GINS2, CDT1, PLK3, TPRA1, NPM2, LIG1, NUP210, CDKN2A, UBE2C, H2AFX, ARID3A, CDC25B, MAD2L2, KIF18B, CCNE1, UBE2S, PRC1, INCENP, CDK4, TIMELESS, TACC3, FAM64A, ANAPC5, KIF2C, INSM1, MCM2 | 349 | 1357 | 16792 | 1.524634 | 1 | 0.216869 | 0.213075 |
| GOTERM_BP_ALL | GO:0090068~positive regulation of cell cycle process | 13 | 3.037383 | 0.006392 | PLK3, NPM2, CDKN2A, UBE2C, CDCA5, LRP5, ARID3A, DBF4B, CDC25B, AURKA, CDK4, E2F1, INSM1 | 349 | 251 | 16792 | 2.491992 | 1 | 0.238627 | 0.234451 |
| GOTERM_BP_ALL | GO:0006810~transport | 119 | 27.80374 | 0.006519 | SPON2, SCARB1, EXOC3L1, CPNE7, SERPINE1, TONSL, COX6A1, AQP3, IPO4, SCAMP5, RINL, RPS19, TNFRSF4, PPFIA3, SLC39A3, CHRNB2, KCNH2, UNC13A, KCNH6, NUP210, SERPINF2, MYO7A, BHLHA15, ATP11A, MAPK8IP2, SRCIN1, MFSD12, FOXP3, MAPK8IP1, LOC102724428, RFX6, ORAI1, SIK1, TRIB3, TSNARE1, ABCG1, STX1A, TTYH3, TMEM63C, C2CD4A, CACNA1D, C2CD4B, SLC38A11, C2CD4C, SLC1A5, CPLX2, CPLX1, TIMM50, C2, VTN, TRPM2, SOCS1, BMF, CYC1, PLK3, PTPRN2, SLC52A2, STARD10, ASPSCR1, ABCA7, NR0B2, SYT7, PARP10, G6PC3, FXYD2, MELTF, SNAP25, SERPINA1, NXT1, DYRK2, ZC3H3, BICDL1, ASGR1, AACS, FFAR2, KIF1A, SLC18A1, MAP4K2, ATP6V0B, ABCC8, TESC, APOA2, KCNAB2, SYP, ADRA2C, AP3B2, VGF, CHI3L1, SLC29A4, KIF2C, TMEM9, LRP5, ATP1A3, RRBP1, TIMM10, SLC7A1, P2RY6, TTR, RAB26, UCP2, E2F1, SLC25A22, KCNJ11, CDKN2A, GAD1, CADPS, CCK, AGT, FAM155B, NFASC, FABP4, SLC25A39, SLCO3A1, RAB15, APOC1, NEURL1, KCNK3, CDK5R2, FOXA2 | 349 | 4663 | 16792 | 1.227887 | 1 | 0.241418 | 0.237194 |
| GOTERM_BP_ALL | GO:0045956~positive regulation of calcium ion-dependent exocytosis | 4 | 0.934579 | 0.006683 | SCAMP5, STX1A, SYT7, CDK5R2 | 349 | 19 | 16792 | 10.12939 | 1 | 0.243058 | 0.238805 |
| GOTERM_BP_ALL | GO:0007219~Notch signaling pathway | 10 | 2.336449 | 0.006729 | DLL4, MESP1, HEYL, HEY1, RPS19, NEURL1, GDPD5, ASCL1, NR0B2, DLL3 | 349 | 162 | 16792 | 2.970038 | 1 | 0.243058 | 0.238805 |
| GOTERM_BP_ALL | GO:0051640~organelle localization | 20 | 4.672897 | 0.006766 | MAP4K2, SNAP25, SERPINA1, UNC13A, CDCA5, CADPS, C2CD4A, C2CD4B, C2CD4C, MYO7A, BHLHA15, TPGS1, CPLX2, CENPA, AP3B2, CPLX1, SYT7, AURKA, KIF2C, STX1A | 349 | 489 | 16792 | 1.967878 | 1 | 0.243058 | 0.238805 |
| GOTERM_BP_ALL | GO:0010605~negative regulation of macromolecule metabolic process | 66 | 15.42056 | 0.006773 | MESP1, ZNF296, SERPINA1, SERPINE1, SERPINA10, CELF4, TONSL, SCX, SHB, WFDC2, CDC20, DUSP10, PTTG1, HEY1, SOX18, SALL4, TNFRSF4, TGIF1, SPINK4, NUP210, TESC, H2AFX, SERPINF2, SRCIN1, FOXP3, MAPK8IP1, EID2, TIMELESS, DPEP1, TRIB3, ANAPC5, CEBPB, UHRF1, PRKDC, LRP5, ASCL1, ASCL2, RELB, DLL4, CST3, VTN, CST1, PCSK1N, PARD6A, SOCS1, PCBP3, DHX34, E2F1, PRAME, POLR2I, HOXC6, PLK3, CDKN2A, UBE2C, CBX2, ABCA7, NR0B2, AGT, PARP10, MAD2L2, DRAP1, HEYL, FABP4, MAP3K10, INSM1, FOXA2 | 349 | 2326 | 16792 | 1.365247 | 1 | 0.243058 | 0.238805 |
| GOTERM_BP_ALL | GO:0048732~gland development | 18 | 4.205607 | 0.006949 | MESP1, CEBPB, TPH1, PRKDC, LRP5, ASCL1, ETV4, CRIP1, AACS, AURKA, CST3, VTN, HOXB9, UCP2, NEURL1, ORAI1, INSM1, EPHB3 | 349 | 421 | 16792 | 2.057157 | 1 | 0.247421 | 0.243092 |
| GOTERM_BP_ALL | GO:0009987~cellular process | 335 | 78.27103 | 0.007068 | SPON2, ZNF296, CLPB, CPNE7, HDAC11, PLEKHB1, SERPINE1, PPAN, TONSL, NUDT1, SCX, STMN3, TPGS1, AQP3, RRP9, SCAMP5, LIPF, CDC20, LIPE, RPS19, SOX18, ZXDC, ARL6IP4, MNX1, EPHB3, IER3, SDS, UNC13A, NUP210, SERPINF2, SOX12, MYO7A, BHLHA15, CPT1C, MAPK8IP2, MAPK8IP1, SULT2B1, EID2, HOXB9, CLDN3, RFX6, ASPHD1, HMBS, CCDC86, ORAI1, SIK1, HOXB2, FAM64A, TRIB3, PLPP2, STX1A, BEX2, TPH1, BEX1, TTYH3, TMEM63C, SHMT2, PRKDC, PPP1R35, CDCA5, C2CD4A, C2CD4B, C2CD4C, WDR62, NCAPH, C2, TRPM2, ZNHIT2, ADAMTS14, PCBP3, DHX34, MIER2, DHX37, HOXC9, HOXC6, RTEL1-TNFRSF6B, CDT1, PLK3, PTPRN2, STARD10, ABCA7, NBEAL2, PGA3, NR0B2, PGA5, ST18, TMEM198, PGA4, PARP10, NDUFAF8, ARTN, PAH, FXYD2, FBXL6, PCSK1, MESP1, SERPINA1, GPAA1, MRPS12, SERPINA10, HEPACAM2, BICDL1, MYL6B, SHB, HOXC10, WFDC2, ASGR1, AACS, HEY1, ADAMTSL2, FFAR2, KIF1A, SLC18A1, TEAD4, MAP4K2, ATP6V0B, RPS6KL1, MYOC, GP1BB, TESC, SYP, AP3B2, MTSS1, PDRG1, MMP11, CTU1, KIFC2, INCENP, SLC29A4, VWA1, KIF2C, TIMM10, GDPD5, ASCL1, CENPA, CRIP1, SLC7A1, ASCL2, RELB, RECQL4, CST3, CST1, NPW, MTHFD1L, UCP2, RAD54L, PRAME, DTD1, GINS2, LRRC24, NPM2, ZNF580, CDKN2A, GDF15, IL34, GAD1, TMEM176B, TMEM176A, STAC3, NPM3, AGT, GUCA2A, MAD2L2, HEYL, FABP4, SMOC1, SHKBP1, TACC3, RIMBP2, CDK5R2, FOXA2, EXOC3L1, SCARB1, RTKN, CRTC1, GALNT18, REG1A, OGDHL, COX6A1, IPO4, TMEM145, PTTG1, SALL4, PHLDA2, TXNL4A, TNFRSF4, PPFIA3, SLC39A3, CHRNB2, SPINK4, KCNH2, TPRA1, DGAT2, KCNH6, LIG1, KMT5C, ATP11A, RUNDC3A, FOXP3, SRCIN1, PGF, TRAIP, CDC25B, LOC102724428, CCNE1, TIMELESS, DPEP1, ANAPC5, TSNARE1, RPP25, ABCG1, LY6E, GNAZ, REG3A, SHC2, RNASEH2A, DPT, FOXO6, SLC38A11, CACNA1D, SLC1A5, CPLX2, SEZ6L2, PKMYT1, CPLX1, TIMM50, NME1-NME2, VTN, ADGRG1, PCSK1N, SOCS1, ADGRG5, RHPN1, H1FX, BMF, CYC1, SAC3D1, UBE2C, SLC52A2, CBX2, CYBRD1, GGH, SMAD9, SYT7, DRAP1, SDK1, AMACR, G6PC3, KIF18B, UBE2S, CDK4, MAFG, MAP3K10, SP5, MELTF, FGFR4, ULBP2, RNF187, SNAP25, DAGLA, DYRK2, PYCRL, PODXL2, ZC3H3, CELF4, RND1, DUSP10, SPR, DACT2, MYBL2, CA8, GRASP, TGIF1, PLEKHG4, EMX1, ABCC8, MMP1, H2AFX, APOA2, C10ORF11, KCNAB2, ADRA2C, ETV4, VGF, CHI3L1, TNFRSF25, PFDN2, MCM2, CEBPB, NAXE, UHRF1, RGS16, LRP5, ATP1A3, RRBP1, DBF4B, LAGE3, CLN6, SRM, AURKA, DLL3, DLL4, P2RY6, TTR, PARD6A, PUF60, GNG4, RAB26, POLD1, NT5DC2, E2F1, POLR2I, SLC25A22, SEC11C, CTHRC1, HES4, OR51E1, CMTM8, PPP1R14B, PPP1R14C, KCNJ11, CADPS, ARID3A, CCK, FAM155B, NFASC, SLC25A39, PRC1, RAB15, TSPAN18, MFAP2, APOC1, NEURL1, INSM1, KCNK3 | 349 | 15558 | 16792 | 1.03602 | 1 | 0.249765 | 0.245394 |
| GOTERM_BP_ALL | GO:0000082~G1/S transition of mitotic cell cycle | 12 | 2.803738 | 0.007193 | CDT1, PLK3, CCNE1, CDKN2A, PRKDC, CDK4, CDCA5, E2F1, ARID3A, PKMYT1, AURKA, MCM2 | 349 | 224 | 16792 | 2.577569 | 1 | 0.252247 | 0.247833 |
| GOTERM_BP_ALL | GO:0060429~epithelium development | 35 | 8.17757 | 0.007441 | REG3A, MESP1, CEBPB, PRKDC, LRP5, SCX, ASCL1, AQP3, DLL3, NME1-NME2, DLL4, CST3, PARD6A, HEY1, MTHFD1L, SOX18, SALL4, ADAMTSL2, DACT2, CTHRC1, EMX1, SMAD9, MYO7A, ETV4, PGF, AGT, MTSS1, HEYL, CLDN3, RFX6, MAFG, TIMELESS, ORAI1, HOXB2, INSM1 | 349 | 1060 | 16792 | 1.58869 | 1 | 0.258991 | 0.254459 |
| GOTERM_BP_ALL | GO:0045444~fat cell differentiation | 11 | 2.570093 | 0.00808 | MMP11, CEBPB, TPH1, DUSP10, FABP4, SOCS1, LRP5, E2F1, TRIB3, FFAR2, AACS | 349 | 197 | 16792 | 2.686603 | 1 | 0.279126 | 0.274242 |
| GOTERM_BP_ALL | GO:0042391~regulation of membrane potential | 16 | 3.738318 | 0.008355 | KCNH2, CHRNB2, KCNH6, MYOC, KCNJ11, CDKN2A, CRTC1, CELF4, ATP1A3, CACNA1D, CCK, MAPK8IP2, AGT, UCP2, KCNK3, STX1A | 349 | 360 | 16792 | 2.138427 | 1 | 0.282465 | 0.277522 |
| GOTERM_BP_ALL | GO:0051234~establishment of localization | 121 | 28.27103 | 0.008392 | SPON2, SCARB1, EXOC3L1, CPNE7, SERPINE1, TONSL, COX6A1, AQP3, IPO4, SCAMP5, RINL, RPS19, TNFRSF4, PPFIA3, SLC39A3, CHRNB2, KCNH2, UNC13A, KCNH6, NUP210, SERPINF2, MYO7A, BHLHA15, ATP11A, MAPK8IP2, SRCIN1, MFSD12, FOXP3, MAPK8IP1, LOC102724428, RFX6, ORAI1, SIK1, TRIB3, TSNARE1, ABCG1, STX1A, TTYH3, TMEM63C, CDCA5, C2CD4A, CACNA1D, C2CD4B, SLC38A11, C2CD4C, SLC1A5, CPLX2, CPLX1, TIMM50, C2, VTN, TRPM2, SOCS1, BMF, CYC1, PLK3, PTPRN2, SLC52A2, STARD10, ASPSCR1, ABCA7, NR0B2, SYT7, PARP10, G6PC3, FXYD2, MELTF, SNAP25, SERPINA1, NXT1, DYRK2, ZC3H3, BICDL1, ASGR1, AACS, FFAR2, KIF1A, SLC18A1, MAP4K2, ATP6V0B, ABCC8, TESC, APOA2, KCNAB2, SYP, ADRA2C, AP3B2, VGF, CHI3L1, SLC29A4, KIF2C, TMEM9, LRP5, ATP1A3, RRBP1, TIMM10, CENPA, SLC7A1, P2RY6, TTR, RAB26, UCP2, E2F1, SLC25A22, KCNJ11, CDKN2A, GAD1, CADPS, CCK, AGT, FAM155B, NFASC, FABP4, SLC25A39, SLCO3A1, RAB15, APOC1, NEURL1, KCNK3, CDK5R2, FOXA2 | 349 | 4790 | 16792 | 1.215421 | 1 | 0.282465 | 0.277522 |
| GOTERM_BP_ALL | GO:0032792~negative regulation of CREB transcription factor activity | 3 | 0.700935 | 0.008395 | LOC102724428, SIK1, FOXP3 | 349 | 7 | 16792 | 20.62055 | 1 | 0.282465 | 0.277522 |
| GOTERM_BP_ALL | GO:0033344~cholesterol efflux | 5 | 1.168224 | 0.008421 | SCARB1, APOC1, APOA2, ABCA7, ABCG1 | 349 | 39 | 16792 | 6.16854 | 1 | 0.282465 | 0.277522 |
| GOTERM_BP_ALL | GO:1903532~positive regulation of secretion by cell | 15 | 3.504673 | 0.008537 | CHRNB2, UNC13A, CCK, NR0B2, AACS, SYT7, AGT, SCAMP5, TRPM2, RFX6, RAB15, FFAR2, TNFRSF4, CDK5R2, STX1A | 349 | 327 | 16792 | 2.207092 | 1 | 0.284298 | 0.279324 |
| GOTERM_BP_ALL | GO:0032269~negative regulation of cellular protein metabolic process | 33 | 7.71028 | 0.008777 | SERPINA1, PRKDC, SERPINA10, SERPINE1, LRP5, CELF4, SHB, WFDC2, CDC20, CST3, VTN, PCSK1N, CST1, SOCS1, DUSP10, PARD6A, PTTG1, SPINK4, CDKN2A, UBE2C, TESC, SERPINF2, SRCIN1, FOXP3, AGT, MAPK8IP1, PARP10, MAD2L2, FABP4, DPEP1, TRIB3, ANAPC5, INSM1 | 349 | 992 | 16792 | 1.600587 | 1 | 0.290189 | 0.285111 |
| GOTERM_BP_ALL | GO:0003002~regionalization | 15 | 3.504673 | 0.008973 | EMX1, MESP1, PRKDC, LRP5, ASCL1, HOXC10, AURKA, DLL3, DLL4, ADGRG1, HOXB9, HOXB2, HOXC9, FOXA2, HOXC6 | 349 | 329 | 16792 | 2.193675 | 1 | 0.294592 | 0.289438 |
| GOTERM_BP_ALL | GO:0030199~collagen fibril organization | 5 | 1.168224 | 0.009207 | MMP11, ADAMTS14, SERPINF2, SCX, DPT | 349 | 40 | 16792 | 6.014327 | 1 | 0.30013 | 0.294878 |
| GOTERM_BP_ALL | GO:0048519~negative regulation of biological process | 117 | 27.33645 | 0.009342 | ZNF296, RTKN, SERPINE1, TONSL, SCX, STMN3, SCAMP5, CDC20, PTTG1, RPS19, SOX18, SALL4, TNFRSF4, IER3, CHRNB2, SPINK4, KCNH2, TPRA1, DGAT2, NUP210, SERPINF2, BHLHA15, SRCIN1, FOXP3, MAPK8IP1, TRAIP, LOC102724428, EID2, TIMELESS, DPEP1, SIK1, TRIB3, ANAPC5, ABCG1, REG3A, TPH1, PRKDC, PPP1R35, DPT, PKMYT1, NME1-NME2, VTN, TRPM2, ADGRG1, PCSK1N, SOCS1, PCBP3, DHX34, HOXC6, PLK3, PTPRN2, UBE2C, CBX2, ABCA7, NR0B2, DRAP1, PARP10, CDK4, MAP3K10, MELTF, MESP1, SERPINA1, DYRK2, SERPINA10, CELF4, SHB, WFDC2, RND1, DUSP10, HEY1, ADAMTSL2, DACT2, TGIF1, MYOC, ABCC8, TESC, H2AFX, APOA2, ADRA2C, ETV4, MTSS1, MMP11, CEBPB, UHRF1, RGS16, LRP5, GDPD5, ASCL1, ASCL2, RELB, AURKA, DLL3, DLL4, CST3, CST1, PARD6A, GNG4, UCP2, E2F1, PRAME, POLR2I, CTHRC1, KCNJ11, CDKN2A, TMEM176B, TMEM176A, ARID3A, CCK, AGT, MAD2L2, HEYL, FABP4, APOC1, NEURL1, RIMBP2, INSM1, FOXA2 | 349 | 4623 | 16792 | 1.217696 | 1 | 0.3024 | 0.297109 |
| GOTERM_BP_ALL | GO:0000070~mitotic sister chromatid segregation | 9 | 2.102804 | 0.009561 | MAD2L2, KIF18B, UBE2C, PRC1, CDCA5, TACC3, KIF2C, CENPA, NCAPH | 349 | 142 | 16792 | 3.049518 | 1 | 0.307354 | 0.301976 |
| GOTERM_BP_ALL | GO:0007623~circadian rhythm | 10 | 2.336449 | 0.009785 | CHRNB2, CST3, LOC102724428, TPH1, CDK4, CRTC1, SERPINE1, TIMELESS, SIK1, RELB | 349 | 172 | 16792 | 2.797361 | 1 | 0.312366 | 0.3069 |
| GOTERM_BP_ALL | GO:0008203~cholesterol metabolic process | 8 | 1.869159 | 0.010051 | SCARB1, LIPE, APOC1, APOA2, LRP5, NR0B2, ABCG1, CLN6 | 349 | 115 | 16792 | 3.347104 | 1 | 0.318656 | 0.31308 |
| GOTERM_BP_ALL | GO:0008283~cell proliferation | 54 | 12.61682 | 0.010239 | SCARB1, DAGLA, REG1B, REG1A, SCX, SHB, HOXC10, CDC20, SOX18, SALL4, TNFRSF4, CHRNB2, EMX1, TESC, SERPINF2, ETV4, FOXP3, MTSS1, PGF, TRAIP, CDC25B, KIF2C, REG3A, CEBPB, SHMT2, UHRF1, PRKDC, LRP5, DPT, DBF4B, ASCL1, ASCL2, CRIP1, NME1-NME2, DLL4, CST3, ADGRG1, E2F1, PRAME, CTHRC1, ZNF580, CDKN2A, IL34, CCK, AGT, PARP10, ARTN, PRC1, CDK4, NEURL1, TACC3, INSM1, FGFR4, RNF187 | 349 | 1861 | 16792 | 1.396125 | 1 | 0.319188 | 0.313603 |
| GOTERM_BP_ALL | GO:0042593~glucose homeostasis | 11 | 2.570093 | 0.010274 | NME1-NME2, PTPRN2, VGF, RFX6, KCNJ11, UCP2, ASPSCR1, FFAR2, BHLHA15, FGFR4, AACS | 349 | 204 | 16792 | 2.594415 | 1 | 0.319188 | 0.313603 |
| GOTERM_BP_ALL | GO:0033500~carbohydrate homeostasis | 11 | 2.570093 | 0.010274 | NME1-NME2, PTPRN2, VGF, RFX6, KCNJ11, UCP2, ASPSCR1, FFAR2, BHLHA15, FGFR4, AACS | 349 | 204 | 16792 | 2.594415 | 1 | 0.319188 | 0.313603 |
| GOTERM_BP_ALL | GO:1901987~regulation of cell cycle phase transition | 15 | 3.504673 | 0.010726 | PLK3, TPRA1, NPM2, CDKN2A, PRKDC, UBE2C, CDCA5, ARID3A, DBF4B, AURKA, MAD2L2, CDC20, CDK4, E2F1, ANAPC5 | 349 | 336 | 16792 | 2.147974 | 1 | 0.329724 | 0.323955 |
| GOTERM_BP_ALL | GO:0006261~DNA-dependent DNA replication | 9 | 2.102804 | 0.010766 | CDT1, GINS2, RNASEH2A, LIG1, CCNE1, POLD1, TONSL, DBF4B, MCM2 | 349 | 145 | 16792 | 2.986424 | 1 | 0.329724 | 0.323955 |
| GOTERM_BP_ALL | GO:0014047~glutamate secretion | 5 | 1.168224 | 0.010919 | SNAP25, CCK, CPLX1, STX1A, PPFIA3 | 349 | 42 | 16792 | 5.72793 | 1 | 0.329724 | 0.323955 |
| GOTERM_BP_ALL | GO:0006302~double-strand break repair | 11 | 2.570093 | 0.010942 | RECQL4, MAD2L2, GINS2, RTEL1-TNFRSF6B, LIG1, PRKDC, CDCA5, H2AFX, TIMELESS, TONSL, RAD54L | 349 | 206 | 16792 | 2.569227 | 1 | 0.329724 | 0.323955 |
| GOTERM_BP_ALL | GO:0010604~positive regulation of macromolecule metabolic process | 77 | 17.99065 | 0.010972 | MESP1, DYRK2, CRTC1, SERPINE1, CELF4, SCX, CDC20, HEY1, SOX18, SALL4, ZXDC, MYBL2, TNFRSF4, MAP4K2, TESC, H2AFX, SERPINF2, APOA2, SOX12, BHLHA15, ADRA2C, MAPK8IP2, ETV4, SRCIN1, FOXP3, CDC25B, HOXB9, RFX6, CCNE1, TIMELESS, CHI3L1, TNFRSF25, TRIB3, ANAPC5, CEBPB, SHC2, BEX1, UHRF1, PRKDC, LRP5, DBF4B, ASCL1, CLN6, AURKA, RELB, NME1-NME2, DLL4, CST3, VTN, SOCS1, E2F1, POLR2I, PLK3, NPM2, ZNF580, CDKN2A, GDF15, UBE2C, IL34, SMAD9, ARID3A, ABCA7, CCK, NR0B2, AGT, MAD2L2, HEYL, UBE2S, CDK4, MAFG, NEURL1, MAP3K10, MELTF, FGFR4, CDK5R2, RNF187, FOXA2 | 349 | 2856 | 16792 | 1.297208 | 1 | 0.329724 | 0.323955 |
| GOTERM_BP_ALL | GO:0016081~synaptic vesicle docking | 3 | 0.700935 | 0.011041 | SNAP25, UNC13A, STX1A | 349 | 8 | 16792 | 18.04298 | 1 | 0.329724 | 0.323955 |
| GOTERM_BP_ALL | GO:0051248~negative regulation of protein metabolic process | 34 | 7.943925 | 0.011114 | SERPINA1, PRKDC, SERPINA10, SERPINE1, LRP5, CELF4, SHB, WFDC2, CDC20, CST3, VTN, PCSK1N, CST1, SOCS1, DUSP10, PARD6A, PTTG1, SPINK4, CDKN2A, UBE2C, TESC, SERPINF2, ABCA7, SRCIN1, FOXP3, AGT, MAPK8IP1, PARP10, MAD2L2, FABP4, DPEP1, TRIB3, ANAPC5, INSM1 | 349 | 1049 | 16792 | 1.559482 | 1 | 0.329788 | 0.324018 |
| GOTERM_BP_ALL | GO:1903307~positive regulation of regulated secretory pathway | 5 | 1.168224 | 0.011846 | SCAMP5, RAB15, STX1A, SYT7, CDK5R2 | 349 | 43 | 16792 | 5.594722 | 1 | 0.349153 | 0.343043 |
| GOTERM_BP_ALL | GO:0070201~regulation of establishment of protein localization | 28 | 6.542056 | 0.011918 | SNAP25, DYRK2, LRP5, TONSL, CACNA1D, AACS, SCAMP5, TRPM2, SOCS1, UCP2, E2F1, FFAR2, BMF, TNFRSF4, PLK3, KCNJ11, CDKN2A, ABCC8, APOA2, ADRA2C, SRCIN1, NR0B2, FOXP3, SYT7, PARP10, RFX6, STX1A, FOXA2 | 349 | 820 | 16792 | 1.642938 | 1 | 0.349153 | 0.343043 |
| GOTERM_BP_ALL | GO:1903305~regulation of regulated secretory pathway | 8 | 1.869159 | 0.012512 | SCAMP5, RAB15, C2CD4A, C2CD4B, C2CD4C, STX1A, SYT7, CDK5R2 | 349 | 120 | 16792 | 3.207641 | 1 | 0.363965 | 0.357596 |
| GOTERM_BP_ALL | GO:0051223~regulation of protein transport | 26 | 6.074766 | 0.012679 | SNAP25, DYRK2, LRP5, TONSL, CACNA1D, AACS, SCAMP5, TRPM2, SOCS1, UCP2, FFAR2, TNFRSF4, PLK3, KCNJ11, CDKN2A, ABCC8, APOA2, ADRA2C, SRCIN1, NR0B2, FOXP3, SYT7, PARP10, RFX6, STX1A, FOXA2 | 349 | 746 | 16792 | 1.676917 | 1 | 0.363965 | 0.357596 |
| GOTERM_BP_ALL | GO:0030162~regulation of proteolysis | 25 | 5.841121 | 0.012719 | SERPINA1, SERPINA10, SERPINE1, CLN6, WFDC2, AURKA, C2, CDC20, CST3, VTN, PCSK1N, CST1, PTTG1, SPINK4, PLK3, CDKN2A, UBE2C, SERPINF2, CCK, AGT, DPEP1, TRIB3, ANAPC5, MELTF, FGFR4 | 349 | 708 | 16792 | 1.698962 | 1 | 0.363965 | 0.357596 |
| GOTERM_BP_ALL | GO:0008202~steroid metabolic process | 13 | 3.037383 | 0.012758 | SCARB1, DGAT2, LRP5, APOA2, NR0B2, CLN6, AGT, SULT2B1, LIPE, AMACR, APOC1, FGFR4, ABCG1 | 349 | 276 | 16792 | 2.266268 | 1 | 0.363965 | 0.357596 |
| GOTERM_BP_ALL | GO:0050432~catecholamine secretion | 5 | 1.168224 | 0.012823 | CHRNB2, CADPS, ADRA2C, STX1A, AGT | 349 | 44 | 16792 | 5.46757 | 1 | 0.363965 | 0.357596 |
| GOTERM_BP_ALL | GO:0045940~positive regulation of steroid metabolic process | 4 | 0.934579 | 0.012935 | APOC1, APOA2, ABCG1, AGT | 349 | 24 | 16792 | 8.019102 | 1 | 0.363965 | 0.357596 |
| GOTERM_BP_ALL | GO:0044843~cell cycle G1/S phase transition | 12 | 2.803738 | 0.013026 | CDT1, PLK3, CCNE1, CDKN2A, PRKDC, CDK4, CDCA5, E2F1, ARID3A, PKMYT1, AURKA, MCM2 | 349 | 244 | 16792 | 2.366292 | 1 | 0.363965 | 0.357596 |
| GOTERM_BP_ALL | GO:1902652~secondary alcohol metabolic process | 8 | 1.869159 | 0.013052 | SCARB1, LIPE, APOC1, APOA2, LRP5, NR0B2, ABCG1, CLN6 | 349 | 121 | 16792 | 3.181131 | 1 | 0.363965 | 0.357596 |
| GOTERM_BP_ALL | GO:0055092~sterol homeostasis | 6 | 1.401869 | 0.013346 | SCARB1, FABP4, DGAT2, APOA2, LRP5, ABCG1 | 349 | 68 | 16792 | 4.245407 | 1 | 0.36774 | 0.361305 |
| GOTERM_BP_ALL | GO:0042632~cholesterol homeostasis | 6 | 1.401869 | 0.013346 | SCARB1, FABP4, DGAT2, APOA2, LRP5, ABCG1 | 349 | 68 | 16792 | 4.245407 | 1 | 0.36774 | 0.361305 |
| GOTERM_BP_ALL | GO:0048511~rhythmic process | 14 | 3.271028 | 0.013561 | CHRNB2, CEBPB, TPH1, CRTC1, PRKDC, SERPINE1, RELB, CST3, LOC102724428, TRPM2, VGF, CDK4, TIMELESS, SIK1 | 349 | 312 | 16792 | 2.158989 | 1 | 0.371326 | 0.364829 |
| GOTERM_BP_ALL | GO:0065008~regulation of biological quality | 92 | 21.49533 | 0.013637 | PCSK1, SCARB1, EXOC3L1, SNAP25, SERPINA1, DAGLA, GPAA1, CRTC1, SERPINE1, SERPINA10, CELF4, TONSL, SCX, AQP3, AACS, CDC20, RPS19, FFAR2, PPFIA3, EPHB3, SLC39A3, KCNH2, CHRNB2, EMX1, ATP6V0B, DGAT2, UNC13A, KCNH6, LIG1, MYOC, ABCC8, GP1BB, TESC, SERPINF2, APOA2, BHLHA15, SYP, ATP11A, ADRA2C, MAPK8IP2, FOXP3, VGF, RFX6, ABCG1, STX1A, PRKDC, LRP5, C2CD4A, ATP1A3, CACNA1D, C2CD4B, C2CD4C, CPLX2, CPLX1, CLN6, AURKA, NME1-NME2, VTN, TRPM2, PCSK1N, TTR, POLD1, UCP2, E2F1, BMF, DTD1, RTEL1-TNFRSF6B, LRRC24, PTPRN2, KCNJ11, CDKN2A, UBE2C, GAD1, CADPS, ASPSCR1, CYBRD1, ABCA7, CCK, NR0B2, AGT, SYT7, FAM155B, FABP4, UBE2S, CDK4, MAFG, PAH, NEURL1, MELTF, FGFR4, KCNK3, FOXA2 | 349 | 3548 | 16792 | 1.247617 | 1 | 0.371326 | 0.364829 |
| GOTERM_BP_ALL | GO:0010873~positive regulation of cholesterol esterification | 3 | 0.700935 | 0.014002 | APOC1, APOA2, AGT | 349 | 9 | 16792 | 16.0382 | 1 | 0.37904 | 0.372407 |
| GOTERM_BP_ALL | GO:0042325~regulation of phosphorylation | 42 | 9.813084 | 0.014159 | SHC2, PRKDC, LRP5, DBF4B, PKMYT1, SHB, VTN, SOCS1, DUSP10, PARD6A, TNFRSF4, MAP4K2, PPP1R14B, PPP1R14C, MYOC, CDKN2A, GDF15, UBE2C, IL34, TESC, SERPINF2, ABCA7, CCK, ADRA2C, MAPK8IP2, SRCIN1, AGT, MAPK8IP1, CDC25B, MAD2L2, FABP4, CCNE1, CDK4, NEURL1, MAP3K10, CHI3L1, TNFRSF25, TRIB3, INSM1, FGFR4, CDK5R2, FOXA2 | 349 | 1393 | 16792 | 1.450692 | 1 | 0.381052 | 0.374385 |
| GOTERM_BP_ALL | GO:0045596~negative regulation of cell differentiation | 23 | 5.373832 | 0.014299 | REG3A, MESP1, CDKN2A, TMEM176B, TMEM176A, LRP5, BHLHA15, ASCL1, SHB, ASCL2, FOXP3, DLL3, NME1-NME2, MAD2L2, MMP11, ADGRG1, DUSP10, E2F1, TRIB3, PRAME, MELTF, ABCG1, FOXA2 | 349 | 640 | 16792 | 1.729119 | 1 | 0.382598 | 0.375903 |
| GOTERM_BP_ALL | GO:0048469~cell maturation | 9 | 2.102804 | 0.014527 | NFASC, MYOC, SOX18, BHLHA15, SHB, ASCL1, CDC25B, AURKA, RND1 | 349 | 153 | 16792 | 2.830271 | 1 | 0.386459 | 0.379697 |
| GOTERM_BP_ALL | GO:0051656~establishment of organelle localization | 17 | 3.971963 | 0.014649 | MAP4K2, SNAP25, SERPINA1, UNC13A, CDCA5, CADPS, C2CD4A, C2CD4B, C2CD4C, MYO7A, CPLX2, CENPA, AP3B2, CPLX1, SYT7, KIF2C, STX1A | 349 | 420 | 16792 | 1.947496 | 1 | 0.38748 | 0.3807 |
| GOTERM_BP_ALL | GO:0045931~positive regulation of mitotic cell cycle | 8 | 1.869159 | 0.014773 | UBE2C, CDK4, CDCA5, LRP5, DBF4B, SHB, CDC25B, AURKA | 349 | 124 | 16792 | 3.104169 | 1 | 0.387906 | 0.381119 |
| GOTERM_BP_ALL | GO:0021517~ventral spinal cord development | 5 | 1.168224 | 0.014927 | DLL4, GDPD5, ASCL1, MNX1, HOXC10 | 349 | 46 | 16792 | 5.229849 | 1 | 0.387906 | 0.381119 |
| GOTERM_BP_ALL | GO:0008593~regulation of Notch signaling pathway | 6 | 1.401869 | 0.014989 | DLL4, MESP1, HEY1, NEURL1, GDPD5, ASCL1 | 349 | 70 | 16792 | 4.12411 | 1 | 0.387906 | 0.381119 |
| GOTERM_BP_ALL | GO:0048699~generation of neurons | 41 | 9.579439 | 0.015 | SNAP25, SPON2, CEBPB, DAGLA, CRTC1, BICDL1, FOXO6, STMN3, GDPD5, ASCL1, ASCL2, HOXC10, AURKA, RND1, DLL3, NME1-NME2, CDC20, DLL4, ADGRG1, DUSP10, MNX1, EPHB3, CTHRC1, CHRNB2, EMX1, UNC13A, MYOC, CCK, MYO7A, ADRA2C, MAPK8IP2, ETV4, SRCIN1, HEYL, SDK1, NFASC, ARTN, NEURL1, INSM1, CDK5R2, FOXA2 | 349 | 1357 | 16792 | 1.453721 | 1 | 0.387906 | 0.381119 |
| GOTERM_BP_ALL | GO:0045861~negative regulation of proteolysis | 14 | 3.271028 | 0.015288 | SPINK4, SERPINA1, CDKN2A, SERPINF2, SERPINA10, SERPINE1, WFDC2, AGT, CST3, VTN, PCSK1N, CST1, PTTG1, DPEP1 | 349 | 317 | 16792 | 2.124936 | 1 | 0.39316 | 0.386281 |
| GOTERM_BP_ALL | GO:0019220~regulation of phosphate metabolic process | 47 | 10.98131 | 0.015735 | SHC2, PRKDC, PPP1R35, LRP5, DBF4B, PKMYT1, SHB, NME1-NME2, VTN, SOCS1, DUSP10, PARD6A, TNFRSF4, MAP4K2, PPP1R14B, PPP1R14C, MYOC, CDKN2A, GDF15, UBE2C, IL34, TESC, SERPINF2, ABCA7, CCK, ADRA2C, MAPK8IP2, RUNDC3A, SRCIN1, GUCA2A, AGT, MAPK8IP1, CDC25B, MAD2L2, FABP4, CCNE1, CDK4, NEURL1, RIMBP2, MAP3K10, CHI3L1, TNFRSF25, TRIB3, INSM1, FGFR4, CDK5R2, FOXA2 | 349 | 1610 | 16792 | 1.404588 | 1 | 0.401564 | 0.394537 |
| GOTERM_BP_ALL | GO:0051174~regulation of phosphorus metabolic process | 47 | 10.98131 | 0.015788 | SHC2, PRKDC, PPP1R35, LRP5, DBF4B, PKMYT1, SHB, NME1-NME2, VTN, SOCS1, DUSP10, PARD6A, TNFRSF4, MAP4K2, PPP1R14B, PPP1R14C, MYOC, CDKN2A, GDF15, UBE2C, IL34, TESC, SERPINF2, ABCA7, CCK, ADRA2C, MAPK8IP2, RUNDC3A, SRCIN1, GUCA2A, AGT, MAPK8IP1, CDC25B, MAD2L2, FABP4, CCNE1, CDK4, NEURL1, RIMBP2, MAP3K10, CHI3L1, TNFRSF25, TRIB3, INSM1, FGFR4, CDK5R2, FOXA2 | 349 | 1611 | 16792 | 1.403716 | 1 | 0.401564 | 0.394537 |
| GOTERM_BP_ALL | GO:0006271~DNA strand elongation involved in DNA replication | 4 | 0.934579 | 0.016119 | GINS2, RNASEH2A, LIG1, POLD1 | 349 | 26 | 16792 | 7.402248 | 1 | 0.407737 | 0.400602 |
| GOTERM_BP_ALL | GO:0051047~positive regulation of secretion | 15 | 3.504673 | 0.016488 | CHRNB2, UNC13A, CCK, NR0B2, AACS, SYT7, AGT, SCAMP5, TRPM2, RFX6, RAB15, FFAR2, TNFRSF4, CDK5R2, STX1A | 349 | 355 | 16792 | 2.033012 | 1 | 0.414788 | 0.40753 |
| GOTERM_BP_ALL | GO:0048523~negative regulation of cellular process | 108 | 25.23364 | 0.016748 | ZNF296, SERPINE1, TONSL, SCX, STMN3, SCAMP5, CDC20, PTTG1, SOX18, SALL4, TNFRSF4, IER3, SPINK4, KCNH2, TPRA1, DGAT2, SERPINF2, BHLHA15, SRCIN1, FOXP3, MAPK8IP1, TRAIP, LOC102724428, EID2, TIMELESS, DPEP1, SIK1, TRIB3, ANAPC5, ABCG1, REG3A, PRKDC, PPP1R35, DPT, PKMYT1, NME1-NME2, VTN, TRPM2, ADGRG1, PCSK1N, SOCS1, PCBP3, DHX34, HOXC6, PLK3, UBE2C, CBX2, ABCA7, NR0B2, DRAP1, PARP10, CDK4, MAP3K10, MELTF, MESP1, SERPINA1, SERPINA10, CELF4, SHB, WFDC2, RND1, DUSP10, HEY1, ADAMTSL2, DACT2, TGIF1, MYOC, ABCC8, TESC, H2AFX, APOA2, ADRA2C, ETV4, MTSS1, MMP11, CEBPB, UHRF1, RGS16, LRP5, GDPD5, ASCL1, ASCL2, RELB, AURKA, DLL3, DLL4, CST3, CST1, PARD6A, GNG4, UCP2, E2F1, PRAME, CTHRC1, KCNJ11, CDKN2A, TMEM176B, TMEM176A, ARID3A, AGT, MAD2L2, HEYL, FABP4, APOC1, NEURL1, RIMBP2, INSM1, FOXA2 | 349 | 4300 | 16792 | 1.20846 | 1 | 0.419066 | 0.411733 |
| GOTERM_BP_ALL | GO:0032880~regulation of protein localization | 31 | 7.242991 | 0.016879 | SNAP25, DYRK2, CDCA5, LRP5, TONSL, CACNA1D, AACS, SCAMP5, TRPM2, SOCS1, PARD6A, UCP2, E2F1, FFAR2, BMF, TNFRSF4, PLK3, KCNJ11, CDKN2A, ABCC8, APOA2, KCNAB2, ADRA2C, SRCIN1, NR0B2, FOXP3, SYT7, PARP10, RFX6, STX1A, FOXA2 | 349 | 962 | 16792 | 1.550471 | 1 | 0.419537 | 0.412196 |
| GOTERM_BP_ALL | GO:0051348~negative regulation of transferase activity | 15 | 3.504673 | 0.017181 | CDKN2A, UBE2C, TESC, LRP5, SHB, SRCIN1, MAPK8IP1, MAD2L2, CDC20, SOCS1, DUSP10, FABP4, TRIB3, ANAPC5, FOXA2 | 349 | 357 | 16792 | 2.021622 | 1 | 0.419537 | 0.412196 |
| GOTERM_BP_ALL | GO:0033673~negative regulation of kinase activity | 12 | 2.803738 | 0.017199 | DUSP10, FABP4, SOCS1, CDKN2A, UBE2C, TESC, LRP5, TRIB3, SHB, SRCIN1, FOXA2, MAPK8IP1 | 349 | 254 | 16792 | 2.273131 | 1 | 0.419537 | 0.412196 |
| GOTERM_BP_ALL | GO:0048168~regulation of neuronal synaptic plasticity | 5 | 1.168224 | 0.017238 | UNC13A, NEURL1, SYP, AGT, PPFIA3 | 349 | 48 | 16792 | 5.011939 | 1 | 0.419537 | 0.412196 |
| GOTERM_BP_ALL | GO:0010755~regulation of plasminogen activation | 3 | 0.700935 | 0.017265 | SERPINF2, SERPINE1, MELTF | 349 | 10 | 16792 | 14.43438 | 1 | 0.419537 | 0.412196 |
| GOTERM_BP_ALL | GO:0046890~regulation of lipid biosynthetic process | 8 | 1.869159 | 0.017311 | LOC102724428, SCARB1, DGAT2, APOC1, TRIB3, SIK1, FGFR4, ABCG1 | 349 | 128 | 16792 | 3.007163 | 1 | 0.419537 | 0.412196 |
| GOTERM_BP_ALL | GO:0030301~cholesterol transport | 6 | 1.401869 | 0.017699 | SCARB1, APOC1, APOA2, ABCA7, ABCG1, SYT7 | 349 | 73 | 16792 | 3.954626 | 1 | 0.426712 | 0.419245 |
| GOTERM_BP_ALL | GO:0016125~sterol metabolic process | 8 | 1.869159 | 0.017991 | SCARB1, LIPE, APOC1, APOA2, LRP5, NR0B2, ABCG1, CLN6 | 349 | 129 | 16792 | 2.983852 | 1 | 0.430428 | 0.422896 |
| GOTERM_BP_ALL | GO:0060284~regulation of cell development | 28 | 6.542056 | 0.018216 | SNAP25, CRTC1, FOXO6, GDPD5, ASCL1, SHB, ASCL2, AURKA, DLL3, NME1-NME2, CDC20, DLL4, ADGRG1, DUSP10, RPS19, EPHB3, CHRNB2, EMX1, UNC13A, MYOC, ADRA2C, SRCIN1, MAD2L2, HEYL, SDK1, NEURL1, MELTF, FOXA2 | 349 | 851 | 16792 | 1.58309 | 1 | 0.430428 | 0.422896 |
| GOTERM_BP_ALL | GO:0071702~organic substance transport | 71 | 16.58879 | 0.01825 | SCARB1, EXOC3L1, SNAP25, NXT1, DYRK2, ZC3H3, TONSL, AQP3, AACS, IPO4, SCAMP5, RINL, RPS19, FFAR2, SLC18A1, TNFRSF4, PPFIA3, CHRNB2, ATP6V0B, NUP210, ABCC8, TESC, APOA2, MYO7A, ATP11A, ADRA2C, AP3B2, SRCIN1, FOXP3, VGF, RFX6, CHI3L1, SLC29A4, TSNARE1, ABCG1, STX1A, LRP5, CACNA1D, SLC38A11, RRBP1, TIMM10, SLC1A5, SLC7A1, CPLX1, TIMM50, TRPM2, SOCS1, TTR, RAB26, UCP2, SLC25A22, PLK3, PTPRN2, KCNJ11, CDKN2A, SLC52A2, CADPS, STARD10, ASPSCR1, ABCA7, CCK, NR0B2, AGT, SYT7, PARP10, NFASC, G6PC3, SLCO3A1, RAB15, APOC1, FOXA2 | 349 | 2657 | 16792 | 1.285712 | 1 | 0.430428 | 0.422896 |
| GOTERM_BP_ALL | GO:0044708~single-organism behavior | 16 | 3.738318 | 0.018261 | CHRNB2, SNAP25, CEBPB, CRTC1, FOXO6, ATP1A3, ABCA7, CCK, SEZ6L2, TPGS1, MAPK8IP2, CST3, SDK1, MAFG, VWA1, FOXA2 | 349 | 395 | 16792 | 1.948946 | 1 | 0.430428 | 0.422896 |
| GOTERM_BP_ALL | GO:0006006~glucose metabolic process | 10 | 2.336449 | 0.018318 | LOC102724428, SDS, G6PC3, DGAT2, DYRK2, KCNJ11, LRP5, SIK1, PHLDA2, FOXA2 | 349 | 191 | 16792 | 2.51909 | 1 | 0.430428 | 0.422896 |
| GOTERM_BP_ALL | GO:0000819~sister chromatid segregation | 11 | 2.570093 | 0.018671 | MAD2L2, CDC20, KIF18B, INCENP, UBE2C, PRC1, CDCA5, TACC3, KIF2C, CENPA, NCAPH | 349 | 224 | 16792 | 2.362771 | 1 | 0.436505 | 0.428867 |
| GOTERM_BP_ALL | GO:0071840~cellular component organization or biogenesis | 150 | 35.04673 | 0.018898 | SPON2, SCARB1, CRTC1, HDAC11, SERPINE1, PPAN, SCX, STMN3, TPGS1, AQP3, IPO4, RRP9, SCAMP5, CDC20, PTTG1, RPS19, TXNL4A, MNX1, EPHB3, SLC39A3, CHRNB2, UNC13A, LIG1, NUP210, KMT5C, SERPINF2, SOX12, MYO7A, BHLHA15, ATP11A, MAPK8IP2, SRCIN1, FOXP3, CDC25B, EID2, CLDN3, TIMELESS, CCDC86, FAM64A, ANAPC5, TSNARE1, RPP25, ABCG1, STX1A, SHMT2, PRKDC, CDCA5, DPT, FOXO6, C2CD4A, C2CD4B, C2CD4C, WDR62, SEZ6L2, PKMYT1, NCAPH, TIMM50, C2, NME1-NME2, VTN, TRPM2, ADAMTS14, DHX37, H1FX, BMF, SAC3D1, RTEL1-TNFRSF6B, PLK3, UBE2C, CBX2, ABCA7, NBEAL2, SYT7, PARP10, SDK1, NDUFAF8, KIF18B, ARTN, UBE2S, CDK4, FXYD2, MELTF, FGFR4, SNAP25, SERPINA1, GPAA1, MRPS12, CELF4, HEPACAM2, BICDL1, RND1, HEY1, ADAMTSL2, DACT2, MYBL2, EMX1, ATP6V0B, MYOC, MMP1, TESC, H2AFX, APOA2, SYP, ETV4, MTSS1, MMP11, INCENP, VWA1, PFDN2, KIF2C, MCM2, NAXE, UHRF1, LRP5, TIMM10, GDPD5, CENPA, CLN6, AURKA, RECQL4, CST3, TTR, PARD6A, GNG4, RAB26, POLD1, E2F1, RAD54L, CTHRC1, CMTM8, GINS2, LRRC24, NPM2, CDKN2A, GDF15, CADPS, CCK, NPM3, AGT, MAD2L2, HEYL, NFASC, PRC1, SHKBP1, APOC1, MFAP2, NEURL1, TACC3, INSM1, FOXA2 | 349 | 6265 | 16792 | 1.151986 | 1 | 0.439594 | 0.431901 |
| GOTERM_BP_ALL | GO:0050790~regulation of catalytic activity | 64 | 14.95327 | 0.019058 | SCARB1, SERPINA1, RTKN, SERPINE1, SERPINA10, STMN3, COX6A1, SHB, WFDC2, CDC20, DUSP10, RINL, PTTG1, SPR, EPHB3, SPINK4, PLEKHG4, MAP4K2, TESC, SERPINF2, APOA2, ADRA2C, RUNDC3A, SRCIN1, MAPK8IP1, CDC25B, CCNE1, DPEP1, CHI3L1, TRIB3, ANAPC5, SHC2, UHRF1, PPP1R35, RGS16, LRP5, DBF4B, PKMYT1, CST3, VTN, CST1, PCSK1N, SOCS1, PPP1R14B, PTPRN2, PPP1R14C, NPM2, CDKN2A, UBE2C, CCK, GUCA2A, AGT, MAD2L2, FABP4, UBE2S, ARTN, CDK4, NEURL1, APOC1, RIMBP2, MAP3K10, FGFR4, CDK5R2, FOXA2 | 349 | 2357 | 16792 | 1.306464 | 1 | 0.441098 | 0.433379 |
| GOTERM_BP_ALL | GO:0015918~sterol transport | 6 | 1.401869 | 0.019674 | SCARB1, APOC1, APOA2, ABCA7, ABCG1, SYT7 | 349 | 75 | 16792 | 3.849169 | 1 | 0.451535 | 0.443634 |
| GOTERM_BP_ALL | GO:0043276~anoikis | 4 | 0.934579 | 0.019704 | LOC102724428, E2F1, BMF, SIK1 | 349 | 28 | 16792 | 6.873516 | 1 | 0.451535 | 0.443634 |
| GOTERM_BP_ALL | GO:0001932~regulation of protein phosphorylation | 39 | 9.11215 | 0.019941 | SHC2, PRKDC, LRP5, DBF4B, PKMYT1, SHB, VTN, SOCS1, DUSP10, PARD6A, TNFRSF4, MAP4K2, MYOC, CDKN2A, GDF15, UBE2C, IL34, TESC, SERPINF2, ABCA7, CCK, ADRA2C, MAPK8IP2, SRCIN1, AGT, MAPK8IP1, CDC25B, MAD2L2, FABP4, CCNE1, CDK4, NEURL1, MAP3K10, CHI3L1, TNFRSF25, TRIB3, INSM1, FGFR4, CDK5R2 | 349 | 1300 | 16792 | 1.443438 | 1 | 0.454712 | 0.446755 |
| GOTERM_BP_ALL | GO:0019218~regulation of steroid metabolic process | 6 | 1.401869 | 0.020714 | DGAT2, APOC1, APOA2, FGFR4, ABCG1, AGT | 349 | 76 | 16792 | 3.798522 | 1 | 0.46549 | 0.457345 |
| GOTERM_BP_ALL | GO:0006835~dicarboxylic acid transport | 6 | 1.401869 | 0.020714 | SNAP25, CCK, SLC25A22, CPLX1, STX1A, PPFIA3 | 349 | 76 | 16792 | 3.798522 | 1 | 0.46549 | 0.457345 |
| GOTERM_BP_ALL | GO:0034374~low-density lipoprotein particle remodeling | 3 | 0.700935 | 0.020816 | APOA2, ABCG1, AGT | 349 | 11 | 16792 | 13.12217 | 1 | 0.46549 | 0.457345 |
| GOTERM_BP_ALL | GO:2000047~regulation of cell-cell adhesion mediated by cadherin | 3 | 0.700935 | 0.020816 | MAD2L2, SERPINF2, FOXA2 | 349 | 11 | 16792 | 13.12217 | 1 | 0.46549 | 0.457345 |
| GOTERM_BP_ALL | GO:0051783~regulation of nuclear division | 9 | 2.102804 | 0.021147 | MAD2L2, CDC20, NPM2, UBE2C, CDCA5, LRP5, TACC3, PKMYT1, AURKA | 349 | 164 | 16792 | 2.640436 | 1 | 0.468363 | 0.460167 |
| GOTERM_BP_ALL | GO:0009749~response to glucose | 9 | 2.102804 | 0.021147 | NME1-NME2, PTPRN2, RFX6, KCNJ11, UCP2, APOA2, NR0B2, AACS, FOXA2 | 349 | 164 | 16792 | 2.640436 | 1 | 0.468363 | 0.460167 |
| GOTERM_BP_ALL | GO:0033993~response to lipid | 28 | 6.542056 | 0.021422 | SPON2, SCARB1, CEBPB, SERPINE1, ATP1A3, ASCL1, AQP3, AACS, NME1-NME2, CST3, DUSP10, HEY1, UCP2, E2F1, FFAR2, TNFRSF4, DGAT2, KCNJ11, ABCC8, TESC, APOA2, NR0B2, HEYL, CCNE1, CDK4, TNFRSF25, FGFR4, ABCG1 | 349 | 860 | 16792 | 1.566522 | 1 | 0.472212 | 0.463949 |
| GOTERM_BP_ALL | GO:0097421~liver regeneration | 4 | 0.934579 | 0.021648 | VTN, CEBPB, UCP2, AURKA | 349 | 29 | 16792 | 6.636498 | 1 | 0.474916 | 0.466605 |
| GOTERM_BP_ALL | GO:0009743~response to carbohydrate | 10 | 2.336449 | 0.022544 | NME1-NME2, CST3, PTPRN2, RFX6, KCNJ11, UCP2, APOA2, NR0B2, AACS, FOXA2 | 349 | 198 | 16792 | 2.430031 | 1 | 0.492252 | 0.483639 |
| GOTERM_BP_ALL | GO:0021987~cerebral cortex development | 7 | 1.635514 | 0.022971 | EMX1, ADGRG1, H2AFX, TACC3, WDR62, ASCL1, CDK5R2 | 349 | 106 | 16792 | 3.17738 | 1 | 0.499225 | 0.490489 |
| GOTERM_BP_ALL | GO:0052548~regulation of endopeptidase activity | 15 | 3.504673 | 0.02339 | SPINK4, SERPINA1, CDKN2A, SERPINF2, SERPINA10, SERPINE1, CCK, WFDC2, AGT, CST3, VTN, PCSK1N, CST1, PTTG1, DPEP1 | 349 | 372 | 16792 | 1.940105 | 1 | 0.503417 | 0.494609 |
| GOTERM_BP_ALL | GO:0009628~response to abiotic stimulus | 32 | 7.476636 | 0.023413 | CLPB, CRTC1, PRKDC, PLEKHB1, ATP1A3, SCX, ASCL1, ASCL2, CRIP1, RELB, CST3, TRPM2, PCSK1N, POLD1, UCP2, E2F1, RAD54L, CHRNB2, PLK3, ABCC8, H2AFX, PGF, AGT, LOC102724428, CLDN3, VGF, CDK4, TIMELESS, CHI3L1, SIK1, KCNK3, STX1A | 349 | 1029 | 16792 | 1.496276 | 1 | 0.503417 | 0.494609 |
| GOTERM_BP_ALL | GO:0010564~regulation of cell cycle process | 21 | 4.906542 | 0.023491 | PLK3, CDT1, TPRA1, NPM2, CDKN2A, PRKDC, UBE2C, CDCA5, LRP5, ARID3A, DBF4B, PKMYT1, CDC25B, AURKA, MAD2L2, CDC20, CDK4, E2F1, TACC3, ANAPC5, INSM1 | 349 | 595 | 16792 | 1.698163 | 1 | 0.503417 | 0.494609 |
| GOTERM_BP_ALL | GO:0045746~negative regulation of Notch signaling pathway | 4 | 0.934579 | 0.023692 | DLL4, HEY1, NEURL1, GDPD5 | 349 | 30 | 16792 | 6.415282 | 1 | 0.503673 | 0.49486 |
| GOTERM_BP_ALL | GO:0009952~anterior/posterior pattern specification | 10 | 2.336449 | 0.023905 | HOXB9, PRKDC, LRP5, HOXB2, HOXC9, HOXC10, FOXA2, AURKA, HOXC6, DLL3 | 349 | 200 | 16792 | 2.405731 | 1 | 0.503673 | 0.49486 |
| GOTERM_BP_ALL | GO:0002700~regulation of production of molecular mediator of immune response | 7 | 1.635514 | 0.023928 | SPON2, PRKDC, APOA2, FFAR2, PGC, TNFRSF4, FOXP3 | 349 | 107 | 16792 | 3.147685 | 1 | 0.503673 | 0.49486 |
| GOTERM_BP_ALL | GO:0021515~cell differentiation in spinal cord | 5 | 1.168224 | 0.023948 | DLL4, GDPD5, ASCL1, MNX1, HOXC10 | 349 | 53 | 16792 | 4.539114 | 1 | 0.503673 | 0.49486 |
| GOTERM_BP_ALL | GO:0031145~anaphase-promoting complex-dependent catabolic process | 6 | 1.401869 | 0.024047 | CDC20, PTTG1, UBE2C, UBE2S, ANAPC5, AURKA | 349 | 79 | 16792 | 3.654274 | 1 | 0.503673 | 0.49486 |
| GOTERM_BP_ALL | GO:0051090~regulation of sequence-specific DNA binding transcription factor activity | 15 | 3.504673 | 0.024156 | BEX1, CDKN2A, CRTC1, LRP5, NR0B2, FOXP3, AGT, PARP10, MAD2L2, HEYL, LOC102724428, MAP3K10, SIK1, TNFRSF4, FOXA2 | 349 | 373 | 16792 | 1.934904 | 1 | 0.503692 | 0.494878 |
| GOTERM_BP_ALL | GO:0034434~sterol esterification | 3 | 0.700935 | 0.024641 | APOC1, APOA2, AGT | 349 | 12 | 16792 | 12.02865 | 1 | 0.504766 | 0.495934 |
| GOTERM_BP_ALL | GO:0034435~cholesterol esterification | 3 | 0.700935 | 0.024641 | APOC1, APOA2, AGT | 349 | 12 | 16792 | 12.02865 | 1 | 0.504766 | 0.495934 |
| GOTERM_BP_ALL | GO:0034433~steroid esterification | 3 | 0.700935 | 0.024641 | APOC1, APOA2, AGT | 349 | 12 | 16792 | 12.02865 | 1 | 0.504766 | 0.495934 |
| GOTERM_BP_ALL | GO:0032268~regulation of cellular protein metabolic process | 63 | 14.71963 | 0.024744 | SERPINA1, SERPINE1, SERPINA10, CELF4, SHB, WFDC2, CDC20, DUSP10, PTTG1, TNFRSF4, SPINK4, MAP4K2, MYOC, TESC, SERPINF2, ADRA2C, MAPK8IP2, SRCIN1, FOXP3, MAPK8IP1, CDC25B, CCNE1, DPEP1, CHI3L1, TNFRSF25, TRIB3, ANAPC5, SHC2, UHRF1, PRKDC, LRP5, DBF4B, PKMYT1, CLN6, AURKA, CST3, VTN, CST1, PCSK1N, PARD6A, SOCS1, DTD1, PLK3, PPP1R14B, PPP1R14C, CDKN2A, GDF15, UBE2C, IL34, ABCA7, CCK, AGT, PARP10, MAD2L2, FABP4, UBE2S, CDK4, NEURL1, MAP3K10, MELTF, INSM1, FGFR4, CDK5R2 | 349 | 2346 | 16792 | 1.29208 | 1 | 0.504766 | 0.495934 |
| GOTERM_BP_ALL | GO:0009746~response to hexose | 9 | 2.102804 | 0.02477 | NME1-NME2, PTPRN2, RFX6, KCNJ11, UCP2, APOA2, NR0B2, AACS, FOXA2 | 349 | 169 | 16792 | 2.562317 | 1 | 0.504766 | 0.495934 |
| GOTERM_BP_ALL | GO:0071156~regulation of cell cycle arrest | 7 | 1.635514 | 0.02491 | PLK3, CDKN2A, CDK4, E2F1, ARID3A, INSM1, AURKA | 349 | 108 | 16792 | 3.11854 | 1 | 0.504766 | 0.495934 |
| GOTERM_BP_ALL | GO:0002526~acute inflammatory response | 8 | 1.869159 | 0.024971 | VTN, REG3A, CEBPB, SERPINA1, SERPINF2, APOA2, FFAR2, C2 | 349 | 138 | 16792 | 2.789253 | 1 | 0.504766 | 0.495934 |
| GOTERM_BP_ALL | GO:0060627~regulation of vesicle-mediated transport | 17 | 3.971963 | 0.025324 | SCARB1, SERPINE1, C2CD4A, ABCA7, C2CD4B, C2CD4C, CPLX2, CPLX1, SYT7, C2, SCAMP5, VTN, RAB26, RAB15, APOC1, CDK5R2, STX1A | 349 | 449 | 16792 | 1.821711 | 1 | 0.509675 | 0.500756 |
| GOTERM_BP_ALL | GO:0051253~negative regulation of RNA metabolic process | 37 | 8.64486 | 0.025825 | MESP1, CEBPB, ZNF296, UHRF1, CELF4, TONSL, SCX, ASCL1, ASCL2, RELB, DLL4, PCBP3, HEY1, SOX18, DHX34, SALL4, E2F1, PRAME, TNFRSF4, HOXC6, TGIF1, PLK3, CDKN2A, CBX2, H2AFX, NR0B2, FOXP3, MAD2L2, DRAP1, HEYL, EID2, FABP4, TIMELESS, MAP3K10, TRIB3, INSM1, FOXA2 | 349 | 1240 | 16792 | 1.435678 | 1 | 0.515535 | 0.506513 |
| GOTERM_BP_ALL | GO:1903053~regulation of extracellular matrix organization | 4 | 0.934579 | 0.025838 | CST3, MELTF, FGFR4, AGT | 349 | 31 | 16792 | 6.208337 | 1 | 0.515535 | 0.506513 |
| GOTERM_BP_ALL | GO:0006469~negative regulation of protein kinase activity | 11 | 2.570093 | 0.026483 | DUSP10, FABP4, SOCS1, CDKN2A, UBE2C, TESC, LRP5, TRIB3, SHB, SRCIN1, MAPK8IP1 | 349 | 237 | 16792 | 2.233168 | 1 | 0.526129 | 0.516922 |
| GOTERM_BP_ALL | GO:0007088~regulation of mitotic nuclear division | 8 | 1.869159 | 0.026743 | MAD2L2, NPM2, UBE2C, CDCA5, LRP5, TACC3, PKMYT1, AURKA | 349 | 140 | 16792 | 2.749406 | 1 | 0.529034 | 0.519777 |
| GOTERM_BP_ALL | GO:0051050~positive regulation of transport | 29 | 6.775701 | 0.027135 | SERPINE1, CACNA1D, AACS, C2, SCAMP5, VTN, TRPM2, E2F1, FFAR2, BMF, TNFRSF4, KCNH2, CHRNB2, PLK3, UNC13A, KCNJ11, ABCC8, TESC, ABCA7, CCK, NR0B2, SYT7, AGT, RFX6, RAB15, ORAI1, ABCG1, STX1A, CDK5R2 | 349 | 920 | 16792 | 1.516656 | 1 | 0.534496 | 0.525143 |
| GOTERM_BP_ALL | GO:0010038~response to metal ion | 13 | 3.037383 | 0.027468 | ABCC8, CPNE7, NUDT1, GGH, CYBRD1, ASCL1, AQP3, CRIP1, TRPM2, FABP4, CDK4, DPEP1, KCNK3 | 349 | 308 | 16792 | 2.030812 | 1 | 0.537372 | 0.527969 |
| GOTERM_BP_ALL | GO:0001708~cell fate specification | 6 | 1.401869 | 0.027706 | MESP1, SOX18, ASCL1, MNX1, HOXC10, FOXA2 | 349 | 82 | 16792 | 3.520581 | 1 | 0.537372 | 0.527969 |
| GOTERM_BP_ALL | GO:0006906~vesicle fusion | 7 | 1.635514 | 0.028018 | SNAP25, C2CD4A, C2CD4B, TSNARE1, C2CD4C, STX1A, SYT7 | 349 | 111 | 16792 | 3.034255 | 1 | 0.537372 | 0.527969 |
| GOTERM_BP_ALL | GO:0090207~regulation of triglyceride metabolic process | 4 | 0.934579 | 0.028084 | LOC102724428, SCARB1, DGAT2, SIK1 | 349 | 32 | 16792 | 6.014327 | 1 | 0.537372 | 0.527969 |
| GOTERM_BP_ALL | GO:0034381~plasma lipoprotein particle clearance | 4 | 0.934579 | 0.028084 | SCARB1, DGAT2, APOC1, APOA2 | 349 | 32 | 16792 | 6.014327 | 1 | 0.537372 | 0.527969 |
| GOTERM_BP_ALL | GO:0022616~DNA strand elongation | 4 | 0.934579 | 0.028084 | GINS2, RNASEH2A, LIG1, POLD1 | 349 | 32 | 16792 | 6.014327 | 1 | 0.537372 | 0.527969 |
| GOTERM_BP_ALL | GO:0009888~tissue development | 49 | 11.4486 | 0.028093 | MESP1, SCX, MYL6B, AQP3, AACS, HEY1, SOX18, SALL4, ADAMTSL2, DACT2, EMX1, DGAT2, MYO7A, ETV4, MTSS1, PGF, LOC102724428, CLDN3, RFX6, TIMELESS, ORAI1, CHI3L1, HOXB2, SIK1, REG3A, CEBPB, PRKDC, LRP5, ASCL1, ASCL2, CRIP1, DLL3, NME1-NME2, DLL4, CST3, VTN, PARD6A, MTHFD1L, CTHRC1, STAC3, SMAD9, AGT, MAD2L2, HEYL, ARTN, MAFG, NEURL1, INSM1, FOXA2 | 349 | 1756 | 16792 | 1.342606 | 1 | 0.537372 | 0.527969 |
| GOTERM_BP_ALL | GO:0051785~positive regulation of nuclear division | 5 | 1.168224 | 0.028636 | NPM2, UBE2C, CDCA5, LRP5, AURKA | 349 | 56 | 16792 | 4.295948 | 1 | 0.543262 | 0.533756 |
| GOTERM_BP_ALL | GO:0051937~catecholamine transport | 5 | 1.168224 | 0.028636 | CHRNB2, CADPS, ADRA2C, STX1A, AGT | 349 | 56 | 16792 | 4.295948 | 1 | 0.543262 | 0.533756 |
| GOTERM_BP_ALL | GO:0034284~response to monosaccharide | 9 | 2.102804 | 0.028807 | NME1-NME2, PTPRN2, RFX6, KCNJ11, UCP2, APOA2, NR0B2, AACS, FOXA2 | 349 | 174 | 16792 | 2.488687 | 1 | 0.543326 | 0.533818 |
| GOTERM_BP_ALL | GO:0045833~negative regulation of lipid metabolic process | 6 | 1.401869 | 0.029 | LOC102724428, DGAT2, APOC1, APOA2, TRIB3, SIK1 | 349 | 83 | 16792 | 3.478165 | 1 | 0.543326 | 0.533818 |
| GOTERM_BP_ALL | GO:0009891~positive regulation of biosynthetic process | 49 | 11.4486 | 0.029081 | SCARB1, MESP1, DYRK2, CRTC1, SERPINE1, SCX, HEY1, SOX18, SALL4, ZXDC, MYBL2, DGAT2, TESC, SERPINF2, APOA2, SOX12, BHLHA15, RUNDC3A, ETV4, FOXP3, HOXB9, RFX6, CCNE1, ABCG1, CEBPB, BEX1, UHRF1, PRKDC, LRP5, DBF4B, ASCL1, RELB, NME1-NME2, CST3, E2F1, POLR2I, NPM2, CDKN2A, SMAD9, ARID3A, GUCA2A, AGT, MAD2L2, HEYL, CDK4, MAFG, FGFR4, RNF187, FOXA2 | 349 | 1760 | 16792 | 1.339555 | 1 | 0.543326 | 0.533818 |
| GOTERM_BP_ALL | GO:0045598~regulation of fat cell differentiation | 7 | 1.635514 | 0.029109 | MMP11, CEBPB, TPH1, DUSP10, LRP5, E2F1, TRIB3 | 349 | 112 | 16792 | 3.007163 | 1 | 0.543326 | 0.533818 |
| GOTERM_BP_ALL | GO:0016043~cellular component organization | 145 | 33.8785 | 0.029308 | SPON2, SCARB1, CRTC1, HDAC11, SERPINE1, PPAN, SCX, STMN3, TPGS1, AQP3, IPO4, SCAMP5, CDC20, PTTG1, RPS19, TXNL4A, MNX1, EPHB3, SLC39A3, CHRNB2, UNC13A, LIG1, NUP210, KMT5C, SERPINF2, SOX12, MYO7A, BHLHA15, ATP11A, MAPK8IP2, SRCIN1, FOXP3, CDC25B, EID2, CLDN3, TIMELESS, FAM64A, ANAPC5, TSNARE1, ABCG1, STX1A, SHMT2, PRKDC, CDCA5, DPT, FOXO6, C2CD4A, C2CD4B, C2CD4C, WDR62, SEZ6L2, PKMYT1, NCAPH, TIMM50, C2, NME1-NME2, VTN, TRPM2, ADAMTS14, H1FX, BMF, SAC3D1, RTEL1-TNFRSF6B, PLK3, UBE2C, CBX2, ABCA7, NBEAL2, SYT7, PARP10, SDK1, NDUFAF8, KIF18B, ARTN, UBE2S, CDK4, FXYD2, MELTF, FGFR4, SNAP25, SERPINA1, GPAA1, MRPS12, CELF4, HEPACAM2, BICDL1, RND1, HEY1, ADAMTSL2, DACT2, MYBL2, EMX1, ATP6V0B, MYOC, MMP1, TESC, H2AFX, APOA2, SYP, ETV4, MTSS1, MMP11, INCENP, VWA1, PFDN2, KIF2C, MCM2, NAXE, UHRF1, LRP5, TIMM10, GDPD5, CENPA, CLN6, AURKA, RECQL4, CST3, TTR, PARD6A, GNG4, RAB26, POLD1, E2F1, RAD54L, CTHRC1, CMTM8, GINS2, LRRC24, NPM2, CDKN2A, GDF15, CADPS, CCK, AGT, MAD2L2, HEYL, NFASC, PRC1, SHKBP1, APOC1, MFAP2, NEURL1, TACC3, INSM1, FOXA2 | 349 | 6109 | 16792 | 1.142023 | 1 | 0.544838 | 0.535304 |
| GOTERM_BP_ALL | GO:0048878~chemical homeostasis | 32 | 7.476636 | 0.029568 | SCARB1, LRP5, ATP1A3, AQP3, AACS, CLN6, NME1-NME2, TRPM2, POLD1, UCP2, FFAR2, KCNH2, ATP6V0B, PTPRN2, DGAT2, MYOC, KCNJ11, APOA2, ASPSCR1, CYBRD1, CCK, BHLHA15, AGT, FAM155B, FABP4, VGF, RFX6, MAFG, MELTF, FGFR4, KCNK3, ABCG1 | 349 | 1049 | 16792 | 1.467748 | 1 | 0.54748 | 0.5379 |
| GOTERM_BP_ALL | GO:0048278~vesicle docking | 5 | 1.168224 | 0.030311 | SNAP25, UNC13A, TSNARE1, CPLX2, STX1A | 349 | 57 | 16792 | 4.22058 | 1 | 0.559005 | 0.549223 |
| GOTERM_BP_ALL | GO:0045165~cell fate commitment | 11 | 2.570093 | 0.030558 | DLL4, MESP1, CEBPB, PRKDC, SOX18, SOX12, ASCL1, FOXP3, MNX1, HOXC10, FOXA2 | 349 | 243 | 16792 | 2.178028 | 1 | 0.560296 | 0.550492 |
| GOTERM_BP_ALL | GO:0006468~protein phosphorylation | 51 | 11.91589 | 0.03069 | DYRK2, SHB, LIPE, DUSP10, TNFRSF4, EPHB3, KCNH2, MAP4K2, RPS6KL1, MYOC, TESC, SERPINF2, ADRA2C, MAPK8IP2, SRCIN1, MAPK8IP1, CDC25B, LOC102724428, CCNE1, CHI3L1, TNFRSF25, TRIB3, SIK1, SHC2, PRKDC, LRP5, DBF4B, PKMYT1, AURKA, NME1-NME2, VTN, PARD6A, SOCS1, PLK3, CDKN2A, GDF15, UBE2C, IL34, SMAD9, ABCA7, CCK, AGT, MAD2L2, FABP4, ARTN, CDK4, NEURL1, MAP3K10, INSM1, FGFR4, CDK5R2 | 349 | 1852 | 16792 | 1.32497 | 1 | 0.560296 | 0.550492 |
| GOTERM_BP_ALL | GO:0045859~regulation of protein kinase activity | 24 | 5.607477 | 0.030744 | MAP4K2, SHC2, CDKN2A, UBE2C, TESC, LRP5, DBF4B, PKMYT1, ADRA2C, SHB, SRCIN1, AGT, CDC25B, MAPK8IP1, SOCS1, DUSP10, FABP4, CCNE1, CDK4, NEURL1, MAP3K10, CHI3L1, TRIB3, CDK5R2 | 349 | 730 | 16792 | 1.58185 | 1 | 0.560296 | 0.550492 |
| GOTERM_BP_ALL | GO:1903507~negative regulation of nucleic acid-templated transcription | 35 | 8.17757 | 0.031113 | MESP1, CEBPB, ZNF296, UHRF1, TONSL, SCX, ASCL1, ASCL2, RELB, DLL4, PCBP3, HEY1, SOX18, SALL4, E2F1, PRAME, TNFRSF4, HOXC6, TGIF1, PLK3, CDKN2A, CBX2, H2AFX, NR0B2, FOXP3, MAD2L2, DRAP1, HEYL, EID2, FABP4, TIMELESS, MAP3K10, TRIB3, INSM1, FOXA2 | 349 | 1177 | 16792 | 1.430766 | 1 | 0.564788 | 0.554905 |
| GOTERM_BP_ALL | GO:0071158~positive regulation of cell cycle arrest | 6 | 1.401869 | 0.031702 | PLK3, CDKN2A, E2F1, ARID3A, INSM1, AURKA | 349 | 85 | 16792 | 3.396326 | 1 | 0.57323 | 0.563199 |
| GOTERM_BP_ALL | GO:0031328~positive regulation of cellular biosynthetic process | 48 | 11.21495 | 0.03201 | MESP1, SCARB1, CEBPB, BEX1, DYRK2, UHRF1, CRTC1, PRKDC, SERPINE1, LRP5, SCX, DBF4B, ASCL1, RELB, NME1-NME2, CST3, HEY1, SOX18, SALL4, ZXDC, E2F1, MYBL2, POLR2I, DGAT2, NPM2, CDKN2A, TESC, SERPINF2, APOA2, SMAD9, ARID3A, SOX12, BHLHA15, ETV4, RUNDC3A, FOXP3, GUCA2A, AGT, MAD2L2, HEYL, HOXB9, RFX6, CCNE1, CDK4, MAFG, FGFR4, RNF187, FOXA2 | 349 | 1729 | 16792 | 1.335744 | 1 | 0.576085 | 0.566005 |
| GOTERM_BP_ALL | GO:0051246~regulation of protein metabolic process | 66 | 15.42056 | 0.032108 | SERPINA1, SERPINE1, SERPINA10, CELF4, SHB, WFDC2, CDC20, DUSP10, PTTG1, TNFRSF4, SPINK4, MAP4K2, MYOC, TESC, SERPINF2, APOA2, ADRA2C, MAPK8IP2, SRCIN1, FOXP3, MAPK8IP1, CDC25B, CCNE1, DPEP1, CHI3L1, TNFRSF25, TRIB3, ANAPC5, CEBPB, SHC2, UHRF1, PRKDC, LRP5, DBF4B, PKMYT1, CLN6, AURKA, C2, CST3, VTN, CST1, PCSK1N, PARD6A, SOCS1, DTD1, PLK3, PPP1R14B, PPP1R14C, CDKN2A, GDF15, UBE2C, IL34, ABCA7, CCK, AGT, PARP10, MAD2L2, FABP4, UBE2S, CDK4, NEURL1, MAP3K10, MELTF, INSM1, FGFR4, CDK5R2 | 349 | 2512 | 16792 | 1.264158 | 1 | 0.576085 | 0.566005 |
| GOTERM_BP_ALL | GO:0051726~regulation of cell cycle | 30 | 7.009346 | 0.032268 | BEX2, PRKDC, CDCA5, LRP5, DBF4B, PKMYT1, GDPD5, ASCL1, SHB, AURKA, CDC20, E2F1, MYBL2, CDT1, PLK3, TPRA1, NPM2, CDKN2A, UBE2C, ARID3A, CDC25B, MAD2L2, LOC102724428, CCNE1, CDK4, TACC3, SIK1, ANAPC5, INSM1, CDK5R2 | 349 | 976 | 16792 | 1.478933 | 1 | 0.576715 | 0.566624 |
| GOTERM_BP_ALL | GO:0042558~pteridine-containing compound metabolic process | 4 | 0.934579 | 0.032878 | SPR, MTHFD1L, SHMT2, GGH | 349 | 34 | 16792 | 5.660543 | 1 | 0.585355 | 0.575112 |
| GOTERM_BP_ALL | GO:0048172~regulation of short-term neuronal synaptic plasticity | 3 | 0.700935 | 0.033064 | UNC13A, SYP, PPFIA3 | 349 | 14 | 16792 | 10.31027 | 1 | 0.586419 | 0.576157 |
| GOTERM_BP_ALL | GO:0050896~response to stimulus | 191 | 44.62617 | 0.033594 | SPON2, SCARB1, RTKN, CLPB, CRTC1, CPNE7, PLEKHB1, SERPINE1, NUDT1, TONSL, SCX, STMN3, AQP3, SCAMP5, TMEM145, PTTG1, RPS19, SALL4, TNFRSF4, MNX1, EPHB3, IER3, CHRNB2, SPINK4, KCNH2, TPRA1, DGAT2, UNC13A, LIG1, NUP210, SERPINF2, BHLHA15, MAPK8IP2, PGC, RUNDC3A, FOXP3, PGF, MAPK8IP1, TRAIP, LOC102724428, EID2, CLDN3, HOXB9, RFX6, CCNE1, TIMELESS, DPEP1, ORAI1, SIK1, TRIB3, PLPP2, ABCG1, STX1A, LY6E, REG3A, GNAZ, SHC2, TPH1, RNASEH2A, SHMT2, PRKDC, CDCA5, CACNA1D, CPLX2, SEZ6L2, TIMM50, C2, LYPD8, NME1-NME2, VTN, TRPM2, ADGRG1, PCSK1N, SOCS1, ADGRG5, RHPN1, BMF, CYC1, RTEL1-TNFRSF6B, PLK3, PTPRN2, STARD10, CYBRD1, GGH, SMAD9, ABCA7, NR0B2, SYT7, TMEM198, SDK1, ARTN, CDK4, MAFG, MAP3K10, SP5, ULBP2, FGFR4, MESP1, SERPINA1, DAGLA, DYRK2, ZC3H3, SERPINA10, CELF4, SHB, ASGR1, AACS, RND1, DUSP10, HEY1, ADAMTSL2, DACT2, FFAR2, CA8, GRASP, SLC18A1, TEAD4, TGIF1, PLEKHG4, EMX1, MAP4K2, ATP6V0B, RPS6KL1, MYOC, ABCC8, GP1BB, TESC, H2AFX, APOA2, SYP, ADRA2C, ETV4, MTSS1, VGF, VWA1, CHI3L1, TNFRSF25, MCM2, CEBPB, UHRF1, RGS16, LRP5, ATP1A3, GDPD5, ASCL1, CRIP1, ASCL2, RELB, AURKA, DLL3, DLL4, RECQL4, CST3, P2RY6, CST1, PARD6A, NPW, GNG4, RAB26, POLD1, UCP2, E2F1, RAD54L, PRAME, POLR2I, CTHRC1, OR51E1, CMTM8, GINS2, PPP1R14B, KCNJ11, ZNF580, CDKN2A, GDF15, IL34, GAD1, STAC3, ARID3A, CCK, AGT, MAD2L2, HEYL, NFASC, FABP4, RAB15, TSPAN18, SMOC1, SHKBP1, NEURL1, KCNK3, FOXA2 | 349 | 8329 | 16792 | 1.103361 | 1 | 0.593531 | 0.583145 |
| GOTERM_BP_ALL | GO:0090174~organelle membrane fusion | 7 | 1.635514 | 0.033748 | SNAP25, C2CD4A, C2CD4B, TSNARE1, C2CD4C, STX1A, SYT7 | 349 | 116 | 16792 | 2.903468 | 1 | 0.593991 | 0.583597 |
| GOTERM_BP_ALL | GO:0035295~tube development | 20 | 4.672897 | 0.034185 | MESP1, LRP5, SMAD9, ASCL1, ETV4, PGF, AGT, MTSS1, DLL4, HEYL, ADGRG1, MTHFD1L, SOX18, SALL4, ADAMTSL2, DACT2, TIMELESS, CHI3L1, EPHB3, CTHRC1 | 349 | 580 | 16792 | 1.659125 | 1 | 0.599397 | 0.588908 |
| GOTERM_BP_ALL | GO:0050793~regulation of developmental process | 57 | 13.31776 | 0.035238 | SNAP25, MESP1, CRTC1, SERPINE1, CELF4, SCX, SHB, AQP3, CDC20, DUSP10, HEY1, RPS19, PHLDA2, EPHB3, CHRNB2, EMX1, UNC13A, MYOC, TESC, BHLHA15, ADRA2C, ETV4, SRCIN1, FOXP3, PGF, LOC102724428, CHI3L1, SIK1, REG3A, CEBPB, PRKDC, FOXO6, GDPD5, ASCL1, ASCL2, AURKA, DLL3, NME1-NME2, DLL4, CST3, ADGRG1, PARD6A, CTHRC1, LRRC24, CDKN2A, GDF15, IL34, TMEM176B, TMEM176A, AGT, MAD2L2, HEYL, SDK1, MAFG, NEURL1, MELTF, FOXA2 | 349 | 2129 | 16792 | 1.288179 | 1 | 0.614152 | 0.603405 |
| GOTERM_BP_ALL | GO:0006904~vesicle docking involved in exocytosis | 4 | 0.934579 | 0.035424 | SNAP25, UNC13A, CPLX2, STX1A | 349 | 35 | 16792 | 5.498813 | 1 | 0.614152 | 0.603405 |
| GOTERM_BP_ALL | GO:0045742~positive regulation of epidermal growth factor receptor signaling pathway | 4 | 0.934579 | 0.035424 | SHKBP1, NEURL1, ADRA2C, AGT | 349 | 35 | 16792 | 5.498813 | 1 | 0.614152 | 0.603405 |
| GOTERM_BP_ALL | GO:0045935~positive regulation of nucleobase-containing compound metabolic process | 46 | 10.74766 | 0.03607 | MESP1, CEBPB, BEX1, UHRF1, CRTC1, PRKDC, SERPINE1, LRP5, CELF4, SCX, DBF4B, ASCL1, RELB, NME1-NME2, CST3, HEY1, SOX18, SALL4, ZXDC, E2F1, MYBL2, POLR2I, NPM2, CDKN2A, TESC, H2AFX, SERPINF2, SMAD9, ARID3A, SOX12, BHLHA15, ETV4, RUNDC3A, FOXP3, GUCA2A, AGT, MAD2L2, HEYL, HOXB9, RFX6, CCNE1, MAFG, TIMELESS, FGFR4, RNF187, FOXA2 | 349 | 1658 | 16792 | 1.334905 | 1 | 0.6184 | 0.607579 |
| GOTERM_BP_ALL | GO:0071805~potassium ion transmembrane transport | 9 | 2.102804 | 0.03616 | KCNH2, KCNJ11, KCNH6, ABCC8, FXYD2, ATP1A3, CACNA1D, KCNAB2, KCNK3 | 349 | 182 | 16792 | 2.379294 | 1 | 0.6184 | 0.607579 |
| GOTERM_BP_ALL | GO:0071804~cellular potassium ion transport | 9 | 2.102804 | 0.03616 | KCNH2, KCNJ11, KCNH6, ABCC8, FXYD2, ATP1A3, CACNA1D, KCNAB2, KCNK3 | 349 | 182 | 16792 | 2.379294 | 1 | 0.6184 | 0.607579 |
| GOTERM_BP_ALL | GO:0010035~response to inorganic substance | 17 | 3.971963 | 0.036204 | ZNF580, ABCC8, CPNE7, NUDT1, GGH, CYBRD1, ASCL1, AQP3, CRIP1, CST3, TRPM2, FABP4, CDK4, UCP2, TIMELESS, DPEP1, KCNK3 | 349 | 469 | 16792 | 1.744026 | 1 | 0.6184 | 0.607579 |
| GOTERM_BP_ALL | GO:1902679~negative regulation of RNA biosynthetic process | 35 | 8.17757 | 0.036614 | MESP1, CEBPB, ZNF296, UHRF1, TONSL, SCX, ASCL1, ASCL2, RELB, DLL4, PCBP3, HEY1, SOX18, SALL4, E2F1, PRAME, TNFRSF4, HOXC6, TGIF1, PLK3, CDKN2A, CBX2, H2AFX, NR0B2, FOXP3, MAD2L2, DRAP1, HEYL, EID2, FABP4, TIMELESS, MAP3K10, TRIB3, INSM1, FOXA2 | 349 | 1194 | 16792 | 1.410395 | 1 | 0.622237 | 0.611348 |
| GOTERM_BP_ALL | GO:0052547~regulation of peptidase activity | 15 | 3.504673 | 0.037356 | SPINK4, SERPINA1, CDKN2A, SERPINF2, SERPINA10, SERPINE1, CCK, WFDC2, AGT, CST3, VTN, PCSK1N, CST1, PTTG1, DPEP1 | 349 | 396 | 16792 | 1.822523 | 1 | 0.622237 | 0.611348 |
| GOTERM_BP_ALL | GO:0098813~nuclear chromosome segregation | 12 | 2.803738 | 0.037443 | MAD2L2, CDC20, KIF18B, PTTG1, INCENP, UBE2C, PRC1, CDCA5, TACC3, KIF2C, CENPA, NCAPH | 349 | 287 | 16792 | 2.011761 | 1 | 0.622237 | 0.611348 |
| GOTERM_BP_ALL | GO:0031399~regulation of protein modification process | 46 | 10.74766 | 0.037464 | SHC2, PRKDC, LRP5, DBF4B, PKMYT1, SHB, CDC20, VTN, SOCS1, DUSP10, PARD6A, TNFRSF4, MAP4K2, PPP1R14B, PPP1R14C, MYOC, CDKN2A, GDF15, UBE2C, IL34, TESC, SERPINF2, ABCA7, CCK, ADRA2C, MAPK8IP2, SRCIN1, FOXP3, AGT, MAPK8IP1, CDC25B, PARP10, MAD2L2, FABP4, CCNE1, UBE2S, CDK4, NEURL1, MAP3K10, CHI3L1, TNFRSF25, TRIB3, ANAPC5, INSM1, FGFR4, CDK5R2 | 349 | 1664 | 16792 | 1.330091 | 1 | 0.622237 | 0.611348 |
| GOTERM_BP_ALL | GO:0042770~signal transduction in response to DNA damage | 7 | 1.635514 | 0.037525 | MAD2L2, PLK3, CDKN2A, PRKDC, E2F1, ARID3A, AURKA | 349 | 119 | 16792 | 2.830271 | 1 | 0.622237 | 0.611348 |
| GOTERM_BP_ALL | GO:0051438~regulation of ubiquitin-protein transferase activity | 7 | 1.635514 | 0.037525 | MAD2L2, CDC20, CDKN2A, UBE2C, UBE2S, TRIB3, ANAPC5 | 349 | 119 | 16792 | 2.830271 | 1 | 0.622237 | 0.611348 |
| GOTERM_BP_ALL | GO:0042136~neurotransmitter biosynthetic process | 3 | 0.700935 | 0.037638 | DAGLA, GAD1, PAH | 349 | 15 | 16792 | 9.622923 | 1 | 0.622237 | 0.611348 |
| GOTERM_BP_ALL | GO:0044331~cell-cell adhesion mediated by cadherin | 3 | 0.700935 | 0.037638 | MAD2L2, SERPINF2, FOXA2 | 349 | 15 | 16792 | 9.622923 | 1 | 0.622237 | 0.611348 |
| GOTERM_BP_ALL | GO:0010715~regulation of extracellular matrix disassembly | 3 | 0.700935 | 0.037638 | CST3, MELTF, FGFR4 | 349 | 15 | 16792 | 9.622923 | 1 | 0.622237 | 0.611348 |
| GOTERM_BP_ALL | GO:1901186~positive regulation of ERBB signaling pathway | 4 | 0.934579 | 0.038069 | SHKBP1, NEURL1, ADRA2C, AGT | 349 | 36 | 16792 | 5.346068 | 1 | 0.627124 | 0.61615 |
| GOTERM_BP_ALL | GO:0043549~regulation of kinase activity | 25 | 5.841121 | 0.038807 | SHC2, LRP5, DBF4B, PKMYT1, SHB, SOCS1, DUSP10, MAP4K2, CDKN2A, UBE2C, TESC, ADRA2C, SRCIN1, AGT, MAPK8IP1, CDC25B, FABP4, CCNE1, CDK4, NEURL1, MAP3K10, CHI3L1, TRIB3, CDK5R2, FOXA2 | 349 | 788 | 16792 | 1.526479 | 1 | 0.633974 | 0.622881 |
| GOTERM_BP_ALL | GO:0000122~negative regulation of transcription from RNA polymerase II promoter | 24 | 5.607477 | 0.038857 | TGIF1, PLK3, ZNF296, UHRF1, CBX2, ASCL1, ASCL2, NR0B2, FOXP3, RELB, DLL4, MAD2L2, DRAP1, HEYL, EID2, HEY1, PCBP3, SOX18, SALL4, E2F1, TIMELESS, TRIB3, INSM1, FOXA2 | 349 | 748 | 16792 | 1.543784 | 1 | 0.633974 | 0.622881 |
| GOTERM_BP_ALL | GO:0007389~pattern specification process | 16 | 3.738318 | 0.038896 | EMX1, MESP1, PRKDC, LRP5, ASCL1, HOXC10, AURKA, DLL3, DLL4, ADGRG1, HOXB9, SOX18, HOXB2, HOXC9, FOXA2, HOXC6 | 349 | 436 | 16792 | 1.765674 | 1 | 0.633974 | 0.622881 |
| GOTERM_BP_ALL | GO:1901701~cellular response to oxygen-containing compound | 28 | 6.542056 | 0.0396 | SPON2, SCARB1, CEBPB, PRKDC, SERPINE1, AACS, NME1-NME2, CST3, TRPM2, P2RY6, SOCS1, GNG4, UCP2, E2F1, FFAR2, CHRNB2, ATP6V0B, PTPRN2, DGAT2, ZNF580, KCNJ11, TESC, AGT, RFX6, NEURL1, TIMELESS, DPEP1, TRIB3 | 349 | 912 | 16792 | 1.477203 | 1 | 0.64319 | 0.631935 |
| GOTERM_BP_ALL | GO:0051338~regulation of transferase activity | 29 | 6.775701 | 0.040592 | SHC2, LRP5, DBF4B, PKMYT1, SHB, CDC20, SOCS1, DUSP10, MAP4K2, CDKN2A, UBE2C, TESC, ADRA2C, SRCIN1, AGT, MAPK8IP1, CDC25B, MAD2L2, FABP4, CCNE1, UBE2S, CDK4, NEURL1, MAP3K10, CHI3L1, TRIB3, ANAPC5, CDK5R2, FOXA2 | 349 | 955 | 16792 | 1.461072 | 1 | 0.656569 | 0.64508 |
| GOTERM_BP_ALL | GO:0051899~membrane depolarization | 6 | 1.401869 | 0.040727 | CHRNB2, KCNH2, MYOC, CDKN2A, CCK, CACNA1D | 349 | 91 | 16792 | 3.172392 | 1 | 0.656569 | 0.64508 |
| GOTERM_BP_ALL | GO:0061104~adrenal chromaffin cell differentiation | 2 | 0.46729 | 0.04102 | INSM1, ASCL1 | 349 | 2 | 16792 | 48.11461 | 1 | 0.656569 | 0.64508 |
| GOTERM_BP_ALL | GO:0072413~signal transduction involved in mitotic cell cycle checkpoint | 5 | 1.168224 | 0.041559 | PLK3, PRKDC, E2F1, ARID3A, AURKA | 349 | 63 | 16792 | 3.81862 | 1 | 0.656569 | 0.64508 |
| GOTERM_BP_ALL | GO:1902402~signal transduction involved in mitotic DNA damage checkpoint | 5 | 1.168224 | 0.041559 | PLK3, PRKDC, E2F1, ARID3A, AURKA | 349 | 63 | 16792 | 3.81862 | 1 | 0.656569 | 0.64508 |
| GOTERM_BP_ALL | GO:1902403~signal transduction involved in mitotic DNA integrity checkpoint | 5 | 1.168224 | 0.041559 | PLK3, PRKDC, E2F1, ARID3A, AURKA | 349 | 63 | 16792 | 3.81862 | 1 | 0.656569 | 0.64508 |
| GOTERM_BP_ALL | GO:0072431~signal transduction involved in mitotic G1 DNA damage checkpoint | 5 | 1.168224 | 0.041559 | PLK3, PRKDC, E2F1, ARID3A, AURKA | 349 | 63 | 16792 | 3.81862 | 1 | 0.656569 | 0.64508 |
| GOTERM_BP_ALL | GO:1902400~intracellular signal transduction involved in G1 DNA damage checkpoint | 5 | 1.168224 | 0.041559 | PLK3, PRKDC, E2F1, ARID3A, AURKA | 349 | 63 | 16792 | 3.81862 | 1 | 0.656569 | 0.64508 |
| GOTERM_BP_ALL | GO:0009719~response to endogenous stimulus | 43 | 10.04673 | 0.042381 | CEBPB, PRKDC, LRP5, ATP1A3, SCX, ASCL1, AACS, NME1-NME2, DLL4, TRPM2, P2RY6, SOCS1, PARD6A, HEY1, GNG4, UCP2, ADAMTSL2, E2F1, CYC1, POLR2I, CHRNB2, ATP6V0B, KCNJ11, GDF15, ABCC8, APOA2, GGH, SMAD9, NR0B2, PGF, AGT, HEYL, LOC102724428, SDK1, EID2, VGF, CCNE1, NEURL1, TIMELESS, SIK1, TRIB3, FGFR4, ABCG1 | 349 | 1548 | 16792 | 1.336517 | 1 | 0.663946 | 0.652328 |
| GOTERM_BP_ALL | GO:0007096~regulation of exit from mitosis | 3 | 0.700935 | 0.042437 | NPM2, UBE2C, CDCA5 | 349 | 16 | 16792 | 9.02149 | 1 | 0.663946 | 0.652328 |
| GOTERM_BP_ALL | GO:0007154~cell communication | 147 | 34.34579 | 0.042481 | SCARB1, EXOC3L1, RTKN, CRTC1, PLEKHB1, SERPINE1, NUDT1, SCX, STMN3, TPGS1, CDC20, TMEM145, RPS19, SALL4, TNFRSF4, PPFIA3, EPHB3, IER3, CHRNB2, KCNH2, TPRA1, UNC13A, SERPINF2, BHLHA15, MAPK8IP2, RUNDC3A, FOXP3, PGF, MAPK8IP1, TRAIP, LOC102724428, EID2, CLDN3, HOXB9, RFX6, CCNE1, SIK1, TRIB3, PLPP2, STX1A, LY6E, GNAZ, SHC2, PRKDC, C2CD4A, CACNA1D, C2CD4B, C2CD4C, CPLX2, SEZ6L2, CPLX1, TIMM50, NME1-NME2, VTN, TRPM2, ADGRG1, PCSK1N, SOCS1, ADGRG5, RHPN1, BMF, PLK3, PTPRN2, STARD10, SMAD9, ABCA7, NR0B2, SYT7, TMEM198, ARTN, CDK4, MAP3K10, FGFR4, PCSK1, MESP1, SNAP25, DAGLA, DYRK2, ZC3H3, CELF4, SHB, ASGR1, AACS, RND1, DUSP10, HEY1, ADAMTSL2, DACT2, FFAR2, CA8, GRASP, SLC18A1, TEAD4, PLEKHG4, MAP4K2, ATP6V0B, RPS6KL1, MYOC, ABCC8, GP1BB, SYP, ADRA2C, MTSS1, VGF, CHI3L1, TNFRSF25, CEBPB, RGS16, LRP5, ATP1A3, GDPD5, ASCL1, CRIP1, RELB, AURKA, DLL3, DLL4, P2RY6, PARD6A, NPW, GNG4, RAB26, UCP2, E2F1, PRAME, POLR2I, CTHRC1, OR51E1, KCNJ11, CDKN2A, GDF15, GAD1, CADPS, STAC3, ARID3A, CCK, AGT, MAD2L2, HEYL, NFASC, RAB15, TSPAN18, SMOC1, SHKBP1, NEURL1, KCNK3, FOXA2 | 349 | 6275 | 16792 | 1.127147 | 1 | 0.663946 | 0.652328 |
| GOTERM_BP_ALL | GO:0007610~behavior | 19 | 4.439252 | 0.042599 | CHRNB2, SNAP25, CEBPB, CRTC1, FOXO6, ATP1A3, ABCA7, CCK, SEZ6L2, TPGS1, MAPK8IP2, CLN6, AGT, CST3, SDK1, NPW, MAFG, VWA1, FOXA2 | 349 | 557 | 16792 | 1.641253 | 1 | 0.663946 | 0.652328 |
| GOTERM_BP_ALL | GO:0048706~embryonic skeletal system development | 7 | 1.635514 | 0.042965 | HOXB9, MTHFD1L, SCX, HOXB2, HOXC9, SLC39A3, HOXC6 | 349 | 123 | 16792 | 2.73823 | 1 | 0.666879 | 0.655209 |
| GOTERM_BP_ALL | GO:2000026~regulation of multicellular organismal development | 47 | 10.98131 | 0.043327 | REG3A, SNAP25, MESP1, CEBPB, CRTC1, SERPINE1, CELF4, FOXO6, SCX, GDPD5, ASCL1, SHB, AQP3, ASCL2, DLL3, NME1-NME2, CDC20, DLL4, ADGRG1, DUSP10, PARD6A, HEY1, PHLDA2, EPHB3, CTHRC1, CHRNB2, EMX1, LRRC24, UNC13A, CDKN2A, IL34, TESC, TMEM176B, TMEM176A, ADRA2C, ETV4, SRCIN1, FOXP3, PGF, AGT, MAD2L2, HEYL, SDK1, MAFG, NEURL1, CHI3L1, FOXA2 | 349 | 1721 | 16792 | 1.313996 | 1 | 0.666879 | 0.655209 |
| GOTERM_BP_ALL | GO:0010033~response to organic substance | 72 | 16.82243 | 0.043369 | SPON2, SCARB1, SERPINE1, SCX, AQP3, AACS, TMEM145, DUSP10, HEY1, ADAMTSL2, FFAR2, TNFRSF4, TGIF1, CHRNB2, ATP6V0B, DGAT2, ABCC8, TESC, SERPINF2, APOA2, BHLHA15, SYP, PGF, TRAIP, LOC102724428, EID2, VGF, RFX6, CCNE1, TIMELESS, CHI3L1, TNFRSF25, TRIB3, SIK1, ABCG1, MCM2, CEBPB, SHMT2, PRKDC, LRP5, ATP1A3, ASCL1, CRIP1, RELB, NME1-NME2, DLL4, CST3, VTN, TRPM2, P2RY6, PARD6A, SOCS1, GNG4, UCP2, E2F1, CYC1, POLR2I, PTPRN2, KCNJ11, GDF15, GGH, SMAD9, NR0B2, AGT, HEYL, SDK1, FABP4, CDK4, NEURL1, SP5, FGFR4, FOXA2 | 349 | 2822 | 16792 | 1.227588 | 1 | 0.666879 | 0.655209 |
| GOTERM_BP_ALL | GO:0032371~regulation of sterol transport | 4 | 0.934579 | 0.043652 | APOC1, APOA2, ABCA7, ABCG1 | 349 | 38 | 16792 | 5.064696 | 1 | 0.666879 | 0.655209 |
| GOTERM_BP_ALL | GO:0061098~positive regulation of protein tyrosine kinase activity | 4 | 0.934579 | 0.043652 | NEURL1, ADRA2C, SRCIN1, AGT | 349 | 38 | 16792 | 5.064696 | 1 | 0.666879 | 0.655209 |
| GOTERM_BP_ALL | GO:0032374~regulation of cholesterol transport | 4 | 0.934579 | 0.043652 | APOC1, APOA2, ABCA7, ABCG1 | 349 | 38 | 16792 | 5.064696 | 1 | 0.666879 | 0.655209 |
| GOTERM_BP_ALL | GO:0016050~vesicle organization | 12 | 2.803738 | 0.044168 | SNAP25, SERPINA1, UNC13A, CADPS, C2CD4A, C2CD4B, TSNARE1, C2CD4C, MYO7A, SYP, STX1A, SYT7 | 349 | 295 | 16792 | 1.957205 | 1 | 0.671642 | 0.659889 |
| GOTERM_BP_ALL | GO:0055088~lipid homeostasis | 7 | 1.635514 | 0.044399 | SCARB1, FABP4, DGAT2, POLD1, APOA2, LRP5, ABCG1 | 349 | 124 | 16792 | 2.716148 | 1 | 0.671642 | 0.659889 |
| GOTERM_BP_ALL | GO:0050773~regulation of dendrite development | 7 | 1.635514 | 0.044399 | CHRNB2, CDC20, SDK1, CRTC1, NEURL1, FOXO6, SRCIN1 | 349 | 124 | 16792 | 2.716148 | 1 | 0.671642 | 0.659889 |
| GOTERM_BP_ALL | GO:0045892~negative regulation of transcription, DNA-templated | 33 | 7.71028 | 0.045 | MESP1, CEBPB, ZNF296, UHRF1, SCX, ASCL1, ASCL2, RELB, DLL4, PCBP3, HEY1, SOX18, SALL4, E2F1, PRAME, TNFRSF4, TGIF1, PLK3, CDKN2A, CBX2, H2AFX, NR0B2, FOXP3, MAD2L2, DRAP1, HEYL, EID2, FABP4, TIMELESS, MAP3K10, TRIB3, INSM1, FOXA2 | 349 | 1130 | 16792 | 1.405117 | 1 | 0.677099 | 0.665251 |
| GOTERM_BP_ALL | GO:0010557~positive regulation of macromolecule biosynthetic process | 44 | 10.28037 | 0.045052 | MESP1, CEBPB, BEX1, DYRK2, UHRF1, CRTC1, PRKDC, SERPINE1, LRP5, SCX, DBF4B, ASCL1, RELB, NME1-NME2, CST3, HEY1, SOX18, SALL4, ZXDC, E2F1, MYBL2, POLR2I, NPM2, CDKN2A, TESC, SERPINF2, APOA2, SMAD9, ARID3A, SOX12, BHLHA15, ETV4, FOXP3, AGT, MAD2L2, HEYL, HOXB9, RFX6, CCNE1, CDK4, MAFG, FGFR4, RNF187, FOXA2 | 349 | 1597 | 16792 | 1.325637 | 1 | 0.677099 | 0.665251 |
| GOTERM_BP_ALL | GO:0072422~signal transduction involved in DNA damage checkpoint | 5 | 1.168224 | 0.045766 | PLK3, PRKDC, E2F1, ARID3A, AURKA | 349 | 65 | 16792 | 3.701124 | 1 | 0.683386 | 0.671428 |
| GOTERM_BP_ALL | GO:0072401~signal transduction involved in DNA integrity checkpoint | 5 | 1.168224 | 0.045766 | PLK3, PRKDC, E2F1, ARID3A, AURKA | 349 | 65 | 16792 | 3.701124 | 1 | 0.683386 | 0.671428 |
| GOTERM_BP_ALL | GO:0048791~calcium ion-regulated exocytosis of neurotransmitter | 4 | 0.934579 | 0.046588 | C2CD4A, C2CD4B, C2CD4C, SYT7 | 349 | 39 | 16792 | 4.934832 | 1 | 0.688992 | 0.676936 |
| GOTERM_BP_ALL | GO:0046461~neutral lipid catabolic process | 4 | 0.934579 | 0.046588 | LIPE, FABP4, DAGLA, APOA2 | 349 | 39 | 16792 | 4.934832 | 1 | 0.688992 | 0.676936 |
| GOTERM_BP_ALL | GO:0046464~acylglycerol catabolic process | 4 | 0.934579 | 0.046588 | LIPE, FABP4, DAGLA, APOA2 | 349 | 39 | 16792 | 4.934832 | 1 | 0.688992 | 0.676936 |
| GOTERM_BP_ALL | GO:0015672~monovalent inorganic cation transport | 17 | 3.971963 | 0.04686 | KCNH2, ATP6V0B, KCNH6, KCNJ11, ABCC8, TESC, ATP1A3, CACNA1D, SLC38A11, KCNAB2, COX6A1, AGT, TRPM2, FXYD2, UCP2, CYC1, KCNK3 | 349 | 486 | 16792 | 1.683021 | 1 | 0.690813 | 0.678725 |
| GOTERM_BP_ALL | GO:0065007~biological regulation | 255 | 59.57944 | 0.047394 | SPON2, ZNF296, HDAC11, PLEKHB1, SERPINE1, TONSL, SCX, STMN3, AQP3, SCAMP5, CDC20, RINL, RPS19, SOX18, ZXDC, MNX1, EPHB3, IER3, UNC13A, NUP210, SERPINF2, SOX12, BHLHA15, MAPK8IP2, MAPK8IP1, EID2, HOXB9, CLDN3, RFX6, ORAI1, SIK1, HOXB2, TRIB3, PLPP2, STX1A, BEX2, TPH1, BEX1, SHMT2, PRKDC, PPP1R35, CDCA5, C2CD4A, C2CD4B, C2CD4C, C2, TRPM2, PCBP3, DHX34, MIER2, HOXC9, HOXC6, RTEL1-TNFRSF6B, CDT1, PLK3, PTPRN2, STARD10, ABCA7, NR0B2, ST18, TMEM198, PARP10, ARTN, PAH, FXYD2, PCSK1, MESP1, SERPINA1, GPAA1, SERPINA10, SHB, HOXC10, WFDC2, AACS, HEY1, ADAMTSL2, FFAR2, TEAD4, MAP4K2, ATP6V0B, RPS6KL1, MYOC, GP1BB, TESC, SYP, MTSS1, MMP11, KIF2C, GDPD5, ASCL1, CRIP1, ASCL2, RELB, CST3, CST1, NPW, UCP2, PRAME, DTD1, LRRC24, NPM2, ZNF580, CDKN2A, GDF15, IL34, GAD1, TMEM176B, TMEM176A, STAC3, AGT, GUCA2A, MAD2L2, HEYL, FABP4, SMOC1, SHKBP1, TACC3, RIMBP2, CDK5R2, FOXA2, EXOC3L1, SCARB1, RTKN, CRTC1, REG1A, COX6A1, TMEM145, PTTG1, SALL4, PHLDA2, TNFRSF4, PPFIA3, SLC39A3, CHRNB2, SPINK4, KCNH2, TPRA1, DGAT2, KCNH6, LIG1, KMT5C, ATP11A, PGC, RUNDC3A, FOXP3, SRCIN1, PGF, TRAIP, CDC25B, LOC102724428, CCNE1, TIMELESS, DPEP1, ANAPC5, RELL2, ABCG1, LY6E, GNAZ, REG3A, SHC2, DPT, FOXO6, CACNA1D, CPLX2, SEZ6L2, PKMYT1, CPLX1, TIMM50, NME1-NME2, VTN, ADGRG1, PCSK1N, SOCS1, ADGRG5, RHPN1, BMF, UBE2C, CBX2, ASPSCR1, CYBRD1, SMAD9, SYT7, DRAP1, SDK1, KIF18B, UBE2S, CDK4, MAFG, MAP3K10, SP5, MELTF, FGFR4, RNF187, SNAP25, DAGLA, DYRK2, ZC3H3, CELF4, RND1, DUSP10, SPR, DACT2, MYBL2, CA8, GRASP, TGIF1, PLEKHG4, EMX1, ABCC8, MMP1, H2AFX, APOA2, KCNAB2, ADRA2C, ETV4, VGF, CHI3L1, TNFRSF25, PFDN2, CEBPB, UHRF1, RGS16, LRP5, ATP1A3, DBF4B, CLN6, AURKA, DLL3, DLL4, P2RY6, TTR, PARD6A, PUF60, GNG4, RAB26, POLD1, E2F1, POLR2I, CTHRC1, HES4, OR51E1, PPP1R14B, PPP1R14C, KCNJ11, CADPS, ARID3A, CCK, FAM155B, PRC1, RAB15, TSPAN18, APOC1, NEURL1, INSM1, KCNK3 | 349 | 11548 | 16792 | 1.062455 | 1 | 0.695093 | 0.68293 |
| GOTERM_BP_ALL | GO:0014061~regulation of norepinephrine secretion | 3 | 0.700935 | 0.047451 | ADRA2C, STX1A, AGT | 349 | 17 | 16792 | 8.490814 | 1 | 0.695093 | 0.68293 |
| GOTERM_BP_ALL | GO:0032637~interleukin-8 production | 5 | 1.168224 | 0.047955 | ZNF580, SERPINE1, APOA2, CHI3L1, FFAR2 | 349 | 66 | 16792 | 3.645046 | 1 | 0.698065 | 0.68585 |
| GOTERM_BP_ALL | GO:0072395~signal transduction involved in cell cycle checkpoint | 5 | 1.168224 | 0.047955 | PLK3, PRKDC, E2F1, ARID3A, AURKA | 349 | 66 | 16792 | 3.645046 | 1 | 0.698065 | 0.68585 |
| GOTERM_BP_ALL | GO:0051224~negative regulation of protein transport | 9 | 2.102804 | 0.048226 | PARP10, DYRK2, KCNJ11, ABCC8, UCP2, APOA2, TONSL, SRCIN1, FOXP3 | 349 | 193 | 16792 | 2.243687 | 1 | 0.699811 | 0.687566 |
| GOTERM_BP_ALL | GO:0044700~single organism signaling | 145 | 33.8785 | 0.048385 | SCARB1, EXOC3L1, RTKN, CRTC1, PLEKHB1, SERPINE1, SCX, STMN3, TPGS1, CDC20, TMEM145, RPS19, SALL4, TNFRSF4, PPFIA3, EPHB3, IER3, CHRNB2, KCNH2, TPRA1, UNC13A, SERPINF2, BHLHA15, MAPK8IP2, RUNDC3A, FOXP3, PGF, MAPK8IP1, TRAIP, LOC102724428, EID2, CLDN3, HOXB9, RFX6, CCNE1, SIK1, TRIB3, PLPP2, STX1A, LY6E, GNAZ, SHC2, PRKDC, C2CD4A, CACNA1D, C2CD4B, C2CD4C, CPLX2, SEZ6L2, CPLX1, TIMM50, NME1-NME2, VTN, TRPM2, ADGRG1, PCSK1N, SOCS1, ADGRG5, RHPN1, BMF, PLK3, PTPRN2, STARD10, SMAD9, ABCA7, NR0B2, SYT7, TMEM198, ARTN, CDK4, MAP3K10, FGFR4, PCSK1, MESP1, SNAP25, DAGLA, DYRK2, ZC3H3, CELF4, SHB, AACS, RND1, DUSP10, HEY1, ADAMTSL2, DACT2, FFAR2, CA8, GRASP, SLC18A1, TEAD4, PLEKHG4, MAP4K2, ATP6V0B, RPS6KL1, MYOC, ABCC8, GP1BB, SYP, ADRA2C, MTSS1, VGF, CHI3L1, TNFRSF25, CEBPB, RGS16, LRP5, ATP1A3, GDPD5, ASCL1, CRIP1, RELB, AURKA, DLL3, DLL4, P2RY6, PARD6A, NPW, GNG4, RAB26, UCP2, E2F1, PRAME, POLR2I, CTHRC1, OR51E1, KCNJ11, CDKN2A, GDF15, GAD1, CADPS, STAC3, ARID3A, CCK, AGT, MAD2L2, HEYL, NFASC, RAB15, TSPAN18, SMOC1, SHKBP1, NEURL1, KCNK3, FOXA2 | 349 | 6207 | 16792 | 1.123992 | 1 | 0.699924 | 0.687677 |
| GOTERM_BP_ALL | GO:0019432~triglyceride biosynthetic process | 4 | 0.934579 | 0.049619 | LOC102724428, SCARB1, DGAT2, SIK1 | 349 | 40 | 16792 | 4.811461 | 1 | 0.715346 | 0.702828 |
| GOTERM_BP_ALL | GO:0023052~signaling | 146 | 34.11215 | 0.04976 | SCARB1, EXOC3L1, RTKN, CRTC1, PLEKHB1, SERPINE1, SCX, STMN3, TPGS1, CDC20, TMEM145, RPS19, SALL4, TNFRSF4, PPFIA3, EPHB3, IER3, CHRNB2, KCNH2, TPRA1, UNC13A, SERPINF2, BHLHA15, MAPK8IP2, RUNDC3A, FOXP3, PGF, MAPK8IP1, TRAIP, LOC102724428, EID2, CLDN3, HOXB9, RFX6, CCNE1, SIK1, TRIB3, PLPP2, STX1A, LY6E, GNAZ, SHC2, PRKDC, C2CD4A, CACNA1D, C2CD4B, C2CD4C, CPLX2, SEZ6L2, CPLX1, TIMM50, NME1-NME2, VTN, TRPM2, ADGRG1, PCSK1N, SOCS1, ADGRG5, RHPN1, BMF, PLK3, PTPRN2, STARD10, SMAD9, ABCA7, NR0B2, SYT7, TMEM198, ARTN, CDK4, FXYD2, MAP3K10, FGFR4, PCSK1, MESP1, SNAP25, DAGLA, DYRK2, ZC3H3, CELF4, SHB, AACS, RND1, DUSP10, HEY1, ADAMTSL2, DACT2, FFAR2, CA8, GRASP, SLC18A1, TEAD4, PLEKHG4, MAP4K2, ATP6V0B, RPS6KL1, MYOC, ABCC8, GP1BB, SYP, ADRA2C, MTSS1, VGF, CHI3L1, TNFRSF25, CEBPB, RGS16, LRP5, ATP1A3, GDPD5, ASCL1, CRIP1, RELB, AURKA, DLL3, DLL4, P2RY6, PARD6A, NPW, GNG4, RAB26, UCP2, E2F1, PRAME, POLR2I, CTHRC1, OR51E1, KCNJ11, CDKN2A, GDF15, GAD1, CADPS, STAC3, ARID3A, CCK, AGT, MAD2L2, HEYL, NFASC, RAB15, TSPAN18, SMOC1, SHKBP1, NEURL1, KCNK3, FOXA2 | 349 | 6260 | 16792 | 1.122162 | 1 | 0.715346 | 0.702828 |
| GOTERM_BP_ALL | GO:0023051~regulation of signaling | 77 | 17.99065 | 0.050314 | SNAP25, MESP1, DYRK2, CRTC1, ZC3H3, SERPINE1, CELF4, STMN3, SHB, AACS, CDC20, DUSP10, HEY1, ADAMTSL2, DACT2, FFAR2, TNFRSF4, PPFIA3, IER3, CHRNB2, PLEKHG4, MAP4K2, UNC13A, MYOC, ABCC8, SERPINF2, SYP, ADRA2C, MAPK8IP2, MAPK8IP1, TRAIP, EID2, VGF, RFX6, CHI3L1, TNFRSF25, TRIB3, STX1A, SHC2, RGS16, LRP5, ATP1A3, CACNA1D, GDPD5, ASCL1, CPLX2, SEZ6L2, AURKA, DLL4, VTN, TRPM2, ADGRG1, SOCS1, GNG4, UCP2, E2F1, BMF, PRAME, CTHRC1, PLK3, KCNJ11, CDKN2A, GDF15, STARD10, ABCA7, NR0B2, AGT, SYT7, TMEM198, MAD2L2, HEYL, FXYD2, SHKBP1, NEURL1, MAP3K10, FGFR4, FOXA2 | 349 | 3070 | 16792 | 1.206783 | 1 | 0.721063 | 0.708446 |
| GOTERM_BP_ALL | GO:0030182~neuron differentiation | 35 | 8.17757 | 0.050646 | SNAP25, SPON2, CEBPB, CRTC1, BICDL1, FOXO6, STMN3, GDPD5, ASCL1, HOXC10, AURKA, RND1, NME1-NME2, CDC20, DLL4, MNX1, EPHB3, CTHRC1, CHRNB2, EMX1, UNC13A, MYOC, CCK, MYO7A, ADRA2C, MAPK8IP2, ETV4, SRCIN1, HEYL, SDK1, NFASC, ARTN, NEURL1, INSM1, FOXA2 | 349 | 1226 | 16792 | 1.373582 | 1 | 0.723588 | 0.710927 |
| GOTERM_BP_ALL | GO:0007613~memory | 6 | 1.401869 | 0.051163 | CHRNB2, CEBPB, CRTC1, FOXO6, ATP1A3, ABCA7 | 349 | 97 | 16792 | 2.976162 | 1 | 0.728721 | 0.71597 |
| GOTERM_BP_ALL | GO:0019318~hexose metabolic process | 10 | 2.336449 | 0.05191 | LOC102724428, SDS, G6PC3, DGAT2, DYRK2, KCNJ11, LRP5, SIK1, PHLDA2, FOXA2 | 349 | 231 | 16792 | 2.082884 | 1 | 0.734039 | 0.721194 |
| GOTERM_BP_ALL | GO:0051241~negative regulation of multicellular organismal process | 30 | 7.009346 | 0.051985 | REG3A, MESP1, TPH1, SERPINE1, LRP5, ASCL1, SHB, ASCL2, RELB, DLL3, NME1-NME2, DLL4, CST3, VTN, ADGRG1, DUSP10, RPS19, CDKN2A, TMEM176B, TMEM176A, SERPINF2, APOA2, ADRA2C, ETV4, FOXP3, AGT, TRAIP, MAD2L2, APOC1, FOXA2 | 349 | 1017 | 16792 | 1.41931 | 1 | 0.734039 | 0.721194 |
| GOTERM_BP_ALL | GO:0061351~neural precursor cell proliferation | 7 | 1.635514 | 0.052012 | DLL4, EMX1, ADGRG1, DAGLA, ARTN, INSM1, ASCL1 | 349 | 129 | 16792 | 2.61087 | 1 | 0.734039 | 0.721194 |
| GOTERM_BP_ALL | GO:0015837~amine transport | 5 | 1.168224 | 0.052505 | CHRNB2, ADRA2C, SLC18A1, STX1A, AGT | 349 | 68 | 16792 | 3.537839 | 1 | 0.735388 | 0.72252 |
| GOTERM_BP_ALL | GO:0043691~reverse cholesterol transport | 3 | 0.700935 | 0.052668 | SCARB1, APOA2, ABCG1 | 349 | 18 | 16792 | 8.019102 | 1 | 0.735388 | 0.72252 |
| GOTERM_BP_ALL | GO:0050433~regulation of catecholamine secretion | 4 | 0.934579 | 0.052743 | CHRNB2, ADRA2C, STX1A, AGT | 349 | 41 | 16792 | 4.694109 | 1 | 0.735388 | 0.72252 |
| GOTERM_BP_ALL | GO:0097028~dendritic cell differentiation | 4 | 0.934579 | 0.052743 | TRPM2, TMEM176B, TMEM176A, RELB | 349 | 41 | 16792 | 4.694109 | 1 | 0.735388 | 0.72252 |
| GOTERM_BP_ALL | GO:0071333~cellular response to glucose stimulus | 6 | 1.401869 | 0.053041 | NME1-NME2, PTPRN2, RFX6, KCNJ11, UCP2, AACS | 349 | 98 | 16792 | 2.945793 | 1 | 0.737319 | 0.724417 |
| GOTERM_BP_ALL | GO:0021510~spinal cord development | 6 | 1.401869 | 0.054959 | DLL4, SOX12, GDPD5, ASCL1, MNX1, HOXC10 | 349 | 99 | 16792 | 2.916037 | 1 | 0.761688 | 0.748359 |
| GOTERM_BP_ALL | GO:0051093~negative regulation of developmental process | 23 | 5.373832 | 0.055685 | REG3A, MESP1, CDKN2A, PRKDC, TMEM176B, TMEM176A, SERPINE1, BHLHA15, ASCL1, SHB, ETV4, ASCL2, FOXP3, AGT, DLL3, DLL4, NME1-NME2, MAD2L2, CST3, ADGRG1, DUSP10, MELTF, FOXA2 | 349 | 739 | 16792 | 1.497478 | 1 | 0.768669 | 0.755219 |
| GOTERM_BP_ALL | GO:0046463~acylglycerol biosynthetic process | 4 | 0.934579 | 0.055961 | LOC102724428, SCARB1, DGAT2, SIK1 | 349 | 42 | 16792 | 4.582344 | 1 | 0.768669 | 0.755219 |
| GOTERM_BP_ALL | GO:0046460~neutral lipid biosynthetic process | 4 | 0.934579 | 0.055961 | LOC102724428, SCARB1, DGAT2, SIK1 | 349 | 42 | 16792 | 4.582344 | 1 | 0.768669 | 0.755219 |
| GOTERM_BP_ALL | GO:0031400~negative regulation of protein modification process | 19 | 4.439252 | 0.05688 | CDKN2A, PRKDC, UBE2C, TESC, LRP5, SHB, SRCIN1, FOXP3, MAPK8IP1, PARP10, MAD2L2, CDC20, PARD6A, SOCS1, DUSP10, FABP4, TRIB3, ANAPC5, INSM1 | 349 | 578 | 16792 | 1.581622 | 1 | 0.776998 | 0.763402 |
| GOTERM_BP_ALL | GO:0030879~mammary gland development | 7 | 1.635514 | 0.05694 | CEBPB, TPH1, HOXB9, NEURL1, LRP5, ORAI1, ETV4 | 349 | 132 | 16792 | 2.551533 | 1 | 0.776998 | 0.763402 |
| GOTERM_BP_ALL | GO:0022406~membrane docking | 5 | 1.168224 | 0.057282 | SNAP25, UNC13A, TSNARE1, CPLX2, STX1A | 349 | 70 | 16792 | 3.436758 | 1 | 0.776998 | 0.763402 |
| GOTERM_BP_ALL | GO:0060562~epithelial tube morphogenesis | 12 | 2.803738 | 0.057861 | DLL4, MESP1, MTHFD1L, SOX18, SALL4, TIMELESS, LRP5, ETV4, AGT, PGF, MTSS1, CTHRC1 | 349 | 309 | 16792 | 1.868529 | 1 | 0.776998 | 0.763402 |
| GOTERM_BP_ALL | GO:0060074~synapse maturation | 3 | 0.700935 | 0.058078 | CDC20, NEURL1, SEZ6L2 | 349 | 19 | 16792 | 7.597044 | 1 | 0.776998 | 0.763402 |
| GOTERM_BP_ALL | GO:0031639~plasminogen activation | 3 | 0.700935 | 0.058078 | SERPINF2, SERPINE1, MELTF | 349 | 19 | 16792 | 7.597044 | 1 | 0.776998 | 0.763402 |
| GOTERM_BP_ALL | GO:0051882~mitochondrial depolarization | 3 | 0.700935 | 0.058078 | MYOC, CDKN2A, CCK | 349 | 19 | 16792 | 7.597044 | 1 | 0.776998 | 0.763402 |
| GOTERM_BP_ALL | GO:0010458~exit from mitosis | 3 | 0.700935 | 0.058078 | UBE2C, UBE2S, CDCA5 | 349 | 19 | 16792 | 7.597044 | 1 | 0.776998 | 0.763402 |
| GOTERM_BP_ALL | GO:0034377~plasma lipoprotein particle assembly | 3 | 0.700935 | 0.058078 | APOC1, APOA2, ABCA7 | 349 | 19 | 16792 | 7.597044 | 1 | 0.776998 | 0.763402 |
| GOTERM_BP_ALL | GO:0071326~cellular response to monosaccharide stimulus | 6 | 1.401869 | 0.058913 | NME1-NME2, PTPRN2, RFX6, KCNJ11, UCP2, AACS | 349 | 101 | 16792 | 2.858294 | 1 | 0.781397 | 0.767724 |
| GOTERM_BP_ALL | GO:0042752~regulation of circadian rhythm | 6 | 1.401869 | 0.058913 | CHRNB2, LOC102724428, PRKDC, CRTC1, TIMELESS, SIK1 | 349 | 101 | 16792 | 2.858294 | 1 | 0.781397 | 0.767724 |
| GOTERM_BP_ALL | GO:0071331~cellular response to hexose stimulus | 6 | 1.401869 | 0.058913 | NME1-NME2, PTPRN2, RFX6, KCNJ11, UCP2, AACS | 349 | 101 | 16792 | 2.858294 | 1 | 0.781397 | 0.767724 |
| GOTERM_BP_ALL | GO:0006811~ion transport | 40 | 9.345794 | 0.05925 | SNAP25, TTYH3, TMEM63C, ATP1A3, CACNA1D, SLC38A11, SLC1A5, COX6A1, SLC7A1, AQP3, CPLX1, TRPM2, P2RY6, UCP2, CYC1, SLC25A22, PPFIA3, SLC39A3, KCNH2, CHRNB2, ATP6V0B, KCNH6, KCNJ11, ABCC8, TESC, STARD10, CCK, KCNAB2, BHLHA15, MAPK8IP2, AGT, FAM155B, LOC102724428, SLCO3A1, FXYD2, ORAI1, SIK1, MELTF, KCNK3, STX1A | 349 | 1458 | 16792 | 1.320017 | 1 | 0.783628 | 0.769916 |
| GOTERM_BP_ALL | GO:0035239~tube morphogenesis | 13 | 3.037383 | 0.059586 | MESP1, LRP5, ETV4, PGF, AGT, MTSS1, DLL4, MTHFD1L, SOX18, SALL4, TIMELESS, EPHB3, CTHRC1 | 349 | 348 | 16792 | 1.797385 | 1 | 0.785814 | 0.772064 |
| GOTERM_BP_ALL | GO:0098657~import into cell | 5 | 1.168224 | 0.059755 | FAM155B, SNAP25, TRPM2, FXYD2, AGT | 349 | 71 | 16792 | 3.388353 | 1 | 0.785814 | 0.772064 |
| GOTERM_BP_ALL | GO:0003358~noradrenergic neuron development | 2 | 0.46729 | 0.060896 | INSM1, ASCL1 | 349 | 3 | 16792 | 32.07641 | 1 | 0.786844 | 0.773076 |
| GOTERM_BP_ALL | GO:0060266~negative regulation of respiratory burst involved in inflammatory response | 2 | 0.46729 | 0.060896 | DUSP10, RPS19 | 349 | 3 | 16792 | 32.07641 | 1 | 0.786844 | 0.773076 |
| GOTERM_BP_ALL | GO:0010868~negative regulation of triglyceride biosynthetic process | 2 | 0.46729 | 0.060896 | LOC102724428, SIK1 | 349 | 3 | 16792 | 32.07641 | 1 | 0.786844 | 0.773076 |
| GOTERM_BP_ALL | GO:0060268~negative regulation of respiratory burst | 2 | 0.46729 | 0.060896 | DUSP10, RPS19 | 349 | 3 | 16792 | 32.07641 | 1 | 0.786844 | 0.773076 |
| GOTERM_BP_ALL | GO:0010899~regulation of phosphatidylcholine catabolic process | 2 | 0.46729 | 0.060896 | SCARB1, APOC1 | 349 | 3 | 16792 | 32.07641 | 1 | 0.786844 | 0.773076 |
| GOTERM_BP_ALL | GO:0031099~regeneration | 8 | 1.869159 | 0.060924 | VTN, CEBPB, CDK4, UCP2, APOA2, NR0B2, PGF, AURKA | 349 | 168 | 16792 | 2.291172 | 1 | 0.786844 | 0.773076 |
| GOTERM_BP_ALL | GO:0009790~embryo development | 28 | 6.542056 | 0.061043 | MESP1, CEBPB, PRKDC, LRP5, CELF4, SCX, ASCL2, HOXC10, DLL3, DLL4, VTN, HEY1, MTHFD1L, SOX18, SALL4, HOXC9, CTHRC1, HOXC6, SLC39A3, EMX1, TPRA1, NPM2, MYO7A, HOXB9, MAFG, MFAP2, HOXB2, FOXA2 | 349 | 954 | 16792 | 1.412169 | 1 | 0.786844 | 0.773076 |
| GOTERM_BP_ALL | GO:0010563~negative regulation of phosphorus metabolic process | 18 | 4.205607 | 0.061363 | CDKN2A, PRKDC, UBE2C, TESC, PPP1R35, LRP5, PKMYT1, SHB, SRCIN1, MAPK8IP1, PARD6A, SOCS1, DUSP10, FABP4, RIMBP2, TRIB3, INSM1, FOXA2 | 349 | 544 | 16792 | 1.592028 | 1 | 0.786844 | 0.773076 |
| GOTERM_BP_ALL | GO:0045936~negative regulation of phosphate metabolic process | 18 | 4.205607 | 0.061363 | CDKN2A, PRKDC, UBE2C, TESC, PPP1R35, LRP5, PKMYT1, SHB, SRCIN1, MAPK8IP1, PARD6A, SOCS1, DUSP10, FABP4, RIMBP2, TRIB3, INSM1, FOXA2 | 349 | 544 | 16792 | 1.592028 | 1 | 0.786844 | 0.773076 |
| GOTERM_BP_ALL | GO:0051173~positive regulation of nitrogen compound metabolic process | 47 | 10.98131 | 0.061642 | MESP1, CEBPB, BEX1, UHRF1, CRTC1, PRKDC, SERPINE1, LRP5, CELF4, SCX, DBF4B, ASCL1, RELB, NME1-NME2, CST3, HEY1, SOX18, SALL4, ZXDC, E2F1, MYBL2, POLR2I, NPM2, CDKN2A, TESC, H2AFX, SERPINF2, SMAD9, ARID3A, SOX12, BHLHA15, ETV4, RUNDC3A, FOXP3, GUCA2A, AGT, MAD2L2, HEYL, HOXB9, RFX6, CCNE1, CDK4, MAFG, TIMELESS, FGFR4, RNF187, FOXA2 | 349 | 1767 | 16792 | 1.279789 | 1 | 0.788235 | 0.774442 |
| GOTERM_BP_ALL | GO:0042326~negative regulation of phosphorylation | 15 | 3.504673 | 0.062204 | CDKN2A, PRKDC, UBE2C, TESC, LRP5, SHB, SRCIN1, MAPK8IP1, PARD6A, SOCS1, DUSP10, FABP4, TRIB3, INSM1, FOXA2 | 349 | 428 | 16792 | 1.68626 | 1 | 0.788284 | 0.77449 |
| GOTERM_BP_ALL | GO:0031571~mitotic G1 DNA damage checkpoint | 5 | 1.168224 | 0.062284 | PLK3, PRKDC, E2F1, ARID3A, AURKA | 349 | 72 | 16792 | 3.341293 | 1 | 0.788284 | 0.77449 |
| GOTERM_BP_ALL | GO:0044819~mitotic G1/S transition checkpoint | 5 | 1.168224 | 0.062284 | PLK3, PRKDC, E2F1, ARID3A, AURKA | 349 | 72 | 16792 | 3.341293 | 1 | 0.788284 | 0.77449 |
| GOTERM_BP_ALL | GO:1904950~negative regulation of establishment of protein localization | 9 | 2.102804 | 0.06264 | PARP10, DYRK2, KCNJ11, ABCC8, UCP2, APOA2, TONSL, SRCIN1, FOXP3 | 349 | 204 | 16792 | 2.122704 | 1 | 0.788284 | 0.77449 |
| GOTERM_BP_ALL | GO:2000179~positive regulation of neural precursor cell proliferation | 4 | 0.934579 | 0.062668 | DLL4, ADGRG1, INSM1, ASCL1 | 349 | 44 | 16792 | 4.374056 | 1 | 0.788284 | 0.77449 |
| GOTERM_BP_ALL | GO:0032757~positive regulation of interleukin-8 production | 4 | 0.934579 | 0.062668 | ZNF580, SERPINE1, APOA2, FFAR2 | 349 | 44 | 16792 | 4.374056 | 1 | 0.788284 | 0.77449 |
| GOTERM_BP_ALL | GO:0010906~regulation of glucose metabolic process | 6 | 1.401869 | 0.063025 | LOC102724428, DGAT2, DYRK2, SIK1, PHLDA2, FOXA2 | 349 | 103 | 16792 | 2.802793 | 1 | 0.790637 | 0.776802 |
| GOTERM_BP_ALL | GO:0043153~entrainment of circadian clock by photoperiod | 3 | 0.700935 | 0.06367 | LOC102724428, CRTC1, SIK1 | 349 | 20 | 16792 | 7.217192 | 1 | 0.796492 | 0.782555 |
| GOTERM_BP_ALL | GO:0010646~regulation of cell communication | 75 | 17.52336 | 0.06408 | SNAP25, MESP1, DYRK2, CRTC1, ZC3H3, SERPINE1, CELF4, STMN3, SHB, AACS, CDC20, DUSP10, HEY1, ADAMTSL2, DACT2, FFAR2, TNFRSF4, PPFIA3, IER3, CHRNB2, PLEKHG4, MAP4K2, UNC13A, MYOC, ABCC8, SERPINF2, SYP, ADRA2C, MAPK8IP2, MAPK8IP1, TRAIP, EID2, VGF, RFX6, CHI3L1, TNFRSF25, TRIB3, STX1A, SHC2, RGS16, LRP5, CACNA1D, GDPD5, ASCL1, CPLX2, SEZ6L2, AURKA, DLL4, VTN, TRPM2, ADGRG1, SOCS1, GNG4, UCP2, E2F1, BMF, PRAME, CTHRC1, PLK3, KCNJ11, CDKN2A, GDF15, STARD10, ABCA7, NR0B2, AGT, SYT7, TMEM198, MAD2L2, HEYL, SHKBP1, NEURL1, MAP3K10, FGFR4, FOXA2 | 349 | 3020 | 16792 | 1.194899 | 1 | 0.796492 | 0.782555 |
| GOTERM_BP_ALL | GO:0033554~cellular response to stress | 47 | 10.98131 | 0.064657 | CEBPB, RNASEH2A, DYRK2, CLPB, UHRF1, PRKDC, CDCA5, NUDT1, TONSL, CRIP1, AURKA, RELB, NME1-NME2, SCAMP5, RECQL4, CST3, TRPM2, DUSP10, PTTG1, POLD1, UCP2, E2F1, RAD54L, POLR2I, IER3, GINS2, RTEL1-TNFRSF6B, PLK3, MAP4K2, LIG1, ZNF580, NUP210, CDKN2A, H2AFX, SERPINF2, ARID3A, BHLHA15, MAPK8IP2, AGT, MTSS1, MAPK8IP1, MAD2L2, TIMELESS, MAP3K10, DPEP1, TRIB3, KCNK3 | 349 | 1773 | 16792 | 1.275458 | 1 | 0.796492 | 0.782555 |
| GOTERM_BP_ALL | GO:0045944~positive regulation of transcription from RNA polymerase II promoter | 30 | 7.009346 | 0.064706 | MESP1, CEBPB, BEX1, UHRF1, CRTC1, PRKDC, SERPINE1, LRP5, SCX, ASCL1, RELB, NME1-NME2, HEY1, SOX18, SALL4, E2F1, MYBL2, CDKN2A, SERPINF2, SMAD9, ARID3A, SOX12, BHLHA15, ETV4, FOXP3, HEYL, HOXB9, RFX6, MAFG, FOXA2 | 349 | 1041 | 16792 | 1.386588 | 1 | 0.796492 | 0.782555 |
| GOTERM_BP_ALL | GO:0001101~response to acid chemical | 11 | 2.570093 | 0.064771 | NME1-NME2, CEBPB, SOCS1, TESC, UCP2, NEURL1, E2F1, FFAR2, FGFR4, ASCL1, AQP3 | 349 | 278 | 16792 | 1.903816 | 1 | 0.796492 | 0.782555 |
| GOTERM_BP_ALL | GO:0031323~regulation of cellular metabolic process | 136 | 31.7757 | 0.064776 | SCARB1, ZNF296, CRTC1, HDAC11, SERPINE1, TONSL, SCX, CDC20, PTTG1, SOX18, SALL4, ZXDC, PHLDA2, TNFRSF4, MNX1, CHRNB2, SPINK4, DGAT2, KMT5C, SERPINF2, SOX12, BHLHA15, MAPK8IP2, SRCIN1, RUNDC3A, FOXP3, MAPK8IP1, CDC25B, LOC102724428, EID2, HOXB9, RFX6, CCNE1, TIMELESS, DPEP1, SIK1, HOXB2, TRIB3, ANAPC5, ABCG1, SHC2, BEX1, PRKDC, PPP1R35, FOXO6, PKMYT1, NME1-NME2, VTN, PCSK1N, SOCS1, PCBP3, DHX34, MIER2, HOXC9, HOXC6, RTEL1-TNFRSF6B, CDT1, PLK3, UBE2C, CBX2, SMAD9, ABCA7, NR0B2, ST18, DRAP1, PARP10, UBE2S, CDK4, MAFG, MAP3K10, SP5, MELTF, FGFR4, RNF187, MESP1, SERPINA1, DYRK2, SERPINA10, CELF4, SHB, HOXC10, WFDC2, DUSP10, HEY1, MYBL2, TEAD4, TGIF1, EMX1, MAP4K2, MYOC, TESC, H2AFX, APOA2, ADRA2C, ETV4, CHI3L1, TNFRSF25, CEBPB, UHRF1, LRP5, DBF4B, ASCL1, ASCL2, CLN6, RELB, AURKA, DLL4, CST3, CST1, PARD6A, PUF60, E2F1, PRAME, POLR2I, DTD1, HES4, PPP1R14B, PPP1R14C, NPM2, ZNF580, CDKN2A, GDF15, IL34, ARID3A, CCK, GUCA2A, AGT, MAD2L2, HEYL, FABP4, APOC1, NEURL1, RIMBP2, INSM1, CDK5R2, FOXA2 | 349 | 5844 | 16792 | 1.11971 | 1 | 0.796492 | 0.782555 |
| GOTERM_BP_ALL | GO:0006094~gluconeogenesis | 5 | 1.168224 | 0.064869 | LOC102724428, SDS, G6PC3, DGAT2, SIK1 | 349 | 73 | 16792 | 3.295521 | 1 | 0.796492 | 0.782555 |
| GOTERM_BP_ALL | GO:0044783~G1 DNA damage checkpoint | 5 | 1.168224 | 0.064869 | PLK3, PRKDC, E2F1, ARID3A, AURKA | 349 | 73 | 16792 | 3.295521 | 1 | 0.796492 | 0.782555 |
| GOTERM_BP_ALL | GO:0080090~regulation of primary metabolic process | 135 | 31.54206 | 0.065104 | SCARB1, ZNF296, CRTC1, HDAC11, SERPINE1, TONSL, SCX, CDC20, PTTG1, SOX18, SALL4, ZXDC, PHLDA2, TNFRSF4, MNX1, CHRNB2, SPINK4, DGAT2, KMT5C, SERPINF2, SOX12, BHLHA15, MAPK8IP2, SRCIN1, RUNDC3A, FOXP3, MAPK8IP1, CDC25B, LOC102724428, EID2, HOXB9, RFX6, CCNE1, TIMELESS, DPEP1, SIK1, HOXB2, TRIB3, ANAPC5, ABCG1, SHC2, BEX1, PRKDC, FOXO6, PKMYT1, C2, NME1-NME2, VTN, PCSK1N, SOCS1, PCBP3, DHX34, MIER2, HOXC9, HOXC6, RTEL1-TNFRSF6B, CDT1, PLK3, UBE2C, CBX2, SMAD9, ABCA7, NR0B2, ST18, DRAP1, PARP10, UBE2S, CDK4, MAFG, MAP3K10, SP5, MELTF, FGFR4, RNF187, MESP1, SERPINA1, DYRK2, SERPINA10, CELF4, SHB, HOXC10, WFDC2, DUSP10, HEY1, MYBL2, TEAD4, TGIF1, EMX1, MAP4K2, MYOC, TESC, H2AFX, APOA2, ADRA2C, ETV4, CHI3L1, TNFRSF25, CEBPB, UHRF1, LRP5, DBF4B, ASCL1, ASCL2, CLN6, RELB, AURKA, DLL4, CST3, CST1, PARD6A, PUF60, E2F1, PRAME, POLR2I, DTD1, HES4, PPP1R14B, PPP1R14C, NPM2, ZNF580, CDKN2A, GDF15, IL34, ARID3A, CCK, GUCA2A, AGT, MAD2L2, HEYL, FABP4, APOC1, NEURL1, INSM1, CDK5R2, FOXA2 | 349 | 5798 | 16792 | 1.120295 | 1 | 0.797271 | 0.783321 |
| GOTERM_BP_ALL | GO:0015031~protein transport | 49 | 11.4486 | 0.065359 | SNAP25, NXT1, DYRK2, TONSL, AACS, IPO4, SCAMP5, RINL, RPS19, FFAR2, TNFRSF4, ATP6V0B, NUP210, ABCC8, TESC, APOA2, MYO7A, ADRA2C, AP3B2, SRCIN1, FOXP3, VGF, RFX6, CHI3L1, TSNARE1, STX1A, LRP5, CACNA1D, RRBP1, TIMM10, CPLX1, TIMM50, TRPM2, SOCS1, RAB26, UCP2, PLK3, PTPRN2, KCNJ11, CDKN2A, CADPS, ASPSCR1, NR0B2, AGT, SYT7, PARP10, NFASC, RAB15, FOXA2 | 349 | 1860 | 16792 | 1.267536 | 1 | 0.797473 | 0.783519 |
| GOTERM_BP_ALL | GO:0042592~homeostatic process | 44 | 10.28037 | 0.066016 | SCARB1, CRTC1, PRKDC, LRP5, ATP1A3, SCX, AQP3, AACS, CLN6, NME1-NME2, TRPM2, RPS19, POLD1, UCP2, FFAR2, SLC39A3, KCNH2, RTEL1-TNFRSF6B, EMX1, ATP6V0B, PTPRN2, DGAT2, LIG1, MYOC, KCNJ11, UBE2C, APOA2, ASPSCR1, CYBRD1, CCK, BHLHA15, FOXP3, AGT, FAM155B, FABP4, VGF, RFX6, UBE2S, MAFG, MELTF, FGFR4, KCNK3, ABCG1, FOXA2 | 349 | 1645 | 16792 | 1.286956 | 1 | 0.797473 | 0.783519 |
| GOTERM_BP_ALL | GO:0001933~negative regulation of protein phosphorylation | 14 | 3.271028 | 0.066134 | CDKN2A, PRKDC, UBE2C, TESC, LRP5, SHB, SRCIN1, MAPK8IP1, PARD6A, SOCS1, DUSP10, FABP4, TRIB3, INSM1 | 349 | 393 | 16792 | 1.714007 | 1 | 0.797473 | 0.783519 |
| GOTERM_BP_ALL | GO:0031018~endocrine pancreas development | 4 | 0.934579 | 0.066155 | RFX6, INSM1, MNX1, FOXA2 | 349 | 45 | 16792 | 4.276855 | 1 | 0.797473 | 0.783519 |
| GOTERM_BP_ALL | GO:0045840~positive regulation of mitotic nuclear division | 4 | 0.934579 | 0.066155 | UBE2C, CDCA5, LRP5, AURKA | 349 | 45 | 16792 | 4.276855 | 1 | 0.797473 | 0.783519 |
| GOTERM_BP_ALL | GO:0070542~response to fatty acid | 4 | 0.934579 | 0.066155 | NME1-NME2, UCP2, E2F1, FFAR2 | 349 | 45 | 16792 | 4.276855 | 1 | 0.797473 | 0.783519 |
| GOTERM_BP_ALL | GO:0005996~monosaccharide metabolic process | 10 | 2.336449 | 0.066939 | LOC102724428, SDS, G6PC3, DGAT2, DYRK2, KCNJ11, LRP5, SIK1, PHLDA2, FOXA2 | 349 | 243 | 16792 | 1.980025 | 1 | 0.804838 | 0.790755 |
| GOTERM_BP_ALL | GO:0021953~central nervous system neuron differentiation | 8 | 1.869159 | 0.068978 | CHRNB2, DLL4, EMX1, GDPD5, ASCL1, MNX1, HOXC10, EPHB3 | 349 | 173 | 16792 | 2.224953 | 1 | 0.823402 | 0.808994 |
| GOTERM_BP_ALL | GO:0065005~protein-lipid complex assembly | 3 | 0.700935 | 0.069436 | APOC1, APOA2, ABCA7 | 349 | 21 | 16792 | 6.873516 | 1 | 0.823402 | 0.808994 |
| GOTERM_BP_ALL | GO:0008104~protein localization | 62 | 14.48598 | 0.069441 | EXOC3L1, SNAP25, NXT1, DYRK2, GPAA1, TONSL, AACS, IPO4, SCAMP5, RINL, RPS19, FFAR2, GRASP, TNFRSF4, ATP6V0B, NUP210, ABCC8, TESC, APOA2, MYO7A, KCNAB2, ADRA2C, AP3B2, SRCIN1, FOXP3, VGF, RFX6, CHI3L1, TSNARE1, ABCG1, STX1A, CDCA5, LRP5, CACNA1D, RRBP1, TIMM10, CENPA, CPLX1, AURKA, TIMM50, TRPM2, PARD6A, SOCS1, RAB26, UCP2, E2F1, BMF, PLK3, CMTM8, PTPRN2, KCNJ11, CDKN2A, CADPS, ASPSCR1, ABCA7, NR0B2, AGT, SYT7, PARP10, NFASC, RAB15, FOXA2 | 349 | 2448 | 16792 | 1.218589 | 1 | 0.823402 | 0.808994 |
| GOTERM_BP_ALL | GO:0048666~neuron development | 28 | 6.542056 | 0.069477 | SPON2, CRTC1, BICDL1, FOXO6, STMN3, GDPD5, ASCL1, AURKA, RND1, NME1-NME2, CDC20, MNX1, EPHB3, CTHRC1, CHRNB2, EMX1, UNC13A, MYOC, CCK, MYO7A, MAPK8IP2, ETV4, SRCIN1, SDK1, NFASC, ARTN, NEURL1, INSM1 | 349 | 965 | 16792 | 1.396072 | 1 | 0.823402 | 0.808994 |
| GOTERM_BP_ALL | GO:0050709~negative regulation of protein secretion | 6 | 1.401869 | 0.06949 | KCNJ11, ABCC8, UCP2, APOA2, SRCIN1, FOXP3 | 349 | 106 | 16792 | 2.723469 | 1 | 0.823402 | 0.808994 |
| GOTERM_BP_ALL | GO:0060563~neuroepithelial cell differentiation | 4 | 0.934579 | 0.069729 | EMX1, MYO7A, INSM1, ASCL1 | 349 | 46 | 16792 | 4.183879 | 1 | 0.823402 | 0.808994 |
| GOTERM_BP_ALL | GO:0051055~negative regulation of lipid biosynthetic process | 4 | 0.934579 | 0.069729 | LOC102724428, APOC1, TRIB3, SIK1 | 349 | 46 | 16792 | 4.183879 | 1 | 0.823402 | 0.808994 |
| GOTERM_BP_ALL | GO:0050794~regulation of cellular process | 232 | 54.20561 | 0.07025 | ZNF296, HDAC11, PLEKHB1, SERPINE1, TONSL, SCX, STMN3, AQP3, SCAMP5, CDC20, RPS19, SOX18, ZXDC, MNX1, EPHB3, IER3, UNC13A, NUP210, SERPINF2, SOX12, BHLHA15, MAPK8IP2, MAPK8IP1, EID2, HOXB9, CLDN3, RFX6, SIK1, HOXB2, TRIB3, PLPP2, STX1A, BEX2, TPH1, BEX1, SHMT2, PRKDC, PPP1R35, CDCA5, C2CD4A, C2CD4B, C2CD4C, C2, TRPM2, PCBP3, DHX34, MIER2, HOXC9, HOXC6, RTEL1-TNFRSF6B, CDT1, PLK3, STARD10, ABCA7, NR0B2, ST18, TMEM198, PARP10, ARTN, FXYD2, MESP1, SERPINA1, SERPINA10, SHB, HOXC10, WFDC2, AACS, HEY1, ADAMTSL2, FFAR2, TEAD4, MAP4K2, ATP6V0B, RPS6KL1, MYOC, GP1BB, TESC, SYP, MTSS1, MMP11, KIF2C, GDPD5, ASCL1, CRIP1, ASCL2, RELB, CST3, CST1, NPW, UCP2, PRAME, DTD1, LRRC24, NPM2, ZNF580, CDKN2A, GDF15, IL34, TMEM176B, TMEM176A, STAC3, AGT, GUCA2A, MAD2L2, HEYL, FABP4, SMOC1, SHKBP1, TACC3, RIMBP2, CDK5R2, FOXA2, SCARB1, RTKN, CRTC1, REG1A, TMEM145, PTTG1, SALL4, PHLDA2, TNFRSF4, PPFIA3, CHRNB2, SPINK4, KCNH2, TPRA1, DGAT2, KCNH6, KMT5C, RUNDC3A, FOXP3, SRCIN1, PGF, TRAIP, CDC25B, LOC102724428, CCNE1, TIMELESS, DPEP1, ANAPC5, ABCG1, LY6E, GNAZ, REG3A, SHC2, DPT, FOXO6, CACNA1D, CPLX2, SEZ6L2, PKMYT1, CPLX1, TIMM50, NME1-NME2, VTN, ADGRG1, PCSK1N, SOCS1, ADGRG5, RHPN1, BMF, UBE2C, CBX2, SMAD9, SYT7, DRAP1, SDK1, KIF18B, UBE2S, CDK4, MAFG, MAP3K10, SP5, MELTF, FGFR4, RNF187, SNAP25, DAGLA, DYRK2, ZC3H3, CELF4, RND1, DUSP10, DACT2, MYBL2, CA8, GRASP, TGIF1, PLEKHG4, EMX1, ABCC8, MMP1, H2AFX, APOA2, KCNAB2, ADRA2C, ETV4, VGF, CHI3L1, TNFRSF25, PFDN2, CEBPB, UHRF1, RGS16, LRP5, ATP1A3, DBF4B, CLN6, AURKA, DLL3, DLL4, P2RY6, PARD6A, PUF60, GNG4, RAB26, E2F1, POLR2I, CTHRC1, HES4, OR51E1, PPP1R14B, PPP1R14C, KCNJ11, ARID3A, CCK, PRC1, RAB15, TSPAN18, APOC1, NEURL1, INSM1 | 349 | 10489 | 16792 | 1.064219 | 1 | 0.827452 | 0.812973 |
| GOTERM_BP_ALL | GO:0003006~developmental process involved in reproduction | 20 | 4.672897 | 0.070951 | CEBPB, NPM2, PRKDC, TESC, CBX2, NUDT1, CELF4, SCX, TPGS1, SHB, ASCL2, CRIP1, CDC25B, AURKA, CST3, ADGRG1, VGF, HEY1, NEURL1, PHLDA2 | 349 | 636 | 16792 | 1.513038 | 1 | 0.833589 | 0.819002 |
| GOTERM_BP_ALL | GO:0031327~negative regulation of cellular biosynthetic process | 40 | 9.345794 | 0.071263 | MESP1, CEBPB, ZNF296, UHRF1, CELF4, TONSL, SCX, ASCL1, ASCL2, RELB, DLL4, PCBP3, HEY1, SOX18, SALL4, E2F1, PRAME, TNFRSF4, HOXC6, TGIF1, PLK3, CDKN2A, CBX2, H2AFX, ABCA7, NR0B2, FOXP3, MAD2L2, DRAP1, HEYL, LOC102724428, EID2, FABP4, APOC1, TIMELESS, MAP3K10, SIK1, TRIB3, INSM1, FOXA2 | 349 | 1480 | 16792 | 1.300395 | 1 | 0.835134 | 0.82052 |
| GOTERM_BP_ALL | GO:0006954~inflammatory response | 20 | 4.672897 | 0.072017 | REG3A, CEBPB, SERPINA1, ZNF580, IL34, SERPINF2, SERPINE1, APOA2, FOXP3, AGT, C2, RELB, VTN, DUSP10, FABP4, RPS19, CHI3L1, TNFRSF25, FFAR2, TNFRSF4 | 349 | 637 | 16792 | 1.510663 | 1 | 0.841838 | 0.827107 |
| GOTERM_BP_ALL | GO:0010629~negative regulation of gene expression | 40 | 9.345794 | 0.072505 | MESP1, CEBPB, ZNF296, UHRF1, SERPINE1, CELF4, SCX, ASCL1, ASCL2, RELB, DLL4, PCBP3, HEY1, SOX18, SALL4, E2F1, PRAME, POLR2I, TNFRSF4, TGIF1, PLK3, NUP210, CDKN2A, CBX2, H2AFX, SERPINF2, NR0B2, FOXP3, AGT, PARP10, MAD2L2, DRAP1, HEYL, EID2, FABP4, TIMELESS, MAP3K10, TRIB3, INSM1, FOXA2 | 349 | 1485 | 16792 | 1.296017 | 1 | 0.845402 | 0.830609 |
| GOTERM_BP_ALL | GO:0045921~positive regulation of exocytosis | 5 | 1.168224 | 0.072953 | SCAMP5, RAB15, STX1A, SYT7, CDK5R2 | 349 | 76 | 16792 | 3.165435 | 1 | 0.846365 | 0.831555 |
| GOTERM_BP_ALL | GO:0019319~hexose biosynthetic process | 5 | 1.168224 | 0.072953 | LOC102724428, SDS, G6PC3, DGAT2, SIK1 | 349 | 76 | 16792 | 3.165435 | 1 | 0.846365 | 0.831555 |
| GOTERM_BP_ALL | GO:0006357~regulation of transcription from RNA polymerase II promoter | 48 | 11.21495 | 0.073827 | MESP1, CEBPB, ZNF296, BEX1, UHRF1, CRTC1, PRKDC, SERPINE1, LRP5, FOXO6, SCX, ASCL1, ASCL2, RELB, NME1-NME2, DLL4, PCBP3, HEY1, SOX18, SALL4, E2F1, MYBL2, MNX1, HOXC6, TGIF1, TEAD4, PLK3, CDKN2A, CBX2, SERPINF2, SMAD9, ARID3A, SOX12, BHLHA15, ETV4, NR0B2, FOXP3, MAD2L2, DRAP1, HEYL, EID2, HOXB9, RFX6, MAFG, TIMELESS, TRIB3, INSM1, FOXA2 | 349 | 1835 | 16792 | 1.258584 | 1 | 0.852415 | 0.837499 |
| GOTERM_BP_ALL | GO:0051276~chromosome organization | 33 | 7.71028 | 0.073843 | UHRF1, PRKDC, CDCA5, HDAC11, CENPA, IPO4, NCAPH, AURKA, CDC20, RECQL4, PTTG1, POLD1, RAD54L, H1FX, GINS2, RTEL1-TNFRSF6B, NPM2, LIG1, CDKN2A, KMT5C, UBE2C, CBX2, H2AFX, FOXP3, PARP10, MAD2L2, KIF18B, PRC1, INCENP, TACC3, KIF2C, MCM2, FOXA2 | 349 | 1184 | 16792 | 1.341032 | 1 | 0.852415 | 0.837499 |
| GOTERM_BP_ALL | GO:0006813~potassium ion transport | 9 | 2.102804 | 0.074584 | KCNH2, KCNJ11, KCNH6, ABCC8, FXYD2, ATP1A3, CACNA1D, KCNAB2, KCNK3 | 349 | 212 | 16792 | 2.042602 | 1 | 0.858824 | 0.843796 |
| GOTERM_BP_ALL | GO:0030534~adult behavior | 7 | 1.635514 | 0.075327 | CHRNB2, SDK1, MAFG, ATP1A3, SEZ6L2, TPGS1, FOXA2 | 349 | 142 | 16792 | 2.371847 | 1 | 0.862573 | 0.847479 |
| GOTERM_BP_ALL | GO:0032461~positive regulation of protein oligomerization | 3 | 0.700935 | 0.075365 | MMP1, CCK, BMF | 349 | 22 | 16792 | 6.561084 | 1 | 0.862573 | 0.847479 |
| GOTERM_BP_ALL | GO:0050767~regulation of neurogenesis | 21 | 4.906542 | 0.075468 | CHRNB2, EMX1, SNAP25, UNC13A, CRTC1, FOXO6, GDPD5, ASCL1, ADRA2C, ASCL2, SRCIN1, DLL3, DLL4, NME1-NME2, CDC20, SDK1, HEYL, ADGRG1, DUSP10, NEURL1, EPHB3 | 349 | 682 | 16792 | 1.481535 | 1 | 0.862573 | 0.847479 |
| GOTERM_BP_ALL | GO:2000177~regulation of neural precursor cell proliferation | 5 | 1.168224 | 0.075757 | DLL4, EMX1, ADGRG1, INSM1, ASCL1 | 349 | 77 | 16792 | 3.124326 | 1 | 0.863736 | 0.848622 |
| GOTERM_BP_ALL | GO:1902580~single-organism cellular localization | 31 | 7.242991 | 0.076336 | SNAP25, DYRK2, TONSL, C2CD4A, C2CD4B, TIMM10, C2CD4C, CPLX2, CENPA, CPLX1, IPO4, TIMM50, RPS19, RAB26, E2F1, BMF, CYC1, KIF1A, CMTM8, UNC13A, NUP210, TESC, CADPS, MYO7A, AP3B2, SYT7, AGT, PARP10, NFASC, ABCG1, STX1A | 349 | 1102 | 16792 | 1.353496 | 1 | 0.867362 | 0.852185 |
| GOTERM_BP_ALL | GO:0002697~regulation of immune effector process | 12 | 2.803738 | 0.076449 | SPON2, VTN, DUSP10, RPS19, PRKDC, APOA2, FFAR2, SHB, PGC, TNFRSF4, FOXP3, C2 | 349 | 325 | 16792 | 1.77654 | 1 | 0.867362 | 0.852185 |
| GOTERM_BP_ALL | GO:0006974~cellular response to DNA damage stimulus | 24 | 5.607477 | 0.076772 | GINS2, RTEL1-TNFRSF6B, PLK3, RNASEH2A, DYRK2, LIG1, CDKN2A, UHRF1, PRKDC, CDCA5, H2AFX, NUDT1, TONSL, ARID3A, CRIP1, AURKA, RECQL4, MAD2L2, PTTG1, POLD1, E2F1, TIMELESS, RAD54L, POLR2I | 349 | 807 | 16792 | 1.430918 | 1 | 0.868714 | 0.853513 |
| GOTERM_BP_ALL | GO:0009994~oocyte differentiation | 4 | 0.934579 | 0.077131 | NPM2, SHB, CDC25B, AURKA | 349 | 48 | 16792 | 4.009551 | 1 | 0.868714 | 0.853513 |
| GOTERM_BP_ALL | GO:0006953~acute-phase response | 4 | 0.934579 | 0.077131 | REG3A, CEBPB, SERPINA1, SERPINF2 | 349 | 48 | 16792 | 4.009551 | 1 | 0.868714 | 0.853513 |
| GOTERM_BP_ALL | GO:0030855~epithelial cell differentiation | 18 | 4.205607 | 0.077439 | EMX1, REG3A, MESP1, CEBPB, SCX, MYO7A, ASCL1, AQP3, MTSS1, NME1-NME2, CST3, CLDN3, HEY1, RFX6, SOX18, MAFG, DACT2, INSM1 | 349 | 563 | 16792 | 1.5383 | 1 | 0.870065 | 0.85484 |
| GOTERM_BP_ALL | GO:0045184~establishment of protein localization | 52 | 12.14953 | 0.078076 | SNAP25, NXT1, DYRK2, TONSL, AACS, IPO4, SCAMP5, RINL, RPS19, FFAR2, TNFRSF4, ATP6V0B, NUP210, ABCC8, TESC, APOA2, MYO7A, ADRA2C, AP3B2, SRCIN1, FOXP3, VGF, RFX6, CHI3L1, TSNARE1, ABCG1, STX1A, LRP5, CACNA1D, RRBP1, TIMM10, CPLX1, TIMM50, TRPM2, SOCS1, RAB26, UCP2, E2F1, BMF, PLK3, PTPRN2, KCNJ11, CDKN2A, CADPS, ASPSCR1, NR0B2, AGT, SYT7, PARP10, NFASC, RAB15, FOXA2 | 349 | 2021 | 16792 | 1.237981 | 1 | 0.875098 | 0.859786 |
| GOTERM_BP_ALL | GO:0071322~cellular response to carbohydrate stimulus | 6 | 1.401869 | 0.078658 | NME1-NME2, PTPRN2, RFX6, KCNJ11, UCP2, AACS | 349 | 110 | 16792 | 2.624433 | 1 | 0.877367 | 0.862014 |
| GOTERM_BP_ALL | GO:0032963~collagen metabolic process | 6 | 1.401869 | 0.078658 | CST3, MMP11, ADAMTS14, MMP1, SERPINF2, SCX | 349 | 110 | 16792 | 2.624433 | 1 | 0.877367 | 0.862014 |
| GOTERM_BP_ALL | GO:0019222~regulation of metabolic process | 142 | 33.17757 | 0.078885 | SCARB1, ZNF296, CRTC1, HDAC11, SERPINE1, TONSL, SCX, CDC20, PTTG1, RPS19, SOX18, SALL4, ZXDC, PHLDA2, TNFRSF4, MNX1, CHRNB2, SPINK4, DGAT2, NUP210, KMT5C, SERPINF2, SOX12, BHLHA15, MAPK8IP2, SRCIN1, RUNDC3A, FOXP3, MAPK8IP1, CDC25B, LOC102724428, EID2, HOXB9, RFX6, CCNE1, TIMELESS, DPEP1, SIK1, HOXB2, TRIB3, ANAPC5, ABCG1, SHC2, BEX1, PRKDC, PPP1R35, FOXO6, PKMYT1, C2, NME1-NME2, VTN, PCSK1N, SOCS1, PCBP3, DHX34, MIER2, HOXC9, HOXC6, RTEL1-TNFRSF6B, CDT1, PLK3, UBE2C, CBX2, SMAD9, ABCA7, NR0B2, ST18, DRAP1, PARP10, UBE2S, CDK4, MAFG, MAP3K10, SP5, MELTF, FGFR4, RNF187, MESP1, SERPINA1, DYRK2, ZC3H3, SERPINA10, CELF4, SHB, HOXC10, WFDC2, DUSP10, SPR, HEY1, MYBL2, TEAD4, TGIF1, EMX1, MAP4K2, MYOC, TESC, H2AFX, APOA2, ADRA2C, ETV4, CHI3L1, TNFRSF25, CEBPB, UHRF1, LRP5, DBF4B, ASCL1, CRIP1, ASCL2, CLN6, RELB, AURKA, DLL4, CST3, CST1, PARD6A, PUF60, E2F1, PRAME, POLR2I, DTD1, HES4, PPP1R14B, PPP1R14C, NPM2, ZNF580, CDKN2A, GDF15, IL34, ARID3A, CCK, GUCA2A, AGT, MAD2L2, HEYL, FABP4, APOC1, NEURL1, RIMBP2, INSM1, CDK5R2, FOXA2 | 349 | 6171 | 16792 | 1.107158 | 1 | 0.87779 | 0.86243 |
| GOTERM_BP_ALL | GO:0048771~tissue remodeling | 7 | 1.635514 | 0.079365 | DLL4, CST3, TPH1, LRP5, AGT, SYT7, CTHRC1 | 349 | 144 | 16792 | 2.338905 | 1 | 0.879423 | 0.864035 |
| GOTERM_BP_ALL | GO:0061302~smooth muscle cell-matrix adhesion | 2 | 0.46729 | 0.080362 | VTN, SERPINE1 | 349 | 4 | 16792 | 24.05731 | 1 | 0.879423 | 0.864035 |
| GOTERM_BP_ALL | GO:0003072~renal control of peripheral vascular resistance involved in regulation of systemic arterial blood pressure | 2 | 0.46729 | 0.080362 | SERPINF2, AGT | 349 | 4 | 16792 | 24.05731 | 1 | 0.879423 | 0.864035 |
| GOTERM_BP_ALL | GO:0060264~regulation of respiratory burst involved in inflammatory response | 2 | 0.46729 | 0.080362 | DUSP10, RPS19 | 349 | 4 | 16792 | 24.05731 | 1 | 0.879423 | 0.864035 |
| GOTERM_BP_ALL | GO:1902866~regulation of retina development in camera-type eye | 2 | 0.46729 | 0.080362 | DLL4, CELF4 | 349 | 4 | 16792 | 24.05731 | 1 | 0.879423 | 0.864035 |
| GOTERM_BP_ALL | GO:0002536~respiratory burst involved in inflammatory response | 2 | 0.46729 | 0.080362 | DUSP10, RPS19 | 349 | 4 | 16792 | 24.05731 | 1 | 0.879423 | 0.864035 |
| GOTERM_BP_ALL | GO:0002034~regulation of blood vessel size by renin-angiotensin | 2 | 0.46729 | 0.080362 | SERPINF2, AGT | 349 | 4 | 16792 | 24.05731 | 1 | 0.879423 | 0.864035 |
| GOTERM_BP_ALL | GO:0045604~regulation of epidermal cell differentiation | 4 | 0.934579 | 0.080957 | NME1-NME2, REG3A, MAFG, AQP3 | 349 | 49 | 16792 | 3.927724 | 1 | 0.882959 | 0.867508 |
| GOTERM_BP_ALL | GO:0048169~regulation of long-term neuronal synaptic plasticity | 3 | 0.700935 | 0.081448 | NEURL1, SYP, AGT | 349 | 23 | 16792 | 6.275819 | 1 | 0.882959 | 0.867508 |
| GOTERM_BP_ALL | GO:0021895~cerebral cortex neuron differentiation | 3 | 0.700935 | 0.081448 | EMX1, GDPD5, ASCL1 | 349 | 23 | 16792 | 6.275819 | 1 | 0.882959 | 0.867508 |
| GOTERM_BP_ALL | GO:0033260~nuclear DNA replication | 3 | 0.700935 | 0.081448 | GINS2, LIG1, DBF4B | 349 | 23 | 16792 | 6.275819 | 1 | 0.882959 | 0.867508 |
| GOTERM_BP_ALL | GO:1902680~positive regulation of RNA biosynthetic process | 37 | 8.64486 | 0.08227 | MESP1, CEBPB, BEX1, UHRF1, CRTC1, PRKDC, SERPINE1, LRP5, SCX, ASCL1, RELB, NME1-NME2, HEY1, SOX18, SALL4, ZXDC, E2F1, MYBL2, POLR2I, CDKN2A, TESC, SERPINF2, SMAD9, ARID3A, SOX12, BHLHA15, ETV4, FOXP3, AGT, MAD2L2, HEYL, HOXB9, RFX6, CCNE1, MAFG, RNF187, FOXA2 | 349 | 1369 | 16792 | 1.300395 | 1 | 0.889782 | 0.874213 |
| GOTERM_BP_ALL | GO:0050789~regulation of biological process | 240 | 56.07477 | 0.082908 | SPON2, ZNF296, HDAC11, PLEKHB1, SERPINE1, TONSL, SCX, STMN3, AQP3, SCAMP5, CDC20, RPS19, SOX18, ZXDC, MNX1, EPHB3, IER3, UNC13A, NUP210, SERPINF2, SOX12, BHLHA15, MAPK8IP2, MAPK8IP1, EID2, HOXB9, CLDN3, RFX6, ORAI1, SIK1, HOXB2, TRIB3, PLPP2, STX1A, BEX2, TPH1, BEX1, SHMT2, PRKDC, PPP1R35, CDCA5, C2CD4A, C2CD4B, C2CD4C, C2, TRPM2, PCBP3, DHX34, MIER2, HOXC9, HOXC6, RTEL1-TNFRSF6B, CDT1, PLK3, PTPRN2, STARD10, ABCA7, NR0B2, ST18, TMEM198, PARP10, ARTN, FXYD2, MESP1, SERPINA1, SERPINA10, SHB, HOXC10, WFDC2, AACS, HEY1, ADAMTSL2, FFAR2, TEAD4, MAP4K2, ATP6V0B, RPS6KL1, MYOC, GP1BB, TESC, SYP, MTSS1, MMP11, KIF2C, GDPD5, ASCL1, CRIP1, ASCL2, RELB, CST3, CST1, NPW, UCP2, PRAME, DTD1, LRRC24, NPM2, ZNF580, CDKN2A, GDF15, IL34, TMEM176B, TMEM176A, STAC3, AGT, GUCA2A, MAD2L2, HEYL, FABP4, SMOC1, SHKBP1, TACC3, RIMBP2, CDK5R2, FOXA2, SCARB1, RTKN, CRTC1, REG1A, TMEM145, PTTG1, SALL4, PHLDA2, TNFRSF4, PPFIA3, CHRNB2, SPINK4, KCNH2, TPRA1, DGAT2, KCNH6, KMT5C, PGC, RUNDC3A, FOXP3, SRCIN1, PGF, TRAIP, CDC25B, LOC102724428, CCNE1, TIMELESS, DPEP1, ANAPC5, RELL2, ABCG1, LY6E, GNAZ, REG3A, SHC2, DPT, FOXO6, CACNA1D, CPLX2, SEZ6L2, PKMYT1, CPLX1, TIMM50, NME1-NME2, VTN, ADGRG1, PCSK1N, SOCS1, ADGRG5, RHPN1, BMF, UBE2C, CBX2, ASPSCR1, SMAD9, SYT7, DRAP1, SDK1, KIF18B, UBE2S, CDK4, MAFG, MAP3K10, SP5, MELTF, FGFR4, RNF187, SNAP25, DAGLA, DYRK2, ZC3H3, CELF4, RND1, DUSP10, SPR, DACT2, MYBL2, CA8, GRASP, TGIF1, PLEKHG4, EMX1, ABCC8, MMP1, H2AFX, APOA2, KCNAB2, ADRA2C, ETV4, VGF, CHI3L1, TNFRSF25, PFDN2, CEBPB, UHRF1, RGS16, LRP5, ATP1A3, DBF4B, CLN6, AURKA, DLL3, DLL4, P2RY6, PARD6A, PUF60, GNG4, RAB26, E2F1, POLR2I, CTHRC1, HES4, OR51E1, PPP1R14B, PPP1R14C, KCNJ11, ARID3A, CCK, PRC1, RAB15, TSPAN18, APOC1, NEURL1, INSM1, KCNK3 | 349 | 10922 | 16792 | 1.05727 | 1 | 0.894597 | 0.878943 |
| GOTERM_BP_ALL | GO:0051254~positive regulation of RNA metabolic process | 38 | 8.878505 | 0.083258 | MESP1, CEBPB, BEX1, UHRF1, CRTC1, PRKDC, SERPINE1, LRP5, CELF4, SCX, ASCL1, RELB, NME1-NME2, HEY1, SOX18, SALL4, ZXDC, E2F1, MYBL2, POLR2I, CDKN2A, TESC, SERPINF2, SMAD9, ARID3A, SOX12, BHLHA15, ETV4, FOXP3, AGT, MAD2L2, HEYL, HOXB9, RFX6, CCNE1, MAFG, RNF187, FOXA2 | 349 | 1415 | 16792 | 1.292124 | 1 | 0.896279 | 0.880595 |
| GOTERM_BP_ALL | GO:0045934~negative regulation of nucleobase-containing compound metabolic process | 37 | 8.64486 | 0.084025 | MESP1, CEBPB, ZNF296, UHRF1, CELF4, TONSL, SCX, ASCL1, ASCL2, RELB, DLL4, PCBP3, HEY1, SOX18, DHX34, SALL4, E2F1, PRAME, TNFRSF4, HOXC6, TGIF1, PLK3, CDKN2A, CBX2, H2AFX, NR0B2, FOXP3, MAD2L2, DRAP1, HEYL, EID2, FABP4, TIMELESS, MAP3K10, TRIB3, INSM1, FOXA2 | 349 | 1371 | 16792 | 1.298498 | 1 | 0.902437 | 0.886646 |
| GOTERM_BP_ALL | GO:0046364~monosaccharide biosynthetic process | 5 | 1.168224 | 0.084487 | LOC102724428, SDS, G6PC3, DGAT2, SIK1 | 349 | 80 | 16792 | 3.007163 | 1 | 0.903062 | 0.88726 |
| GOTERM_BP_ALL | GO:0051444~negative regulation of ubiquitin-protein transferase activity | 5 | 1.168224 | 0.084487 | MAD2L2, CDC20, CDKN2A, UBE2C, ANAPC5 | 349 | 80 | 16792 | 3.007163 | 1 | 0.903062 | 0.88726 |
| GOTERM_BP_ALL | GO:0046503~glycerolipid catabolic process | 4 | 0.934579 | 0.084863 | LIPE, FABP4, DAGLA, APOA2 | 349 | 50 | 16792 | 3.849169 | 1 | 0.903062 | 0.88726 |
| GOTERM_BP_ALL | GO:0090303~positive regulation of wound healing | 4 | 0.934579 | 0.084863 | VTN, REG3A, SERPINF2, SERPINE1 | 349 | 50 | 16792 | 3.849169 | 1 | 0.903062 | 0.88726 |
| GOTERM_BP_ALL | GO:0009890~negative regulation of biosynthetic process | 40 | 9.345794 | 0.085129 | MESP1, CEBPB, ZNF296, UHRF1, CELF4, TONSL, SCX, ASCL1, ASCL2, RELB, DLL4, PCBP3, HEY1, SOX18, SALL4, E2F1, PRAME, TNFRSF4, HOXC6, TGIF1, PLK3, CDKN2A, CBX2, H2AFX, ABCA7, NR0B2, FOXP3, MAD2L2, DRAP1, HEYL, LOC102724428, EID2, FABP4, APOC1, TIMELESS, MAP3K10, SIK1, TRIB3, INSM1, FOXA2 | 349 | 1504 | 16792 | 1.279644 | 1 | 0.90381 | 0.887995 |
| GOTERM_BP_ALL | GO:0006281~DNA repair | 17 | 3.971963 | 0.085399 | GINS2, RTEL1-TNFRSF6B, RNASEH2A, LIG1, UHRF1, PRKDC, CDCA5, H2AFX, NUDT1, TONSL, RECQL4, MAD2L2, PTTG1, POLD1, TIMELESS, RAD54L, POLR2I | 349 | 530 | 16792 | 1.543299 | 1 | 0.904602 | 0.888773 |
| GOTERM_BP_ALL | GO:1901615~organic hydroxy compound metabolic process | 14 | 3.271028 | 0.085711 | CHRNB2, SCARB1, TPH1, DGAT2, LRP5, APOA2, NR0B2, CLN6, LIPE, TTR, PAH, APOC1, INSM1, ABCG1 | 349 | 410 | 16792 | 1.642938 | 1 | 0.905839 | 0.889988 |
| GOTERM_BP_ALL | GO:0007162~negative regulation of cell adhesion | 9 | 2.102804 | 0.086236 | MAD2L2, CEBPB, MYOC, CDKN2A, SERPINE1, DACT2, MELTF, FOXP3, RND1 | 349 | 219 | 16792 | 1.977313 | 1 | 0.909311 | 0.8934 |
| GOTERM_BP_ALL | GO:0009648~photoperiodism | 3 | 0.700935 | 0.087677 | LOC102724428, CRTC1, SIK1 | 349 | 24 | 16792 | 6.014327 | 1 | 0.921082 | 0.904965 |
| GOTERM_BP_ALL | GO:0007417~central nervous system development | 26 | 6.074766 | 0.087751 | PRKDC, HDAC11, WDR62, GDPD5, SEZ6L2, ASCL1, HOXC10, DLL4, CST3, VTN, ADGRG1, DUSP10, E2F1, MNX1, EPHB3, CHRNB2, EMX1, H2AFX, SMAD9, SOX12, NEURL1, TACC3, HOXB2, PAFAH1B3, KCNK3, CDK5R2 | 349 | 904 | 16792 | 1.383827 | 1 | 0.921082 | 0.904965 |
| GOTERM_BP_ALL | GO:0001678~cellular glucose homeostasis | 6 | 1.401869 | 0.088442 | NME1-NME2, PTPRN2, RFX6, KCNJ11, UCP2, AACS | 349 | 114 | 16792 | 2.532348 | 1 | 0.926236 | 0.910028 |
| GOTERM_BP_ALL | GO:0007059~chromosome segregation | 12 | 2.803738 | 0.088704 | MAD2L2, CDC20, KIF18B, PTTG1, INCENP, UBE2C, PRC1, CDCA5, TACC3, KIF2C, CENPA, NCAPH | 349 | 334 | 16792 | 1.728669 | 1 | 0.926884 | 0.910665 |
| GOTERM_BP_ALL | GO:0006366~transcription from RNA polymerase II promoter | 47 | 10.98131 | 0.089686 | MESP1, CEBPB, ZNF296, BEX1, UHRF1, CRTC1, PRKDC, SERPINE1, LRP5, SCX, ASCL1, ASCL2, RELB, NME1-NME2, DLL4, PTTG1, PCBP3, HEY1, SOX18, SALL4, E2F1, MYBL2, POLR2I, TGIF1, TEAD4, PLK3, CDKN2A, CBX2, SERPINF2, SMAD9, ARID3A, SOX12, BHLHA15, ETV4, NR0B2, FOXP3, MAD2L2, DRAP1, HEYL, EID2, HOXB9, RFX6, MAFG, TIMELESS, TRIB3, INSM1, FOXA2 | 349 | 1820 | 16792 | 1.24252 | 1 | 0.93504 | 0.918679 |
| GOTERM_BP_ALL | GO:0044259~multicellular organismal macromolecule metabolic process | 6 | 1.401869 | 0.090982 | CST3, MMP11, ADAMTS14, MMP1, SERPINF2, SCX | 349 | 115 | 16792 | 2.510328 | 1 | 0.946421 | 0.92986 |
| GOTERM_BP_ALL | GO:1901264~carbohydrate derivative transport | 4 | 0.934579 | 0.092911 | SCARB1, G6PC3, SLC29A4, ABCG1 | 349 | 52 | 16792 | 3.701124 | 1 | 0.962156 | 0.94532 |
| GOTERM_BP_ALL | GO:0010812~negative regulation of cell-substrate adhesion | 4 | 0.934579 | 0.092911 | MYOC, CDKN2A, SERPINE1, MELTF | 349 | 52 | 16792 | 3.701124 | 1 | 0.962156 | 0.94532 |
| GOTERM_BP_ALL | GO:0010628~positive regulation of gene expression | 44 | 10.28037 | 0.09333 | MESP1, CEBPB, BEX1, UHRF1, CRTC1, PRKDC, SERPINE1, LRP5, CELF4, SCX, ASCL1, RELB, NME1-NME2, DLL4, HEY1, SOX18, SALL4, ZXDC, E2F1, MYBL2, POLR2I, ZNF580, CDKN2A, TESC, SERPINF2, SMAD9, ARID3A, SOX12, BHLHA15, ETV4, NR0B2, FOXP3, AGT, MAD2L2, HEYL, HOXB9, RFX6, CCNE1, CDK4, MAFG, MELTF, FGFR4, RNF187, FOXA2 | 349 | 1692 | 16792 | 1.251207 | 1 | 0.963096 | 0.946243 |
| GOTERM_BP_ALL | GO:0070613~regulation of protein processing | 5 | 1.168224 | 0.093685 | VTN, SERPINF2, SERPINE1, MELTF, C2 | 349 | 83 | 16792 | 2.898471 | 1 | 0.963096 | 0.946243 |
| GOTERM_BP_ALL | GO:0002028~regulation of sodium ion transport | 5 | 1.168224 | 0.093685 | LOC102724428, TESC, FXYD2, SIK1, AGT | 349 | 83 | 16792 | 2.898471 | 1 | 0.963096 | 0.946243 |
| GOTERM_BP_ALL | GO:0001556~oocyte maturation | 3 | 0.700935 | 0.094042 | SHB, CDC25B, AURKA | 349 | 25 | 16792 | 5.773754 | 1 | 0.963096 | 0.946243 |
| GOTERM_BP_ALL | GO:0061311~cell surface receptor signaling pathway involved in heart development | 3 | 0.700935 | 0.094042 | DLL4, MESP1, HEY1 | 349 | 25 | 16792 | 5.773754 | 1 | 0.963096 | 0.946243 |
| GOTERM_BP_ALL | GO:0033036~macromolecule localization | 69 | 16.1215 | 0.094782 | SCARB1, EXOC3L1, SNAP25, NXT1, DYRK2, GPAA1, ZC3H3, TONSL, AACS, IPO4, SCAMP5, RINL, RPS19, FFAR2, GRASP, TNFRSF4, ATP6V0B, DGAT2, NUP210, ABCC8, TESC, APOA2, MYO7A, KCNAB2, ATP11A, ADRA2C, AP3B2, SRCIN1, FOXP3, VGF, RFX6, CHI3L1, TSNARE1, ABCG1, STX1A, CDCA5, LRP5, CACNA1D, RRBP1, TIMM10, CENPA, CPLX1, AURKA, TIMM50, TRPM2, PARD6A, SOCS1, RAB26, UCP2, E2F1, BMF, PLK3, CMTM8, PTPRN2, KCNJ11, CDKN2A, CADPS, STARD10, ASPSCR1, ABCA7, NR0B2, AGT, SYT7, PARP10, NFASC, SLCO3A1, RAB15, APOC1, FOXA2 | 349 | 2817 | 16792 | 1.178526 | 1 | 0.968529 | 0.951582 |
| GOTERM_BP_ALL | GO:0046486~glycerolipid metabolic process | 13 | 3.037383 | 0.095332 | SCARB1, DGAT2, DAGLA, GPAA1, CPNE7, APOA2, LIPF, LOC102724428, LIPE, FABP4, APOC1, SIK1, FGFR4 | 349 | 379 | 16792 | 1.650369 | 1 | 0.97201 | 0.955002 |
| GOTERM_BP_ALL | GO:0006310~DNA recombination | 10 | 2.336449 | 0.095849 | RECQL4, GINS2, RTEL1-TNFRSF6B, LIG1, PRKDC, POLD1, H2AFX, TONSL, RAD54L, FOXP3 | 349 | 262 | 16792 | 1.836436 | 1 | 0.974485 | 0.957433 |
| GOTERM_BP_ALL | GO:1903508~positive regulation of nucleic acid-templated transcription | 36 | 8.411215 | 0.096809 | MESP1, CEBPB, BEX1, UHRF1, CRTC1, PRKDC, SERPINE1, LRP5, SCX, ASCL1, RELB, NME1-NME2, HEY1, SOX18, SALL4, ZXDC, E2F1, MYBL2, CDKN2A, TESC, SERPINF2, SMAD9, ARID3A, SOX12, BHLHA15, ETV4, FOXP3, AGT, MAD2L2, HEYL, HOXB9, RFX6, CCNE1, MAFG, RNF187, FOXA2 | 349 | 1349 | 16792 | 1.284007 | 1 | 0.974485 | 0.957433 |
| GOTERM_BP_ALL | GO:0045893~positive regulation of transcription, DNA-templated | 36 | 8.411215 | 0.096809 | MESP1, CEBPB, BEX1, UHRF1, CRTC1, PRKDC, SERPINE1, LRP5, SCX, ASCL1, RELB, NME1-NME2, HEY1, SOX18, SALL4, ZXDC, E2F1, MYBL2, CDKN2A, TESC, SERPINF2, SMAD9, ARID3A, SOX12, BHLHA15, ETV4, FOXP3, AGT, MAD2L2, HEYL, HOXB9, RFX6, CCNE1, MAFG, RNF187, FOXA2 | 349 | 1349 | 16792 | 1.284007 | 1 | 0.974485 | 0.957433 |
| GOTERM_BP_ALL | GO:0051983~regulation of chromosome segregation | 5 | 1.168224 | 0.096853 | MAD2L2, UBE2C, CDCA5, TACC3, KIF2C | 349 | 84 | 16792 | 2.863965 | 1 | 0.974485 | 0.957433 |
| GOTERM_BP_ALL | GO:1903317~regulation of protein maturation | 5 | 1.168224 | 0.096853 | VTN, SERPINF2, SERPINE1, MELTF, C2 | 349 | 84 | 16792 | 2.863965 | 1 | 0.974485 | 0.957433 |
| GOTERM_BP_ALL | GO:0014910~regulation of smooth muscle cell migration | 4 | 0.934579 | 0.097049 | VTN, P2RY6, SERPINE1, AGT | 349 | 53 | 16792 | 3.631292 | 1 | 0.974485 | 0.957433 |
| GOTERM_BP_ALL | GO:0061178~regulation of insulin secretion involved in cellular response to glucose stimulus | 4 | 0.934579 | 0.097049 | RFX6, UCP2, LRP5, FOXA2 | 349 | 53 | 16792 | 3.631292 | 1 | 0.974485 | 0.957433 |
| GOTERM_BP_ALL | GO:0048865~stem cell fate commitment | 2 | 0.46729 | 0.099425 | SOX18, ASCL1 | 349 | 5 | 16792 | 19.24585 | 1 | 0.985524 | 0.968279 |
| GOTERM_BP_ALL | GO:0010757~negative regulation of plasminogen activation | 2 | 0.46729 | 0.099425 | SERPINF2, SERPINE1 | 349 | 5 | 16792 | 19.24585 | 1 | 0.985524 | 0.968279 |
| GOTERM_BP_ALL | GO:0033567~DNA replication, Okazaki fragment processing | 2 | 0.46729 | 0.099425 | RNASEH2A, LIG1 | 349 | 5 | 16792 | 19.24585 | 1 | 0.985524 | 0.968279 |
| GOTERM_BP_ALL | GO:0098703~calcium ion import across plasma membrane | 2 | 0.46729 | 0.099425 | FAM155B, TRPM2 | 349 | 5 | 16792 | 19.24585 | 1 | 0.985524 | 0.968279 |
| GOTERM_BP_ALL | GO:2001199~negative regulation of dendritic cell differentiation | 2 | 0.46729 | 0.099425 | TMEM176B, TMEM176A | 349 | 5 | 16792 | 19.24585 | 1 | 0.985524 | 0.968279 |
| GOTERM_BP_ALL | GO:0031915~positive regulation of synaptic plasticity | 2 | 0.46729 | 0.099425 | CDC20, CPLX2 | 349 | 5 | 16792 | 19.24585 | 1 | 0.985524 | 0.968279 |

| **Table S3: Gene Ontology-Molecular Function (GO-MF) Functional Enrichment Results using DAVID** | | | | | | | | | | | |  |
| --- | --- | --- | --- | --- | --- | --- | --- | --- | --- | --- | --- | --- |
|  |  |  |  |  |  |  |  |  |  |  |  |  |
| Category | Term | Count | % | PValue | Genes | List Total | Pop Hits | Pop Total | Fold Enrichment | Bonferroni | Benjamini | FDR |
| GOTERM_MF_ALL | GO:0004857~enzyme inhibitor activity | 23 | 5.373832 | 1.63E-05 | SPINK4, PPP1R14B, RTKN, PPP1R14C, SERPINA1, CDKN2A, TESC, PPP1R35, SERPINF2, SERPINA10, SERPINE1, APOA2, WFDC2, AGT, MAPK8IP1, CST3, PCSK1N, CST1, SOCS1, PTTG1, APOC1, DPEP1, TRIB3 | 344 | 389 | 16881 | 2.901469 | 0.013762 | 0.013857 | 0.013711 |
| GOTERM_MF_ALL | GO:0046983~protein dimerization activity | 45 | 10.51402 | 4.57E-05 | MESP1, SCARB1, CEBPB, NAXE, SCX, TIMM10, ASCL1, CENPA, ASCL2, CLN6, ASGR1, SRM, TTR, RPS19, HEY1, MTHFD1L, SOX18, E2F1, HES4, KCNH2, CHRNB2, DGAT2, SDS, NUP210, GAD1, TESC, H2AFX, SERPINF2, APOA2, ARID3A, MYO7A, BHLHA15, ADRA2C, NR0B2, FOXP3, PGF, DRAP1, HEYL, MAFG, TIMELESS, MAP3K10, PAFAH1B3, KCNK3, ABCG1, STX1A | 344 | 1160 | 16881 | 1.903681 | 0.03818 | 0.016069 | 0.015899 |
| GOTERM_MF_ALL | GO:0000149~SNARE binding | 12 | 2.803738 | 5.66E-05 | SNAP25, EXOC3L1, UNC13A, ABCC8, C2CD4A, C2CD4B, TSNARE1, C2CD4C, CPLX2, CPLX1, STX1A, SYT7 | 344 | 127 | 16881 | 4.636788 | 0.047065 | 0.016069 | 0.015899 |
| GOTERM_MF_ALL | GO:0042802~identical protein binding | 49 | 11.4486 | 1.15E-04 | SCARB1, SERPINA1, MSI1, ASGR1, RPS19, GRASP, KCNH2, DGAT2, SDS, GP1BB, TESC, SERPINF2, APOA2, MYO7A, BHLHA15, SYP, ADRA2C, FOXP3, MTSS1, PGF, MAPK8IP1, CLDN3, TIMELESS, ORAI1, VWA1, PAFAH1B3, ABCG1, CEBPB, NAXE, SHMT2, UHRF1, TIMM10, ASCL1, CLN6, SRM, CST3, VTN, TTR, PUF60, MTHFD1L, STAC3, ARID3A, NR0B2, DRAP1, HEYL, SDK1, PRC1, MAP3K10, KCNK3 | 344 | 1358 | 16881 | 1.770664 | 0.093287 | 0.024481 | 0.024222 |
| GOTERM_MF_ALL | GO:0005515~protein binding | 251 | 58.64486 | 2.62E-04 | SPON2, CLPB, CPNE7, HDAC11, PLEKHB1, SERPINE1, TONSL, NUDT1, SCX, STMN3, TPGS1, SCAMP5, CDC20, LIPE, RPS19, SOX18, ZXDC, ARL6IP4, CHGB, IER3, SDS, UNC13A, NUP210, SERPINF2, MYO7A, BHLHA15, CPT1C, MAPK8IP2, MAPK8IP1, SULT2B1, EID2, HOXB9, CLDN3, RFX6, CCDC86, ORAI1, SIK1, FAM64A, TRIB3, PLPP2, STX1A, BEX2, BEX1, SHMT2, PRKDC, PPP1R35, CDCA5, C2CD4A, C2CD4B, C2CD4C, WDR62, NCAPH, HOXC9, CDT1, PLK3, STARD10, NR0B2, PARP10, NDUFAF8, ARTN, MESP1, SERPINA1, GPAA1, MRPS12, HEPACAM2, BICDL1, MYL6B, SHB, ASGR1, AACS, HEY1, ADAMTSL2, FFAR2, KIF1A, TEAD4, MAP4K2, MYOC, GP1BB, TESC, SYP, MTSS1, PDRG1, CTU1, KIFC2, INCENP, VWA1, KIF2C, PAFAH1B3, GRAMD1A, TIMM10, GDPD5, ASCL1, CENPA, SLC7A1, ASCL2, RELB, RECQL4, CST3, CST1, NPW, MTHFD1L, UCP2, RAD54L, PRAME, GINS2, NPM2, ZNF580, CDKN2A, GDF15, IL34, GAD1, STAC3, NPM3, AGT, GUCA2A, MAD2L2, SH3RF3, HEYL, SMOC1, SHKBP1, TACC3, FOXA2, EXOC3L1, SCARB1, RTKN, CRTC1, REG1A, MSI1, OGDHL, KLHL35, IPO4, PTTG1, SALL4, TXNL4A, PPFIA3, CHRNB2, KCNH2, DGAT2, KMT5C, FBXW9, ATP11A, RUNDC3A, FOXP3, SRCIN1, PGF, TRAIP, CDC25B, LOC102724428, CCNE1, TIMELESS, DPEP1, ANAPC5, TSNARE1, RELL2, RPP25, ABCG1, GNAZ, REG3A, SHC2, TROAP, FOXO6, CACNA1D, SLC1A5, CPLX2, PKMYT1, CPLX1, TIMM50, VTN, ADGRG1, PCSK1N, SOCS1, RHPN1, HYI, H1FX, C16ORF59, BMF, SAC3D1, UBE2C, SLC52A2, CBX2, ASPSCR1, CYBRD1, SMAD9, SYT7, DRAP1, SDK1, AMACR, KIF18B, UBE2S, CDK4, MAFG, MAP3K10, MELTF, FGFR4, ULBP2, RNF187, SNAP25, DYRK2, NXT1, PYCRL, PODXL2, ZC3H3, RND1, DACT2, MYBL2, CA8, GRASP, TGIF1, PLEKHG4, EMX1, ABCC8, H2AFX, APOA2, ADRA2C, ETV4, VGF, PFDN2, MCM2, CEBPB, NAXE, UHRF1, RGS16, LRP5, ATP1A3, DBF4B, LAGE3, CLN6, SRM, AURKA, DLL3, DLL4, P2RY6, TTR, PARD6A, PUF60, GNG4, RAB26, POLD1, E2F1, CTHRC1, HES4, CMTM8, KCNJ11, CADPS, ARID3A, CCK, NFASC, PRC1, RAB15, INSM1, KCNK3 | 344 | 10806 | 16881 | 1.139852 | 0.199779 | 0.044568 | 0.044097 |
| GOTERM_MF_ALL | GO:0019905~syntaxin binding | 9 | 2.102804 | 5.26E-04 | SNAP25, UNC13A, ABCC8, C2CD4A, C2CD4B, C2CD4C, CPLX2, CPLX1, SYT7 | 344 | 91 | 16881 | 4.853341 | 0.361131 | 0.074656 | 0.073868 |
| GOTERM_MF_ALL | GO:0004866~endopeptidase inhibitor activity | 12 | 2.803738 | 8.79E-04 | SPINK4, CST3, PCSK1N, CST1, SERPINA1, PTTG1, SERPINF2, SERPINA10, SERPINE1, DPEP1, WFDC2, AGT | 344 | 174 | 16881 | 3.384322 | 0.527229 | 0.102494 | 0.101411 |
| GOTERM_MF_ALL | GO:0061135~endopeptidase regulator activity | 12 | 2.803738 | 0.001158 | SPINK4, CST3, PCSK1N, CST1, SERPINA1, PTTG1, SERPINF2, SERPINA10, SERPINE1, DPEP1, WFDC2, AGT | 344 | 180 | 16881 | 3.271512 | 0.627372 | 0.102494 | 0.101411 |
| GOTERM_MF_ALL | GO:0019900~kinase binding | 25 | 5.841121 | 0.001158 | CEBPB, SHC2, DBF4B, AURKA, LIPE, SOCS1, PARD6A, RPS19, DACT2, E2F1, MAP4K2, MYOC, CDKN2A, CADPS, MAPK8IP2, SRCIN1, MAPK8IP1, CDC25B, MAD2L2, LOC102724428, CCNE1, PRC1, SIK1, TRIB3, STX1A | 344 | 595 | 16881 | 2.061877 | 0.627463 | 0.102494 | 0.101411 |
| GOTERM_MF_ALL | GO:0019901~protein kinase binding | 23 | 5.373832 | 0.001321 | MAP4K2, SHC2, MYOC, CDKN2A, CADPS, DBF4B, MAPK8IP2, SRCIN1, CDC25B, AURKA, MAPK8IP1, MAD2L2, LOC102724428, LIPE, PARD6A, SOCS1, RPS19, CCNE1, PRC1, DACT2, E2F1, TRIB3, SIK1 | 344 | 531 | 16881 | 2.125558 | 0.675794 | 0.102494 | 0.101411 |
| GOTERM_MF_ALL | GO:0030414~peptidase inhibitor activity | 12 | 2.803738 | 0.001323 | SPINK4, CST3, PCSK1N, CST1, SERPINA1, PTTG1, SERPINF2, SERPINA10, SERPINE1, DPEP1, WFDC2, AGT | 344 | 183 | 16881 | 3.21788 | 0.676378 | 0.102494 | 0.101411 |
| GOTERM_MF_ALL | GO:0008134~transcription factor binding | 22 | 5.140187 | 0.001899 | CEBPB, BEX1, CDKN2A, CRTC1, PRKDC, HDAC11, SCX, ASCL1, NR0B2, FOXP3, MAD2L2, HEYL, LOC102724428, PARD6A, HEY1, DACT2, E2F1, MAP3K10, SIK1, PRAME, FOXA2, HES4 | 344 | 512 | 16881 | 2.108591 | 0.802017 | 0.134447 | 0.133027 |
| GOTERM_MF_ALL | GO:0042803~protein homodimerization activity | 28 | 6.542056 | 0.002051 | SCARB1, CEBPB, NAXE, TIMM10, ASCL1, CLN6, ASGR1, SRM, RPS19, MTHFD1L, KCNH2, DGAT2, SDS, TESC, SERPINF2, APOA2, ARID3A, MYO7A, BHLHA15, ADRA2C, NR0B2, FOXP3, PGF, HEYL, TIMELESS, MAP3K10, KCNK3, ABCG1 | 344 | 730 | 16881 | 1.88224 | 0.826158 | 0.134447 | 0.133027 |
| GOTERM_MF_ALL | GO:0004867~serine-type endopeptidase inhibitor activity | 8 | 1.869159 | 0.003621 | SPINK4, PCSK1N, SERPINA1, SERPINF2, SERPINA10, SERPINE1, WFDC2, AGT | 344 | 97 | 16881 | 4.047231 | 0.954513 | 0.220055 | 0.21773 |
| GOTERM_MF_ALL | GO:0030234~enzyme regulator activity | 34 | 7.943925 | 0.003874 | SERPINA1, RTKN, PPP1R35, RGS16, SERPINA10, SERPINE1, DBF4B, COX6A1, WFDC2, CDC20, CST3, PCSK1N, CST1, SOCS1, RINL, PTTG1, SPINK4, PPP1R14B, PPP1R14C, CDKN2A, TESC, SERPINF2, APOA2, MAPK8IP2, RUNDC3A, GUCA2A, AGT, MAPK8IP1, CCNE1, CDK4, APOC1, DPEP1, TRIB3, CDK5R2 | 344 | 996 | 16881 | 1.675172 | 0.963383 | 0.220055 | 0.21773 |
| GOTERM_MF_ALL | GO:0043565~sequence-specific DNA binding | 36 | 8.411215 | 0.004285 | MESP1, CEBPB, UHRF1, FOXO6, SCX, ASCL1, CENPA, ASCL2, CRIP1, HOXC10, RELB, HEY1, SOX18, SALL4, E2F1, MYBL2, HOXC9, MNX1, HOXC6, TGIF1, EMX1, ARID3A, SOX12, BHLHA15, ETV4, FOXP3, HEYL, HOXB9, RFX6, MAFG, SP5, HOXB2, KIF2C, INSM1, MCM2, FOXA2 | 344 | 1080 | 16881 | 1.635756 | 0.974227 | 0.228161 | 0.22575 |
| GOTERM_MF_ALL | GO:0017075~syntaxin-1 binding | 4 | 0.934579 | 0.00541 | SNAP25, UNC13A, CPLX2, CPLX1 | 344 | 18 | 16881 | 10.90504 | 0.990162 | 0.258381 | 0.255652 |
| GOTERM_MF_ALL | GO:0061134~peptidase regulator activity | 12 | 2.803738 | 0.005459 | SPINK4, CST3, PCSK1N, CST1, SERPINA1, PTTG1, SERPINF2, SERPINA10, SERPINE1, DPEP1, WFDC2, AGT | 344 | 220 | 16881 | 2.676691 | 0.990568 | 0.258381 | 0.255652 |
| GOTERM_MF_ALL | GO:0046982~protein heterodimerization activity | 19 | 4.439252 | 0.007001 | CHRNB2, CEBPB, GAD1, H2AFX, APOA2, SCX, ADRA2C, CENPA, PGF, DRAP1, HEYL, TTR, SOX18, MAFG, TIMELESS, PAFAH1B3, KCNK3, ABCG1, STX1A | 344 | 465 | 16881 | 2.00512 | 0.997486 | 0.313939 | 0.310623 |
| GOTERM_MF_ALL | GO:0005215~transporter activity | 41 | 9.579439 | 0.007708 | SCARB1, TTYH3, TMEM63C, CPNE7, LRP5, ATP1A3, CACNA1D, SLC38A11, TIMM10, SLC1A5, COX6A1, SLC7A1, AQP3, CPLX1, IPO4, TRPM2, SLC25A22, SLC18A1, SLC39A3, KCNH2, CHRNB2, ATP6V0B, KCNH6, KCNJ11, ABCC8, SLC52A2, STARD10, APOA2, ABCA7, KCNAB2, SYP, ATP11A, AP3B2, FAM155B, FABP4, SLCO3A1, FXYD2, ORAI1, SLC29A4, KCNK3, ABCG1 | 344 | 1327 | 16881 | 1.516187 | 0.99863 | 0.32837 | 0.324902 |
| GOTERM_MF_ALL | GO:0019887~protein kinase regulator activity | 10 | 2.336449 | 0.008668 | SOCS1, CCNE1, CDKN2A, CDK4, TESC, TRIB3, DBF4B, MAPK8IP2, CDK5R2, MAPK8IP1 | 344 | 172 | 16881 | 2.853062 | 0.999399 | 0.351665 | 0.34795 |
| GOTERM_MF_ALL | GO:0019207~kinase regulator activity | 10 | 2.336449 | 0.016308 | SOCS1, CCNE1, CDKN2A, CDK4, TESC, TRIB3, DBF4B, MAPK8IP2, CDK5R2, MAPK8IP1 | 344 | 191 | 16881 | 2.56925 | 0.999999 | 0.599352 | 0.59302 |
| GOTERM_MF_ALL | GO:0008140~cAMP response element binding protein binding | 3 | 0.700935 | 0.016631 | LOC102724428, CRTC1, SIK1 | 344 | 10 | 16881 | 14.7218 | 0.999999 | 0.599352 | 0.59302 |
| GOTERM_MF_ALL | GO:0098772~molecular function regulator | 41 | 9.579439 | 0.016883 | SHC2, SERPINA1, RTKN, PPP1R35, RGS16, SERPINA10, SERPINE1, DBF4B, COX6A1, WFDC2, CDC20, CST3, PCSK1N, CST1, SOCS1, RINL, PTTG1, SPINK4, PLEKHG4, PPP1R14B, PPP1R14C, CDKN2A, TESC, SERPINF2, APOA2, KCNAB2, MAPK8IP2, RUNDC3A, GUCA2A, AGT, MAPK8IP1, CCNE1, ARTN, CDK4, FXYD2, APOC1, DPEP1, TRIB3, FGFR4, STX1A, CDK5R2 | 344 | 1397 | 16881 | 1.440214 | 0.999999 | 0.599352 | 0.59302 |
| GOTERM_MF_ALL | GO:0016887~ATPase activity | 17 | 3.971963 | 0.017986 | RTEL1-TNFRSF6B, ATP6V0B, CLPB, ABCC8, ATP1A3, ABCA7, MYO7A, ATP11A, RECQL4, KIF18B, KIFC2, DHX34, FXYD2, DHX37, KIF2C, KIF1A, ABCG1 | 344 | 439 | 16881 | 1.900309 | 1 | 0.612968 | 0.606493 |
| GOTERM_MF_ALL | GO:1990837~sequence-specific double-stranded DNA binding | 24 | 5.607477 | 0.023576 | TGIF1, MESP1, CEBPB, UHRF1, SCX, ARID3A, SOX12, BHLHA15, ASCL1, CENPA, ETV4, ASCL2, CRIP1, RELB, HEYL, HEY1, RFX6, SOX18, SP5, KIF2C, MYBL2, INSM1, FOXA2, MCM2 | 344 | 725 | 16881 | 1.624475 | 1 | 0.750323 | 0.742397 |
| GOTERM_MF_ALL | GO:0003690~double-stranded DNA binding | 26 | 6.074766 | 0.023778 | MESP1, CEBPB, UHRF1, PRKDC, SCX, ASCL1, CENPA, ASCL2, CRIP1, RELB, PCBP3, HEY1, SOX18, MYBL2, TGIF1, ARID3A, SOX12, BHLHA15, ETV4, HEYL, RFX6, SP5, KIF2C, INSM1, MCM2, FOXA2 | 344 | 805 | 16881 | 1.584956 | 1 | 0.750323 | 0.742397 |
| GOTERM_MF_ALL | GO:0004869~cysteine-type endopeptidase inhibitor activity | 5 | 1.168224 | 0.026892 | CST3, CST1, PTTG1, DPEP1, WFDC2 | 344 | 56 | 16881 | 4.381489 | 1 | 0.786557 | 0.778249 |
| GOTERM_MF_ALL | GO:0008289~lipid binding | 22 | 5.140187 | 0.027038 | SPON2, SCARB1, UNC13A, GPAA1, CADPS, STARD10, APOA2, C2CD4A, ATP1A3, NBEAL2, C2CD4B, C2CD4C, SYP, SYT7, LIPF, NME1-NME2, FABP4, APOC1, DPEP1, FFAR2, CDK5R2, ABCG1 | 344 | 656 | 16881 | 1.64573 | 1 | 0.786557 | 0.778249 |
| GOTERM_MF_ALL | GO:0055106~ubiquitin-protein transferase regulator activity | 3 | 0.700935 | 0.027696 | CDC20, CDKN2A, TRIB3 | 344 | 13 | 16881 | 11.32446 | 1 | 0.786557 | 0.778249 |
| GOTERM_MF_ALL | GO:0000981~RNA polymerase II transcription factor activity, sequence-specific DNA binding | 22 | 5.140187 | 0.029792 | TGIF1, MESP1, CEBPB, SCX, FOXO6, ARID3A, SOX12, BHLHA15, ASCL1, ETV4, ASCL2, FOXP3, RELB, HEYL, HEY1, RFX6, PCBP3, SOX18, MAFG, MYBL2, INSM1, FOXA2 | 344 | 662 | 16881 | 1.630814 | 1 | 0.818807 | 0.810158 |
| GOTERM_MF_ALL | GO:0055103~ligase regulator activity | 3 | 0.700935 | 0.031885 | CDC20, CDKN2A, TRIB3 | 344 | 14 | 16881 | 10.51557 | 1 | 0.834213 | 0.825401 |
| GOTERM_MF_ALL | GO:0019894~kinesin binding | 4 | 0.934579 | 0.033701 | KIF18B, PRC1, MAPK8IP2, MAPK8IP1 | 344 | 35 | 16881 | 5.608306 | 1 | 0.834213 | 0.825401 |
| GOTERM_MF_ALL | GO:0004190~aspartic-type endopeptidase activity | 4 | 0.934579 | 0.033701 | PGA3, PGC, PGA5, PGA4 | 344 | 35 | 16881 | 5.608306 | 1 | 0.834213 | 0.825401 |
| GOTERM_MF_ALL | GO:0005249~voltage-gated potassium channel activity | 6 | 1.401869 | 0.034963 | KCNH2, KCNJ11, KCNH6, ABCC8, KCNAB2, KCNK3 | 344 | 89 | 16881 | 3.30827 | 1 | 0.834213 | 0.825401 |
| GOTERM_MF_ALL | GO:0015079~potassium ion transmembrane transporter activity | 8 | 1.869159 | 0.035827 | KCNH2, KCNJ11, KCNH6, ABCC8, FXYD2, ATP1A3, KCNAB2, KCNK3 | 344 | 152 | 16881 | 2.582772 | 1 | 0.834213 | 0.825401 |
| GOTERM_MF_ALL | GO:0070001~aspartic-type peptidase activity | 4 | 0.934579 | 0.036228 | PGA3, PGC, PGA5, PGA4 | 344 | 36 | 16881 | 5.452519 | 1 | 0.834213 | 0.825401 |
| GOTERM_MF_ALL | GO:0019899~enzyme binding | 48 | 11.21495 | 0.040224 | CEBPB, SHC2, SERPINA1, RTKN, NXT1, PRKDC, PPP1R35, SERPINE1, BICDL1, DBF4B, IPO4, AURKA, CDC20, CST3, LIPE, CST1, SOCS1, PARD6A, RPS19, POLD1, DACT2, E2F1, GRASP, KCNH2, MAP4K2, NPM2, MYOC, CDKN2A, UBE2C, H2AFX, SERPINF2, CADPS, MAPK8IP2, SRCIN1, FOXP3, MAPK8IP1, CDC25B, MAD2L2, LOC102724428, CCNE1, UBE2S, PRC1, SIK1, TRIB3, ANAPC5, INSM1, STX1A, MCM2 | 344 | 1789 | 16881 | 1.316651 | 1 | 0.886693 | 0.877326 |
| GOTERM_MF_ALL | GO:0003714~transcription corepressor activity | 10 | 2.336449 | 0.041908 | TGIF1, DRAP1, HEYL, TONSL, MAP3K10, TRIB3, NR0B2, FOXP3, RELB, HOXC6 | 344 | 226 | 16881 | 2.171357 | 1 | 0.886693 | 0.877326 |
| GOTERM_MF_ALL | GO:0003700~transcription factor activity, sequence-specific DNA binding | 35 | 8.17757 | 0.041914 | MESP1, CEBPB, UHRF1, FOXO6, SCX, ASCL1, ASCL2, RELB, NME1-NME2, PTTG1, PCBP3, HEY1, SOX18, SALL4, ZXDC, E2F1, MYBL2, MNX1, HOXC6, TGIF1, TEAD4, SMAD9, ARID3A, SOX12, BHLHA15, ETV4, FOXP3, ST18, DRAP1, HEYL, RFX6, MAFG, HOXB2, INSM1, FOXA2 | 344 | 1231 | 16881 | 1.395243 | 1 | 0.886693 | 0.877326 |
| GOTERM_MF_ALL | GO:0001071~nucleic acid binding transcription factor activity | 35 | 8.17757 | 0.042669 | MESP1, CEBPB, UHRF1, FOXO6, SCX, ASCL1, ASCL2, RELB, NME1-NME2, PTTG1, PCBP3, HEY1, SOX18, SALL4, ZXDC, E2F1, MYBL2, MNX1, HOXC6, TGIF1, TEAD4, SMAD9, ARID3A, SOX12, BHLHA15, ETV4, FOXP3, ST18, DRAP1, HEYL, RFX6, MAFG, HOXB2, INSM1, FOXA2 | 344 | 1232 | 16881 | 1.39411 | 1 | 0.886693 | 0.877326 |
| GOTERM_MF_ALL | GO:0017111~nucleoside-triphosphatase activity | 24 | 5.607477 | 0.047256 | GINS2, RTEL1-TNFRSF6B, GNAZ, ATP6V0B, CLPB, ABCC8, NUDT1, ATP1A3, ABCA7, MYL6B, MYO7A, ATP11A, RND1, RECQL4, KIF18B, KIFC2, DHX34, FXYD2, DHX37, RAD54L, KIF2C, KIF1A, ABCG1, MCM2 | 344 | 779 | 16881 | 1.511867 | 1 | 0.916793 | 0.907108 |
| GOTERM_MF_ALL | GO:0043492~ATPase activity, coupled to movement of substances | 7 | 1.635514 | 0.048103 | ATP6V0B, ABCC8, FXYD2, ATP1A3, ABCA7, ATP11A, ABCG1 | 344 | 129 | 16881 | 2.662858 | 1 | 0.916793 | 0.907108 |
| GOTERM_MF_ALL | GO:0005261~cation channel activity | 12 | 2.803738 | 0.048339 | CHRNB2, FAM155B, KCNH2, TRPM2, KCNJ11, KCNH6, ABCC8, TMEM63C, ORAI1, CACNA1D, KCNAB2, KCNK3 | 344 | 306 | 16881 | 1.924419 | 1 | 0.916793 | 0.907108 |
| GOTERM_MF_ALL | GO:0016462~pyrophosphatase activity | 25 | 5.841121 | 0.049652 | GNAZ, CLPB, NUDT1, ATP1A3, MYL6B, RND1, RECQL4, TRPM2, DHX34, DHX37, RAD54L, KIF1A, GINS2, RTEL1-TNFRSF6B, ATP6V0B, ABCC8, ABCA7, ATP11A, MYO7A, KIF18B, KIFC2, FXYD2, KIF2C, ABCG1, MCM2 | 344 | 824 | 16881 | 1.488855 | 1 | 0.916793 | 0.907108 |
| GOTERM_MF_ALL | GO:0019212~phosphatase inhibitor activity | 4 | 0.934579 | 0.050263 | PPP1R14B, PPP1R14C, TESC, PPP1R35 | 344 | 41 | 16881 | 4.787578 | 1 | 0.916793 | 0.907108 |
| GOTERM_MF_ALL | GO:0016818~hydrolase activity, acting on acid anhydrides, in phosphorus-containing anhydrides | 25 | 5.841121 | 0.050574 | GNAZ, CLPB, NUDT1, ATP1A3, MYL6B, RND1, RECQL4, TRPM2, DHX34, DHX37, RAD54L, KIF1A, GINS2, RTEL1-TNFRSF6B, ATP6V0B, ABCC8, ABCA7, ATP11A, MYO7A, KIF18B, KIFC2, FXYD2, KIF2C, ABCG1, MCM2 | 344 | 826 | 16881 | 1.48525 | 1 | 0.916793 | 0.907108 |
| GOTERM_MF_ALL | GO:0016817~hydrolase activity, acting on acid anhydrides | 25 | 5.841121 | 0.052376 | GNAZ, CLPB, NUDT1, ATP1A3, MYL6B, RND1, RECQL4, TRPM2, DHX34, DHX37, RAD54L, KIF1A, GINS2, RTEL1-TNFRSF6B, ATP6V0B, ABCC8, ABCA7, ATP11A, MYO7A, KIF18B, KIFC2, FXYD2, KIF2C, ABCG1, MCM2 | 344 | 828 | 16881 | 1.481663 | 1 | 0.929677 | 0.919857 |
| GOTERM_MF_ALL | GO:0022843~voltage-gated cation channel activity | 7 | 1.635514 | 0.054302 | KCNH2, KCNJ11, KCNH6, ABCC8, CACNA1D, KCNAB2, KCNK3 | 344 | 133 | 16881 | 2.582772 | 1 | 0.944196 | 0.934222 |
| GOTERM_MF_ALL | GO:0022892~substrate-specific transporter activity | 32 | 7.476636 | 0.058142 | TTYH3, TMEM63C, ATP1A3, CACNA1D, SLC38A11, TIMM10, SLC1A5, COX6A1, SLC7A1, AQP3, IPO4, TRPM2, SLC25A22, SLC18A1, SLC39A3, KCNH2, CHRNB2, ATP6V0B, KCNH6, KCNJ11, ABCC8, APOA2, ABCA7, KCNAB2, ATP11A, FAM155B, SLCO3A1, FXYD2, ORAI1, SLC29A4, KCNK3, ABCG1 | 344 | 1137 | 16881 | 1.381113 | 1 | 0.990747 | 0.980281 |
| GOTERM_MF_ALL | GO:0035939~microsatellite binding | 2 | 0.46729 | 0.059729 | HEYL, HEY1 | 344 | 3 | 16881 | 32.71512 | 1 | 0.997832 | 0.987292 |
| GOTERM_MF_ALL | GO:0042623~ATPase activity, coupled | 12 | 2.803738 | 0.069167 | RECQL4, RTEL1-TNFRSF6B, ATP6V0B, ABCC8, DHX34, FXYD2, DHX37, ATP1A3, ABCA7, ATP11A, MYO7A, ABCG1 | 344 | 326 | 16881 | 1.806356 | 1 | 1 | 0.990599 |
| GOTERM_MF_ALL | GO:0001228~transcriptional activator activity, RNA polymerase II transcription regulatory region sequence-specific binding | 12 | 2.803738 | 0.069167 | MESP1, HEYL, CEBPB, RFX6, SOX18, MAFG, SCX, ARID3A, MYBL2, SOX12, BHLHA15, ETV4 | 344 | 326 | 16881 | 1.806356 | 1 | 1 | 0.990599 |
| GOTERM_MF_ALL | GO:0022838~substrate-specific channel activity | 15 | 3.504673 | 0.071246 | KCNH2, CHRNB2, TTYH3, KCNH6, KCNJ11, TMEM63C, ABCC8, CACNA1D, KCNAB2, AQP3, FAM155B, TRPM2, FXYD2, ORAI1, KCNK3 | 344 | 445 | 16881 | 1.654135 | 1 | 1 | 0.990599 |
| GOTERM_MF_ALL | GO:0005242~inward rectifier potassium channel activity | 3 | 0.700935 | 0.072826 | KCNH2, KCNJ11, ABCC8 | 344 | 22 | 16881 | 6.691728 | 1 | 1 | 0.990599 |
| GOTERM_MF_ALL | GO:0097367~carbohydrate derivative binding | 56 | 13.08411 | 0.077303 | SPON2, SCARB1, SERPINA1, RTKN, DYRK2, CLPB, PODXL2, GPAA1, SERPINA10, SHB, AACS, RND1, KIF1A, EPHB3, MAP4K2, RPS6KL1, LIG1, ABCC8, MYO7A, ATP11A, PGF, LOC102724428, KIFC2, DPEP1, CHI3L1, TRIB3, SIK1, KIF2C, ABCG1, STX1A, MCM2, GNAZ, PRKDC, ATP1A3, PKMYT1, AURKA, NME1-NME2, RECQL4, VTN, ADGRG1, MTHFD1L, DHX34, RAB26, DHX37, RAD54L, RTEL1-TNFRSF6B, PLK3, KCNJ11, UBE2C, ABCA7, KIF18B, UBE2S, RAB15, CDK4, MAP3K10, FGFR4 | 344 | 2241 | 16881 | 1.226269 | 1 | 1 | 0.990599 |
| GOTERM_MF_ALL | GO:0022836~gated channel activity | 12 | 2.803738 | 0.078485 | CHRNB2, FAM155B, KCNH2, TRPM2, TTYH3, KCNJ11, KCNH6, ABCC8, TMEM63C, CACNA1D, KCNAB2, KCNK3 | 344 | 334 | 16881 | 1.76309 | 1 | 1 | 0.990599 |
| GOTERM_MF_ALL | GO:0042626~ATPase activity, coupled to transmembrane movement of substances | 6 | 1.401869 | 0.082943 | ATP6V0B, ABCC8, FXYD2, ATP1A3, ABCA7, ABCG1 | 344 | 114 | 16881 | 2.582772 | 1 | 1 | 0.990599 |
| GOTERM_MF_ALL | GO:0016820~hydrolase activity, acting on acid anhydrides, catalyzing transmembrane movement of substances | 6 | 1.401869 | 0.087795 | ATP6V0B, ABCC8, FXYD2, ATP1A3, ABCA7, ABCG1 | 344 | 116 | 16881 | 2.538242 | 1 | 1 | 0.990599 |
| GOTERM_MF_ALL | GO:0004860~protein kinase inhibitor activity | 5 | 1.168224 | 0.094781 | SOCS1, CDKN2A, TESC, TRIB3, MAPK8IP1 | 344 | 85 | 16881 | 2.886628 | 1 | 1 | 0.990599 |
| GOTERM_MF_ALL | GO:0043425~bHLH transcription factor binding | 3 | 0.700935 | 0.097247 | MAP3K10, SCX, ASCL1 | 344 | 26 | 16881 | 5.662232 | 1 | 1 | 0.990599 |
| GOTERM_MF_ALL | GO:0015272~ATP-activated inward rectifier potassium channel activity | 2 | 0.46729 | 0.097559 | KCNJ11, ABCC8 | 344 | 5 | 16881 | 19.62907 | 1 | 1 | 0.990599 |
| GOTERM_MF_ALL | GO:0005267~potassium channel activity | 6 | 1.401869 | 0.097933 | KCNH2, KCNJ11, KCNH6, ABCC8, KCNAB2, KCNK3 | 344 | 120 | 16881 | 2.453634 | 1 | 1 | 0.990599 |
| GOTERM_MF_ALL | GO:0005488~binding | 302 | 70.56075 | 0.099137 | SPON2, ZNF296, CLPB, CPNE7, HDAC11, PLEKHB1, SERPINE1, PPAN, TONSL, NUDT1, SCX, STMN3, TPGS1, RRP9, SCAMP5, LIPF, CDC20, LIPE, RPS19, SOX18, ZXDC, ARL6IP4, MNX1, EPHB3, CHGB, IER3, SDS, UNC13A, NUP210, SERPINF2, SOX12, MYO7A, BHLHA15, CPT1C, MAPK8IP2, MAPK8IP1, SULT2B1, EID2, HOXB9, CLDN3, RFX6, CCDC86, ORAI1, SIK1, HOXB2, FAM64A, TRIB3, PLPP2, STX1A, BEX2, TPH1, BEX1, SHMT2, PRKDC, PPP1R35, CDCA5, C2CD4A, C2CD4B, C2CD4C, WDR62, NCAPH, C2, TRPM2, ZNHIT2, ADAMTS14, PCBP3, DHX34, MIER2, DHX37, HOXC9, HOXC6, RTEL1-TNFRSF6B, CDT1, PLK3, STARD10, ABCA7, NBEAL2, NR0B2, ST18, PARP10, NDUFAF8, ARTN, PAH, MESP1, SERPINA1, GPAA1, MRPS12, SERPINA10, HEPACAM2, BICDL1, MYL6B, SHB, HOXC10, ASGR1, AACS, HEY1, ADAMTSL2, FFAR2, KIF1A, TEAD4, MAP4K2, RPS6KL1, MYOC, GP1BB, TESC, SYP, MTSS1, PDRG1, MMP11, CTU1, KIFC2, INCENP, VWA1, KIF2C, PAFAH1B3, GRAMD1A, TIMM10, GDPD5, ASCL1, CENPA, CRIP1, SLC7A1, ASCL2, RELB, RECQL4, CST3, CST1, NPW, MTHFD1L, UCP2, RAD54L, PRAME, ZSWIM4, DTD1, GINS2, NPM2, ZNF580, CDKN2A, GDF15, IL34, GAD1, STAC3, NPM3, AGT, GUCA2A, MAD2L2, SH3RF3, HEYL, FABP4, TMEM158, SMOC1, SHKBP1, TACC3, CDK5R2, FOXA2, EXOC3L1, SCARB1, RTKN, CRTC1, GALNT18, REG1B, REG1A, MSI1, OGDHL, KLHL35, IPO4, PTTG1, SALL4, TXNL4A, PPFIA3, CHRNB2, KCNH2, DGAT2, LIG1, KMT5C, FBXW9, ATP11A, RUNDC3A, FOXP3, SRCIN1, PGF, TRAIP, CDC25B, LOC102724428, CCNE1, TIMELESS, DPEP1, ANAPC5, TSNARE1, RELL2, RPP25, ABCG1, GNAZ, REG3A, SHC2, RNASEH2A, TROAP, FOXO6, CACNA1D, SLC1A5, CPLX2, PKMYT1, CPLX1, TIMM50, NME1-NME2, VTN, ADGRG1, PCSK1N, SOCS1, ZNF428, RHPN1, HYI, H1FX, C16ORF59, BMF, CYC1, SAC3D1, UBE2C, SLC52A2, CBX2, ASPSCR1, CYBRD1, SMAD9, SYT7, DRAP1, SDK1, AMACR, KIF18B, UBE2S, CDK4, MAFG, MAP3K10, SP5, MELTF, FGFR4, ULBP2, RNF187, SNAP25, DAGLA, DYRK2, NXT1, PYCRL, PODXL2, ZC3H3, CELF4, RND1, SPR, DACT2, MYBL2, CA8, GRASP, TGIF1, PLEKHG4, EMX1, ABCC8, MMP1, H2AFX, APOA2, ADRA2C, ETV4, VGF, CHI3L1, PFDN2, MCM2, CEBPB, NAXE, UHRF1, RGS16, LRP5, ATP1A3, RRBP1, DBF4B, LAGE3, CLN6, SRM, AURKA, DLL3, DLL4, P2RY6, TTR, PARD6A, PUF60, GNG4, RAB26, POLD1, NT5DC2, E2F1, POLR2I, CTHRC1, HES4, CMTM8, KCNJ11, CADPS, ARID3A, CCK, NFASC, PRC1, RAB15, APOC1, NEURL1, INSM1, KCNK3 | 344 | 14381 | 16881 | 1.030523 | 1 | 1 | 0.990599 |
| GOTERM_MF_ALL | GO:0005216~ion channel activity | 14 | 3.271028 | 0.099558 | KCNH2, CHRNB2, TTYH3, KCNH6, KCNJ11, TMEM63C, ABCC8, CACNA1D, KCNAB2, FAM155B, TRPM2, FXYD2, ORAI1, KCNK3 | 344 | 429 | 16881 | 1.601439 | 1 | 1 | 0.990599 |

| **Table S4: Gene Ontology-Cellular Component (GO-CC) Functional Enrichment Results using DAVID** | | | | | | | | | | | |  |
| --- | --- | --- | --- | --- | --- | --- | --- | --- | --- | --- | --- | --- |
|  |  |  |  |  |  |  |  |  |  |  |  |  |
| Category | Term | Count | % | PValue | Genes | List Total | Pop Hits | Pop Total | Fold Enrichment | Bonferroni | Benjamini | FDR |
| GOTERM_CC_ALL | GO:0033267~axon part | 18 | 4.205607 | 2.03E-06 | PTPRN2, DAGLA, UNC13A, MYOC, KCNJ11, CCK, KCNAB2, SYP, ADRA2C, CPLX2, AP3B2, CPLX1, SYT7, AURKA, MAPK8IP1, NFASC, KIF1A, SLC18A1 | 366 | 218 | 18224 | 4.111295 | 0.001118 | 0.001119 | 0.001094 |
| GOTERM_CC_ALL | GO:0030424~axon | 25 | 5.841121 | 4.18E-06 | DAGLA, ATP1A3, STMN3, GDPD5, TPGS1, CPLX2, CPLX1, AURKA, CST3, KIF1A, SLC18A1, PTPRN2, UNC13A, MYOC, KCNJ11, CCK, KCNAB2, SYP, ADRA2C, CPT1C, SRCIN1, AP3B2, SYT7, MAPK8IP1, NFASC | 366 | 418 | 18224 | 2.978011 | 0.002298 | 0.00115 | 0.001125 |
| GOTERM_CC_ALL | GO:0043195~terminal bouton | 8 | 1.869159 | 2.58E-04 | UNC13A, PTPRN2, CCK, SYP, CPLX2, SLC18A1, CPLX1, SYT7 | 366 | 63 | 18224 | 6.322838 | 0.132323 | 0.047306 | 0.046276 |
| GOTERM_CC_ALL | GO:0043679~axon terminus | 10 | 2.336449 | 4.50E-04 | UNC13A, PTPRN2, CCK, KCNAB2, SYP, ADRA2C, CPLX2, SLC18A1, CPLX1, SYT7 | 366 | 113 | 18224 | 4.406403 | 0.21965 | 0.061989 | 0.060639 |
| GOTERM_CC_ALL | GO:0044306~neuron projection terminus | 10 | 2.336449 | 9.35E-04 | UNC13A, PTPRN2, CCK, KCNAB2, SYP, ADRA2C, CPLX2, SLC18A1, CPLX1, SYT7 | 366 | 125 | 18224 | 3.983388 | 0.402776 | 0.079389 | 0.07766 |
| GOTERM_CC_ALL | GO:0005680~anaphase-promoting complex | 5 | 1.168224 | 0.001037 | MAD2L2, CDC20, UBE2C, UBE2S, ANAPC5 | 366 | 23 | 18224 | 10.82442 | 0.43536 | 0.079389 | 0.07766 |
| GOTERM_CC_ALL | GO:0005578~proteinaceous extracellular matrix | 18 | 4.205607 | 0.001041 | SPON2, SERPINA1, MYOC, MMP1, COL22A1, DPT, CST3, MMP11, VTN, ADAMTS14, SMOC1, MFAP2, ADAMTSL2, VWA1, CHI3L1, RELL2, DGCR6, CTHRC1 | 366 | 361 | 18224 | 2.482721 | 0.436644 | 0.079389 | 0.07766 |
| GOTERM_CC_ALL | GO:0045202~synapse | 29 | 6.775701 | 0.001345 | SNAP25, PRR7, CRTC1, ATP1A3, CPLX2, CPLX1, SCAMP5, SLC18A1, GRASP, PPFIA3, CHRNB2, PTPRN2, UNC13A, ABCC8, GAD1, CADPS, CCK, KCNAB2, SYP, MYO7A, CPT1C, MAPK8IP2, SRCIN1, SYT7, MAPK8IP1, SDK1, NEURL1, RIMBP2, STX1A | 366 | 755 | 18224 | 1.912554 | 0.523522 | 0.079389 | 0.07766 |
| GOTERM_CC_ALL | GO:0099501~exocytic vesicle membrane | 7 | 1.635514 | 0.001481 | SCAMP5, PTPRN2, ABCC8, SYP, SLC18A1, STX1A, SYT7 | 366 | 62 | 18224 | 5.621717 | 0.55807 | 0.079389 | 0.07766 |
| GOTERM_CC_ALL | GO:0030672~synaptic vesicle membrane | 7 | 1.635514 | 0.001481 | SCAMP5, PTPRN2, ABCC8, SYP, SLC18A1, STX1A, SYT7 | 366 | 62 | 18224 | 5.621717 | 0.55807 | 0.079389 | 0.07766 |
| GOTERM_CC_ALL | GO:0000152~nuclear ubiquitin ligase complex | 6 | 1.401869 | 0.001615 | MAD2L2, CDC20, UBE2C, UBE2S, CBX2, ANAPC5 | 366 | 43 | 18224 | 6.94777 | 0.589679 | 0.079389 | 0.07766 |
| GOTERM_CC_ALL | GO:0098793~presynapse | 15 | 3.504673 | 0.001729 | SNAP25, PTPRN2, UNC13A, ABCC8, GAD1, CADPS, CCK, SYP, CPLX2, CPLX1, SYT7, SCAMP5, SLC18A1, STX1A, PPFIA3 | 366 | 282 | 18224 | 2.648529 | 0.614607 | 0.079389 | 0.07766 |
| GOTERM_CC_ALL | GO:0043005~neuron projection | 34 | 7.943925 | 0.002027 | SNAP25, GNAZ, TPH1, DAGLA, CRTC1, ATP1A3, STMN3, GDPD5, TPGS1, CPLX2, CPLX1, AURKA, CST3, TRPM2, KIF1A, SLC18A1, EPHB3, PLK3, PTPRN2, UNC13A, MYOC, KCNJ11, CCK, KCNAB2, SYP, ADRA2C, CPT1C, SRCIN1, AP3B2, SYT7, MAPK8IP1, NFASC, NEURL1, STX1A | 366 | 966 | 18224 | 1.752526 | 0.673144 | 0.085931 | 0.084059 |
| GOTERM_CC_ALL | GO:0070032~synaptobrevin 2-SNAP-25-syntaxin-1a-complexin I complex | 3 | 0.700935 | 0.002337 | SNAP25, CPLX1, STX1A | 366 | 4 | 18224 | 37.34426 | 0.724524 | 0.087882 | 0.085968 |
| GOTERM_CC_ALL | GO:0097458~neuron part | 43 | 10.04673 | 0.002392 | SNAP25, GNAZ, TPH1, DAGLA, CRTC1, ATP1A3, STMN3, GDPD5, TPGS1, SEZ6L2, ASCL1, CPLX2, CPLX1, AURKA, SCAMP5, CST3, TRPM2, KIF1A, SLC18A1, PPFIA3, EPHB3, PLK3, PTPRN2, UNC13A, MYOC, KCNJ11, ABCC8, GAD1, CADPS, CCK, KCNAB2, SYP, MYO7A, ADRA2C, CPT1C, MAPK8IP2, SRCIN1, AP3B2, SYT7, MAPK8IP1, NFASC, NEURL1, STX1A | 366 | 1333 | 18224 | 1.606205 | 0.732815 | 0.087882 | 0.085968 |
| GOTERM_CC_ALL | GO:0031201~SNARE complex | 6 | 1.401869 | 0.004803 | SCAMP5, SNAP25, TSNARE1, CPLX2, CPLX1, STX1A | 366 | 55 | 18224 | 5.431893 | 0.92955 | 0.165405 | 0.161803 |
| GOTERM_CC_ALL | GO:0031012~extracellular matrix | 21 | 4.906542 | 0.0052 | SPON2, SERPINA1, MYOC, PRKDC, MMP1, COL22A1, SERPINE1, DPT, CST3, MMP11, VTN, ADAMTS14, RPS19, SMOC1, MFAP2, ADAMTSL2, VWA1, CHI3L1, RELL2, DGCR6, CTHRC1 | 366 | 530 | 18224 | 1.972904 | 0.943455 | 0.168544 | 0.164873 |
| GOTERM_CC_ALL | GO:0044456~synapse part | 23 | 5.373832 | 0.005609 | CHRNB2, SNAP25, PRR7, PTPRN2, UNC13A, ABCC8, GAD1, CADPS, ATP1A3, CCK, KCNAB2, SYP, MAPK8IP2, CPLX2, SRCIN1, CPLX1, SYT7, SCAMP5, NEURL1, SLC18A1, GRASP, STX1A, PPFIA3 | 366 | 607 | 18224 | 1.886695 | 0.954924 | 0.171706 | 0.167966 |
| GOTERM_CC_ALL | GO:0099503~secretory vesicle | 19 | 4.439252 | 0.0064 | PCSK1, EXOC3L1, SNAP25, PTPRN2, SERPINA1, ABCC8, SERPINF2, SERPINE1, NUDT1, SYP, SYT7, SCAMP5, PCSK1N, RAB26, BMF, SLC18A1, STX1A, PPFIA3, CHGB | 366 | 467 | 18224 | 2.025813 | 0.970916 | 0.173231 | 0.169458 |
| GOTERM_CC_ALL | GO:0044304~main axon | 6 | 1.401869 | 0.006954 | NFASC, DAGLA, KCNJ11, MYOC, CCK, KCNAB2 | 366 | 60 | 18224 | 4.979235 | 0.978618 | 0.173231 | 0.169458 |
| GOTERM_CC_ALL | GO:1990351~transporter complex | 15 | 3.504673 | 0.006971 | KCNH2, CHRNB2, SNAP25, TTYH3, KCNJ11, ABCC8, ATP1A3, CACNA1D, ABCA7, KCNAB2, TIMM10, CPT1C, FXYD2, CYC1, STX1A | 366 | 330 | 18224 | 2.263289 | 0.978816 | 0.173231 | 0.169458 |
| GOTERM_CC_ALL | GO:0000785~chromatin | 19 | 4.439252 | 0.007236 | CEBPB, NPM2, CDKN2A, UHRF1, KMT5C, CDCA5, CBX2, H2AFX, DBF4B, CENPA, IPO4, INCENP, SOX18, CDK4, SALL4, E2F1, TIMELESS, H1FX, MCM2 | 366 | 472 | 18224 | 2.004353 | 0.981717 | 0.173231 | 0.169458 |
| GOTERM_CC_ALL | GO:0031974~membrane-enclosed lumen | 111 | 25.93458 | 0.007292 | CRTC1, HDAC11, SERPINE1, PPAN, NUDT1, TONSL, OGDHL, IPO4, RRP9, CDC20, RPS19, SOX18, SALL4, ARL6IP4, TXNL4A, MNX1, LIG1, KMT5C, SERPINF2, SOX12, TRAIP, CDC25B, EID2, HOXB9, CCNE1, TIMELESS, CCDC86, HOXB2, TRIB3, FAM64A, ANAPC5, RPP25, ABCG1, RNASEH2A, SHMT2, PRKDC, CDCA5, C2CD4B, PKMYT1, TIMM50, VTN, DHX37, H1FX, HOXC6, CDT1, PLK3, PTPRN2, UBE2C, CBX2, ASPSCR1, SMAD9, PGA3, NR0B2, PGA5, PGA4, PARP10, AMACR, CDK4, MAFG, FGFR4, RNF187, PCSK1, SERPINA1, NXT1, DYRK2, ZC3H3, MRPS12, HOXC10, DUSP10, SPR, HEY1, MYBL2, TEAD4, TGIF1, MYOC, H2AFX, APOA2, ETV4, AP3B2, MMP11, INCENP, PFDN2, MCM2, CEBPB, NAXE, UHRF1, DBF4B, TIMM10, CENPA, CLN6, RELB, AURKA, PUF60, MTHFD1L, POLD1, E2F1, RAD54L, POLR2I, SLC25A22, CMTM8, GINS2, NPM2, CDKN2A, COL22A1, ARID3A, NPM3, MAD2L2, HEYL, PRC1, INSM1, FOXA2 | 366 | 4457 | 18224 | 1.240061 | 0.982275 | 0.173231 | 0.169458 |
| GOTERM_CC_ALL | GO:0097486~multivesicular body lumen | 3 | 0.700935 | 0.00786 | PGA3, PGA5, PGA4 | 366 | 7 | 18224 | 21.33958 | 0.987066 | 0.173231 | 0.169458 |
| GOTERM_CC_ALL | GO:0031906~late endosome lumen | 3 | 0.700935 | 0.00786 | PGA3, PGA5, PGA4 | 366 | 7 | 18224 | 21.33958 | 0.987066 | 0.173231 | 0.169458 |
| GOTERM_CC_ALL | GO:0044428~nuclear part | 100 | 23.36449 | 0.009321 | CRTC1, HDAC11, PPAN, NUDT1, TONSL, IPO4, RRP9, CDC20, RPS19, SOX18, SALL4, ARL6IP4, TXNL4A, MNX1, LIG1, NUP210, KMT5C, SOX12, TRAIP, CDC25B, EID2, HOXB9, CCNE1, TIMELESS, CCDC86, HOXB2, TRIB3, FAM64A, ANAPC5, RPP25, ABCG1, GNAZ, RNASEH2A, SHMT2, PRKDC, CDCA5, C2CD4B, PKMYT1, TIMM50, DHX37, H1FX, HOXC6, CDT1, PLK3, UBE2C, CBX2, ASPSCR1, SMAD9, NR0B2, PARP10, UBE2S, CDK4, MAFG, FGFR4, RNF187, NXT1, DYRK2, ZC3H3, HOXC10, DUSP10, SPR, HEY1, MYBL2, TEAD4, TGIF1, H2AFX, ETV4, AP3B2, INCENP, PFDN2, MCM2, CEBPB, NAXE, UHRF1, DBF4B, ASCL1, CENPA, ASCL2, RELB, AURKA, CST3, PUF60, POLD1, E2F1, RAD54L, POLR2I, SLC25A22, CMTM8, GINS2, NPM2, KCNJ11, CDKN2A, TMEM176B, ARID3A, NPM3, MAD2L2, HEYL, PRC1, INSM1, FOXA2 | 366 | 3981 | 18224 | 1.25075 | 0.994257 | 0.191214 | 0.187049 |
| GOTERM_CC_ALL | GO:0043233~organelle lumen | 109 | 25.46729 | 0.00937 | CRTC1, HDAC11, SERPINE1, PPAN, NUDT1, TONSL, OGDHL, IPO4, RRP9, CDC20, RPS19, SOX18, SALL4, ARL6IP4, TXNL4A, MNX1, LIG1, KMT5C, SERPINF2, SOX12, TRAIP, CDC25B, EID2, HOXB9, CCNE1, TIMELESS, CCDC86, HOXB2, TRIB3, FAM64A, ANAPC5, RPP25, ABCG1, RNASEH2A, SHMT2, PRKDC, CDCA5, C2CD4B, PKMYT1, TIMM50, VTN, DHX37, H1FX, HOXC6, CDT1, PLK3, PTPRN2, UBE2C, CBX2, ASPSCR1, SMAD9, PGA3, NR0B2, PGA5, PGA4, PARP10, AMACR, CDK4, MAFG, FGFR4, RNF187, PCSK1, SERPINA1, NXT1, DYRK2, ZC3H3, MRPS12, HOXC10, DUSP10, SPR, HEY1, MYBL2, TEAD4, TGIF1, H2AFX, APOA2, ETV4, AP3B2, MMP11, INCENP, PFDN2, MCM2, CEBPB, NAXE, UHRF1, DBF4B, CENPA, CLN6, RELB, AURKA, PUF60, MTHFD1L, POLD1, E2F1, RAD54L, POLR2I, SLC25A22, CMTM8, GINS2, NPM2, CDKN2A, COL22A1, ARID3A, NPM3, MAD2L2, HEYL, PRC1, INSM1, FOXA2 | 366 | 4400 | 18224 | 1.233492 | 0.994412 | 0.191214 | 0.187049 |
| GOTERM_CC_ALL | GO:0005654~nucleoplasm | 78 | 18.2243 | 0.010955 | NXT1, DYRK2, CRTC1, ZC3H3, HDAC11, TONSL, HOXC10, RRP9, CDC20, DUSP10, HEY1, SPR, RPS19, SALL4, ARL6IP4, MYBL2, TXNL4A, TGIF1, TEAD4, LIG1, KMT5C, H2AFX, SOX12, AP3B2, ETV4, CDC25B, EID2, HOXB9, CCNE1, INCENP, TIMELESS, HOXB2, TRIB3, PFDN2, ANAPC5, RPP25, ABCG1, MCM2, CEBPB, RNASEH2A, NAXE, PRKDC, CDCA5, DBF4B, PKMYT1, CENPA, AURKA, TIMM50, RELB, PUF60, POLD1, DHX37, E2F1, RAD54L, POLR2I, SLC25A22, HOXC6, GINS2, CDT1, PLK3, CMTM8, CDKN2A, UBE2C, CBX2, ASPSCR1, SMAD9, ARID3A, NR0B2, PARP10, MAD2L2, HEYL, PRC1, CDK4, MAFG, INSM1, FGFR4, RNF187, FOXA2 | 366 | 2996 | 18224 | 1.29633 | 0.997688 | 0.215577 | 0.210882 |
| GOTERM_CC_ALL | GO:1902495~transmembrane transporter complex | 14 | 3.271028 | 0.014058 | KCNH2, CHRNB2, SNAP25, TTYH3, KCNJ11, ABCC8, ATP1A3, CACNA1D, ABCA7, KCNAB2, CPT1C, FXYD2, CYC1, STX1A | 366 | 324 | 18224 | 2.151521 | 0.999591 | 0.253662 | 0.248138 |
| GOTERM_CC_ALL | GO:0008021~synaptic vesicle | 8 | 1.869159 | 0.014081 | SCAMP5, SNAP25, PTPRN2, ABCC8, SYP, SLC18A1, STX1A, SYT7 | 366 | 127 | 18224 | 3.136526 | 0.999596 | 0.253662 | 0.248138 |
| GOTERM_CC_ALL | GO:0000792~heterochromatin | 6 | 1.401869 | 0.014684 | KMT5C, INCENP, CDKN2A, UHRF1, CBX2, SALL4 | 366 | 72 | 18224 | 4.149362 | 0.999711 | 0.253662 | 0.248138 |
| GOTERM_CC_ALL | GO:0070013~intracellular organelle lumen | 106 | 24.76636 | 0.014732 | CRTC1, HDAC11, PPAN, NUDT1, TONSL, OGDHL, IPO4, RRP9, CDC20, RPS19, SOX18, SALL4, ARL6IP4, TXNL4A, MNX1, LIG1, KMT5C, SOX12, TRAIP, CDC25B, EID2, HOXB9, CCNE1, TIMELESS, CCDC86, HOXB2, TRIB3, FAM64A, ANAPC5, RPP25, ABCG1, RNASEH2A, SHMT2, PRKDC, CDCA5, C2CD4B, PKMYT1, TIMM50, VTN, DHX37, H1FX, HOXC6, CDT1, PLK3, PTPRN2, UBE2C, CBX2, ASPSCR1, SMAD9, PGA3, NR0B2, PGA5, PGA4, PARP10, AMACR, CDK4, MAFG, FGFR4, RNF187, SERPINA1, NXT1, DYRK2, ZC3H3, MRPS12, HOXC10, DUSP10, SPR, HEY1, MYBL2, TEAD4, TGIF1, H2AFX, APOA2, ETV4, AP3B2, MMP11, INCENP, PFDN2, MCM2, CEBPB, NAXE, UHRF1, DBF4B, CENPA, CLN6, RELB, AURKA, PUF60, MTHFD1L, POLD1, E2F1, RAD54L, POLR2I, SLC25A22, CMTM8, GINS2, NPM2, CDKN2A, COL22A1, ARID3A, NPM3, MAD2L2, HEYL, PRC1, INSM1, FOXA2 | 366 | 4328 | 18224 | 1.219498 | 0.999719 | 0.253662 | 0.248138 |
| GOTERM_CC_ALL | GO:0031981~nuclear lumen | 90 | 21.02804 | 0.01552 | NXT1, DYRK2, CRTC1, ZC3H3, HDAC11, PPAN, TONSL, HOXC10, IPO4, RRP9, CDC20, DUSP10, HEY1, SPR, RPS19, SOX18, SALL4, ARL6IP4, MYBL2, TXNL4A, MNX1, TGIF1, TEAD4, LIG1, KMT5C, H2AFX, SOX12, AP3B2, ETV4, TRAIP, CDC25B, EID2, HOXB9, CCNE1, INCENP, TIMELESS, CCDC86, HOXB2, TRIB3, FAM64A, PFDN2, ANAPC5, RPP25, ABCG1, MCM2, CEBPB, RNASEH2A, NAXE, UHRF1, PRKDC, CDCA5, C2CD4B, DBF4B, PKMYT1, CENPA, AURKA, TIMM50, RELB, PUF60, POLD1, DHX37, E2F1, RAD54L, H1FX, POLR2I, SLC25A22, HOXC6, GINS2, CDT1, PLK3, CMTM8, NPM2, CDKN2A, UBE2C, CBX2, ASPSCR1, SMAD9, ARID3A, NPM3, NR0B2, PARP10, MAD2L2, HEYL, PRC1, CDK4, MAFG, INSM1, FGFR4, RNF187, FOXA2 | 366 | 3592 | 18224 | 1.247581 | 0.999819 | 0.259014 | 0.253373 |
| GOTERM_CC_ALL | GO:0044427~chromosomal part | 27 | 6.308411 | 0.015983 | CEBPB, UHRF1, PRKDC, CDCA5, TONSL, DBF4B, CENPA, IPO4, NCAPH, AURKA, SOX18, SALL4, POLD1, E2F1, H1FX, MYBL2, GINS2, NPM2, CDKN2A, KMT5C, CBX2, H2AFX, INCENP, CDK4, TIMELESS, KIF2C, MCM2 | 366 | 827 | 18224 | 1.625627 | 0.999861 | 0.259014 | 0.253373 |
| GOTERM_CC_ALL | GO:0044297~cell body | 18 | 4.205607 | 0.01721 | PLK3, GNAZ, NAXE, KCNJ11, CRTC1, CCK, SEZ6L2, GDPD5, ASCL1, MAPK8IP2, CPLX2, SRCIN1, CPLX1, AURKA, MAPK8IP1, CST3, TRPM2, NEURL1 | 366 | 480 | 18224 | 1.867213 | 0.99993 | 0.267298 | 0.261476 |
| GOTERM_CC_ALL | GO:0044454~nuclear chromosome part | 19 | 4.439252 | 0.017464 | GINS2, CEBPB, NPM2, UHRF1, KMT5C, PRKDC, CDCA5, H2AFX, TONSL, DBF4B, CENPA, IPO4, AURKA, INCENP, SOX18, POLD1, E2F1, TIMELESS, MCM2 | 366 | 519 | 18224 | 1.822841 | 0.999939 | 0.267298 | 0.261476 |
| GOTERM_CC_ALL | GO:0048786~presynaptic active zone | 4 | 0.934579 | 0.021688 | UNC13A, GAD1, SYP, PPFIA3 | 366 | 30 | 18224 | 6.63898 | 0.999994 | 0.315295 | 0.308428 |
| GOTERM_CC_ALL | GO:0016023~cytoplasmic, membrane-bounded vesicle | 35 | 8.17757 | 0.021744 | PCSK1, EXOC3L1, SNAP25, SCARB1, SERPINA1, SERPINE1, NUDT1, SLC1A5, SCAMP5, TRPM2, PCSK1N, SOCS1, RINL, RAB26, BMF, SLC18A1, PPFIA3, CHGB, ATP6V0B, PTPRN2, MYOC, ABCC8, GAD1, SERPINF2, CADPS, GGH, SYP, MYO7A, AP3B2, SYT7, MTSS1, TMEM198, VGF, FGFR4, STX1A | 366 | 1183 | 18224 | 1.473146 | 0.999995 | 0.315295 | 0.308428 |
| GOTERM_CC_ALL | GO:0030133~transport vesicle | 14 | 3.271028 | 0.022793 | PCSK1, EXOC3L1, SNAP25, PTPRN2, SERPINA1, ABCC8, SYP, SYT7, SCAMP5, VGF, RAB26, FGFR4, SLC18A1, STX1A | 366 | 346 | 18224 | 2.014719 | 0.999997 | 0.322031 | 0.315018 |
| GOTERM_CC_ALL | GO:0070382~exocytic vesicle | 8 | 1.869159 | 0.023572 | SCAMP5, SNAP25, PTPRN2, ABCC8, SYP, SLC18A1, STX1A, SYT7 | 366 | 141 | 18224 | 2.825098 | 0.999998 | 0.324711 | 0.317639 |
| GOTERM_CC_ALL | GO:0044463~cell projection part | 29 | 6.775701 | 0.027261 | SNAP25, SCARB1, DAGLA, ATP1A3, STMN3, GDPD5, CPLX2, CPLX1, AURKA, KIF1A, SLC18A1, PTPRN2, UNC13A, MYOC, KCNJ11, TESC, CYBRD1, ABCA7, CCK, KCNAB2, SYP, MYO7A, ADRA2C, AP3B2, SYT7, MAPK8IP1, NFASC, NEURL1, DPEP1 | 366 | 952 | 18224 | 1.516784 | 1 | 0.366364 | 0.358385 |
| GOTERM_CC_ALL | GO:0030141~secretory granule | 14 | 3.271028 | 0.029019 | PCSK1, EXOC3L1, PTPRN2, SERPINA1, SERPINF2, SERPINE1, NUDT1, SYT7, PCSK1N, RAB26, BMF, STX1A, PPFIA3, CHGB | 366 | 358 | 18224 | 1.947187 | 1 | 0.380696 | 0.372405 |
| GOTERM_CC_ALL | GO:0000228~nuclear chromosome | 19 | 4.439252 | 0.031347 | GINS2, CEBPB, NPM2, UHRF1, KMT5C, PRKDC, CDCA5, H2AFX, TONSL, DBF4B, CENPA, IPO4, AURKA, INCENP, SOX18, POLD1, E2F1, TIMELESS, MCM2 | 366 | 555 | 18224 | 1.704603 | 1 | 0.401682 | 0.392934 |
| GOTERM_CC_ALL | GO:0008076~voltage-gated potassium channel complex | 6 | 1.401869 | 0.0346 | KCNH2, SNAP25, KCNJ11, ABCC8, KCNAB2, STX1A | 366 | 90 | 18224 | 3.31949 | 1 | 0.40188 | 0.393127 |
| GOTERM_CC_ALL | GO:0005737~cytoplasm | 234 | 54.6729 | 0.035663 | CLPB, CPNE7, PLEKHB1, SERPINE1, TONSL, NUDT1, STMN3, EMC10, TPGS1, AQP3, SCAMP5, LIPF, CDC20, LIPE, RINL, RPS19, MNX1, EPHB3, CHGB, IER3, SDS, UNC13A, NUP210, SERPINF2, MYO7A, CPT1C, MAPK8IP2, MFSD12, MAPK8IP1, SULT2B1, HOXB9, HMBS, ORAI1, SIK1, HOXB2, FAM64A, TRIB3, STX1A, BEX2, TPH1, BEX1, SHMT2, PRKDC, CDCA5, WDR62, NCAPH, TRPM2, PCBP3, DHX34, DHX37, HOXC6, CDT1, PLK3, PTPRN2, STARD10, ABCA7, NBEAL2, PGA3, NR0B2, PGA5, TMEM198, PGA4, PARP10, NDUFAF8, PAH, PCSK1, SERPINA1, GPAA1, MRPS12, HEPACAM2, BICDL1, MYL6B, SHB, AACS, HEY1, KIF1A, SLC18A1, MAP4K2, ATP6V0B, RPS6KL1, MYOC, TESC, MANEAL, SYP, AP3B2, MTSS1, PDRG1, MMP11, CTU1, KIFC2, INCENP, KIF2C, PAFAH1B3, TMEM160, TIMM10, GDPD5, CENPA, CRIP1, ASCL2, RELB, RECQL4, CST3, MTHFD1L, UCP2, DTD1, NPM2, CDKN2A, GDF15, GAD1, COL22A1, AGT, MAD2L2, HEYL, FABP4, TACC3, CDK5R2, FOXA2, ANKRD13B, EXOC3L1, SCARB1, RTKN, CRTC1, GALNT18, MSI1, OGDHL, COX6A1, IPO4, PTTG1, SALL4, PHLDA2, TXNL4A, PPFIA3, KCNH2, DGAT2, LIG1, SSUH2, ATP11A, RUNDC3A, FOXP3, SRCIN1, TRAIP, CDC25B, LOC102724428, CCNE1, ANAPC5, TSNARE1, ABCG1, GNAZ, REG3A, SHC2, RNASEH2A, TROAP, FOXO6, SLC38A11, CACNA1D, SLC1A5, CPLX2, SEZ6L2, PKMYT1, CPLX1, TIMM50, NME1-NME2, VTN, PCSK1N, SOCS1, RHPN1, BMF, CYC1, SAC3D1, UBE2C, ASPSCR1, GGH, SMAD9, SYT7, AMACR, G6PC3, KIF18B, UBE2S, CDK4, MAP3K10, FGFR4, RNF187, SNAP25, DYRK2, NXT1, PYCRL, CELF4, RND1, DUSP10, SPR, DACT2, CA8, GRASP, EMX1, ABCC8, APOA2, KCNAB2, ADRA2C, VGF, CHI3L1, TNFRSF25, PFDN2, TMEM9, MCM2, CEBPB, NAXE, RGS16, LRP5, ATP1A3, RRBP1, DBF4B, CLN6, SRM, AURKA, TTR, PARD6A, RAB26, POLD1, E2F1, SLC25A22, SEC11C, CTHRC1, HN1L, CMTM8, PPP1R14B, PPP1R14C, KCNJ11, CADPS, ARID3A, SLC25A39, PRC1, RAB15, APOC1, NEURL1 | 366 | 10775 | 18224 | 1.081337 | 1 | 0.40188 | 0.393127 |
| GOTERM_CC_ALL | GO:0034705~potassium channel complex | 6 | 1.401869 | 0.036034 | KCNH2, SNAP25, KCNJ11, ABCC8, KCNAB2, STX1A | 366 | 91 | 18224 | 3.283012 | 1 | 0.40188 | 0.393127 |
| GOTERM_CC_ALL | GO:0042995~cell projection | 49 | 11.4486 | 0.03681 | SCARB1, SNAP25, DAGLA, CRTC1, STMN3, TPGS1, RINL, FFAR2, KIF1A, SLC18A1, EPHB3, UNC13A, MYOC, TESC, MYO7A, KCNAB2, SYP, ADRA2C, CPT1C, AP3B2, SRCIN1, MTSS1, MAPK8IP1, DPEP1, STX1A, GNAZ, TPH1, NAXE, ATP1A3, GDPD5, CPLX2, CPLX1, AURKA, NME1-NME2, CST3, TRPM2, ADGRG1, PARD6A, PLK3, PTPRN2, KCNJ11, STARD10, CYBRD1, ABCA7, CCK, SYT7, NFASC, RAB15, NEURL1 | 366 | 1846 | 18224 | 1.321682 | 1 | 0.40188 | 0.393127 |
| GOTERM_CC_ALL | GO:0005694~chromosome | 28 | 6.542056 | 0.03693 | CEBPB, UHRF1, PRKDC, CDCA5, TONSL, DBF4B, CENPA, IPO4, NCAPH, AURKA, RECQL4, SOX18, SALL4, POLD1, E2F1, H1FX, MYBL2, GINS2, NPM2, CDKN2A, KMT5C, CBX2, H2AFX, INCENP, CDK4, TIMELESS, KIF2C, MCM2 | 366 | 937 | 18224 | 1.487925 | 1 | 0.40188 | 0.393127 |
| GOTERM_CC_ALL | GO:0000307~cyclin-dependent protein kinase holoenzyme complex | 4 | 0.934579 | 0.037487 | PUF60, CCNE1, CDK4, HOXC10 | 366 | 37 | 18224 | 5.382957 | 1 | 0.40188 | 0.393127 |
| GOTERM_CC_ALL | GO:0031410~cytoplasmic vesicle | 36 | 8.411215 | 0.039101 | PCSK1, EXOC3L1, SNAP25, SCARB1, SERPINA1, SERPINE1, NUDT1, SLC1A5, SCAMP5, TRPM2, PCSK1N, SOCS1, RINL, RAB26, BMF, SLC18A1, PPFIA3, CHGB, ATP6V0B, PTPRN2, MYOC, ABCC8, GAD1, SERPINF2, CADPS, GGH, SYP, MYO7A, AP3B2, SYT7, MTSS1, TMEM198, VGF, ORAI1, FGFR4, STX1A | 366 | 1284 | 18224 | 1.396047 | 1 | 0.40188 | 0.393127 |
| GOTERM_CC_ALL | GO:0005657~replication fork | 5 | 1.168224 | 0.03932 | GINS2, UHRF1, POLD1, H2AFX, TONSL | 366 | 64 | 18224 | 3.890027 | 1 | 0.40188 | 0.393127 |
| GOTERM_CC_ALL | GO:0070554~synaptobrevin 2-SNAP-25-syntaxin-3-complexin complex | 2 | 0.46729 | 0.039657 | CPLX2, CPLX1 | 366 | 2 | 18224 | 49.79235 | 1 | 0.40188 | 0.393127 |
| GOTERM_CC_ALL | GO:0097708~intracellular vesicle | 36 | 8.411215 | 0.040088 | PCSK1, EXOC3L1, SNAP25, SCARB1, SERPINA1, SERPINE1, NUDT1, SLC1A5, SCAMP5, TRPM2, PCSK1N, SOCS1, RINL, RAB26, BMF, SLC18A1, PPFIA3, CHGB, ATP6V0B, PTPRN2, MYOC, ABCC8, GAD1, SERPINF2, CADPS, GGH, SYP, MYO7A, AP3B2, SYT7, MTSS1, TMEM198, VGF, ORAI1, FGFR4, STX1A | 366 | 1286 | 18224 | 1.393876 | 1 | 0.40188 | 0.393127 |
| GOTERM_CC_ALL | GO:0030658~transport vesicle membrane | 8 | 1.869159 | 0.0401 | SCAMP5, PTPRN2, ABCC8, RAB26, SYP, SLC18A1, STX1A, SYT7 | 366 | 158 | 18224 | 2.521132 | 1 | 0.40188 | 0.393127 |
| GOTERM_CC_ALL | GO:0005771~multivesicular body | 4 | 0.934579 | 0.040115 | CST3, PGA3, PGA5, PGA4 | 366 | 38 | 18224 | 5.2413 | 1 | 0.40188 | 0.393127 |
| GOTERM_CC_ALL | GO:0043025~neuronal cell body | 15 | 3.504673 | 0.043861 | PLK3, KCNJ11, CRTC1, CCK, SEZ6L2, GDPD5, ASCL1, MAPK8IP2, CPLX2, SRCIN1, CPLX1, AURKA, CST3, TRPM2, NEURL1 | 366 | 419 | 18224 | 1.782542 | 1 | 0.431557 | 0.422159 |
| GOTERM_CC_ALL | GO:0036477~somatodendritic compartment | 21 | 4.906542 | 0.045271 | PLK3, GNAZ, KCNJ11, CRTC1, ATP1A3, CCK, SEZ6L2, GDPD5, TPGS1, ASCL1, CPT1C, MAPK8IP2, CPLX2, SRCIN1, CPLX1, AURKA, MAPK8IP1, CST3, TRPM2, NEURL1, EPHB3 | 366 | 662 | 18224 | 1.579516 | 1 | 0.437616 | 0.428085 |
| GOTERM_CC_ALL | GO:0000790~nuclear chromatin | 12 | 2.803738 | 0.054292 | CEBPB, NPM2, KMT5C, UHRF1, SOX18, CDCA5, H2AFX, TIMELESS, E2F1, DBF4B, CENPA, IPO4 | 366 | 316 | 18224 | 1.890849 | 1 | 0.510554 | 0.499435 |
| GOTERM_CC_ALL | GO:0000780~condensed nuclear chromosome, centromeric region | 3 | 0.700935 | 0.054669 | KMT5C, CENPA, AURKA | 366 | 19 | 18224 | 7.86195 | 1 | 0.510554 | 0.499435 |
| GOTERM_CC_ALL | GO:0043227~membrane-bounded organelle | 260 | 60.74766 | 0.056956 | SPON2, ZNF296, CLPB, CPNE7, HDAC11, SERPINE1, PPAN, TONSL, NUDT1, SCX, STMN3, EMC10, AQP3, RRP9, SCAMP5, LIPF, CDC20, RINL, RPS19, SOX18, ZXDC, ARL6IP4, MNX1, CHGB, IER3, SDS, NUP210, SERPINF2, SOX12, MYO7A, BHLHA15, CPT1C, MFSD12, MAPK8IP1, SULT2B1, EID2, HOXB9, CLDN3, RFX6, CCDC86, ORAI1, SIK1, HOXB2, FAM64A, TRIB3, STX1A, BEX2, BEX1, TTYH3, SHMT2, PRKDC, CDCA5, C2CD4A, C2CD4B, WDR62, NCAPH, C2, TRPM2, PCBP3, DHX34, MIER2, DHX37, HOXC9, HOXC6, RTEL1-TNFRSF6B, CDT1, PLK3, PTPRN2, F8A2, ABCA7, NBEAL2, PGA3, NR0B2, PGA5, ST18, TMEM198, PGA4, PARP10, NDUFAF8, PAH, FXYD2, PCSK1, MESP1, SERPINA1, GPAA1, MRPS12, SERPINA10, HEPACAM2, MYL6B, HOXC10, WFDC2, HEY1, SLC18A1, TEAD4, MAP4K2, ATP6V0B, RPS6KL1, MYOC, TESC, MANEAL, SYP, AP3B2, MTSS1, MMP11, CTU1, INCENP, VWA1, KIF2C, PAFAH1B3, TMEM160, TIMM10, GDPD5, ASCL1, CENPA, ASCL2, RELB, RECQL4, CST3, MTHFD1L, UCP2, RAD54L, PRAME, DTD1, GINS2, NPM2, ZNF580, CDKN2A, GDF15, GAD1, COL22A1, TMEM176B, NPM3, AGT, GUCA2A, MAD2L2, HEYL, FABP4, FOXA2, ANKRD13B, EXOC3L1, SCARB1, CRTC1, GALNT18, REG1B, REG1A, MSI1, OGDHL, COX6A1, IPO4, PTTG1, SALL4, TXNL4A, PPFIA3, DGAT2, LIG1, KMT5C, ATP11A, PGC, FOXP3, TRAIP, CDC25B, LOC102724428, CCNE1, TIMELESS, DPEP1, ANAPC5, RPP25, ABCG1, GNAZ, RNASEH2A, DPT, FOXO6, SLC38A11, SLC1A5, CPLX2, SEZ6L2, PKMYT1, TIMM50, NME1-NME2, VTN, ADGRG1, PCSK1N, SOCS1, H1FX, BMF, CYC1, UBE2C, CBX2, ASPSCR1, CYBRD1, GGH, SMAD9, SYT7, DRAP1, SDK1, AMACR, G6PC3, KIF18B, UBE2S, CDK4, MAFG, SP5, MELTF, FGFR4, RNF187, SNAP25, DYRK2, PRAC2, NXT1, ZC3H3, CELF4, DUSP10, SPR, DACT2, MYBL2, DGCR6, TGIF1, EMX1, ABCC8, H2AFX, APOA2, ADRA2C, ETV4, VGF, CHI3L1, PFDN2, TMEM9, MCM2, CEBPB, NAXE, UHRF1, LRP5, ATP1A3, RRBP1, DBF4B, LAGE3, CLN6, AURKA, TTR, PARD6A, PUF60, GNG4, RAB26, POLD1, E2F1, POLR2I, SLC25A22, SEC11C, HES4, HN1L, CMTM8, KCNJ11, CADPS, ARID3A, NFASC, SLC25A39, PRC1, RAB15, APOC1, INSM1 | 366 | 12209 | 18224 | 1.060366 | 1 | 0.523048 | 0.511657 |
| GOTERM_CC_ALL | GO:0070033~synaptobrevin 2-SNAP-25-syntaxin-1a-complexin II complex | 2 | 0.46729 | 0.058893 | CPLX2, STX1A | 366 | 3 | 18224 | 33.1949 | 1 | 0.52967 | 0.518135 |
| GOTERM_CC_ALL | GO:0005902~microvillus | 5 | 1.168224 | 0.061139 | SCARB1, NFASC, STARD10, DPEP1, MYO7A | 366 | 74 | 18224 | 3.364348 | 1 | 0.52967 | 0.518135 |
| GOTERM_CC_ALL | GO:0000793~condensed chromosome | 9 | 2.102804 | 0.061332 | CEBPB, KMT5C, INCENP, CDCA5, H2AFX, KIF2C, CENPA, NCAPH, AURKA | 366 | 210 | 18224 | 2.133958 | 1 | 0.52967 | 0.518135 |
| GOTERM_CC_ALL | GO:0032991~macromolecular complex | 112 | 26.16822 | 0.061523 | EXOC3L1, HDAC11, PPAN, TONSL, SCX, MSI1, OGDHL, EMC10, COX6A1, IPO4, RRP9, SCAMP5, CDC20, RPS19, SOX18, SALL4, TXNL4A, CHRNB2, KCNH2, KMT5C, SERPINF2, SOX12, MYO7A, CPT1C, MAPK8IP2, FOXP3, CCNE1, TIMELESS, ORAI1, CCDC86, ANAPC5, TSNARE1, STX1A, GNAZ, RNASEH2A, BEX1, TTYH3, SHMT2, PRKDC, CDCA5, CACNA1D, CPLX2, CPLX1, NCAPH, TIMM50, VTN, PCBP3, H1FX, BMF, CYC1, PTPRN2, UBE2C, CBX2, SMAD9, ABCA7, NR0B2, KIF18B, UBE2S, CDK4, FXYD2, SNAP25, DYRK2, GPAA1, ZC3H3, MRPS12, MYL6B, HOXC10, MYBL2, KIF1A, ATP6V0B, RPS6KL1, ABCC8, MMP1, H2AFX, APOA2, KCNAB2, AP3B2, PDRG1, KIFC2, INCENP, PFDN2, KIF2C, MCM2, CEBPB, UHRF1, LRP5, ATP1A3, RRBP1, DBF4B, TIMM10, ASCL1, CENPA, ASCL2, RELB, AURKA, TTR, PARD6A, PUF60, GNG4, POLD1, E2F1, POLR2I, CTHRC1, GINS2, NPM2, KCNJ11, CDKN2A, COL22A1, MAD2L2, APOC1, INSM1, CDK5R2 | 366 | 4868 | 18224 | 1.145592 | 1 | 0.52967 | 0.518135 |
| GOTERM_CC_ALL | GO:0043231~intracellular membrane-bounded organelle | 236 | 55.14019 | 0.071668 | ZNF296, CLPB, CPNE7, HDAC11, SERPINE1, PPAN, TONSL, NUDT1, SCX, STMN3, EMC10, AQP3, RRP9, SCAMP5, LIPF, CDC20, RINL, RPS19, SOX18, ZXDC, ARL6IP4, MNX1, CHGB, IER3, SDS, NUP210, SERPINF2, SOX12, MYO7A, BHLHA15, CPT1C, MFSD12, MAPK8IP1, SULT2B1, EID2, HOXB9, RFX6, CCDC86, ORAI1, SIK1, HOXB2, FAM64A, TRIB3, STX1A, BEX2, BEX1, SHMT2, PRKDC, CDCA5, C2CD4A, C2CD4B, WDR62, NCAPH, TRPM2, PCBP3, DHX34, MIER2, DHX37, HOXC9, HOXC6, RTEL1-TNFRSF6B, CDT1, PLK3, PTPRN2, F8A2, ABCA7, NBEAL2, PGA3, NR0B2, PGA5, ST18, TMEM198, PGA4, PARP10, NDUFAF8, FXYD2, PCSK1, MESP1, SERPINA1, GPAA1, MRPS12, HEPACAM2, HOXC10, HEY1, SLC18A1, TEAD4, MAP4K2, ATP6V0B, RPS6KL1, MYOC, TESC, MANEAL, SYP, AP3B2, MTSS1, MMP11, CTU1, INCENP, KIF2C, TMEM160, TIMM10, GDPD5, ASCL1, CENPA, ASCL2, RELB, RECQL4, CST3, MTHFD1L, UCP2, RAD54L, PRAME, DTD1, GINS2, NPM2, ZNF580, CDKN2A, GDF15, GAD1, COL22A1, TMEM176B, NPM3, MAD2L2, HEYL, FABP4, FOXA2, ANKRD13B, EXOC3L1, SCARB1, CRTC1, GALNT18, MSI1, OGDHL, COX6A1, IPO4, PTTG1, SALL4, TXNL4A, PPFIA3, DGAT2, LIG1, KMT5C, ATP11A, FOXP3, TRAIP, CDC25B, LOC102724428, CCNE1, TIMELESS, ANAPC5, RPP25, ABCG1, GNAZ, RNASEH2A, FOXO6, SLC38A11, SLC1A5, CPLX2, SEZ6L2, PKMYT1, TIMM50, NME1-NME2, VTN, PCSK1N, SOCS1, H1FX, BMF, CYC1, UBE2C, CBX2, ASPSCR1, GGH, SMAD9, SYT7, DRAP1, AMACR, G6PC3, KIF18B, UBE2S, CDK4, MAFG, SP5, FGFR4, RNF187, SNAP25, DYRK2, PRAC2, NXT1, ZC3H3, CELF4, DUSP10, SPR, DACT2, MYBL2, DGCR6, TGIF1, EMX1, ABCC8, H2AFX, APOA2, ADRA2C, ETV4, VGF, CHI3L1, PFDN2, TMEM9, MCM2, CEBPB, NAXE, UHRF1, LRP5, ATP1A3, RRBP1, DBF4B, LAGE3, CLN6, AURKA, PARD6A, PUF60, RAB26, POLD1, E2F1, POLR2I, SLC25A22, SEC11C, HES4, HN1L, CMTM8, KCNJ11, CADPS, ARID3A, SLC25A39, PRC1, RAB15, APOC1, INSM1 | 366 | 11035 | 18224 | 1.064884 | 1 | 0.607523 | 0.594292 |
| GOTERM_CC_ALL | GO:0000779~condensed chromosome, centromeric region | 6 | 1.401869 | 0.074649 | CEBPB, KMT5C, INCENP, KIF2C, CENPA, AURKA | 366 | 112 | 18224 | 2.667447 | 1 | 0.623205 | 0.609633 |
| GOTERM_CC_ALL | GO:1904949~ATPase complex | 3 | 0.700935 | 0.08271 | FXYD2, ATP1A3, ABCA7 | 366 | 24 | 18224 | 6.224044 | 1 | 0.670194 | 0.655599 |
| GOTERM_CC_ALL | GO:0098533~ATPase dependent transmembrane transport complex | 3 | 0.700935 | 0.08271 | FXYD2, ATP1A3, ABCA7 | 366 | 24 | 18224 | 6.224044 | 1 | 0.670194 | 0.655599 |
| GOTERM_CC_ALL | GO:0005819~spindle | 11 | 2.570093 | 0.086302 | MAD2L2, CDC20, KIF18B, INCENP, PRC1, HEPACAM2, TACC3, WDR62, SAC3D1, CDC25B, AURKA | 366 | 303 | 18224 | 1.807643 | 1 | 0.689166 | 0.674157 |
| GOTERM_CC_ALL | GO:0098796~membrane protein complex | 29 | 6.775701 | 0.092983 | SNAP25, GNAZ, TTYH3, GPAA1, LRP5, ATP1A3, CACNA1D, EMC10, TIMM10, COX6A1, CPLX2, CPLX1, TIMM50, SCAMP5, VTN, GNG4, CYC1, KCNH2, CHRNB2, ATP6V0B, KCNJ11, ABCC8, ABCA7, KCNAB2, CPT1C, AP3B2, FXYD2, TSNARE1, STX1A | 366 | 1074 | 18224 | 1.344486 | 1 | 0.731906 | 0.715966 |
| GOTERM_CC_ALL | GO:0031904~endosome lumen | 3 | 0.700935 | 0.094921 | PGA3, PGA5, PGA4 | 366 | 26 | 18224 | 5.745271 | 1 | 0.736644 | 0.720601 |

| **Table S5: Gene Ontology (GO) Functional Enrichment Results after Redundancy Reduction using REVIGO** | | | | | |
| --- | --- | --- | --- | --- | --- |
|  |  |  |  |  |  |
| Category | TermID | Name | Genes | Count | Note |
| Biological Process | GO:0007275 | "multicellular organism development" | SPON2, CRTC1, HDAC11, PLEKHB1, SERPINE1, NUDT1, SCX, STMN3, MSI1, TPGS1, AQP3, CDC20, RPS19, SOX18, SALL4, PHLDA2, TNFRSF4, MNX1, EPHB3, SLC39A3, CHRNB2, TPRA1, DGAT2, UNC13A, LIG1, SERPINF2, SOX12, MYO7A, MAPK8IP2, SRCIN1, FOXP3, PGF, LOC102724428, EID2, HOXB9, RFX6, TIMELESS, ORAI1, SIK1, HOXB2, REG3A, TPH1, BEX1, PRKDC, FOXO6, WDR62, CPLX2, SEZ6L2, NME1-NME2, VTN, TRPM2, ADGRG1, ZNHIT2, HOXC9, HOXC6, CBX2, SMAD9, NBEAL2, NR0B2, TMEM198, SDK1, ARTN, CDK4, MAFG, SP5, MESP1, SNAP25, DAGLA, CELF4, BICDL1, MYL6B, SHB, HOXC10, AACS, RND1, DUSP10, HEY1, ADAMTSL2, DACT2, DGCR6, TEAD4, TGIF1, EMX1, MYOC, TESC, H2AFX, APOA2, KCNAB2, ADRA2C, ETV4, MTSS1, MMP11, VGF, CHI3L1, TNFRSF25, PAFAH1B3, CEBPB, LRP5, GDPD5, ASCL1, CRIP1, ASCL2, RELB, AURKA, DLL3, DLL4, RECQL4, CST3, PARD6A, MTHFD1L, RAB26, UCP2, E2F1, RAD54L, CTHRC1, HES4, CMTM8, LRRC24, NPM2, CDKN2A, IL34, TMEM176B, TMEM176A, STAC3, CCK, AGT, MAD2L2, HEYL, NFASC, SMOC1, MFAP2, NEURL1, TACC3, INSM1, KCNK3, CDK5R2, FOXA2 | 137 |  |
| Biological Process | GO:0008150 | "biological_process" | SPON2, ZNF296, CPNE7, HDAC11, PLEKHB1, SERPINE1, TONSL, NUDT1, SCX, STMN3, TPGS1, AQP3, SCAMP5, LIPF, CDC20, LIPE, RPS19, SOX18, MNX1, EPHB3, IER3, SDS, UNC13A, NUP210, SERPINF2, SOX12, MYO7A, BHLHA15, CPT1C, MAPK8IP2, MAPK8IP1, SULT2B1, EID2, HOXB9, CLDN3, RFX6, ASPHD1, HMBS, ORAI1, SIK1, HOXB2, FAM64A, TRIB3, PLPP2, STX1A, BEX2, TPH1, BEX1, TTYH3, TMEM63C, SHMT2, PRKDC, CDCA5, C2CD4A, C2CD4B, C2CD4C, WDR62, NCAPH, C2, TRPM2, ZNHIT2, ADAMTS14, HOXC9, HOXC6, CDT1, PLK3, PTPRN2, STARD10, ABCA7, NBEAL2, PGA3, NR0B2, PGA5, TMEM198, PGA4, PARP10, ARTN, PAH, FXYD2, PCSK1, MESP1, SERPINA1, GPAA1, MRPS12, SERPINA10, HEPACAM2, BICDL1, MYL6B, SHB, HOXC10, WFDC2, ASGR1, AACS, HEY1, ADAMTSL2, FFAR2, KIF1A, SLC18A1, TEAD4, MAP4K2, ATP6V0B, RPS6KL1, MYOC, GP1BB, TESC, SYP, AP3B2, MTSS1, MMP11, KIFC2, INCENP, SLC29A4, VWA1, KIF2C, PAFAH1B3, TIMM10, GDPD5, ASCL1, CENPA, CRIP1, SLC7A1, ASCL2, RELB, RECQL4, CST3, NPW, MTHFD1L, UCP2, RAD54L, PRAME, DTD1, GINS2, LRRC24, NPM2, ZNF580, CDKN2A, GDF15, IL34, GAD1, TMEM176B, TMEM176A, STAC3, AGT, GUCA2A, MAD2L2, HEYL, FABP4, SLCO3A1, SMOC1, SHKBP1, TACC3, CDK5R2, FOXA2, EXOC3L1, SCARB1, RTKN, CRTC1, GALNT18, REG1B, REG1A, MSI1, OGDHL, COX6A1, IPO4, TMEM145, PTTG1, SALL4, PHLDA2, TXNL4A, TNFRSF4, PPFIA3, SLC39A3, CHRNB2, KCNH2, TPRA1, DGAT2, KCNH6, LIG1, ATP11A, PGC, RUNDC3A, FOXP3, SRCIN1, PGF, TRAIP, CDC25B, LOC102724428, CCNE1, TIMELESS, DPEP1, ANAPC5, TSNARE1, ABCG1, LY6E, GNAZ, REG3A, SHC2, DPT, FOXO6, SLC38A11, CACNA1D, SLC1A5, CPLX2, SEZ6L2, PKMYT1, CPLX1, TIMM50, NME1-NME2, VTN, ADGRG1, PCSK1N, SOCS1, ADGRG5, RHPN1, BMF, CYC1, SAC3D1, UBE2C, SLC52A2, CBX2, ASPSCR1, CYBRD1, GGH, SMAD9, SYT7, SDK1, AMACR, G6PC3, KIF18B, UBE2S, CDK4, MAFG, MAP3K10, SP5, MELTF, FGFR4, ULBP2, RNF187, SNAP25, DAGLA, DYRK2, PYCRL, PODXL2, ZC3H3, CELF4, RND1, DUSP10, SPR, DACT2, MYBL2, DGCR6, CA8, GRASP, TGIF1, PLEKHG4, EMX1, ABCC8, MMP1, H2AFX, APOA2, C10ORF11, KCNAB2, ADRA2C, ETV4, VGF, CHI3L1, TNFRSF25, MCM2, CEBPB, NAXE, UHRF1, RGS16, LRP5, ATP1A3, RRBP1, DBF4B, CLN6, AURKA, DLL3, DLL4, P2RY6, TTR, PARD6A, PUF60, GNG4, RAB26, E2F1, POLR2I, SLC25A22, CTHRC1, HES4, OR51E1, CMTM8, KCNJ11, CADPS, ARID3A, CCK, FAM155B, NFASC, SLC25A39, PRC1, RAB15, TSPAN18, MFAP2, APOC1, NEURL1, INSM1, KCNK3 | 308 | Alternative of "GO:0044699" |
| Biological Process | GO:0023061 | "signal release" | EXOC3L1, SNAP25, LRP5, C2CD4A, CACNA1D, C2CD4B, C2CD4C, CPLX2, CPLX1, AACS, TRPM2, UCP2, FFAR2, PPFIA3, PTPRN2, UNC13A, KCNJ11, ABCC8, GAD1, CADPS, ADRA2C, NR0B2, SYT7, VGF, RFX6, STX1A, FOXA2 | 27 |  |
| Biological Process | GO:0032501 | "multicellular organismal process" | SPON2, SCARB1, CRTC1, HDAC11, PLEKHB1, SERPINE1, NUDT1, SCX, STMN3, MSI1, TPGS1, AQP3, SCAMP5, CDC20, RPS19, SOX18, SALL4, PHLDA2, TNFRSF4, MNX1, EPHB3, SLC39A3, CHRNB2, KCNH2, TPRA1, DGAT2, UNC13A, LIG1, SERPINF2, SOX12, MYO7A, MAPK8IP2, SRCIN1, PGC, FOXP3, PGF, TRAIP, LOC102724428, EID2, HOXB9, RFX6, TIMELESS, ORAI1, SIK1, HOXB2, ABCG1, REG3A, TPH1, BEX1, PRKDC, FOXO6, CACNA1D, WDR62, CPLX2, SEZ6L2, NME1-NME2, VTN, TRPM2, ADGRG1, PCSK1N, ADAMTS14, ZNHIT2, SOCS1, HOXC9, HOXC6, CBX2, SMAD9, ABCA7, NBEAL2, PGA3, NR0B2, PGA5, SYT7, PGA4, TMEM198, SDK1, ARTN, CDK4, MAFG, SP5, MESP1, SNAP25, SERPINA1, DAGLA, SERPINA10, CELF4, BICDL1, MYL6B, SHB, HOXC10, AACS, RND1, DUSP10, HEY1, ADAMTSL2, DACT2, FFAR2, DGCR6, TEAD4, TGIF1, EMX1, MYOC, MMP1, GP1BB, TESC, H2AFX, APOA2, KCNAB2, ADRA2C, ETV4, MTSS1, MMP11, VGF, VWA1, CHI3L1, TNFRSF25, PAFAH1B3, CEBPB, LRP5, ATP1A3, RRBP1, GDPD5, ASCL1, CRIP1, ASCL2, RELB, AURKA, DLL3, DLL4, RECQL4, CST3, PARD6A, MTHFD1L, RAB26, UCP2, E2F1, RAD54L, POLR2I, CTHRC1, HES4, CMTM8, LRRC24, NPM2, ZNF580, CDKN2A, IL34, TMEM176B, TMEM176A, STAC3, CCK, AGT, MAD2L2, HEYL, NFASC, FABP4, SMOC1, APOC1, MFAP2, NEURL1, TACC3, INSM1, KCNK3, CDK5R2, FOXA2 | 164 | Alternative of "GO:0044707" |
| Biological Process | GO:0032502 | "developmental process" | SPON2, CRTC1, HDAC11, PLEKHB1, SERPINE1, NUDT1, SCX, STMN3, MSI1, TPGS1, AQP3, CDC20, RPS19, SOX18, SALL4, PHLDA2, TNFRSF4, MNX1, EPHB3, SLC39A3, CHRNB2, TPRA1, DGAT2, UNC13A, LIG1, SERPINF2, SOX12, MYO7A, BHLHA15, MAPK8IP2, SRCIN1, FOXP3, PGF, CDC25B, LOC102724428, EID2, CLDN3, HOXB9, RFX6, TIMELESS, ORAI1, SIK1, HOXB2, REG3A, TPH1, BEX1, PRKDC, FOXO6, WDR62, CPLX2, SEZ6L2, NME1-NME2, VTN, TRPM2, ADGRG1, ZNHIT2, HOXC9, HOXC6, CBX2, SMAD9, NBEAL2, NR0B2, TMEM198, SDK1, ARTN, CDK4, MAFG, SP5, MELTF, MESP1, SNAP25, DAGLA, CELF4, BICDL1, MYL6B, SHB, HOXC10, AACS, RND1, DUSP10, HEY1, ADAMTSL2, DACT2, DGCR6, TEAD4, TGIF1, EMX1, MYOC, TESC, H2AFX, APOA2, KCNAB2, SYP, ADRA2C, ETV4, MTSS1, MMP11, VGF, CHI3L1, TNFRSF25, PAFAH1B3, CEBPB, LRP5, GDPD5, ASCL1, CRIP1, ASCL2, RELB, AURKA, DLL3, DLL4, RECQL4, CST3, PARD6A, MTHFD1L, RAB26, UCP2, E2F1, RAD54L, POLR2I, CTHRC1, HES4, CMTM8, LRRC24, NPM2, CDKN2A, GDF15, IL34, TMEM176B, TMEM176A, STAC3, CCK, AGT, MAD2L2, HEYL, NFASC, SMOC1, MFAP2, NEURL1, TACC3, INSM1, KCNK3, CDK5R2, FOXA2 | 144 | Alternative of "GO:0044767" |
| Biological Process | GO:0044092 | "negative regulation of molecular function" | SERPINA1, RTKN, PPP1R35, SERPINA10, SERPINE1, LRP5, PKMYT1, SHB, WFDC2, AURKA, CDC20, CST3, VTN, PCSK1N, CST1, SOCS1, DUSP10, PTTG1, HEY1, E2F1, TNFRSF4, SPINK4, PPP1R14B, PTPRN2, PPP1R14C, CDKN2A, UBE2C, TESC, SERPINF2, APOA2, SRCIN1, NR0B2, FOXP3, AGT, MAPK8IP1, PARP10, MAD2L2, HEYL, LOC102724428, FABP4, APOC1, RIMBP2, MAP3K10, DPEP1, SIK1, TRIB3, ANAPC5, FOXA2 | 48 |  |
| Biological Process | GO:0051179 | "localization" | SPON2, SCARB1, EXOC3L1, SERPINE1, TONSL, COX6A1, AQP3, IPO4, SCAMP5, RPS19, TNFRSF4, PPFIA3, SLC39A3, CHRNB2, KCNH2, UNC13A, KCNH6, NUP210, SERPINF2, MYO7A, BHLHA15, ATP11A, MAPK8IP2, SRCIN1, FOXP3, LOC102724428, RFX6, ORAI1, SIK1, ABCG1, STX1A, TTYH3, TMEM63C, C2CD4A, CACNA1D, C2CD4B, SLC38A11, C2CD4C, SLC1A5, CPLX2, CPLX1, TIMM50, C2, TRPM2, SOCS1, BMF, CYC1, PTPRN2, SLC52A2, STARD10, ASPSCR1, ABCA7, NR0B2, SYT7, PARP10, G6PC3, FXYD2, MELTF, SNAP25, SERPINA1, DYRK2, GPAA1, AACS, FFAR2, KIF1A, SLC18A1, ATP6V0B, ABCC8, TESC, APOA2, KCNAB2, ADRA2C, AP3B2, VGF, CHI3L1, SLC29A4, LRP5, ATP1A3, TIMM10, CENPA, SLC7A1, P2RY6, TTR, RAB26, UCP2, E2F1, SLC25A22, CMTM8, KCNJ11, GAD1, CADPS, CCK, AGT, FAM155B, NFASC, SLC25A39, SLCO3A1, RAB15, APOC1, NEURL1, KCNK3, CDK5R2, FOXA2 | 103 | Alternative of "GO:1902578" |
| Biological Process | GO:1903047 | "mitotic cell cycle process" | PRKDC, CDCA5, LRP5, HEPACAM2, DBF4B, WDR62, PKMYT1, CENPA, NCAPH, AURKA, CDC20, PTTG1, E2F1, MYBL2, TXNL4A, SAC3D1, GINS2, CDT1, PLK3, TPRA1, NPM2, LIG1, NUP210, CDKN2A, UBE2C, ARID3A, CDC25B, MAD2L2, KIF18B, CCNE1, UBE2S, PRC1, INCENP, CDK4, TIMELESS, TACC3, FAM64A, ANAPC5, KIF2C, MCM2 | 40 |  |
| Biological Process | GO:0000280 | "nuclear division" | CDCA5, LRP5, HEPACAM2, PKMYT1, CENPA, NCAPH, AURKA, CDC20, PTTG1, RAD54L, MYBL2, TXNL4A, SAC3D1, NPM2, UBE2C, CDC25B, MAD2L2, KIF18B, UBE2S, PRC1, INCENP, TIMELESS, TACC3, FAM64A, ANAPC5, KIF2C | 26 |  |
| Biological Process | GO:0051301 | "cell division" | CDCA5, HEPACAM2, CENPA, NCAPH, AURKA, CDC20, PARD6A, PTTG1, TXNL4A, SAC3D1, PLK3, TPRA1, LIG1, CDKN2A, UBE2C, PGF, CDC25B, MAD2L2, KIF18B, CCNE1, UBE2S, PRC1, INCENP, CDK4, TIMELESS, TACC3, FAM64A, ANAPC5, KIF2C | 29 |  |
| Biological Process | GO:0099504 | "synaptic vesicle cycle" | SNAP25, UNC13A, CADPS, C2CD4A, C2CD4B, C2CD4C, CPLX2, SLC18A1, CPLX1, STX1A, SYT7 | 11 |  |
| Biological Process | GO:1903530 | "regulation of secretion by cell" | SNAP25, LRP5, C2CD4A, CACNA1D, C2CD4B, C2CD4C, CPLX2, CPLX1, AACS, SCAMP5, TRPM2, SOCS1, RAB26, UCP2, FFAR2, TNFRSF4, CHRNB2, UNC13A, KCNJ11, ABCC8, APOA2, CCK, ADRA2C, SRCIN1, NR0B2, FOXP3, SYT7, AGT, RFX6, RAB15, STX1A, CDK5R2, FOXA2 | 33 |  |
| Biological Process | GO:0010817 | "regulation of hormone levels" | PCSK1, EXOC3L1, SNAP25, SCARB1, LRP5, CACNA1D, CPLX1, AACS, TRPM2, PCSK1N, TTR, UCP2, FFAR2, PTPRN2, DGAT2, KCNJ11, ABCC8, ADRA2C, NR0B2, SYT7, AGT, VGF, RFX6, STX1A, FOXA2 | 25 |  |
| Biological Process | GO:0045595 | "regulation of cell differentiation" | SNAP25, MESP1, CRTC1, PLEKHB1, SHB, AQP3, CDC20, DUSP10, HEY1, RPS19, EPHB3, CHRNB2, EMX1, UNC13A, MYOC, TESC, SERPINF2, BHLHA15, ADRA2C, SRCIN1, FOXP3, LOC102724428, MMP11, TRIB3, SIK1, ABCG1, REG3A, CEBPB, TPH1, LRP5, FOXO6, GDPD5, ASCL1, ASCL2, AURKA, DLL3, NME1-NME2, DLL4, ADGRG1, E2F1, PRAME, CTHRC1, CDKN2A, GDF15, IL34, TMEM176B, TMEM176A, AGT, MAD2L2, HEYL, SDK1, MAFG, SMOC1, NEURL1, MELTF, INSM1, FOXA2 | 57 |  |
| Biological Process | GO:0071705 | "nitrogen compound transport" | EXOC3L1, SNAP25, NXT1, ZC3H3, LRP5, CACNA1D, SLC38A11, SLC1A5, SLC7A1, AQP3, CPLX1, AACS, TRPM2, TTR, UCP2, FFAR2, SLC25A22, SLC18A1, CHRNB2, PTPRN2, KCNJ11, NUP210, ABCC8, SLC52A2, CADPS, ABCA7, ADRA2C, NR0B2, SYT7, AGT, VGF, RFX6, SLC29A4, STX1A, FOXA2 | 35 |  |
| Biological Process | GO:0006836 | "neurotransmitter transport" | SNAP25, PTPRN2, UNC13A, GAD1, CADPS, C2CD4A, C2CD4B, C2CD4C, CPLX2, CPLX1, SYT7, SLC18A1, STX1A, PPFIA3 | 14 |  |
| Biological Process | GO:0046903 | "secretion" | EXOC3L1, SNAP25, SERPINA1, SERPINE1, LRP5, C2CD4A, CACNA1D, C2CD4B, C2CD4C, CPLX2, CPLX1, AACS, SCAMP5, TRPM2, SOCS1, RAB26, UCP2, FFAR2, TNFRSF4, PPFIA3, CHRNB2, PTPRN2, UNC13A, KCNJ11, ABCC8, GAD1, SERPINF2, CADPS, STARD10, APOA2, CCK, ADRA2C, SRCIN1, NR0B2, FOXP3, SYT7, AGT, VGF, RFX6, RAB15, NEURL1, CHI3L1, STX1A, CDK5R2, FOXA2 | 45 |  |
| Biological Process | GO:0007267 | "cell-cell signaling" | PCSK1, EXOC3L1, SNAP25, MESP1, CRTC1, CELF4, TPGS1, AACS, CDC20, FFAR2, SLC18A1, PPFIA3, CHRNB2, UNC13A, MYOC, ABCC8, BHLHA15, SYP, ADRA2C, MAPK8IP2, PGF, HOXB9, VGF, RFX6, CCNE1, STX1A, LRP5, C2CD4A, CACNA1D, C2CD4B, C2CD4C, CPLX2, CPLX1, TRPM2, ADGRG1, PARD6A, UCP2, CTHRC1, PTPRN2, KCNJ11, GDF15, GAD1, CADPS, STAC3, NR0B2, AGT, SYT7, TMEM198, MAD2L2, NEURL1, KCNK3, FOXA2 | 52 |  |
| Biological Process | GO:0097479 | "synaptic vesicle localization" | SNAP25, UNC13A, CADPS, C2CD4A, C2CD4B, C2CD4C, CPLX2, AP3B2, CPLX1, STX1A, SYT7 | 11 |  |
| Biological Process | GO:0015833 | "peptide transport" | EXOC3L1, SNAP25, PTPRN2, KCNJ11, ABCC8, LRP5, CACNA1D, ADRA2C, NR0B2, CPLX1, AACS, SYT7, TRPM2, VGF, RFX6, UCP2, FFAR2, STX1A, FOXA2 | 19 |  |
| Biological Process | GO:0009914 | "hormone transport" | EXOC3L1, SNAP25, PTPRN2, KCNJ11, ABCC8, LRP5, CACNA1D, ADRA2C, NR0B2, CPLX1, AACS, SYT7, TRPM2, TTR, VGF, RFX6, UCP2, FFAR2, STX1A, FOXA2 | 20 |  |
| Biological Process | GO:0001505 | "regulation of neurotransmitter levels" | SNAP25, PTPRN2, DAGLA, UNC13A, GAD1, CADPS, C2CD4A, C2CD4B, C2CD4C, CPLX2, CPLX1, SYT7, PAH, STX1A, PPFIA3 | 15 |  |
| Molecular Function | GO:0004857 | "enzyme inhibitor activity" | SPINK4, PPP1R14B, RTKN, PPP1R14C, SERPINA1, CDKN2A, TESC, PPP1R35, SERPINF2, SERPINA10, SERPINE1, APOA2, WFDC2, AGT, MAPK8IP1, CST3, PCSK1N, CST1, SOCS1, PTTG1, APOC1, DPEP1, TRIB3 | 23 |  |
| Molecular Function | GO:0046983 | "protein dimerization activity" | MESP1, SCARB1, CEBPB, NAXE, SCX, TIMM10, ASCL1, CENPA, ASCL2, CLN6, ASGR1, SRM, TTR, RPS19, HEY1, MTHFD1L, SOX18, E2F1, HES4, KCNH2, CHRNB2, DGAT2, SDS, NUP210, GAD1, TESC, H2AFX, SERPINF2, APOA2, ARID3A, MYO7A, BHLHA15, ADRA2C, NR0B2, FOXP3, PGF, DRAP1, HEYL, MAFG, TIMELESS, MAP3K10, PAFAH1B3, KCNK3, ABCG1, STX1A | 45 |  |
| Molecular Function | GO:0005515 | "protein binding" | SPON2, CLPB, CPNE7, HDAC11, PLEKHB1, SERPINE1, TONSL, NUDT1, SCX, STMN3, TPGS1, SCAMP5, CDC20, LIPE, RPS19, SOX18, ZXDC, ARL6IP4, CHGB, IER3, SDS, UNC13A, NUP210, SERPINF2, MYO7A, BHLHA15, CPT1C, MAPK8IP2, MAPK8IP1, SULT2B1, EID2, HOXB9, CLDN3, RFX6, CCDC86, ORAI1, SIK1, FAM64A, TRIB3, PLPP2, STX1A, BEX2, BEX1, SHMT2, PRKDC, PPP1R35, CDCA5, C2CD4A, C2CD4B, C2CD4C, WDR62, NCAPH, HOXC9, CDT1, PLK3, STARD10, NR0B2, PARP10, NDUFAF8, ARTN, MESP1, SERPINA1, GPAA1, MRPS12, HEPACAM2, BICDL1, MYL6B, SHB, ASGR1, AACS, HEY1, ADAMTSL2, FFAR2, KIF1A, TEAD4, MAP4K2, MYOC, GP1BB, TESC, SYP, MTSS1, PDRG1, CTU1, KIFC2, INCENP, VWA1, KIF2C, PAFAH1B3, GRAMD1A, TIMM10, GDPD5, ASCL1, CENPA, SLC7A1, ASCL2, RELB, RECQL4, CST3, CST1, NPW, MTHFD1L, UCP2, RAD54L, PRAME, GINS2, NPM2, ZNF580, CDKN2A, GDF15, IL34, GAD1, STAC3, NPM3, AGT, GUCA2A, MAD2L2, SH3RF3, HEYL, SMOC1, SHKBP1, TACC3, FOXA2, EXOC3L1, SCARB1, RTKN, CRTC1, REG1A, MSI1, OGDHL, KLHL35, IPO4, PTTG1, SALL4, TXNL4A, PPFIA3, CHRNB2, KCNH2, DGAT2, KMT5C, FBXW9, ATP11A, RUNDC3A, FOXP3, SRCIN1, PGF, TRAIP, CDC25B, LOC102724428, CCNE1, TIMELESS, DPEP1, ANAPC5, TSNARE1, RELL2, RPP25, ABCG1, GNAZ, REG3A, SHC2, TROAP, FOXO6, CACNA1D, SLC1A5, CPLX2, PKMYT1, CPLX1, TIMM50, VTN, ADGRG1, PCSK1N, SOCS1, RHPN1, HYI, H1FX, C16ORF59, BMF, SAC3D1, UBE2C, SLC52A2, CBX2, ASPSCR1, CYBRD1, SMAD9, SYT7, DRAP1, SDK1, AMACR, KIF18B, UBE2S, CDK4, MAFG, MAP3K10, MELTF, FGFR4, ULBP2, RNF187, SNAP25, DYRK2, NXT1, PYCRL, PODXL2, ZC3H3, RND1, DACT2, MYBL2, CA8, GRASP, TGIF1, PLEKHG4, EMX1, ABCC8, H2AFX, APOA2, ADRA2C, ETV4, VGF, PFDN2, MCM2, CEBPB, NAXE, UHRF1, RGS16, LRP5, ATP1A3, DBF4B, LAGE3, CLN6, SRM, AURKA, DLL3, DLL4, P2RY6, TTR, PARD6A, PUF60, GNG4, RAB26, POLD1, E2F1, CTHRC1, HES4, CMTM8, KCNJ11, CADPS, ARID3A, CCK, NFASC, PRC1, RAB15, INSM1, KCNK3 | 251 |  |
| Molecular Function | GO:0000149 | "SNARE binding" | SNAP25, EXOC3L1, UNC13A, ABCC8, C2CD4A, C2CD4B, TSNARE1, C2CD4C, CPLX2, CPLX1, STX1A, SYT7 | 12 |  |
| Cellular Component | GO:0030424 | "axon" | DAGLA, ATP1A3, STMN3, GDPD5, TPGS1, CPLX2, CPLX1, AURKA, CST3, KIF1A, SLC18A1, PTPRN2, UNC13A, MYOC, KCNJ11, CCK, KCNAB2, SYP, ADRA2C, CPT1C, SRCIN1, AP3B2, SYT7, MAPK8IP1, NFASC | 25 |  |
| Cellular Component | GO:0033267 | "axon part" | PTPRN2, DAGLA, UNC13A, MYOC, KCNJ11, CCK, KCNAB2, SYP, ADRA2C, CPLX2, AP3B2, CPLX1, SYT7, AURKA, MAPK8IP1, NFASC, KIF1A, SLC18A1 | 18 |  |

| **Table S6: Significant Pathways Revealing through Over-representative Analyses in GNEC using WebGestalt** | | | | | | | | | | | |
| --- | --- | --- | --- | --- | --- | --- | --- | --- | --- | --- | --- |
|  |  |  |  |  |  |  |  |  |  |  |  |
| Pathway Database | geneSet | description | link | size | overlap | expect | enrichmentRatio | pValue | overlapId | userId | FDR |
| KEGG | hsa04110 | Cell cycle | http://www.kegg.jp/kegg-bin/show_pathway?hsa04110+1019+1029+10459+1869+4171+51433+5591+898+9088+9232+991+994 | 124 | 12 | 2.573303 | 4.663267 | 8.95E-06 | 1019;1029;10459;1869;4171;51433;5591;898;9088;9232;991;994 | CDKN2A;PTTG1;MCM2;MAD2L2;ANAPC5;PRKDC;E2F1;PKMYT1;CCNE1;CDC20;CDC25B;CDK4 | 0.002946 |
| KEGG | hsa04721 | Synaptic vesicle cycle | http://www.kegg.jp/kegg-bin/show_pathway?hsa04721+10814+10815+23025+533+6570+6616+6804 | 63 | 7 | 1.307404 | 5.354122 | 3.02E-04 | 10814;10815;23025;533;6570;6616;6804 | SLC18A1;SNAP25;UNC13A;ATP6V0B;CPLX2;CPLX1;STX1A | 0.049714 |
| KEGG | hsa04911 | Insulin secretion | http://www.kegg.jp/kegg-bin/show_pathway?hsa04911+3767+478+486+6616+6804+6833+776 | 85 | 7 | 1.763958 | 3.968349 | 0.001844 | 3767;478;486;6616;6804;6833;776 | SNAP25;ATP1A3;FXYD2;STX1A;ABCC8;KCNJ11;CACNA1D | 0.122929 |
| KEGG | hsa00790 | Folate biosynthesis | http://www.kegg.jp/kegg-bin/show_pathway?hsa00790+5053+6697+7166+8836 | 26 | 4 | 0.539564 | 7.4134 | 0.001868 | 5053;6697;7166;8836 | SPR;GGH;PAH;TPH1 | 0.122929 |
| KEGG | hsa04950 | Maturity onset diabetes of the young | http://www.kegg.jp/kegg-bin/show_pathway?hsa04950+168620+222546+3110+3170 | 26 | 4 | 0.539564 | 7.4134 | 0.001868 | 168620;222546;3110;3170 | MNX1;FOXA2;RFX6;BHLHA15 | 0.122929 |
| Panther | P00045 | Notch signaling pathway | http://www.pantherdb.org/pathway/pathwayDiagram.jsp?catAccession=P00045 | 40 | 6 | 1.006178 | 5.963158 | 3.84E-04 | 9148;57801;26508;54567;10683;23462 | DLL4;DLL3;HES4;HEYL;HEY1;NEURL1 | 0.049926 |
| Panther | P00011 | Blood coagulation | http://www.pantherdb.org/pathway/pathwayDiagram.jsp?catAccession=P00011 | 38 | 5 | 0.955869 | 5.23084 | 0.002244 | 5265;5054;5345;2812;51156 | SERPINA1;SERPINF2;GP1BB;SERPINE1;SERPINA10 | 0.11389 |
| Panther | P05734 | Synaptic vesicle trafficking | http://www.pantherdb.org/pathway/pathwayDiagram.jsp?catAccession=P05734 | 24 | 4 | 0.603707 | 6.625731 | 0.002628 | 9066;6855;6616;6804 | SNAP25;STX1A;SYP;SYT7 | 0.11389 |
| Panther | P00050 | Plasminogen activating cascade | http://www.pantherdb.org/pathway/pathwayDiagram.jsp?catAccession=P00050 | 15 | 3 | 0.377317 | 7.950877 | 0.005541 | 4312;5054;5345 | MMP1;SERPINF2;SERPINE1 | 0.147921 |
| Panther | P04375 | 5HT3 type receptor mediated signaling pathway | http://www.pantherdb.org/pathway/pathwayDiagram.jsp?catAccession=P04375 | 16 | 3 | 0.402471 | 7.453947 | 0.006699 | 3777;6570;6616 | SLC18A1;SNAP25;KCNK3 | 0.147921 |
| Panther | P04376 | 5HT4 type receptor mediated signaling pathway | http://www.pantherdb.org/pathway/pathwayDiagram.jsp?catAccession=P04376 | 31 | 4 | 0.779788 | 5.129598 | 0.006827 | 3777;2786;6570;6616 | SLC18A1;SNAP25;KCNK3;GNG4 | 0.147921 |
| Reactome | R-HSA-69242 | S Phase | http://reactome.org/PathwayBrowser/#/R-HSA-69242 | 161 | 12 | 3.310309 | 3.625039 | 1.19E-04 | 1019;11065;113130;1869;3978;4171;51433;51659;5424;81620;898;994 | MCM2;ANAPC5;POLD1;GINS2;CDCA5;CDT1;UBE2C;E2F1;CCNE1;LIG1;CDC25B;CDK4 | 0.160052 |
| Reactome | R-HSA-69278 | Cell Cycle, Mitotic | http://reactome.org/PathwayBrowser/#/R-HSA-69278 | 536 | 24 | 11.02066 | 2.177729 | 2.69E-04 | 1019;1029;1058;11004;11065;113130;1869;23225;23397;3014;3619;3978;4171;4605;51433;51659;5424;6790;81620;898;9088;9232;991;994 | CDKN2A;PTTG1;CENPA;MCM2;ANAPC5;POLD1;GINS2;CDCA5;MYBL2;INCENP;AURKA;NUP210;CDT1;KIF2C;UBE2C;E2F1;NCAPH;PKMYT1;CCNE1;LIG1;H2AFX;CDC20;CDC25B;CDK4 | 0.160052 |
| Reactome | R-HSA-264642 | Acetylcholine Neurotransmitter Release Cycle | http://reactome.org/PathwayBrowser/#/R-HSA-264642 | 17 | 4 | 0.349536 | 11.44375 | 3.35E-04 | 10815;6616;6804;8541 | PPFIA3;SNAP25;CPLX1;STX1A | 0.160052 |
| Reactome | R-HSA-181429 | Serotonin Neurotransmitter Release Cycle | http://reactome.org/PathwayBrowser/#/R-HSA-181429 | 18 | 4 | 0.370097 | 10.80799 | 4.24E-04 | 10815;6616;6804;8541 | PPFIA3;SNAP25;CPLX1;STX1A | 0.160052 |
| Reactome | R-HSA-181430 | Norepinephrine Neurotransmitter Release Cycle | http://reactome.org/PathwayBrowser/#/R-HSA-181430 | 18 | 4 | 0.370097 | 10.80799 | 4.24E-04 | 10815;6616;6804;8541 | PPFIA3;SNAP25;CPLX1;STX1A | 0.160052 |
| Reactome | R-HSA-888590 | GABA synthesis, release, reuptake and degradation | http://reactome.org/PathwayBrowser/#/R-HSA-888590 | 19 | 4 | 0.390658 | 10.23915 | 5.29E-04 | 10815;2571;6616;6804 | SNAP25;GAD1;CPLX1;STX1A | 0.166239 |
| WikiPathway | WP179 | Cell Cycle | http://www.wikipathways.org/wpi/PathwayWidget.php?id=WP179&label[]=CDKN2A&label[]=PTTG1&label[]=MCM2&label[]=MAD2L2&label[]=ANAPC5&label[]=PRKDC&label[]=E2F1&label[]=PKMYT1&label[]=CCNE1&label[]=CDC20&label[]=CDC25B&label[]=CDK4&colors=steelblue | 122 | 12 | 2.880939 | 4.165309 | 2.77E-05 | 4171;991;994;9232;1869;9088;898;51433;10459;1019;5591;1029 | CDKN2A;PTTG1;MCM2;MAD2L2;ANAPC5;PRKDC;E2F1;PKMYT1;CCNE1;CDC20;CDC25B;CDK4 | 0.015245 |
| WikiPathway | WP2267 | Synaptic Vesicle Pathway | http://www.wikipathways.org/wpi/PathwayWidget.php?id=WP2267&label[]=SLC18A1&label[]=SNAP25&label[]=UNC13A&label[]=CPLX2&label[]=CPLX1&label[]=STX1A&label[]=SYP&colors=steelblue | 52 | 7 | 1.227941 | 5.700599 | 1.97E-04 | 10815;10814;6570;23025;6804;6855;6616 | SLC18A1;SNAP25;UNC13A;CPLX2;CPLX1;STX1A;SYP | 0.054377 |

| **Table S7: Differentially Expressed Genes in PNET** | | | |
| --- | --- | --- | --- |
|  |  |  |  |
| **Name** | **ENSEMBL_ID** | **Gene Symbol** | **Expression** |
| tetraspanin 6(TSPAN6) | ENSG00000000003 | TSPAN6 | DOWN |
| cystic fibrosis transmembrane conductance regulator(CFTR) | ENSG00000001626 | CFTR | DOWN |
| myotubularin related protein 7(MTMR7) | ENSG00000003987 | MTMR7 | UP |
| aristaless related homeobox(ARX) | ENSG00000004848 | ARX | UP |
| WD repeat domain 54(WDR54) | ENSG00000005448 | WDR54 | UP |
| transmembrane protein 98(TMEM98) | ENSG00000006042 | TMEM98 | DOWN |
| ATP binding cassette subfamily C member 8(ABCC8) | ENSG00000006071 | ABCC8 | UP |
| USH1 protein network component harmonin(USH1C) | ENSG00000006611 | USH1C | UP |
| prominin 1(PROM1) | ENSG00000007062 | PROM1 | DOWN |
| KIAA0100(KIAA0100) | ENSG00000007202 | KIAA0100 | UP |
| calcium voltage-gated channel auxiliary subunit alpha2delta 2(CACNA2D2) | ENSG00000007402 | CACNA2D2 | UP |
| synapsin I(SYN1) | ENSG00000008056 | SYN1 | UP |
| calcium/calmodulin dependent protein kinase IG(CAMK1G) | ENSG00000008118 | CAMK1G | DOWN |
| microsomal glutathione S-transferase 1(MGST1) | ENSG00000008394 | MGST1 | DOWN |
| interleukin 32(IL32) | ENSG00000008517 | IL32 | DOWN |
| iduronate 2-sulfatase(IDS) | ENSG00000010404 | IDS | UP |
| protease, serine 3(PRSS3) | ENSG00000010438 | PRSS3 | DOWN |
| leucine rich repeat containing 23(LRRC23) | ENSG00000010626 | LRRC23 | UP |
| synaptotagmin 7(SYT7) | ENSG00000011347 | SYT7 | UP |
| decorin(DCN) | ENSG00000011465 | DCN | DOWN |
| glutaminyl-peptide cyclotransferase like(QPCTL) | ENSG00000011478 | QPCTL | DOWN |
| solute carrier family 7 member 14(SLC7A14) | ENSG00000013293 | SLC7A14 | UP |
| myotubularin related protein 11(MTMR11) | ENSG00000014914 | MTMR11 | UP |
| dipeptidase 1 (renal)(DPEP1) | ENSG00000015413 | DPEP1 | DOWN |
| solute carrier family 38 member 5(SLC38A5) | ENSG00000017483 | SLC38A5 | DOWN |
| RUN and FYVE domain containing 3(RUFY3) | ENSG00000018189 | RUFY3 | UP |
| contactin 1(CNTN1) | ENSG00000018236 | CNTN1 | UP |
| synaptotagmin 13(SYT13) | ENSG00000019505 | SYT13 | UP |
| serpin family B member 1(SERPINB1) | ENSG00000021355 | SERPINB1 | DOWN |
| high mobility group box 3(HMGB3) | ENSG00000029993 | HMGB3 | UP |
| GABA type A receptor associated protein like 2(GABARAPL2) | ENSG00000034713 | GABARAPL2 | UP |
| cadherin 10(CDH10) | ENSG00000040731 | CDH10 | UP |
| LIM domain only 3(LMO3) | ENSG00000048540 | LMO3 | DOWN |
| protein phosphatase 1 regulatory subunit 3F(PPP1R3F) | ENSG00000049769 | PPP1R3F | UP |
| cytochrome b-245 alpha chain(CYBA) | ENSG00000051523 | CYBA | DOWN |
| protease, serine 8(PRSS8) | ENSG00000052344 | PRSS8 | DOWN |
| protein tyrosine phosphatase, receptor type N(PTPRN) | ENSG00000054356 | PTPRN | UP |
| forkhead box C1(FOXC1) | ENSG00000054598 | FOXC1 | DOWN |
| spectrin repeat containing nuclear envelope protein 2(SYNE2) | ENSG00000054654 | SYNE2 | DOWN |
| potassium voltage-gated channel subfamily H member 2(KCNH2) | ENSG00000055118 | KCNH2 | UP |
| cytoplasmic FMR1 interacting protein 2(CYFIP2) | ENSG00000055163 | CYFIP2 | UP |
| mucolipin 3(MCOLN3) | ENSG00000055732 | MCOLN3 | DOWN |
| coagulation factor VII(F7) | ENSG00000057593 | F7 | UP |
| calcium/calmodulin dependent protein kinase II beta(CAMK2B) | ENSG00000058404 | CAMK2B | UP |
| Y-box binding protein 3(YBX3) | ENSG00000060138 | YBX3 | DOWN |
| RIMS binding protein 2(RIMBP2) | ENSG00000060709 | RIMBP2 | UP |
| branched chain amino acid transaminase 1(BCAT1) | ENSG00000060982 | BCAT1 | DOWN |
| sperm associated antigen 4(SPAG4) | ENSG00000061656 | SPAG4 | DOWN |
| seizure related 6 homolog(SEZ6) | ENSG00000063015 | SEZ6 | UP |
| LIM and calponin homology domains 1(LIMCH1) | ENSG00000064042 | LIMCH1 | UP |
| sperm autoantigenic protein 17(SPA17) | ENSG00000064199 | SPA17 | UP |
| lysophosphatidic acid receptor 2(LPAR2) | ENSG00000064547 | LPAR2 | DOWN |
| calponin 2(CNN2) | ENSG00000064666 | CNN2 | DOWN |
| synaptosome associated protein 91(SNAP91) | ENSG00000065609 | SNAP91 | UP |
| myosin IXA(MYO9A) | ENSG00000066933 | MYO9A | UP |
| phosphofructokinase, platelet(PFKP) | ENSG00000067057 | PFKP | UP |
| pyruvate kinase, muscle(PKM) | ENSG00000067225 | PKM | UP |
| PDZ domain containing 4(PDZD4) | ENSG00000067840 | PDZD4 | UP |
| fibroblast growth factor receptor 3(FGFR3) | ENSG00000068078 | FGFR3 | DOWN |
| inositol hexakisphosphate kinase 2(IP6K2) | ENSG00000068745 | IP6K2 | DOWN |
| phospholipase A2 group X(PLA2G10) | ENSG00000069764 | PLA2G10 | DOWN |
| adaptor related protein complex 3 mu 2 subunit(AP3M2) | ENSG00000070718 | AP3M2 | UP |
| SEL1L ERAD E3 ligase adaptor subunit(SEL1L) | ENSG00000071537 | SEL1L | DOWN |
| adhesion G protein-coupled receptor L1(ADGRL1) | ENSG00000072071 | ADGRL1 | UP |
| collapsin response mediator protein 1(CRMP1) | ENSG00000072832 | CRMP1 | UP |
| gasdermin B(GSDMB) | ENSG00000073605 | GSDMB | DOWN |
| protein phosphatase 2 regulatory subunit Bgamma(PPP2R2C) | ENSG00000074211 | PPP2R2C | UP |
| TEA domain transcription factor 2(TEAD2) | ENSG00000074219 | TEAD2 | DOWN |
| carbonic anhydrase 12(CA12) | ENSG00000074410 | CA12 | DOWN |
| Rho guanine nucleotide exchange factor 10 like(ARHGEF10L) | ENSG00000074964 | ARHGEF10L | DOWN |
| tumor protein D52(TPD52) | ENSG00000076554 | TPD52 | UP |
| glypican 4(GPC4) | ENSG00000076716 | GPC4 | DOWN |
| actin like 6B(ACTL6B) | ENSG00000077080 | ACTL6B | UP |
| fibulin 1(FBLN1) | ENSG00000077942 | FBLN1 | DOWN |
| amphiphysin(AMPH) | ENSG00000078053 | AMPH | UP |
| endothelin 1(EDN1) | ENSG00000078401 | EDN1 | DOWN |
| MAP kinase interacting serine/threonine kinase 1(MKNK1) | ENSG00000079277 | MKNK1 | DOWN |
| carcinoembryonic antigen related cell adhesion molecule 1(CEACAM1) | ENSG00000079385 | CEACAM1 | UP |
| secretin receptor(SCTR) | ENSG00000080293 | SCTR | DOWN |
| solute carrier family 4 member 4(SLC4A4) | ENSG00000080493 | SLC4A4 | DOWN |
| C-X-C motif chemokine ligand 2(CXCL2) | ENSG00000081041 | CXCL2 | DOWN |
| arginase 2(ARG2) | ENSG00000081181 | ARG2 | UP |
| TNF receptor associated factor 5(TRAF5) | ENSG00000082512 | TRAF5 | DOWN |
| grainyhead like transcription factor 2(GRHL2) | ENSG00000083307 | GRHL2 | DOWN |
| EFR3 homolog B(EFR3B) | ENSG00000084710 | EFR3B | UP |
| MDS1 and EVI1 complex locus(MECOM) | ENSG00000085276 | MECOM | DOWN |
| oviductal glycoprotein 1(OVGP1) | ENSG00000085465 | OVGP1 | UP |
| ATP binding cassette subfamily B member 1(ABCB1) | ENSG00000085563 | ABCB1 | UP |
| hydroxysteroid 17-beta dehydrogenase 2(HSD17B2) | ENSG00000086696 | HSD17B2 | DOWN |
| linker for activation of T-cells family member 2(LAT2) | ENSG00000086730 | LAT2 | UP |
| hydroxysteroid 17-beta dehydrogenase 14(HSD17B14) | ENSG00000087076 | HSD17B14 | UP |
| regulatory factor X2(RFX2) | ENSG00000087903 | RFX2 | UP |
| ArfGAP with SH3 domain, ankyrin repeat and PH domain 3(ASAP3) | ENSG00000088280 | ASAP3 | DOWN |
| coagulation factor XI(F11) | ENSG00000088926 | F11 | DOWN |
| tescalcin(TESC) | ENSG00000088992 | TESC | DOWN |
| chromogranin B(CHGB) | ENSG00000089199 | CHGB | UP |
| poly(A) binding protein cytoplasmic 4(PABPC4) | ENSG00000090621 | PABPC4 | DOWN |
| delta like canonical Notch ligand 3(DLL3) | ENSG00000090932 | DLL3 | UP |
| N-acetyltransferase 14 (putative)(NAT14) | ENSG00000090971 | NAT14 | UP |
| Rap guanine nucleotide exchange factor 4(RAPGEF4) | ENSG00000091428 | RAPGEF4 | UP |
| SEL1L family member 3(SEL1L3) | ENSG00000091490 | SEL1L3 | DOWN |
| apolipoprotein H(APOH) | ENSG00000091583 | APOH | UP |
| carboxypeptidase A1(CPA1) | ENSG00000091704 | CPA1 | DOWN |
| regulator of G-protein signaling 17(RGS17) | ENSG00000091844 | RGS17 | UP |
| angiopoietin 2(ANGPT2) | ENSG00000091879 | ANGPT2 | UP |
| CD200 molecule(CD200) | ENSG00000091972 | CD200 | UP |
| solute carrier family 22 member 17(SLC22A17) | ENSG00000092096 | SLC22A17 | UP |
| phosphoglycerate dehydrogenase(PHGDH) | ENSG00000092621 | PHGDH | DOWN |
| erythrocyte membrane protein band 4.1 like 4B(EPB41L4B) | ENSG00000095203 | EPB41L4B | DOWN |
| carnitine O-acetyltransferase(CRAT) | ENSG00000095321 | CRAT | DOWN |
| progastricsin(PGC) | ENSG00000096088 | PGC | DOWN |
| inositol 1,4,5-trisphosphate receptor type 3(ITPR3) | ENSG00000096433 | ITPR3 | DOWN |
| topoisomerase (DNA) III beta(TOP3B) | ENSG00000100038 | TOP3B | DOWN |
| galectin 2(LGALS2) | ENSG00000100079 | LGALS2 | DOWN |
| glycine C-acetyltransferase(GCAT) | ENSG00000100116 | GCAT | DOWN |
| RAB36, member RAS oncogene family(RAB36) | ENSG00000100228 | RAB36 | UP |
| lipase maturation factor 2(LMF2) | ENSG00000100258 | LMF2 | DOWN |
| protein kinase C and casein kinase substrate in neurons 2(PACSIN2) | ENSG00000100266 | PACSIN2 | DOWN |
| myotubularin related protein 3(MTMR3) | ENSG00000100330 | MTMR3 | DOWN |
| cochlin(COCH) | ENSG00000100473 | COCH | DOWN |
| chromogranin A(CHGA) | ENSG00000100604 | CHGA | UP |
| solute carrier family 8 member A3(SLC8A3) | ENSG00000100678 | SLC8A3 | UP |
| phospholipid transfer protein(PLTP) | ENSG00000100979 | PLTP | DOWN |
| ubiquinol-cytochrome c reductase complex assembly factor 1(UQCC1) | ENSG00000101019 | UQCC1 | UP |
| prefoldin subunit 4(PFDN4) | ENSG00000101132 | PFDN4 | UP |
| eukaryotic translation elongation factor 1 alpha 2(EEF1A2) | ENSG00000101210 | EEF1A2 | UP |
| tribbles pseudokinase 3(TRIB3) | ENSG00000101255 | TRIB3 | UP |
| histocompatibility minor 13(HM13) | ENSG00000101294 | HM13 | DOWN |
| nucleolar protein 4(NOL4) | ENSG00000101746 | NOL4 | UP |
| matrix remodeling associated 5(MXRA5) | ENSG00000101825 | MXRA5 | DOWN |
| phosphoribosyl pyrophosphate synthetase 2(PRPS2) | ENSG00000101911 | PRPS2 | DOWN |
| sushi repeat containing protein, X-linked(SRPX) | ENSG00000101955 | SRPX | DOWN |
| synaptophysin(SYP) | ENSG00000102003 | SYP | UP |
| zinc finger CCCH-type containing 12B(ZC3H12B) | ENSG00000102053 | ZC3H12B | UP |
| proprotein convertase subtilisin/kexin type 1 inhibitor(PCSK1N) | ENSG00000102109 | PCSK1N | UP |
| fibroblast growth factor 14(FGF14) | ENSG00000102466 | FGF14 | UP |
| solute carrier family 25 member 15(SLC25A15) | ENSG00000102743 | SLC25A15 | DOWN |
| katanin catalytic subunit A1 like 1(KATNAL1) | ENSG00000102781 | KATNAL1 | UP |
| dehydrogenase/reductase 12(DHRS12) | ENSG00000102796 | DHRS12 | DOWN |
| olfactomedin 4(OLFM4) | ENSG00000102837 | OLFM4 | DOWN |
| NDRG family member 4(NDRG4) | ENSG00000103034 | NDRG4 | UP |
| crystallin mu(CRYM) | ENSG00000103316 | CRYM | UP |
| aquaporin 8(AQP8) | ENSG00000103375 | AQP8 | DOWN |
| quinolinate phosphoribosyltransferase(QPRT) | ENSG00000103485 | QPRT | DOWN |
| NODAL modulator 1(NOMO1) | ENSG00000103512 | NOMO1 | DOWN |
| synaptotagmin 17(SYT17) | ENSG00000103528 | SYT17 | UP |
| cathepsin H(CTSH) | ENSG00000103811 | CTSH | DOWN |
| family with sequence similarity 189 member A1(FAM189A1) | ENSG00000104059 | FAM189A1 | DOWN |
| secretogranin III(SCG3) | ENSG00000104112 | SCG3 | UP |
| neurocalcin delta(NCALD) | ENSG00000104490 | NCALD | UP |
| eukaryotic translation elongation factor 1 delta(EEF1D) | ENSG00000104529 | EEF1D | DOWN |
| solute carrier family 39 member 14(SLC39A14) | ENSG00000104635 | SLC39A14 | DOWN |
| tumor suppressor candidate 3(TUSC3) | ENSG00000104723 | TUSC3 | UP |
| fibrinogen like 1(FGL1) | ENSG00000104760 | FGL1 | DOWN |
| dihydrodiol dehydrogenase(DHDH) | ENSG00000104808 | DHDH | UP |
| lin-7 homolog B, crumbs cell polarity complex component(LIN7B) | ENSG00000104863 | LIN7B | UP |
| CAP-Gly domain containing linker protein 3(CLIP3) | ENSG00000105270 | CLIP3 | UP |
| amyloid beta precursor like protein 1(APLP1) | ENSG00000105290 | APLP1 | UP |
| phospholipid phosphatase related 2(PLPPR2) | ENSG00000105520 | PLPPR2 | UP |
| ATPase H+/K+ transporting alpha subunit(ATP4A) | ENSG00000105675 | ATP4A | DOWN |
| transmembrane protein 59 like(TMEM59L) | ENSG00000105696 | TMEM59L | UP |
| RUN domain containing 3B(RUNDC3B) | ENSG00000105784 | RUNDC3B | UP |
| caveolin 2(CAV2) | ENSG00000105971 | CAV2 | DOWN |
| LFNG O-fucosylpeptide 3-beta-N-acetylglucosaminyltransferase(LFNG) | ENSG00000106003 | LFNG | DOWN |
| syntaxin 1A(STX1A) | ENSG00000106089 | STX1A | UP |
| EPH receptor B6(EPHB6) | ENSG00000106123 | EPHB6 | DOWN |
| core 1 synthase, glycoprotein-N-acetylgalactosamine 3-beta-galactosyltransferase 1(C1GALT1) | ENSG00000106392 | C1GALT1 | UP |
| retinoic acid receptor responder 2(RARRES2) | ENSG00000106538 | RARRES2 | DOWN |
| anterior gradient 2, protein disulphide isomerase family member(AGR2) | ENSG00000106541 | AGR2 | DOWN |
| prune homolog 2(PRUNE2) | ENSG00000106772 | PRUNE2 | UP |
| SH3 domain containing GRB2 like 2, endophilin A1(SH3GL2) | ENSG00000107295 | SH3GL2 | UP |
| prostaglandin D2 synthase(PTGDS) | ENSG00000107317 | PTGDS | DOWN |
| C-X-C motif chemokine ligand 12(CXCL12) | ENSG00000107562 | CXCL12 | DOWN |
| cadherin related 23(CDH23) | ENSG00000107736 | CDH23 | DOWN |
| DnaJ heat shock protein family (Hsp40) member C12(DNAJC12) | ENSG00000108176 | DNAJC12 | UP |
| RUN domain containing 3A(RUNDC3A) | ENSG00000108309 | RUNDC3A | UP |
| purinergic receptor P2X 1(P2RX1) | ENSG00000108405 | P2RX1 | DOWN |
| calmodulin binding transcription activator 2(CAMTA2) | ENSG00000108509 | CAMTA2 | DOWN |
| mediator complex subunit 13(MED13) | ENSG00000108510 | MED13 | DOWN |
| B9 domain containing 1(B9D1) | ENSG00000108641 | B9D1 | UP |
| C-C motif chemokine ligand 2(CCL2) | ENSG00000108691 | CCL2 | DOWN |
| ATP binding cassette subfamily C member 3(ABCC3) | ENSG00000108846 | ABCC3 | DOWN |
| protein kinase cAMP-dependent type I regulatory subunit alpha(PRKAR1A) | ENSG00000108946 | PRKAR1A | UP |
| transmembrane protein 97(TMEM97) | ENSG00000109084 | TMEM97 | DOWN |
| mitogen-activated protein kinase 10(MAPK10) | ENSG00000109339 | MAPK10 | UP |
| carboxypeptidase E(CPE) | ENSG00000109472 | CPE | UP |
| wolframin ER transmembrane glycoprotein(WFS1) | ENSG00000109501 | WFS1 | UP |
| superoxide dismutase 3, extracellular(SOD3) | ENSG00000109610 | SOD3 | DOWN |
| DEAD-box helicase 25(DDX25) | ENSG00000109832 | DDX25 | UP |
| crystallin alpha B(CRYAB) | ENSG00000109846 | CRYAB | DOWN |
| beta-1,3-glucuronyltransferase 1(B3GAT1) | ENSG00000109956 | B3GAT1 | DOWN |
| cholecystokinin B receptor(CCKBR) | ENSG00000110148 | CCKBR | DOWN |
| centrosomal protein 126(CEP126) | ENSG00000110318 | CEP126 | UP |
| T-cell immune regulator 1, ATPase H+ transporting V0 subunit a3(TCIRG1) | ENSG00000110719 | TCIRG1 | DOWN |
| sodium channel epithelial 1 alpha subunit(SCNN1A) | ENSG00000111319 | SCNN1A | DOWN |
| enolase 2(ENO2) | ENSG00000111674 | ENO2 | UP |
| neural precursor cell expressed, developmentally down-regulated 9(NEDD9) | ENSG00000111859 | NEDD9 | UP |
| tumor protein D52-like 1(TPD52L1) | ENSG00000111907 | TPD52L1 | DOWN |
| geminin, DNA replication inhibitor(GMNN) | ENSG00000112312 | GMNN | DOWN |
| ARFGEF family member 3(ARFGEF3) | ENSG00000112379 | ARFGEF3 | UP |
| solute carrier family 16 member 10(SLC16A10) | ENSG00000112394 | SLC16A10 | DOWN |
| PARK2 coregulated(PACRG) | ENSG00000112530 | PACRG | UP |
| SPARC related modular calcium binding 2(SMOC2) | ENSG00000112562 | SMOC2 | DOWN |
| family with sequence similarity 46 member A(FAM46A) | ENSG00000112773 | FAM46A | UP |
| protease, serine 16(PRSS16) | ENSG00000112812 | PRSS16 | DOWN |
| complement C7(C7) | ENSG00000112936 | C7 | DOWN |
| NME/NM23 family member 5(NME5) | ENSG00000112981 | NME5 | UP |
| phosphodiesterase 4D(PDE4D) | ENSG00000113448 | PDE4D | UP |
| H2A histone family member Y(H2AFY) | ENSG00000113648 | H2AFY | UP |
| retinol binding protein 1(RBP1) | ENSG00000114115 | RBP1 | DOWN |
| serpin family I member 2(SERPINI2) | ENSG00000114204 | SERPINI2 | DOWN |
| chromosome 3 open reading frame 14(C3orf14) | ENSG00000114405 | C3orf14 | UP |
| chromosome 3 open reading frame 52(C3orf52) | ENSG00000114529 | C3orf52 | DOWN |
| podocalyxin like 2(PODXL2) | ENSG00000114631 | PODXL2 | UP |
| ATP binding cassette subfamily C member 5(ABCC5) | ENSG00000114770 | ABCC5 | UP |
| deoxyguanosine kinase(DGUOK) | ENSG00000114956 | DGUOK | UP |
| STEAP3 metalloreductase(STEAP3) | ENSG00000115107 | STEAP3 | DOWN |
| glucagon(GCG) | ENSG00000115263 | GCG | DOWN |
| growth factor receptor bound protein 14(GRB14) | ENSG00000115290 | GRB14 | DOWN |
| acyl-CoA dehydrogenase, long chain(ACADL) | ENSG00000115361 | ACADL | DOWN |
| eva-1 homolog A, regulator of programmed cell death(EVA1A) | ENSG00000115363 | EVA1A | DOWN |
| regenerating family member 1 alpha(REG1A) | ENSG00000115386 | REG1A | DOWN |
| hippocalcin like 1(HPCAL1) | ENSG00000115756 | HPCAL1 | DOWN |
| glutaminyl-peptide cyclotransferase(QPCT) | ENSG00000115828 | QPCT | UP |
| KISS1 receptor(KISS1R) | ENSG00000116014 | KISS1R | DOWN |
| ALMS1, centrosome and basal body associated protein(ALMS1) | ENSG00000116127 | ALMS1 | DOWN |
| microtubule affinity regulating kinase 1(MARK1) | ENSG00000116141 | MARK1 | UP |
| spermidine synthase(SRM) | ENSG00000116649 | SRM | DOWN |
| cystathionine gamma-lyase(CTH) | ENSG00000116761 | CTH | DOWN |
| nuclear receptor subfamily 5 group A member 2(NR5A2) | ENSG00000116833 | NR5A2 | DOWN |
| regulator of G-protein signaling 4(RGS4) | ENSG00000117152 | RGS4 | UP |
| coagulation factor III, tissue factor(F3) | ENSG00000117525 | F3 | DOWN |
| phospholipid phosphatase related 4(PLPPR4) | ENSG00000117600 | PLPPR4 | UP |
| mannosidase alpha class 1C member 1(MAN1C1) | ENSG00000117643 | MAN1C1 | UP |
| beta-1,4-galactosyltransferase 6(B4GALT6) | ENSG00000118276 | B4GALT6 | UP |
| lymphoid restricted membrane protein(LRMP) | ENSG00000118308 | LRMP | DOWN |
| ELOVL fatty acid elongase 4(ELOVL4) | ENSG00000118402 | ELOVL4 | UP |
| SH3 domain GRB2 like endophilin interacting protein 1(SGIP1) | ENSG00000118473 | SGIP1 | UP |
| A-kinase anchoring protein 7(AKAP7) | ENSG00000118507 | AKAP7 | DOWN |
| serum/glucocorticoid regulated kinase 1(SGK1) | ENSG00000118515 | SGK1 | DOWN |
| secreted phosphoprotein 1(SPP1) | ENSG00000118785 | SPP1 | DOWN |
| Kruppel like factor 12(KLF12) | ENSG00000118922 | KLF12 | UP |
| interferon alpha inducible protein 27 like 2(IFI27L2) | ENSG00000119632 | IFI27L2 | UP |
| protein phosphatase 4 regulatory subunit 4(PPP4R4) | ENSG00000119698 | PPP4R4 | UP |
| secreted frizzled related protein 5(SFRP5) | ENSG00000120057 | SFRP5 | DOWN |
| glutamate ionotropic receptor AMPA type subunit 2(GRIA2) | ENSG00000120251 | GRIA2 | UP |
| protocadherin beta 10(PCDHB10) | ENSG00000120324 | PCDHB10 | UP |
| potassium voltage-gated channel subfamily J member 5(KCNJ5) | ENSG00000120457 | KCNJ5 | DOWN |
| testis expressed 11(TEX11) | ENSG00000120498 | TEX11 | DOWN |
| retinol dehydrogenase 10 (all-trans)(RDH10) | ENSG00000121039 | RDH10 | DOWN |
| KIAA0922(KIAA0922) | ENSG00000121210 | KIAA0922 | DOWN |
| phospholipase B domain containing 1(PLBD1) | ENSG00000121316 | PLBD1 | DOWN |
| catalase(CAT) | ENSG00000121691 | CAT | DOWN |
| fibromodulin(FMOD) | ENSG00000122176 | FMOD | DOWN |
| ciliary neurotrophic factor receptor(CNTFR) | ENSG00000122756 | CNTFR | DOWN |
| BicC family RNA binding protein 1(BICC1) | ENSG00000122870 | BICC1 | DOWN |
| protein kinase N1(PKN1) | ENSG00000123143 | PKN1 | UP |
| adhesion G protein-coupled receptor E5(ADGRE5) | ENSG00000123146 | ADGRE5 | DOWN |
| RAB9B, member RAS oncogene family(RAB9B) | ENSG00000123570 | RAB9B | UP |
| coenzyme Q8B(COQ8B) | ENSG00000123815 | COQ8B | UP |
| enkurin domain containing 1(ENKD1) | ENSG00000124074 | ENKD1 | DOWN |
| solute carrier family 12 member 5(SLC12A5) | ENSG00000124140 | SLC12A5 | UP |
| syndecan 4(SDC4) | ENSG00000124145 | SDC4 | DOWN |
| recombination signal binding protein for immunoglobulin kappa J region like(RBPJL) | ENSG00000124232 | RBPJL | DOWN |
| glycine N-methyltransferase(GNMT) | ENSG00000124713 | GNMT | DOWN |
| metallothionein 1G(MT1G) | ENSG00000125144 | MT1G | DOWN |
| metallothionein 2A(MT2A) | ENSG00000125148 | MT2A | DOWN |
| SRY-box 9(SOX9) | ENSG00000125398 | SOX9 | DOWN |
| FosB proto-oncogene, AP-1 transcription factor subunit(FOSB) | ENSG00000125740 | FOSB | DOWN |
| optic atrophy 3 (autosomal recessive, with chorea and spastic paraplegia)(OPA3) | ENSG00000125741 | OPA3 | UP |
| NSF attachment protein beta(NAPB) | ENSG00000125814 | NAPB | UP |
| thioredoxin related transmembrane protein 4(TMX4) | ENSG00000125827 | TMX4 | UP |
| ribosome binding protein 1(RRBP1) | ENSG00000125844 | RRBP1 | DOWN |
| proprotein convertase subtilisin/kexin type 2(PCSK2) | ENSG00000125851 | PCSK2 | UP |
| lysosomal associated membrane protein family member 5(LAMP5) | ENSG00000125869 | LAMP5 | UP |
| transmembrane protein 74B(TMEM74B) | ENSG00000125895 | TMEM74B | UP |
| gastrin releasing peptide receptor(GRPR) | ENSG00000126010 | GRPR | DOWN |
| coagulation factor X(F10) | ENSG00000126218 | F10 | UP |
| kin of IRRE like 2 (Drosophila)(KIRREL2) | ENSG00000126259 | KIRREL2 | DOWN |
| BCL2 like 12(BCL2L12) | ENSG00000126453 | BCL2L12 | DOWN |
| WNK lysine deficient protein kinase 4(WNK4) | ENSG00000126562 | WNK4 | UP |
| fibroblast growth factor receptor-like 1(FGFRL1) | ENSG00000127418 | FGFRL1 | DOWN |
| F-box and leucine rich repeat protein 16(FBXL16) | ENSG00000127585 | FBXL16 | UP |
| paroxysmal nonkinesigenic dyskinesia(PNKD) | ENSG00000127838 | PNKD | UP |
| G protein subunit alpha i1(GNAI1) | ENSG00000127955 | GNAI1 | UP |
| G protein subunit alpha z(GNAZ) | ENSG00000128266 | GNAZ | UP |
| tyrosylprotein sulfotransferase 2(TPST2) | ENSG00000128294 | TPST2 | DOWN |
| mercaptopyruvate sulfurtransferase(MPST) | ENSG00000128309 | MPST | DOWN |
| cadherin related family member 3(CDHR3) | ENSG00000128536 | CDHR3 | UP |
| VGF nerve growth factor inducible(VGF) | ENSG00000128564 | VGF | UP |
| chimerin 1(CHN1) | ENSG00000128656 | CHN1 | UP |
| myosin VC(MYO5C) | ENSG00000128833 | MYO5C | DOWN |
| cingulin like 1(CGNL1) | ENSG00000128849 | CGNL1 | DOWN |
| tropomodulin 2(TMOD2) | ENSG00000128872 | TMOD2 | UP |
| ChaC glutathione specific gamma-glutamylcyclotransferase 1(CHAC1) | ENSG00000128965 | CHAC1 | DOWN |
| THAP domain containing 10(THAP10) | ENSG00000129028 | THAP10 | UP |
| ribonuclease A family member 1, pancreatic(RNASE1) | ENSG00000129538 | RNASE1 | DOWN |
| rhomboid 5 homolog 2(RHBDF2) | ENSG00000129667 | RHBDF2 | DOWN |
| fibroblast growth factor 13(FGF13) | ENSG00000129682 | FGF13 | UP |
| guanidinoacetate N-methyltransferase(GAMT) | ENSG00000130005 | GAMT | DOWN |
| SH3 domain binding protein 4(SH3BP4) | ENSG00000130147 | SH3BP4 | DOWN |
| leucine rich repeats and calponin homology domain containing 2(LRCH2) | ENSG00000130224 | LRCH2 | UP |
| calcyon neuron specific vesicular protein(CALY) | ENSG00000130643 | CALY | UP |
| cytochrome P450 family 2 subfamily E member 1(CYP2E1) | ENSG00000130649 | CYP2E1 | DOWN |
| sphingomyelin phosphodiesterase acid like 3B(SMPDL3B) | ENSG00000130768 | SMPDL3B | DOWN |
| hyaluronan binding protein 4(HABP4) | ENSG00000130956 | HABP4 | UP |
| regucalcin(RGN) | ENSG00000130988 | RGN | DOWN |
| GINS complex subunit 2(GINS2) | ENSG00000131153 | GINS2 | UP |
| proline rich 7, synaptic(PRR7) | ENSG00000131188 | PRR7 | UP |
| tensin 4(TNS4) | ENSG00000131746 | TNS4 | DOWN |
| protein phosphatase 1 regulatory inhibitor subunit 1B(PPP1R1B) | ENSG00000131771 | PPP1R1B | DOWN |
| GTP cyclohydrolase 1(GCH1) | ENSG00000131979 | GCH1 | UP |
| galectin 3(LGALS3) | ENSG00000131981 | LGALS3 | DOWN |
| tripartite motif containing 5(TRIM5) | ENSG00000132256 | TRIM5 | DOWN |
| serpin family F member 1(SERPINF1) | ENSG00000132386 | SERPINF1 | DOWN |
| discs large MAGUK scaffold protein 4(DLG4) | ENSG00000132535 | DLG4 | UP |
| receptor accessory protein 2(REEP2) | ENSG00000132563 | REEP2 | UP |
| synaptosome associated protein 25(SNAP25) | ENSG00000132639 | SNAP25 | UP |
| aminoacylase 3(ACY3) | ENSG00000132744 | ACY3 | DOWN |
| V-set and transmembrane domain containing 2 like(VSTM2L) | ENSG00000132821 | VSTM2L | UP |
| KN motif and ankyrin repeat domains 4(KANK4) | ENSG00000132854 | KANK4 | DOWN |
| synaptotagmin 4(SYT4) | ENSG00000132872 | SYT4 | UP |
| cholinergic receptor muscarinic 3(CHRM3) | ENSG00000133019 | CHRM3 | DOWN |
| brain expressed X-linked 2(BEX2) | ENSG00000133134 | BEX2 | UP |
| dual specificity phosphatase 26 (putative)(DUSP26) | ENSG00000133878 | DUSP26 | UP |
| regenerating family member 4(REG4) | ENSG00000134193 | REG4 | DOWN |
| vav guanine nucleotide exchange factor 3(VAV3) | ENSG00000134215 | VAV3 | DOWN |
| V-set domain containing T cell activation inhibitor 1(VTCN1) | ENSG00000134258 | VTCN1 | DOWN |
| spire type actin nucleation factor 1(SPIRE1) | ENSG00000134278 | SPIRE1 | UP |
| spexin hormone(SPX) | ENSG00000134548 | SPX | DOWN |
| gamma-glutamylamine cyclotransferase(GGACT) | ENSG00000134864 | GGACT | DOWN |
| claudin 10(CLDN10) | ENSG00000134873 | CLDN10 | DOWN |
| phosphoserine aminotransferase 1(PSAT1) | ENSG00000135069 | PSAT1 | DOWN |
| ring finger protein, transmembrane 2(RNFT2) | ENSG00000135119 | RNFT2 | UP |
| UDP glucuronosyltransferase family 2 member A3(UGT2A3) | ENSG00000135220 | UGT2A3 | DOWN |
| proline rich and Gla domain 4(PRRG4) | ENSG00000135378 | PRRG4 | DOWN |
| anti-Mullerian hormone receptor type 2(AMHR2) | ENSG00000135409 | AMHR2 | DOWN |
| protein kinase (cAMP-dependent, catalytic) inhibitor beta(PKIB) | ENSG00000135549 | PKIB | UP |
| family with sequence similarity 129 member A(FAM129A) | ENSG00000135842 | FAM129A | DOWN |
| serpin family E member 2(SERPINE2) | ENSG00000135919 | SERPINE2 | UP |
| NAC alpha domain containing(NACAD) | ENSG00000136274 | NACAD | UP |
| chondroadherin(CHAD) | ENSG00000136457 | CHAD | DOWN |
| GATA binding protein 4(GATA4) | ENSG00000136574 | GATA4 | DOWN |
| cripto, FRL-1, cryptic family 1(CFC1) | ENSG00000136698 | CFC1 | UP |
| tRNA methyltransferase O(TRMO) | ENSG00000136932 | TRMO | UP |
| ectonucleotide pyrophosphatase/phosphodiesterase 2(ENPP2) | ENSG00000136960 | ENPP2 | UP |
| v-myc avian myelocytomatosis viral oncogene homolog(MYC) | ENSG00000136997 | MYC | DOWN |
| KIAA0319(KIAA0319) | ENSG00000137261 | KIAA0319 | UP |
| solute carrier family 22 member 23(SLC22A23) | ENSG00000137266 | SLC22A23 | DOWN |
| colipase(CLPS) | ENSG00000137392 | CLPS | DOWN |
| FH2 domain containing 1(FHDC1) | ENSG00000137460 | FHDC1 | UP |
| matrix metallopeptidase 7(MMP7) | ENSG00000137673 | MMP7 | DOWN |
| poly(ADP-ribose) polymerase family member 6(PARP6) | ENSG00000137817 | PARP6 | UP |
| leucine rich repeat containing 49(LRRC49) | ENSG00000137821 | LRRC49 | UP |
| myocardial zonula adherens protein(MYZAP) | ENSG00000137878 | MYZAP | DOWN |
| GIPC PDZ domain containing family member 2(GIPC2) | ENSG00000137960 | GIPC2 | DOWN |
| solute carrier family 3 member 1(SLC3A1) | ENSG00000138079 | SLC3A1 | DOWN |
| ARP1 actin-related protein 1 homolog A, centractin alpha(ACTR1A) | ENSG00000138107 | ACTR1A | UP |
| CUB and zona pellucida like domains 1(CUZD1) | ENSG00000138161 | CUZD1 | DOWN |
| ADP ribosylation factor like GTPase 3(ARL3) | ENSG00000138175 | ARL3 | UP |
| phospholipase C epsilon 1(PLCE1) | ENSG00000138193 | PLCE1 | DOWN |
| zinc finger protein 365(ZNF365) | ENSG00000138311 | ZNF365 | UP |
| aldehyde oxidase 1(AOX1) | ENSG00000138356 | AOX1 | DOWN |
| HECT, C2 and WW domain containing E3 ubiquitin protein ligase 2(HECW2) | ENSG00000138411 | HECW2 | UP |
| sperm specific antigen 2(SSFA2) | ENSG00000138434 | SSFA2 | DOWN |
| guanylate cyclase activator 1C(GUCA1C) | ENSG00000138472 | GUCA1C | DOWN |
| cartilage intermediate layer protein(CILP) | ENSG00000138615 | CILP | DOWN |
| N-acylethanolamine acid amidase(NAAA) | ENSG00000138744 | NAAA | UP |
| Fraser extracellular matrix complex subunit 1(FRAS1) | ENSG00000138759 | FRAS1 | DOWN |
| annexin A3(ANXA3) | ENSG00000138772 | ANXA3 | DOWN |
| epidermal growth factor(EGF) | ENSG00000138798 | EGF | DOWN |
| solute carrier family 39 member 8(SLC39A8) | ENSG00000138821 | SLC39A8 | DOWN |
| endoplasmic reticulum protein 27(ERP27) | ENSG00000139055 | ERP27 | DOWN |
| solute carrier family 39 member 5(SLC39A5) | ENSG00000139540 | SLC39A5 | DOWN |
| somatostatin receptor 1(SSTR1) | ENSG00000139874 | SSTR1 | UP |
| NOVA alternative splicing regulator 1(NOVA1) | ENSG00000139910 | NOVA1 | UP |
| pellino E3 ubiquitin protein ligase family member 2(PELI2) | ENSG00000139946 | PELI2 | DOWN |
| reticulon 1(RTN1) | ENSG00000139970 | RTN1 | UP |
| fibulin 5(FBLN5) | ENSG00000140092 | FBLN5 | DOWN |
| serpin family A member 10(SERPINA10) | ENSG00000140093 | SERPINA10 | UP |
| SH3 domain containing GRB2 like 3, endophilin A3(SH3GL3) | ENSG00000140600 | SH3GL3 | UP |
| carbohydrate sulfotransferase 4(CHST4) | ENSG00000140835 | CHST4 | DOWN |
| chymotrypsin like(CTRL) | ENSG00000141086 | CTRL | DOWN |
| glucose-fructose oxidoreductase domain containing 2(GFOD2) | ENSG00000141098 | GFOD2 | UP |
| transducer of ERBB2, 1(TOB1) | ENSG00000141232 | TOB1 | DOWN |
| sperm acrosome associated 3(SPACA3) | ENSG00000141316 | SPACA3 | DOWN |
| inositol monophosphatase 2(IMPA2) | ENSG00000141401 | IMPA2 | DOWN |
| asialoglycoprotein receptor 1(ASGR1) | ENSG00000141505 | ASGR1 | UP |
| dymeclin(DYM) | ENSG00000141627 | DYM | DOWN |
| calcium voltage-gated channel subunit alpha1 A(CACNA1A) | ENSG00000141837 | CACNA1A | UP |
| nuclear factor I C(NFIC) | ENSG00000141905 | NFIC | DOWN |
| phospholipid phosphatase 2(PLPP2) | ENSG00000141934 | PLPP2 | DOWN |
| acid phosphatase, testicular(ACPT) | ENSG00000142513 | ACPT | DOWN |
| PR/SET domain 16(PRDM16) | ENSG00000142611 | PRDM16 | DOWN |
| chymotrypsin like elastase family member 2A(CELA2A) | ENSG00000142615 | CELA2A | DOWN |
| interleukin 22 receptor subunit alpha 1(IL22RA1) | ENSG00000142677 | IL22RA1 | DOWN |
| DMRT like family A2(DMRTA2) | ENSG00000142700 | DMRTA2 | DOWN |
| chymotrypsin like elastase family member 3A(CELA3A) | ENSG00000142789 | CELA3A | DOWN |
| dermatopontin(DPT) | ENSG00000143196 | DPT | DOWN |
| family with sequence similarity 163 member A(FAM163A) | ENSG00000143340 | FAM163A | UP |
| annexin A9(ANXA9) | ENSG00000143412 | ANXA9 | DOWN |
| epoxide hydrolase 1(EPHX1) | ENSG00000143819 | EPHX1 | DOWN |
| galactose mutarotase(GALM) | ENSG00000143891 | GALM | DOWN |
| regenerating family member 3 gamma(REG3G) | ENSG00000143954 | REG3G | DOWN |
| AF4/FMR2 family member 3(AFF3) | ENSG00000144218 | AFF3 | UP |
| atypical chemokine receptor 3(ACKR3) | ENSG00000144476 | ACKR3 | DOWN |
| oxysterol binding protein like 10(OSBPL10) | ENSG00000144645 | OSBPL10 | UP |
| leucine rich repeats and immunoglobulin like domains 1(LRIG1) | ENSG00000144749 | LRIG1 | DOWN |
| aldehyde dehydrogenase 1 family member L1(ALDH1L1) | ENSG00000144908 | ALDH1L1 | DOWN |
| transient receptor potential cation channel subfamily C member 1(TRPC1) | ENSG00000144935 | TRPC1 | UP |
| endothelin converting enzyme 2(ECE2) | ENSG00000145194 | ECE2 | DOWN |
| von Willebrand factor A domain containing 5B2(VWA5B2) | ENSG00000145198 | VWA5B2 | UP |
| placenta specific 8(PLAC8) | ENSG00000145287 | PLAC8 | UP |
| synuclein alpha(SNCA) | ENSG00000145335 | SNCA | UP |
| IQ motif containing GTPase activating protein 2(IQGAP2) | ENSG00000145703 | IQGAP2 | DOWN |
| TNF alpha induced protein 8(TNFAIP8) | ENSG00000145779 | TNFAIP8 | DOWN |
| von Willebrand factor D and EGF domains(VWDE) | ENSG00000146530 | VWDE | UP |
| insulin like growth factor binding protein 3(IGFBP3) | ENSG00000146674 | IGFBP3 | UP |
| tripartite motif containing 50(TRIM50) | ENSG00000146755 | TRIM50 | DOWN |
| chromosome X open reading frame 57(CXorf57) | ENSG00000147231 | CXorf57 | UP |
| glypican 3(GPC3) | ENSG00000147257 | GPC3 | DOWN |
| ST18, C2H2C-type zinc finger(ST18) | ENSG00000147488 | ST18 | UP |
| alcohol dehydrogenase, iron containing 1(ADHFE1) | ENSG00000147576 | ADHFE1 | DOWN |
| nuclear factor I B(NFIB) | ENSG00000147862 | NFIB | DOWN |
| interferon alpha 16(IFNA16) | ENSG00000147885 | IFNA16 | DOWN |
| chromosome 9 open reading frame 72(C9orf72) | ENSG00000147894 | C9orf72 | DOWN |
| phospholipid phosphatase related 1(PLPPR1) | ENSG00000148123 | PLPPR1 | UP |
| solute carrier family 25 member 25(SLC25A25) | ENSG00000148339 | SLC25A25 | DOWN |
| pleckstrin homology domain containing S1(PLEKHS1) | ENSG00000148735 | PLEKHS1 | DOWN |
| internexin neuronal intermediate filament protein alpha(INA) | ENSG00000148798 | INA | UP |
| serpin family G member 1(SERPING1) | ENSG00000149131 | SERPING1 | DOWN |
| solute carrier family 43 member 1(SLC43A1) | ENSG00000149150 | SLC43A1 | DOWN |
| suppression of tumorigenicity 14(ST14) | ENSG00000149418 | ST14 | DOWN |
| cleavage and polyadenylation specific factor 7(CPSF7) | ENSG00000149532 | CPSF7 | DOWN |
| glycoprotein hormone alpha 2(GPHA2) | ENSG00000149735 | GPHA2 | DOWN |
| LY6/PLAUR domain containing 6B(LYPD6B) | ENSG00000150556 | LYPD6B | DOWN |
| adrenoceptor alpha 2A(ADRA2A) | ENSG00000150594 | ADRA2A | DOWN |
| calcium voltage-gated channel subunit alpha1 C(CACNA1C) | ENSG00000151067 | CACNA1C | UP |
| methionine adenosyltransferase 1A(MAT1A) | ENSG00000151224 | MAT1A | DOWN |
| potassium channel tetramerization domain containing 14(KCTD14) | ENSG00000151364 | KCTD14 | DOWN |
| quinoid dihydropteridine reductase(QDPR) | ENSG00000151552 | QDPR | UP |
| ring finger protein 144A(RNF144A) | ENSG00000151692 | RNF144A | DOWN |
| transmembrane protein 45B(TMEM45B) | ENSG00000151715 | TMEM45B | UP |
| stress associated endoplasmic reticulum protein family member 2(SERP2) | ENSG00000151778 | SERP2 | UP |
| schwannomin interacting protein 1(SCHIP1) | ENSG00000151967 | SCHIP1 | UP |
| cripto, FRL-1, cryptic family 1B(CFC1B) | ENSG00000152093 | CFC1B | UP |
| ZFP36 ring finger protein like 2(ZFP36L2) | ENSG00000152518 | ZFP36L2 | DOWN |
| phosphofructokinase, muscle(PFKM) | ENSG00000152556 | PFKM | UP |
| calcyphosine like(CAPSL) | ENSG00000152611 | CAPSL | UP |
| cyclin O(CCNO) | ENSG00000152669 | CCNO | DOWN |
| hematopoietically expressed homeobox(HHEX) | ENSG00000152804 | HHEX | DOWN |
| carboxypeptidase B1(CPB1) | ENSG00000153002 | CPB1 | DOWN |
| calmegin(CLGN) | ENSG00000153132 | CLGN | UP |
| JAZF zinc finger 1(JAZF1) | ENSG00000153814 | JAZF1 | UP |
| junctophilin 3(JPH3) | ENSG00000154118 | JPH3 | UP |
| ATP binding cassette subfamily A member 5(ABCA5) | ENSG00000154265 | ABCA5 | UP |
| ubiquitin C-terminal hydrolase L1(UCHL1) | ENSG00000154277 | UCHL1 | UP |
| L-threonine dehydrogenase (pseudogene)(TDH) | ENSG00000154316 | TDH | DOWN |
| family with sequence similarity 167 member A(FAM167A) | ENSG00000154319 | FAM167A | UP |
| RAB6B, member RAS oncogene family(RAB6B) | ENSG00000154917 | RAB6B | UP |
| acyl-CoA synthetase short-chain family member 1(ACSS1) | ENSG00000154930 | ACSS1 | DOWN |
| prominin 2(PROM2) | ENSG00000155066 | PROM2 | DOWN |
| protein tyrosine phosphatase, receptor type N2(PTPRN2) | ENSG00000155093 | PTPRN2 | UP |
| transmembrane protein 55A(TMEM55A) | ENSG00000155099 | TMEM55A | UP |
| tRNA methyltransferase 44 homolog (S. cerevisiae)(TRMT44) | ENSG00000155275 | TRMT44 | DOWN |
| phosphoinositide-3-kinase adaptor protein 1(PIK3AP1) | ENSG00000155629 | PIK3AP1 | DOWN |
| formin 2(FMN2) | ENSG00000155816 | FMN2 | UP |
| RAB39B, member RAS oncogene family(RAB39B) | ENSG00000155961 | RAB39B | UP |
| tetraspanin 7(TSPAN7) | ENSG00000156298 | TSPAN7 | UP |
| tudor domain containing 9(TDRD9) | ENSG00000156414 | TDRD9 | UP |
| SH3 domain containing ring finger 2(SH3RF2) | ENSG00000156463 | SH3RF2 | DOWN |
| protein phosphatase 2 regulatory subunit Bbeta(PPP2R2B) | ENSG00000156475 | PPP2R2B | UP |
| hexokinase domain containing 1(HKDC1) | ENSG00000156510 | HKDC1 | DOWN |
| lipoma HMGIC fusion partner-like 4(LHFPL4) | ENSG00000156959 | LHFPL4 | UP |
| somatostatin(SST) | ENSG00000157005 | SST | DOWN |
| nicotinamide nucleotide adenylyltransferase 2(NMNAT2) | ENSG00000157064 | NMNAT2 | UP |
| potassium voltage-gated channel subfamily J member 6(KCNJ6) | ENSG00000157542 | KCNJ6 | UP |
| transmembrane protein 164(TMEM164) | ENSG00000157600 | TMEM164 | DOWN |
| solute carrier family 30 member 2(SLC30A2) | ENSG00000158014 | SLC30A2 | DOWN |
| histone cluster 1 H2B family member d(HIST1H2BD) | ENSG00000158373 | HIST1H2BD | UP |
| potassium voltage-gated channel subfamily B member 1(KCNB1) | ENSG00000158445 | KCNB1 | UP |
| carboxypeptidase A2(CPA2) | ENSG00000158516 | CPA2 | DOWN |
| glycerophosphodiester phosphodiesterase domain containing 5(GDPD5) | ENSG00000158555 | GDPD5 | DOWN |
| dynein cytoplasmic 1 intermediate chain 1(DYNC1I1) | ENSG00000158560 | DYNC1I1 | UP |
| nitrilase 1(NIT1) | ENSG00000158793 | NIT1 | DOWN |
| cache domain containing 1(CACHD1) | ENSG00000158966 | CACHD1 | DOWN |
| synaptic vesicle glycoprotein 2A(SV2A) | ENSG00000159164 | SV2A | UP |
| ladinin 1(LAD1) | ENSG00000159166 | LAD1 | DOWN |
| regulator of calcineurin 1(RCAN1) | ENSG00000159200 | RCAN1 | DOWN |
| chloride intracellular channel 6(CLIC6) | ENSG00000159212 | CLIC6 | DOWN |
| BTG anti-proliferation factor 2(BTG2) | ENSG00000159388 | BTG2 | DOWN |
| CUGBP, Elav-like family member 3(CELF3) | ENSG00000159409 | CELF3 | UP |
| ATP binding cassette subfamily G member 1(ABCG1) | ENSG00000160179 | ABCG1 | UP |
| trefoil factor 3(TFF3) | ENSG00000160180 | TFF3 | UP |
| radial spoke head 1 homolog(RSPH1) | ENSG00000160188 | RSPH1 | UP |
| phosphodiesterase 9A(PDE9A) | ENSG00000160191 | PDE9A | UP |
| cystathionine-beta-synthase(CBS) | ENSG00000160200 | CBS | DOWN |
| chromosome 21 open reading frame 33(C21orf33) | ENSG00000160221 | C21orf33 | UP |
| spectrin beta, non-erythrocytic 4(SPTBN4) | ENSG00000160460 | SPTBN4 | UP |
| BR serine/threonine kinase 1(BRSK1) | ENSG00000160469 | BRSK1 | UP |
| alpha-2-glycoprotein 1, zinc-binding(AZGP1) | ENSG00000160862 | AZGP1 | DOWN |
| cytochrome P450 family 11 subfamily B member 1(CYP11B1) | ENSG00000160882 | CYP11B1 | DOWN |
| sialic acid binding Ig like lectin 11(SIGLEC11) | ENSG00000161640 | SIGLEC11 | DOWN |
| BICD family like cargo adaptor 2(BICDL2) | ENSG00000162069 | BICDL2 | DOWN |
| SH3 and multiple ankyrin repeat domains 2(SHANK2) | ENSG00000162105 | SHANK2 | DOWN |
| PDZK1 interacting protein 1(PDZK1IP1) | ENSG00000162366 | PDZK1IP1 | DOWN |
| chymotrypsin C(CTRC) | ENSG00000162438 | CTRC | DOWN |
| aldo-keto reductase family 7 member A3(AKR7A3) | ENSG00000162482 | AKR7A3 | DOWN |
| KIAA1522(KIAA1522) | ENSG00000162522 | KIAA1522 | DOWN |
| calcium/calmodulin dependent protein kinase II inhibitor 1(CAMK2N1) | ENSG00000162545 | CAMK2N1 | UP |
| Wnt family member 4(WNT4) | ENSG00000162552 | WNT4 | UP |
| growth factor independent 1 transcriptional repressor(GFI1) | ENSG00000162676 | GFI1 | DOWN |
| potassium sodium-activated channel subfamily T member 2(KCNT2) | ENSG00000162687 | KCNT2 | UP |
| VANGL planar cell polarity protein 2(VANGL2) | ENSG00000162738 | VANGL2 | DOWN |
| peptidase M20 domain containing 1(PM20D1) | ENSG00000162877 | PM20D1 | DOWN |
| coenzyme Q8A(COQ8A) | ENSG00000163050 | COQ8A | DOWN |
| PBX homeobox interacting protein 1(PBXIP1) | ENSG00000163346 | PBXIP1 | UP |
| claudin 1(CLDN1) | ENSG00000163347 | CLDN1 | DOWN |
| chromosome 1 open reading frame 106(C1orf106) | ENSG00000163362 | C1orf106 | DOWN |
| tripartite motif containing 46(TRIM46) | ENSG00000163462 | TRIM46 | UP |
| adenosine A1 receptor(ADORA1) | ENSG00000163485 | ADORA1 | DOWN |
| fibulin 2(FBLN2) | ENSG00000163520 | FBLN2 | DOWN |
| neurofascin(NFASC) | ENSG00000163531 | NFASC | UP |
| islet cell autoantigen 1 like(ICA1L) | ENSG00000163596 | ICA1L | UP |
| CDP-diacylglycerol synthase 1(CDS1) | ENSG00000163624 | CDS1 | UP |
| albumin(ALB) | ENSG00000163631 | ALB | DOWN |
| transcription factor 23(TCF23) | ENSG00000163792 | TCF23 | DOWN |
| Kruppel like factor 15(KLF15) | ENSG00000163884 | KLF15 | DOWN |
| hes related family bHLH transcription factor with YRPW motif-like(HEYL) | ENSG00000163909 | HEYL | DOWN |
| solute carrier family 9 member B2(SLC9B2) | ENSG00000164038 | SLC9B2 | UP |
| bassoon presynaptic cytomatrix protein(BSN) | ENSG00000164061 | BSN | UP |
| hydroxyprostaglandin dehydrogenase 15-(NAD)(HPGD) | ENSG00000164120 | HPGD | UP |
| molybdenum cofactor synthesis 2(MOCS2) | ENSG00000164172 | MOCS2 | DOWN |
| serine peptidase inhibitor, Kazal type 1(SPINK1) | ENSG00000164266 | SPINK1 | DOWN |
| endothelial cell specific molecule 1(ESM1) | ENSG00000164283 | ESM1 | UP |
| forkhead box Q1(FOXQ1) | ENSG00000164379 | FOXQ1 | DOWN |
| transmembrane protein 200A(TMEM200A) | ENSG00000164484 | TMEM200A | UP |
| zinc finger protein 704(ZNF704) | ENSG00000164684 | ZNF704 | DOWN |
| defensin beta 1(DEFB1) | ENSG00000164825 | DEFB1 | DOWN |
| FRAS1 related extracellular matrix 1(FREM1) | ENSG00000164946 | FREM1 | DOWN |
| heparan-alpha-glucosaminide N-acetyltransferase(HGSNAT) | ENSG00000165102 | HGSNAT | DOWN |
| RAS and EF-hand domain containing(RASEF) | ENSG00000165105 | RASEF | DOWN |
| sushi, von Willebrand factor type A, EGF and pentraxin domain containing 1(SVEP1) | ENSG00000165124 | SVEP1 | DOWN |
| transient receptor potential cation channel subfamily V member 6(TRPV6) | ENSG00000165125 | TRPV6 | DOWN |
| fructose-bisphosphatase 1(FBP1) | ENSG00000165140 | FBP1 | DOWN |
| NADH:ubiquinone oxidoreductase subunit B6(NDUFB6) | ENSG00000165264 | NDUFB6 | UP |
| claudin 2(CLDN2) | ENSG00000165376 | CLDN2 | DOWN |
| PBX/knotted 1 homeobox 2(PKNOX2) | ENSG00000165495 | PKNOX2 | DOWN |
| protein kinase C and casein kinase substrate in neurons 3(PACSIN3) | ENSG00000165912 | PACSIN3 | DOWN |
| tandem C2 domains, nuclear(TC2N) | ENSG00000165929 | TC2N | DOWN |
| HtrA serine peptidase 1(HTRA1) | ENSG00000166033 | HTRA1 | UP |
| SV2 related protein(SVOP) | ENSG00000166111 | SVOP | UP |
| glutamic--pyruvic transaminase 2(GPT2) | ENSG00000166123 | GPT2 | DOWN |
| creatine kinase B(CKB) | ENSG00000166165 | CKB | UP |
| gamma-aminobutyric acid type A receptor beta3 subunit(GABRB3) | ENSG00000166206 | GABRB3 | UP |
| COX11, cytochrome c oxidase copper chaperone(COX11) | ENSG00000166260 | COX11 | UP |
| sphingomyelin phosphodiesterase 1(SMPD1) | ENSG00000166311 | SMPD1 | UP |
| cytochrome b5 type A(CYB5A) | ENSG00000166347 | CYB5A | DOWN |
| WD repeat domain 72(WDR72) | ENSG00000166415 | WDR72 | DOWN |
| transmembrane protein 130(TMEM130) | ENSG00000166448 | TMEM130 | UP |
| microfibrillar associated protein 4(MFAP4) | ENSG00000166482 | MFAP4 | DOWN |
| hepatoma-derived growth factor, related protein 3(HDGFRP3) | ENSG00000166503 | HDGFRP3 | UP |
| 5-hydroxytryptamine receptor 3A(HTR3A) | ENSG00000166736 | HTR3A | DOWN |
| perilipin 1(PLIN1) | ENSG00000166819 | PLIN1 | DOWN |
| alanyl aminopeptidase, membrane(ANPEP) | ENSG00000166825 | ANPEP | DOWN |
| ankyrin repeat and death domain containing 1A(ANKDD1A) | ENSG00000166839 | ANKDD1A | DOWN |
| membrane spanning 4-domains A8(MS4A8) | ENSG00000166959 | MS4A8 | UP |
| N-acetyltransferase 16 (putative)(NAT16) | ENSG00000167011 | NAT16 | UP |
| meiotic double-stranded break formation protein 1(MEI1) | ENSG00000167077 | MEI1 | DOWN |
| olfactory receptor family 51 subfamily E member 2(OR51E2) | ENSG00000167332 | OR51E2 | DOWN |
| zinc finger protein 226(ZNF226) | ENSG00000167380 | ZNF226 | UP |
| carbonic anhydrase 4(CA4) | ENSG00000167434 | CA4 | DOWN |
| tweety family member 1(TTYH1) | ENSG00000167614 | TTYH1 | DOWN |
| transmembrane protein 145(TMEM145) | ENSG00000167619 | TMEM145 | UP |
| gamma-glutamyltransferase 6(GGT6) | ENSG00000167741 | GGT6 | DOWN |
| kallikrein 1(KLK1) | ENSG00000167748 | KLK1 | DOWN |
| kallikrein related peptidase 11(KLK11) | ENSG00000167757 | KLK11 | DOWN |
| keratin 80(KRT80) | ENSG00000167767 | KRT80 | DOWN |
| succinate dehydrogenase complex assembly factor 2(SDHAF2) | ENSG00000167985 | SDHAF2 | UP |
| G protein subunit gamma 4(GNG4) | ENSG00000168243 | GNG4 | UP |
| pancreas specific transcription factor, 1a(PTF1A) | ENSG00000168267 | PTF1A | DOWN |
| kinesin family member 5C(KIF5C) | ENSG00000168280 | KIF5C | UP |
| coiled-coil domain containing 110(CCDC110) | ENSG00000168491 | CCDC110 | DOWN |
| neuron specific gene family member 1(NSG1) | ENSG00000168824 | NSG1 | UP |
| chymotrypsinogen B1(CTRB1) | ENSG00000168925 | CTRB1 | DOWN |
| chymotrypsinogen B2(CTRB2) | ENSG00000168928 | CTRB2 | DOWN |
| actin filament associated protein 1 like 2(AFAP1L2) | ENSG00000169129 | AFAP1L2 | DOWN |
| RAB3B, member RAS oncogene family(RAB3B) | ENSG00000169213 | RAB3B | UP |
| C-X-C motif chemokine ligand 11(CXCL11) | ENSG00000169248 | CXCL11 | DOWN |
| G protein regulated inducer of neurite outgrowth 1(GPRIN1) | ENSG00000169258 | GPRIN1 | UP |
| glycoprotein 2(GP2) | ENSG00000169347 | GP2 | DOWN |
| gap junction protein beta 1(GJB1) | ENSG00000169562 | GJB1 | DOWN |
| metallothionein 1E(MT1E) | ENSG00000169715 | MT1E | DOWN |
| G protein pathway suppressor 1(GPS1) | ENSG00000169727 | GPS1 | DOWN |
| neuregulin 4(NRG4) | ENSG00000169752 | NRG4 | DOWN |
| neuroligin 1(NLGN1) | ENSG00000169760 | NLGN1 | UP |
| one cut homeobox 1(ONECUT1) | ENSG00000169856 | ONECUT1 | DOWN |
| RALBP1 associated Eps domain containing 2(REPS2) | ENSG00000169891 | REPS2 | DOWN |
| intermediate filament family orphan 2(IFFO2) | ENSG00000169991 | IFFO2 | DOWN |
| ring finger protein 150(RNF150) | ENSG00000170153 | RNF150 | UP |
| fatty acid binding protein 4(FABP4) | ENSG00000170323 | FABP4 | DOWN |
| ring finger protein 34(RNF34) | ENSG00000170633 | RNF34 | UP |
| carboxyl ester lipase pseudogene(CELP) | ENSG00000170827 | CELP | DOWN |
| carboxyl ester lipase(CEL) | ENSG00000170835 | CEL | DOWN |
| phospholipase A2 group IB(PLA2G1B) | ENSG00000170890 | PLA2G1B | DOWN |
| protein kinase (cAMP-dependent, catalytic) inhibitor alpha(PKIA) | ENSG00000171033 | PKIA | UP |
| protein kinase C epsilon(PRKCE) | ENSG00000171132 | PRKCE | UP |
| neuropilin and tolloid like 2(NETO2) | ENSG00000171208 | NETO2 | UP |
| leucine rich alpha-2-glycoprotein 1(LRG1) | ENSG00000171236 | LRG1 | DOWN |
| cyclin dependent kinase 5 regulatory subunit 2(CDK5R2) | ENSG00000171450 | CDK5R2 | UP |
| HOP homeobox(HOPX) | ENSG00000171476 | HOPX | UP |
| prostaglandin E receptor 4(PTGER4) | ENSG00000171522 | PTGER4 | DOWN |
| microtubule associated protein 6(MAP6) | ENSG00000171533 | MAP6 | UP |
| glycine amidinotransferase(GATM) | ENSG00000171766 | GATM | DOWN |
| zinc finger protein 540(ZNF540) | ENSG00000171817 | ZNF540 | UP |
| secretogranin II(SCG2) | ENSG00000171951 | SCG2 | UP |
| zinc finger protein 57(ZNF57) | ENSG00000171970 | ZNF57 | DOWN |
| regenerating family member 3 alpha(REG3A) | ENSG00000172016 | REG3A | DOWN |
| regenerating family member 1 beta(REG1B) | ENSG00000172023 | REG1B | DOWN |
| cytochrome c, somatic(CYCS) | ENSG00000172115 | CYCS | UP |
| syntrophin beta 1(SNTB1) | ENSG00000172164 | SNTB1 | DOWN |
| bisphosphoglycerate mutase(BPGM) | ENSG00000172331 | BPGM | UP |
| POP7 homolog, ribonuclease P/MRP subunit(POP7) | ENSG00000172336 | POP7 | UP |
| cold shock domain containing C2(CSDC2) | ENSG00000172346 | CSDC2 | DOWN |
| regulator of calcineurin 2(RCAN2) | ENSG00000172348 | RCAN2 | UP |
| transcription elongation factor A like 1(TCEAL1) | ENSG00000172465 | TCEAL1 | UP |
| carboxylesterase 4A(CES4A) | ENSG00000172824 | CES4A | UP |
| ras homolog family member D(RHOD) | ENSG00000173156 | RHOD | DOWN |
| complement C1q A chain(C1QA) | ENSG00000173372 | C1QA | UP |
| serum amyloid A1(SAA1) | ENSG00000173432 | SAA1 | DOWN |
| protein phosphatase 1 regulatory inhibitor subunit 14B(PPP1R14B) | ENSG00000173457 | PPP1R14B | DOWN |
| anterior gradient 3, protein disulphide isomerase family member(AGR3) | ENSG00000173467 | AGR3 | DOWN |
| FK506 binding protein 2(FKBP2) | ENSG00000173486 | FKBP2 | UP |
| adhesion G protein-coupled receptor G2(ADGRG2) | ENSG00000173698 | ADGRG2 | UP |
| mucin 13, cell surface associated(MUC13) | ENSG00000173702 | MUC13 | UP |
| potassium voltage-gated channel subfamily H member 6(KCNH6) | ENSG00000173826 | KCNH6 | UP |
| chromosome 12 open reading frame 76(C12orf76) | ENSG00000174456 | C12orf76 | UP |
| chemerin chemokine-like receptor 1(CMKLR1) | ENSG00000174600 | CMKLR1 | DOWN |
| BR serine/threonine kinase 2(BRSK2) | ENSG00000174672 | BRSK2 | DOWN |
| leptin(LEP) | ENSG00000174697 | LEP | DOWN |
| cornichon family AMPA receptor auxiliary protein 2(CNIH2) | ENSG00000174871 | CNIH2 | UP |
| amylase, alpha 1B (salivary)(AMY1B) | ENSG00000174876 | AMY1B | DOWN |
| aspartate beta-hydroxylase domain containing 1(ASPHD1) | ENSG00000174939 | ASPHD1 | UP |
| zymogen granule protein 16(ZG16) | ENSG00000174992 | ZG16 | DOWN |
| desmin(DES) | ENSG00000175084 | DES | DOWN |
| family with sequence similarity 131 member A(FAM131A) | ENSG00000175182 | FAM131A | UP |
| phytanoyl-CoA dioxygenase domain containing 1(PHYHD1) | ENSG00000175287 | PHYHD1 | DOWN |
| proprotein convertase subtilisin/kexin type 1(PCSK1) | ENSG00000175426 | PCSK1 | UP |
| nuclear protein 1, transcriptional regulator(NUPR1) | ENSG00000176046 | NUPR1 | DOWN |
| janus kinase and microtubule interacting protein 2(JAKMIP2) | ENSG00000176049 | JAKMIP2 | UP |
| zinc finger and SCAN domain containing 2(ZSCAN2) | ENSG00000176371 | ZSCAN2 | UP |
| EP300 interacting inhibitor of differentiation 2B(EID2B) | ENSG00000176401 | EID2B | UP |
| regulating synaptic membrane exocytosis 2(RIMS2) | ENSG00000176406 | RIMS2 | UP |
| cyclin dependent kinase 5 regulatory subunit 1(CDK5R1) | ENSG00000176749 | CDK5R1 | UP |
| iroquois homeobox 5(IRX5) | ENSG00000176842 | IRX5 | DOWN |
| peroxisomal membrane protein 2(PXMP2) | ENSG00000176894 | PXMP2 | DOWN |
| paraneoplastic Ma antigen 1(PNMA1) | ENSG00000176903 | PNMA1 | UP |
| chromosome 8 open reading frame 4(C8orf4) | ENSG00000176907 | C8orf4 | DOWN |
| SIX homeobox 5(SIX5) | ENSG00000177045 | SIX5 | DOWN |
| anoctamin 6(ANO6) | ENSG00000177119 | ANO6 | DOWN |
| TGFB induced factor homeobox 1(TGIF1) | ENSG00000177426 | TGIF1 | DOWN |
| nucleosome assembly protein 1 like 5(NAP1L5) | ENSG00000177432 | NAP1L5 | UP |
| NIM1 serine/threonine protein kinase(NIM1K) | ENSG00000177453 | NIM1K | UP |
| ST8 alpha-N-acetyl-neuraminide alpha-2,8-sialyltransferase 3(ST8SIA3) | ENSG00000177511 | ST8SIA3 | UP |
| solute carrier family 25 member 22(SLC25A22) | ENSG00000177542 | SLC25A22 | DOWN |
| calcium release activated channel regulator 2B(CRACR2B) | ENSG00000177685 | CRACR2B | DOWN |
| coiled-coil domain containing 184(CCDC184) | ENSG00000177875 | CCDC184 | UP |
| plectin(PLEC) | ENSG00000178209 | PLEC | DOWN |
| COX14, cytochrome c oxidase assembly factor(COX14) | ENSG00000178449 | COX14 | UP |
| prolyl 4-hydroxylase, transmembrane(P4HTM) | ENSG00000178467 | P4HTM | UP |
| cortexin 1(CTXN1) | ENSG00000178531 | CTXN1 | UP |
| carbonic anhydrase 8(CA8) | ENSG00000178538 | CA8 | UP |
| cysteine and serine rich nuclear protein 3(CSRNP3) | ENSG00000178662 | CSRNP3 | UP |
| syntaxin 19(STX19) | ENSG00000178750 | STX19 | DOWN |
| regulatory subunit of type II PKA R-subunit (RIIa) domain containing 1(RIIAD1) | ENSG00000178796 | RIIAD1 | UP |
| 5-oxoprolinase (ATP-hydrolysing)(OPLAH) | ENSG00000178814 | OPLAH | DOWN |
| transmembrane protein 52(TMEM52) | ENSG00000178821 | TMEM52 | DOWN |
| ring finger protein 186(RNF186) | ENSG00000178828 | RNF186 | DOWN |
| family with sequence similarity 133 member A(FAM133A) | ENSG00000179083 | FAM133A | UP |
| transmembrane protein 151A(TMEM151A) | ENSG00000179292 | TMEM151A | UP |
| AT-rich interaction domain 3B(ARID3B) | ENSG00000179361 | ARID3B | DOWN |
| ADP ribosylation factor like GTPase 14(ARL14) | ENSG00000179674 | ARL14 | DOWN |
| syncollin(SYCN) | ENSG00000179751 | SYCN | DOWN |
| pipecolic acid and sarcosine oxidase(PIPOX) | ENSG00000179761 | PIPOX | UP |
| Cbp/p300 interacting transactivator with Glu/Asp rich carboxy-terminal domain 4(CITED4) | ENSG00000179862 | CITED4 | DOWN |
| transmembrane protein 86B(TMEM86B) | ENSG00000180089 | TMEM86B | UP |
| growth arrest specific 1(GAS1) | ENSG00000180447 | GAS1 | DOWN |
| ring finger protein 41(RNF41) | ENSG00000181852 | RNF41 | UP |
| paraneoplastic Ma antigen family like 1(PNMAL1) | ENSG00000182013 | PNMAL1 | UP |
| MGAT4 family member C(MGAT4C) | ENSG00000182050 | MGAT4C | UP |
| transmembrane protein 30B(TMEM30B) | ENSG00000182107 | TMEM30B | DOWN |
| repulsive guidance molecule family member a(RGMA) | ENSG00000182175 | RGMA | DOWN |
| EPH receptor B3(EPHB3) | ENSG00000182580 | EPHB3 | DOWN |
| annexin A2(ANXA2) | ENSG00000182718 | ANXA2 | DOWN |
| glutathione S-transferase alpha 5(GSTA5) | ENSG00000182793 | GSTA5 | DOWN |
| regulator of G-protein signaling 7(RGS7) | ENSG00000182901 | RGS7 | UP |
| chromosome 12 open reading frame 60(C12orf60) | ENSG00000182993 | C12orf60 | UP |
| pyrroline-5-carboxylate reductase 1(PYCR1) | ENSG00000183010 | PYCR1 | DOWN |
| Purkinje cell protein 4(PCP4) | ENSG00000183036 | PCP4 | UP |
| NK2 homeobox 5(NKX2-5) | ENSG00000183072 | NKX2-5 | DOWN |
| G protein-coupled receptor 19(GPR19) | ENSG00000183150 | GPR19 | UP |
| RAB interacting factor(RABIF) | ENSG00000183155 | RABIF | DOWN |
| proline rich 14 like(PRR14L) | ENSG00000183530 | PRR14L | DOWN |
| Aly/REF export factor(ALYREF) | ENSG00000183684 | ALYREF | DOWN |
| family with sequence similarity 3 member B(FAM3B) | ENSG00000183844 | FAM3B | DOWN |
| transmembrane protease, serine 2(TMPRSS2) | ENSG00000184012 | TMPRSS2 | DOWN |
| out at first homolog(OAF) | ENSG00000184232 | OAF | DOWN |
| potassium two pore domain channel subfamily K member 12(KCNK12) | ENSG00000184261 | KCNK12 | UP |
| tumor-associated calcium signal transducer 2(TACSTD2) | ENSG00000184292 | TACSTD2 | DOWN |
| cell cycle exit and neuronal differentiation 1(CEND1) | ENSG00000184524 | CEND1 | DOWN |
| histone cluster 2 H2B family member e(HIST2H2BE) | ENSG00000184678 | HIST2H2BE | UP |
| transmembrane protein 186(TMEM186) | ENSG00000184857 | TMEM186 | DOWN |
| transcription elongation factor A like 2(TCEAL2) | ENSG00000184905 | TCEAL2 | UP |
| aquaporin 12A(AQP12A) | ENSG00000184945 | AQP12A | DOWN |
| mucin 6, oligomeric mucus/gel-forming(MUC6) | ENSG00000184956 | MUC6 | DOWN |
| regulatory factor X6(RFX6) | ENSG00000185002 | RFX6 | UP |
| solute carrier family 24 member 3(SLC24A3) | ENSG00000185052 | SLC24A3 | UP |
| fibronectin leucine rich transmembrane protein 2(FLRT2) | ENSG00000185070 | FLRT2 | DOWN |
| aquaporin 12B(AQP12B) | ENSG00000185176 | AQP12B | DOWN |
| TNF alpha induced protein 2(TNFAIP2) | ENSG00000185215 | TNFAIP2 | DOWN |
| mucin 1, cell surface associated(MUC1) | ENSG00000185499 | MUC1 | DOWN |
| family with sequence similarity 131 member C(FAM131C) | ENSG00000185519 | FAM131C | UP |
| delta like non-canonical Notch ligand 1(DLK1) | ENSG00000185559 | DLK1 | DOWN |
| protein disulfide isomerase family A member 2(PDIA2) | ENSG00000185615 | PDIA2 | DOWN |
| PBX homeobox 1(PBX1) | ENSG00000185630 | PBX1 | DOWN |
| ZFP36 ring finger protein like 1(ZFP36L1) | ENSG00000185650 | ZFP36L1 | DOWN |
| nucleosome assembly protein 1 like 3(NAP1L3) | ENSG00000186310 | NAP1L3 | UP |
| beta-secretase 1(BACE1) | ENSG00000186318 | BACE1 | DOWN |
| nucleosome assembly protein 1 like 2(NAP1L2) | ENSG00000186462 | NAP1L2 | UP |
| G protein subunit gamma 2(GNG2) | ENSG00000186469 | GNG2 | UP |
| piccolo presynaptic cytomatrix protein(PCLO) | ENSG00000186472 | PCLO | UP |
| insulin induced gene 1(INSIG1) | ENSG00000186480 | INSIG1 | DOWN |
| proline rich 5(PRR5) | ENSG00000186654 | PRR5 | DOWN |
| transmembrane protein 17(TMEM17) | ENSG00000186889 | TMEM17 | UP |
| pancreatic lipase related protein 1(PNLIPRP1) | ENSG00000187021 | PNLIPRP1 | DOWN |
| TSPY like 4(TSPYL4) | ENSG00000187189 | TSPYL4 | UP |
| metallothionein 1X(MT1X) | ENSG00000187193 | MT1X | DOWN |
| cell death inducing DFFA like effector c(CIDEC) | ENSG00000187288 | CIDEC | DOWN |
| ISG15 ubiquitin-like modifier(ISG15) | ENSG00000187608 | ISG15 | UP |
| alcohol dehydrogenase 1A (class I), alpha polypeptide(ADH1A) | ENSG00000187758 | ADH1A | DOWN |
| zinc finger protein 70(ZNF70) | ENSG00000187792 | ZNF70 | DOWN |
| delta/notch like EGF repeat containing(DNER) | ENSG00000187957 | DNER | UP |
| smoothelin like 2(SMTNL2) | ENSG00000188176 | SMTNL2 | DOWN |
| serpin family A member 5(SERPINA5) | ENSG00000188488 | SERPINA5 | DOWN |
| BCL2 like 15(BCL2L15) | ENSG00000188761 | BCL2L15 | DOWN |
| ribosomal protein L14(RPL14) | ENSG00000188846 | RPL14 | DOWN |
| apolipoprotein D(APOD) | ENSG00000189058 | APOD | DOWN |
| family with sequence similarity 150 member B(FAM150B) | ENSG00000189292 | FAM150B | DOWN |
| family with sequence similarity 179 member A(FAM179A) | ENSG00000189350 | FAM179A | UP |
| C-X-C motif chemokine ligand 17(CXCL17) | ENSG00000189377 | CXCL17 | DOWN |
| myelin transcription factor 1(MYT1) | ENSG00000196132 | MYT1 | UP |
| serpin family A member 3(SERPINA3) | ENSG00000196136 | SERPINA3 | DOWN |
| transmembrane protein 63A(TMEM63A) | ENSG00000196187 | TMEM63A | DOWN |
| copine 4(CPNE4) | ENSG00000196353 | CPNE4 | UP |
| WD repeat domain 5(WDR5) | ENSG00000196363 | WDR5 | DOWN |
| serine palmitoyltransferase small subunit B(SPTSSB) | ENSG00000196542 | SPTSSB | UP |
| calcium voltage-gated channel subunit alpha1 H(CACNA1H) | ENSG00000196557 | CACNA1H | UP |
| ERI1 exoribonuclease family member 2(ERI2) | ENSG00000196678 | ERI2 | DOWN |
| colipase like 2(CLPSL2) | ENSG00000196748 | CLPSL2 | DOWN |
| multivesicular body subunit 12B(MVB12B) | ENSG00000196814 | MVB12B | UP |
| annexin A4(ANXA4) | ENSG00000196975 | ANXA4 | DOWN |
| solute carrier family 6 member 17(SLC6A17) | ENSG00000197106 | SLC6A17 | UP |
| piwi like RNA-mediated gene silencing 2(PIWIL2) | ENSG00000197181 | PIWIL2 | DOWN |
| oxoglutarate dehydrogenase-like(OGDHL) | ENSG00000197444 | OGDHL | UP |
| stathmin 3(STMN3) | ENSG00000197457 | STMN3 | UP |
| solute carrier family 2 member 10(SLC2A10) | ENSG00000197496 | SLC2A10 | DOWN |
| potassium calcium-activated channel subfamily M regulatory beta subunit 2(KCNMB2) | ENSG00000197584 | KCNMB2 | UP |
| ectonucleotide pyrophosphatase/phosphodiesterase 1(ENPP1) | ENSG00000197594 | ENPP1 | DOWN |
| parvin alpha(PARVA) | ENSG00000197702 | PARVA | DOWN |
| PHD finger protein 2(PHF2) | ENSG00000197724 | PHF2 | DOWN |
| complement factor D(CFD) | ENSG00000197766 | CFD | DOWN |
| TEA domain transcription factor 4(TEAD4) | ENSG00000197905 | TEAD4 | DOWN |
| FLVCR1 antisense RNA 1 (head to head)(FLVCR1-AS1) | ENSG00000198468 | FLVCR1-AS1 | UP |
| delta like canonical Notch ligand 1(DLL1) | ENSG00000198719 | DLL1 | DOWN |
| protein phosphatase 1 regulatory inhibitor subunit 14C(PPP1R14C) | ENSG00000198729 | PPP1R14C | UP |
| SPARC related modular calcium binding 1(SMOC1) | ENSG00000198732 | SMOC1 | UP |
| methionine sulfoxide reductase B1(MSRB1) | ENSG00000198736 | MSRB1 | DOWN |
| thymocyte selection associated high mobility group box(TOX) | ENSG00000198846 | TOX | UP |
| dystrophin(DMD) | ENSG00000198947 | DMD | DOWN |
| small nucleolar RNA, C/D box 104(SNORD104) | ENSG00000199753 | SNORD104 | DOWN |
| sterile alpha motif domain containing 5(SAMD5) | ENSG00000203727 | SAMD5 | UP |
| ripply transcriptional repressor 2(RIPPLY2) | ENSG00000203877 | RIPPLY2 | UP |
| transcription elongation factor A3(TCEA3) | ENSG00000204219 | TCEA3 | DOWN |
| collagen type XV alpha 1 chain(COL15A1) | ENSG00000204291 | COL15A1 | DOWN |
| chorionic somatomammotropin hormone like 1(CSHL1) | ENSG00000204414 | CSHL1 | DOWN |
| psoriasis susceptibility 1 candidate 3 (non-protein coding)(PSORS1C3) | ENSG00000204528 | PSORS1C3 | DOWN |
| aspartate dehydrogenase domain containing(ASPDH) | ENSG00000204653 | ASPDH | UP |
| protease, serine 1(PRSS1) | ENSG00000204983 | PRSS1 | DOWN |
| leucine rich repeat containing G protein-coupled receptor 4(LGR4) | ENSG00000205213 | LGR4 | DOWN |
| metallothionein 1H(MT1H) | ENSG00000205358 | MT1H | DOWN |
| metallothionein 1A(MT1A) | ENSG00000205362 | MT1A | DOWN |
| metallothionein 1M(MT1M) | ENSG00000205364 | MT1M | DOWN |
| DnaJ heat shock protein family (Hsp40) member C19(DNAJC19) | ENSG00000205981 | DNAJC19 | UP |
| microRNA 328(MIR328) | ENSG00000207948 | MIR328 | DOWN |
| myeloid/lymphoid or mixed-lineage leukemia; translocated to, 11(MLLT11) | ENSG00000213190 | MLLT11 | UP |
| keratin 222(KRT222) | ENSG00000213424 | KRT222 | UP |
| ATPase H+ transporting V1 subunit G2(ATP6V1G2) | ENSG00000213760 | ATP6V1G2 | UP |
| deoxyribonuclease 1(DNASE1) | ENSG00000213918 | DNASE1 | DOWN |
| integrin subunit alpha 1(ITGA1) | ENSG00000213949 | ITGA1 | UP |
| urothelial cancer associated 1 (non-protein coding)(UCA1) | ENSG00000214049 | UCA1 | DOWN |
| chymotrypsin like elastase family member 2B(CELA2B) | ENSG00000215704 | CELA2B | DOWN |
| long intergenic non-protein coding RNA 339(LINC00339) | ENSG00000218510 | LINC00339 | DOWN |
| chymotrypsin like elastase family member 3B(CELA3B) | ENSG00000219073 | CELA3B | DOWN |
| creatine kinase, mitochondrial 1A(CKMT1A) | ENSG00000223572 | CKMT1A | UP |
| stathmin domain containing 1(STMND1) | ENSG00000230873 | STMND1 | DOWN |
| creatine kinase, mitochondrial 1B(CKMT1B) | ENSG00000237289 | CKMT1B | UP |
| DiGeorge syndrome critical region gene 5 (non-protein coding)(DGCR5) | ENSG00000237517 | DGCR5 | DOWN |
| kelch like family member 41(KLHL41) | ENSG00000239474 | KLHL41 | UP |
| amylase, alpha 2B (pancreatic)(AMY2B) | ENSG00000240038 | AMY2B | DOWN |
| aquaporin 1 (Colton blood group)(AQP1) | ENSG00000240583 | AQP1 | DOWN |
| paraneoplastic Ma antigen 2(PNMA2) | ENSG00000240694 | PNMA2 | UP |
| 4-hydroxy-2-oxoglutarate aldolase 1(HOGA1) | ENSG00000241935 | HOGA1 | DOWN |
| mitochondrial fission process 1(MTFP1) | ENSG00000242114 | MTFP1 | UP |
| Rho GDP dissociation inhibitor gamma(ARHGDIG) | ENSG00000242173 | ARHGDIG | DOWN |
| paternally expressed 10(PEG10) | ENSG00000242265 | PEG10 | UP |
| retrotransposon gag domain containing 4(RGAG4) | ENSG00000242732 | RGAG4 | UP |
| amylase, alpha 2A (pancreatic)(AMY2A) | ENSG00000243480 | AMY2A | DOWN |
| NME/NM23 nucleoside diphosphate kinase 2(NME2) | ENSG00000243678 | NME2 | DOWN |
| left-right determination factor 1(LEFTY1) | ENSG00000243709 | LEFTY1 | DOWN |
| glutathione S-transferase alpha 1(GSTA1) | ENSG00000243955 | GSTA1 | DOWN |
| glutathione S-transferase alpha 2(GSTA2) | ENSG00000244067 | GSTA2 | DOWN |
| CCAAT/enhancer binding protein alpha(CEBPA) | ENSG00000245848 | CEBPA | DOWN |
| alcohol dehydrogenase 1C (class I), gamma polypeptide(ADH1C) | ENSG00000248144 | ADH1C | DOWN |
| long intergenic non-protein coding RNA 1091(LINC01091) | ENSG00000249464 | LINC01091 | UP |
| G protein-coupled receptor 162(GPR162) | ENSG00000250510 | GPR162 | UP |
| RNA, Ro-associated Y4(RNY4) | ENSG00000252316 | RNY4 | DOWN |
| insulin(INS) | ENSG00000254647 | INS | DOWN |
| pepsinogen 5, group I (pepsinogen A)(PGA5) | ENSG00000256713 | PGA5 | DOWN |
| tubulin beta 3 class III(TUBB3) | ENSG00000258947 | TUBB3 | UP |
| ZFP41 zinc finger protein(ZFP41) | ENSG00000264668 | ZFP41 | DOWN |
| pancreatic lipase related protein 2 (gene/pseudogene)(PNLIPRP2) | ENSG00000266200 | PNLIPRP2 | DOWN |
| growth differentiation factor 10(GDF10) | ENSG00000266524 | GDF10 | DOWN |
| secretoglobin family 1B member 2, pseudogene(SCGB1B2P) | ENSG00000268751 | SCGB1B2P | DOWN |
| long intergenic non-protein coding RNA 936(LINC00936) | ENSG00000271614 | LINC00936 | UP |
| uncharacterized LOC389332(LOC389332) | ENSG00000271824 | LOC389332 | UP |
| CD24 molecule(CD24) | ENSG00000272398 | CD24 | DOWN |
| histone cluster 1 H4 family member k(HIST1H4K) | ENSG00000273542 | HIST1H4K | UP |
| HNF1 homeobox B(HNF1B) | ENSG00000275410 | HNF1B | DOWN |
| protease, serine 2(PRSS2) | ENSG00000275896 | PRSS2 | DOWN |
| olfactory receptor family 5 subfamily L member 1 (gene/pseudogene)(OR5L1) | ENSG00000279395 | OR5L1 | DOWN |
| cadherin EGF LAG seven-pass G-type receptor 3(CELSR3) |  | CELSR3 | UP |
| membrane associated ring-CH-type finger 4(MARCH4) |  | MARCH4 | UP |
| aryl hydrocarbon receptor nuclear translocator 2(ARNT2) |  | ARNT2 | UP |
| transmembrane and coiled-coil domain family 3(TMCC3) |  | TMCC3 | UP |
| septin 5(SEPT5) |  | SEPT5 | UP |
| chromosome 15 open reading frame 48(C15orf48) |  | C15orf48 | UP |
| stress responsive DNAJB4 interacting membrane protein 1(SDIM1) |  | SDIM1 | UP |
| protease, serine 3 pseudogene 2(PRSS3P2) |  | PRSS3P2 | DOWN |
| NPHS1, nephrin(NPHS1) |  | NPHS1 | DOWN |
| MGC72080 pseudogene(MGC72080) |  | MGC72080 | DOWN |
| hemoglobin subunit beta pseudogene 1(HBBP1) |  | HBBP1 | DOWN |
| programmed cell death 4 (neoplastic transformation inhibitor)(PDCD4) |  | PDCD4 | DOWN |
| thyroid hormone receptor interactor 6(TRIP6) |  | TRIP6 | DOWN |
| uncharacterized LOC285147(LOC285147) |  | LOC285147 | DOWN |
| pleckstrin homology like domain family B member 1(PHLDB1) |  | PHLDB1 | DOWN |
| semaphorin 3B(SEMA3B) |  | SEMA3B | DOWN |
| annexin A2 pseudogene 1(ANXA2P1) |  | ANXA2P1 | DOWN |
| GRINL1A complex locus 1(GCOM1) |  | GCOM1 | DOWN |

| **Table S8: Differentially Expressed Genes in SCLC** | | | |
| --- | --- | --- | --- |
|  |  |  |  |
| **Name** | **ENSEMBL_ID** | **Gene Symbol** | **Expression** |
| FGR proto-oncogene, Src family tyrosine kinase(FGR) | ENSG00000000938 | FGR | DOWN |
| Y-box binding protein 2(YBX2) | ENSG00000006047 | YBX2 | UP |
| C-X3-C motif chemokine ligand 1(CX3CL1) | ENSG00000006210 | CX3CL1 | DOWN |
| aldehyde dehydrogenase 3 family member B1(ALDH3B1) | ENSG00000006534 | ALDH3B1 | DOWN |
| USH1 protein network component harmonin(USH1C) | ENSG00000006611 | USH1C | UP |
| prominin 1(PROM1) | ENSG00000007062 | PROM1 | UP |
| calcium voltage-gated channel auxiliary subunit alpha2delta 2(CACNA2D2) | ENSG00000007402 | CACNA2D2 | DOWN |
| E2F transcription factor 2(E2F2) | ENSG00000007968 | E2F2 | UP |
| synapsin I(SYN1) | ENSG00000008056 | SYN1 | UP |
| interleukin 32(IL32) | ENSG00000008517 | IL32 | DOWN |
| CD9 molecule(CD9) | ENSG00000010278 | CD9 | DOWN |
| semaphorin 3G(SEMA3G) | ENSG00000010319 | SEMA3G | DOWN |
| decorin(DCN) | ENSG00000011465 | DCN | DOWN |
| solute carrier family 7 member 14(SLC7A14) | ENSG00000013293 | SLC7A14 | UP |
| solute carrier family 11 member 1(SLC11A1) | ENSG00000018280 | SLC11A1 | DOWN |
| V-set and immunoglobulin domain containing 2(VSIG2) | ENSG00000019102 | VSIG2 | DOWN |
| macrophage receptor with collagenous structure(MARCO) | ENSG00000019169 | MARCO | DOWN |
| synaptotagmin 13(SYT13) | ENSG00000019505 | SYT13 | UP |
| CD74 molecule(CD74) | ENSG00000019582 | CD74 | DOWN |
| serpin family B member 1(SERPINB1) | ENSG00000021355 | SERPINB1 | DOWN |
| four and a half LIM domains 1(FHL1) | ENSG00000022267 | FHL1 | DOWN |
| EH domain containing 2(EHD2) | ENSG00000024422 | EHD2 | DOWN |
| DEP domain containing 1(DEPDC1) | ENSG00000024526 | DEPDC1 | UP |
| interferon gamma receptor 1(IFNGR1) | ENSG00000027697 | IFNGR1 | DOWN |
| Rho GTPase activating protein 31(ARHGAP31) | ENSG00000031081 | ARHGAP31 | DOWN |
| leucine rich repeat containing 7(LRRC7) | ENSG00000033122 | LRRC7 | UP |
| vinculin(VCL) | ENSG00000035403 | VCL | DOWN |
| death associated protein kinase 2(DAPK2) | ENSG00000035664 | DAPK2 | DOWN |
| fms related tyrosine kinase 4(FLT4) | ENSG00000037280 | FLT4 | DOWN |
| adrenoceptor beta 1(ADRB1) | ENSG00000043591 | ADRB1 | DOWN |
| Rho GTPase activating protein 6(ARHGAP6) | ENSG00000047648 | ARHGAP6 | DOWN |
| ROS proto-oncogene 1, receptor tyrosine kinase(ROS1) | ENSG00000047936 | ROS1 | DOWN |
| elastin(ELN) | ENSG00000049540 | ELN | DOWN |
| DNA polymerase theta(POLQ) | ENSG00000051341 | POLQ | UP |
| protease, serine 8(PRSS8) | ENSG00000052344 | PRSS8 | DOWN |
| laminin subunit alpha 3(LAMA3) | ENSG00000053747 | LAMA3 | DOWN |
| protein tyrosine phosphatase, receptor type N(PTPRN) | ENSG00000054356 | PTPRN | UP |
| PHD finger protein 21B(PHF21B) | ENSG00000056487 | PHF21B | UP |
| calcium/calmodulin dependent protein kinase II beta(CAMK2B) | ENSG00000058404 | CAMK2B | UP |
| RIMS binding protein 2(RIMBP2) | ENSG00000060709 | RIMBP2 | UP |
| seizure related 6 homolog(SEZ6) | ENSG00000063015 | SEZ6 | UP |
| LIM and calponin homology domains 1(LIMCH1) | ENSG00000064042 | LIMCH1 | DOWN |
| calponin 2(CNN2) | ENSG00000064666 | CNN2 | DOWN |
| calcitonin receptor like receptor(CALCRL) | ENSG00000064989 | CALCRL | DOWN |
| synaptosome associated protein 91(SNAP91) | ENSG00000065609 | SNAP91 | UP |
| tyrosine kinase with immunoglobulin like and EGF like domains 1(TIE1) | ENSG00000066056 | TIE1 | DOWN |
| claudin 18(CLDN18) | ENSG00000066405 | CLDN18 | DOWN |
| Kruppel like factor 6(KLF6) | ENSG00000067082 | KLF6 | DOWN |
| PDZ domain containing 4(PDZD4) | ENSG00000067840 | PDZD4 | UP |
| ATPase plasma membrane Ca2+ transporting 3(ATP2B3) | ENSG00000067842 | ATP2B3 | UP |
| hyaluronoglucosaminidase 2(HYAL2) | ENSG00000068001 | HYAL2 | DOWN |
| profilin 2(PFN2) | ENSG00000070087 | PFN2 | UP |
| follistatin like 3(FSTL3) | ENSG00000070404 | FSTL3 | DOWN |
| LIM and cysteine rich domains 1(LMCD1) | ENSG00000071282 | LMCD1 | DOWN |
| cytochrome b reductase 1(CYBRD1) | ENSG00000071967 | CYBRD1 | DOWN |
| LIM zinc finger domain containing 2(LIMS2) | ENSG00000072163 | LIMS2 | DOWN |
| prostaglandin-endoperoxide synthase 2(PTGS2) | ENSG00000073756 | PTGS2 | DOWN |
| cadherin related family member 2(CDHR2) | ENSG00000074276 | CDHR2 | UP |
| netrin 4(NTN4) | ENSG00000074527 | NTN4 | DOWN |
| scavenger receptor class F member 1(SCARF1) | ENSG00000074660 | SCARF1 | DOWN |
| G2 and S-phase expressed 1(GTSE1) | ENSG00000075218 | GTSE1 | UP |
| RNA binding motif single stranded interacting protein 2(RBMS2) | ENSG00000076067 | RBMS2 | DOWN |
| sperm associated antigen 5(SPAG5) | ENSG00000076382 | SPAG5 | UP |
| ubiquitin conjugating enzyme E2 T(UBE2T) | ENSG00000077152 | UBE2T | UP |
| interleukin 4 receptor(IL4R) | ENSG00000077238 | IL4R | DOWN |
| integrin subunit alpha 8(ITGA8) | ENSG00000077943 | ITGA8 | DOWN |
| lysosomal associated membrane protein 3(LAMP3) | ENSG00000078081 | LAMP3 | DOWN |
| adenylate cyclase 2(ADCY2) | ENSG00000078295 | ADCY2 | UP |
| endothelin 1(EDN1) | ENSG00000078401 | EDN1 | DOWN |
| tensin 1(TNS1) | ENSG00000079308 | TNS1 | DOWN |
| protein tyrosine phosphatase, receptor type H(PTPRH) | ENSG00000080031 | PTPRH | UP |
| carboxypeptidase B2(CPB2) | ENSG00000080618 | CPB2 | DOWN |
| cholinergic receptor nicotinic alpha 3 subunit(CHRNA3) | ENSG00000080644 | CHRNA3 | UP |
| C-X-C motif chemokine ligand 2(CXCL2) | ENSG00000081041 | CXCL2 | DOWN |
| opioid receptor kappa 1(OPRK1) | ENSG00000082556 | OPRK1 | UP |
| ficolin 1(FCN1) | ENSG00000085265 | FCN1 | DOWN |
| immunoglobulin superfamily member 9(IGSF9) | ENSG00000085552 | IGSF9 | UP |
| RAD54-like (S. cerevisiae)(RAD54L) | ENSG00000085999 | RAD54L | UP |
| inositol-trisphosphate 3-kinase C(ITPKC) | ENSG00000086544 | ITPKC | DOWN |
| carcinoembryonic antigen related cell adhesion molecule 6(CEACAM6) | ENSG00000086548 | CEACAM6 | DOWN |
| protein phosphatase 1 regulatory subunit 15A(PPP1R15A) | ENSG00000087074 | PPP1R15A | DOWN |
| G protein subunit alpha o1(GNAO1) | ENSG00000087258 | GNAO1 | UP |
| Cas scaffolding protein family member 4(CASS4) | ENSG00000087589 | CASS4 | DOWN |
| baculoviral IAP repeat containing 5(BIRC5) | ENSG00000089685 | BIRC5 | UP |
| biliverdin reductase B(BLVRB) | ENSG00000090013 | BLVRB | DOWN |
| intercellular adhesion molecule 1(ICAM1) | ENSG00000090339 | ICAM1 | DOWN |
| delta like canonical Notch ligand 3(DLL3) | ENSG00000090932 | DLL3 | UP |
| laminin subunit beta 1(LAMB1) | ENSG00000091136 | LAMB1 | DOWN |
| phosphoglycerate dehydrogenase(PHGDH) | ENSG00000092621 | PHGDH | UP |
| unc-13 homolog D(UNC13D) | ENSG00000092929 | UNC13D | DOWN |
| flavin containing monooxygenase 2(FMO2) | ENSG00000094963 | FMO2 | DOWN |
| SH2 domain containing 3C(SH2D3C) | ENSG00000095370 | SH2D3C | DOWN |
| myosin IIIA(MYO3A) | ENSG00000095777 | MYO3A | UP |
| progastricsin(PGC) | ENSG00000096088 | PGC | DOWN |
| sushi domain containing 2(SUSD2) | ENSG00000099994 | SUSD2 | DOWN |
| galectin 2(LGALS2) | ENSG00000100079 | LGALS2 | DOWN |
| TIMP metallopeptidase inhibitor 3(TIMP3) | ENSG00000100234 | TIMP3 | DOWN |
| platelet derived growth factor subunit B(PDGFB) | ENSG00000100311 | PDGFB | DOWN |
| potassium two pore domain channel subfamily K member 10(KCNK10) | ENSG00000100433 | KCNK10 | UP |
| tripartite motif containing 9(TRIM9) | ENSG00000100505 | TRIM9 | UP |
| cyclin dependent kinase inhibitor 3(CDKN3) | ENSG00000100526 | CDKN3 | UP |
| Ras and Rab interactor 3(RIN3) | ENSG00000100599 | RIN3 | DOWN |
| NFKB inhibitor alpha(NFKBIA) | ENSG00000100906 | NFKBIA | DOWN |
| solute carrier organic anion transporter family member 4A1(SLCO4A1) | ENSG00000101187 | SLCO4A1 | DOWN |
| myosin light chain 9(MYL9) | ENSG00000101335 | MYL9 | DOWN |
| CUGBP, Elav-like family member 4(CELF4) | ENSG00000101489 | CELF4 | UP |
| nucleolar protein 4(NOL4) | ENSG00000101746 | NOL4 | UP |
| sushi repeat containing protein, X-linked(SRPX) | ENSG00000101955 | SRPX | DOWN |
| proprotein convertase subtilisin/kexin type 1 inhibitor(PCSK1N) | ENSG00000102109 | PCSK1N | UP |
| regulator of cell cycle(RGCC) | ENSG00000102760 | RGCC | DOWN |
| mesothelin(MSLN) | ENSG00000102854 | MSLN | DOWN |
| plasmolipin(PLLP) | ENSG00000102934 | PLLP | DOWN |
| sphingomyelin phosphodiesterase 3(SMPD3) | ENSG00000103056 | SMPD3 | UP |
| cysteine rich secretory protein LCCL domain containing 2(CRISPLD2) | ENSG00000103196 | CRISPLD2 | DOWN |
| forkhead box F1(FOXF1) | ENSG00000103241 | FOXF1 | DOWN |
| TOX high mobility group box family member 3(TOX3) | ENSG00000103460 | TOX3 | UP |
| aquaporin 9(AQP9) | ENSG00000103569 | AQP9 | DOWN |
| adaptor related protein complex 3 beta 2 subunit(AP3B2) | ENSG00000103723 | AP3B2 | UP |
| cathepsin H(CTSH) | ENSG00000103811 | CTSH | DOWN |
| frizzled class receptor 3(FZD3) | ENSG00000104290 | FZD3 | UP |
| EYA transcriptional coactivator and phosphatase 1(EYA1) | ENSG00000104313 | EYA1 | UP |
| ganglioside induced differentiation associated protein 1(GDAP1) | ENSG00000104381 | GDAP1 | UP |
| anti-silencing function 1B histone chaperone(ASF1B) | ENSG00000105011 | ASF1B | UP |
| periaxin(PRX) | ENSG00000105227 | PRX | DOWN |
| fibronectin type III and SPRY domain containing 1(FSD1) | ENSG00000105255 | FSD1 | UP |
| amyloid beta precursor like protein 1(APLP1) | ENSG00000105290 | APLP1 | UP |
| carcinoembryonic antigen related cell adhesion molecule 4(CEACAM4) | ENSG00000105352 | CEACAM4 | DOWN |
| intercellular adhesion molecule 5(ICAM5) | ENSG00000105376 | ICAM5 | DOWN |
| ATPase Na+/K+ transporting subunit alpha 3(ATP1A3) | ENSG00000105409 | ATP1A3 | UP |
| glutamate ionotropic receptor NMDA type subunit 2D(GRIN2D) | ENSG00000105464 | GRIN2D | UP |
| glutamate ionotropic receptor kainate type subunit 5(GRIK5) | ENSG00000105737 | GRIK5 | UP |
| caveolin 2(CAV2) | ENSG00000105971 | CAV2 | DOWN |
| caveolin 1(CAV1) | ENSG00000105974 | CAV1 | DOWN |
| transferrin receptor 2(TFR2) | ENSG00000106327 | TFR2 | UP |
| serpin family E member 1(SERPINE1) | ENSG00000106366 | SERPINE1 | DOWN |
| aryl hydrocarbon receptor(AHR) | ENSG00000106546 | AHR | DOWN |
| LIM homeobox 2(LHX2) | ENSG00000106689 | LHX2 | UP |
| endoglin(ENG) | ENSG00000106991 | ENG | DOWN |
| SH3 domain containing GRB2 like 2, endophilin A1(SH3GL2) | ENSG00000107295 | SH3GL2 | UP |
| prostaglandin D2 synthase(PTGDS) | ENSG00000107317 | PTGDS | DOWN |
| Ras association domain family member 4(RASSF4) | ENSG00000107551 | RASSF4 | DOWN |
| chromosome 10 open reading frame 54(C10orf54) | ENSG00000107738 | C10orf54 | DOWN |
| mitogen-activated protein kinase kinase kinase 8(MAP3K8) | ENSG00000107968 | MAP3K8 | DOWN |
| RUN domain containing 3A(RUNDC3A) | ENSG00000108309 | RUNDC3A | UP |
| colony stimulating factor 3(CSF3) | ENSG00000108342 | CSF3 | DOWN |
| solute carrier family 6 member 4(SLC6A4) | ENSG00000108576 | SLC6A4 | DOWN |
| peripheral myelin protein 22(PMP22) | ENSG00000109099 | PMP22 | DOWN |
| SH3 domain containing 19(SH3D19) | ENSG00000109686 | SH3D19 | DOWN |
| HGF activator(HGFAC) | ENSG00000109758 | HGFAC | UP |
| zinc finger and BTB domain containing 16(ZBTB16) | ENSG00000109906 | ZBTB16 | DOWN |
| folate receptor 1(FOLR1) | ENSG00000110195 | FOLR1 | DOWN |
| von Willebrand factor(VWF) | ENSG00000110799 | VWF | DOWN |
| bridging integrator 2(BIN2) | ENSG00000110934 | BIN2 | DOWN |
| synaptotagmin 10(SYT10) | ENSG00000110975 | SYT10 | UP |
| aldehyde dehydrogenase 2 family (mitochondrial)(ALDH2) | ENSG00000111275 | ALDH2 | DOWN |
| Rho GDP dissociation inhibitor beta(ARHGDIB) | ENSG00000111348 | ARHGDIB | DOWN |
| neural precursor cell expressed, developmentally down-regulated 9(NEDD9) | ENSG00000111859 | NEDD9 | DOWN |
| SAM and SH3 domain containing 1(SASH1) | ENSG00000111961 | SASH1 | DOWN |
| chloride intracellular channel 5(CLIC5) | ENSG00000112782 | CLIC5 | DOWN |
| heparin binding EGF like growth factor(HBEGF) | ENSG00000113070 | HBEGF | DOWN |
| protocadherin 12(PCDH12) | ENSG00000113555 | PCDH12 | DOWN |
| hyaluronoglucosaminidase 1(HYAL1) | ENSG00000114378 | HYAL1 | DOWN |
| chromosome 3 open reading frame 14(C3orf14) | ENSG00000114405 | C3orf14 | UP |
| vasoactive intestinal peptide receptor 1(VIPR1) | ENSG00000114812 | VIPR1 | DOWN |
| troponin C1, slow skeletal and cardiac type(TNNC1) | ENSG00000114854 | TNNC1 | DOWN |
| spectrin beta, non-erythrocytic 1(SPTBN1) | ENSG00000115306 | SPTBN1 | DOWN |
| acyl-CoA dehydrogenase, long chain(ACADL) | ENSG00000115361 | ACADL | DOWN |
| interleukin 1 receptor like 1(IL1RL1) | ENSG00000115602 | IL1RL1 | DOWN |
| interleukin 18 receptor 1(IL18R1) | ENSG00000115604 | IL18R1 | DOWN |
| endothelial PAS domain protein 1(EPAS1) | ENSG00000116016 | EPAS1 | DOWN |
| opioid receptor delta 1(OPRD1) | ENSG00000116329 | OPRD1 | UP |
| DLG associated protein 3(DLGAP3) | ENSG00000116544 | DLGAP3 | UP |
| cell division cycle 20(CDC20) | ENSG00000117399 | CDC20 | UP |
| coagulation factor III, tissue factor(F3) | ENSG00000117525 | F3 | DOWN |
| NIMA related kinase 2(NEK2) | ENSG00000117650 | NEK2 | UP |
| kinesin family member 14(KIF14) | ENSG00000118193 | KIF14 | UP |
| filamin A interacting protein 1(FILIP1) | ENSG00000118407 | FILIP1 | DOWN |
| TNF alpha induced protein 3(TNFAIP3) | ENSG00000118503 | TNFAIP3 | DOWN |
| serum/glucocorticoid regulated kinase 1(SGK1) | ENSG00000118515 | SGK1 | DOWN |
| connective tissue growth factor(CTGF) | ENSG00000118523 | CTGF | DOWN |
| transcription factor 21(TCF21) | ENSG00000118526 | TCF21 | DOWN |
| Kruppel like factor 9(KLF9) | ENSG00000119138 | KLF9 | DOWN |
| WD repeat domain 34(WDR34) | ENSG00000119333 | WDR34 | UP |
| colony stimulating factor 3 receptor(CSF3R) | ENSG00000119535 | CSF3R | DOWN |
| latent transforming growth factor beta binding protein 2(LTBP2) | ENSG00000119681 | LTBP2 | DOWN |
| helicase, lymphoid-specific(HELLS) | ENSG00000119969 | HELLS | UP |
| dual specificity phosphatase 1(DUSP1) | ENSG00000120129 | DUSP1 | DOWN |
| TEK receptor tyrosine kinase(TEK) | ENSG00000120156 | TEK | DOWN |
| myc target 1(MYCT1) | ENSG00000120279 | MYCT1 | DOWN |
| centromere protein L(CENPL) | ENSG00000120334 | CENPL | UP |
| suppressor of cytokine signaling 2(SOCS2) | ENSG00000120833 | SOCS2 | DOWN |
| T-box 2(TBX2) | ENSG00000121068 | TBX2 | DOWN |
| T-box 4(TBX4) | ENSG00000121075 | TBX4 | DOWN |
| catalase(CAT) | ENSG00000121691 | CAT | DOWN |
| CUB and Sushi multiple domains 2(CSMD2) | ENSG00000121904 | CSMD2 | UP |
| receptor activity modifying protein 3(RAMP3) | ENSG00000122679 | RAMP3 | DOWN |
| surfactant protein A1(SFTPA1) | ENSG00000122852 | SFTPA1 | DOWN |
| neurogenin 3(NEUROG3) | ENSG00000122859 | NEUROG3 | UP |
| serglycin(SRGN) | ENSG00000122862 | SRGN | DOWN |
| cyclin dependent kinase inhibitor 2C(CDKN2C) | ENSG00000123080 | CDKN2C | UP |
| matrix metallopeptidase 19(MMP19) | ENSG00000123342 | MMP19 | DOWN |
| SCL/TAL1 interrupting locus(STIL) | ENSG00000123473 | STIL | UP |
| G0/G1 switch 2(G0S2) | ENSG00000123689 | G0S2 | DOWN |
| complement component 4 binding protein alpha(C4BPA) | ENSG00000123838 | C4BPA | DOWN |
| syndecan 4(SDC4) | ENSG00000124145 | SDC4 | DOWN |
| hypoxia inducible factor 3 alpha subunit(HIF3A) | ENSG00000124440 | HIF3A | DOWN |
| serpin family B member 6(SERPINB6) | ENSG00000124570 | SERPINB6 | DOWN |
| histone cluster 1 H2B family member j(HIST1H2BJ) | ENSG00000124635 | HIST1H2BJ | UP |
| triggering receptor expressed on myeloid cells 1(TREM1) | ENSG00000124731 | TREM1 | DOWN |
| AHNAK nucleoprotein(AHNAK) | ENSG00000124942 | AHNAK | DOWN |
| complement C3(C3) | ENSG00000125730 | C3 | DOWN |
| FosB proto-oncogene, AP-1 transcription factor subunit(FOSB) | ENSG00000125740 | FOSB | DOWN |
| CD93 molecule(CD93) | ENSG00000125810 | CD93 | DOWN |
| bone morphogenetic protein 2(BMP2) | ENSG00000125845 | BMP2 | DOWN |
| leucine rich repeat neuronal 4(LRRN4) | ENSG00000125872 | LRRN4 | DOWN |
| related RAS viral (r-ras) oncogene homolog(RRAS) | ENSG00000126458 | RRAS | DOWN |
| SIX homeobox 1(SIX1) | ENSG00000126778 | SIX1 | UP |
| DLG associated protein 5(DLGAP5) | ENSG00000126787 | DLGAP5 | UP |
| ATPase 13A4(ATP13A4) | ENSG00000127249 | ATP13A4 | DOWN |
| protein tyrosine phosphatase, receptor type B(PTPRB) | ENSG00000127329 | PTPRB | DOWN |
| Kruppel like factor 2(KLF2) | ENSG00000127528 | KLF2 | DOWN |
| F2R like thrombin/trypsin receptor 3(F2RL3) | ENSG00000127533 | F2RL3 | DOWN |
| protein kinase, membrane associated tyrosine/threonine 1(PKMYT1) | ENSG00000127564 | PKMYT1 | UP |
| G protein subunit gamma 11(GNG11) | ENSG00000127920 | GNG11 | DOWN |
| STEAP4 metalloreductase(STEAP4) | ENSG00000127954 | STEAP4 | DOWN |
| ZFP36 ring finger protein(ZFP36) | ENSG00000128016 | ZFP36 | DOWN |
| kinase insert domain receptor(KDR) | ENSG00000128052 | KDR | DOWN |
| CDC42 effector protein 1(CDC42EP1) | ENSG00000128283 | CDC42EP1 | DOWN |
| VGF nerve growth factor inducible(VGF) | ENSG00000128564 | VGF | UP |
| homeobox D10(HOXD10) | ENSG00000128710 | HOXD10 | UP |
| potassium voltage-gated channel subfamily C member 1(KCNC1) | ENSG00000129159 | KCNC1 | UP |
| ribonuclease A family member 1, pancreatic(RNASE1) | ENSG00000129538 | RNASE1 | DOWN |
| dedicator of cytokinesis 6(DOCK6) | ENSG00000130158 | DOCK6 | DOWN |
| kinesin family member 1A(KIF1A) | ENSG00000130294 | KIF1A | UP |
| unc-13 homolog A(UNC13A) | ENSG00000130477 | UNC13A | UP |
| motor neuron and pancreas homeobox 1(MNX1) | ENSG00000130675 | MNX1 | UP |
| spectrin repeat containing nuclear envelope protein 1(SYNE1) | ENSG00000131018 | SYNE1 | DOWN |
| leukocyte immunoglobulin like receptor B2(LILRB2) | ENSG00000131042 | LILRB2 | DOWN |
| complement C1q like 1(C1QL1) | ENSG00000131094 | C1QL1 | UP |
| napsin A aspartic peptidase(NAPSA) | ENSG00000131400 | NAPSA | DOWN |
| amine oxidase, copper containing 3(AOC3) | ENSG00000131471 | AOC3 | DOWN |
| receptor activity modifying protein 2(RAMP2) | ENSG00000131477 | RAMP2 | DOWN |
| peroxisome proliferator activated receptor gamma(PPARG) | ENSG00000132170 | PPARG | DOWN |
| protein tyrosine phosphatase, receptor type E(PTPRE) | ENSG00000132334 | PTPRE | DOWN |
| synaptosome associated protein 25(SNAP25) | ENSG00000132639 | SNAP25 | UP |
| ATPase phospholipid transporting 8A2(ATP8A2) | ENSG00000132932 | ATP8A2 | UP |
| arachidonate 5-lipoxygenase activating protein(ALOX5AP) | ENSG00000132965 | ALOX5AP | DOWN |
| brain expressed X-linked 1(BEX1) | ENSG00000133169 | BEX1 | UP |
| PDZ domain containing 2(PDZD2) | ENSG00000133401 | PDZD2 | DOWN |
| surfactant protein D(SFTPD) | ENSG00000133661 | SFTPD | DOWN |
| phosphatidylethanolamine binding protein 4(PEBP4) | ENSG00000134020 | PEBP4 | DOWN |
| epithelial membrane protein 1(EMP1) | ENSG00000134531 | EMP1 | DOWN |
| cell division cycle associated 8(CDCA8) | ENSG00000134690 | CDCA8 | UP |
| platelet derived growth factor receptor alpha(PDGFRA) | ENSG00000134853 | PDGFRA | DOWN |
| ADAM metallopeptidase with thrombospondin type 1 motif 8(ADAMTS8) | ENSG00000134917 | ADAMTS8 | DOWN |
| transmembrane protein 2(TMEM2) | ENSG00000135048 | TMEM2 | DOWN |
| family with sequence similarity 189 member A2(FAM189A2) | ENSG00000135063 | FAM189A2 | DOWN |
| musashi RNA binding protein 1(MSI1) | ENSG00000135097 | MSI1 | UP |
| deltex E3 ubiquitin ligase 1(DTX1) | ENSG00000135144 | DTX1 | UP |
| CD36 molecule(CD36) | ENSG00000135218 | CD36 | DOWN |
| beta-1,4-N-acetyl-galactosaminyltransferase 1(B4GALNT1) | ENSG00000135454 | B4GALNT1 | UP |
| syntaxin 11(STX11) | ENSG00000135604 | STX11 | DOWN |
| dysferlin(DYSF) | ENSG00000135636 | DYSF | DOWN |
| regulator of G-protein signaling 8(RGS8) | ENSG00000135824 | RGS8 | UP |
| solute carrier family 19 member 3(SLC19A3) | ENSG00000135917 | SLC19A3 | DOWN |
| DNA damage regulated autophagy modulator 1(DRAM1) | ENSG00000136048 | DRAM1 | DOWN |
| sciellin(SCEL) | ENSG00000136155 | SCEL | DOWN |
| integral membrane protein 2B(ITM2B) | ENSG00000136156 | ITM2B | DOWN |
| endothelin receptor type B(EDNRB) | ENSG00000136160 | EDNRB | DOWN |
| interleukin 6(IL6) | ENSG00000136244 | IL6 | DOWN |
| Kruppel like factor 4(KLF4) | ENSG00000136826 | KLF4 | DOWN |
| ectonucleotide pyrophosphatase/phosphodiesterase 2(ENPP2) | ENSG00000136960 | ENPP2 | DOWN |
| interleukin 33(IL33) | ENSG00000137033 | IL33 | DOWN |
| forkhead box F2(FOXF2) | ENSG00000137273 | FOXF2 | DOWN |
| ring finger protein 144B(RNF144B) | ENSG00000137393 | RNF144B | DOWN |
| arrestin beta 1(ARRB1) | ENSG00000137486 | ARRB1 | DOWN |
| leucine rich repeat containing 32(LRRC32) | ENSG00000137507 | LRRC32 | DOWN |
| solute carrier organic anion transporter family member 5A1(SLCO5A1) | ENSG00000137571 | SLCO5A1 | UP |
| transient receptor potential cation channel subfamily C member 6(TRPC6) | ENSG00000137672 | TRPC6 | DOWN |
| Yes associated protein 1(YAP1) | ENSG00000137693 | YAP1 | DOWN |
| thrombospondin 1(THBS1) | ENSG00000137801 | THBS1 | DOWN |
| nucleolar and spindle associated protein 1(NUSAP1) | ENSG00000137804 | NUSAP1 | UP |
| SMAD family member 6(SMAD6) | ENSG00000137834 | SMAD6 | DOWN |
| dual oxidase 1(DUOX1) | ENSG00000137857 | DUOX1 | DOWN |
| cytochrome P450 family 19 subfamily A member 1(CYP19A1) | ENSG00000137869 | CYP19A1 | UP |
| hyperpolarization activated cyclic nucleotide gated potassium channel 4(HCN4) | ENSG00000138622 | HCN4 | UP |
| annexin A3(ANXA3) | ENSG00000138772 | ANXA3 | DOWN |
| solute carrier family 39 member 8(SLC39A8) | ENSG00000138821 | SLC39A8 | DOWN |
| dual specificity phosphatase 6(DUSP6) | ENSG00000139318 | DUSP6 | DOWN |
| lumican(LUM) | ENSG00000139329 | LUM | DOWN |
| forkhead box N4(FOXN4) | ENSG00000139445 | FOXN4 | UP |
| activin A receptor like type 1(ACVRL1) | ENSG00000139567 | ACVRL1 | DOWN |
| serine/arginine repetitive matrix 4(SRRM4) | ENSG00000139767 | SRRM4 | UP |
| NOVA alternative splicing regulator 1(NOVA1) | ENSG00000139910 | NOVA1 | UP |
| Jun dimerization protein 2(JDP2) | ENSG00000140044 | JDP2 | DOWN |
| fibulin 5(FBLN5) | ENSG00000140092 | FBLN5 | DOWN |
| dual oxidase maturation factor 1(DUOXA1) | ENSG00000140254 | DUOXA1 | DOWN |
| dispatched RND transporter family member 2(DISP2) | ENSG00000140323 | DISP2 | UP |
| Rh family C glycoprotein(RHCG) | ENSG00000140519 | RHCG | UP |
| naked cuticle homolog 1(NKD1) | ENSG00000140807 | NKD1 | DOWN |
| ADAM metallopeptidase with thrombospondin type 1 motif 18(ADAMTS18) | ENSG00000140873 | ADAMTS18 | UP |
| rhomboid like 3(RHBDL3) | ENSG00000141314 | RHBDL3 | UP |
| adenylate cyclase activating polypeptide 1(ADCYAP1) | ENSG00000141433 | ADCYAP1 | UP |
| interferon induced transmembrane protein 3(IFITM3) | ENSG00000142089 | IFITM3 | DOWN |
| EPH receptor A2(EPHA2) | ENSG00000142627 | EPHA2 | DOWN |
| Rho guanine nucleotide exchange factor 19(ARHGEF19) | ENSG00000142632 | ARHGEF19 | UP |
| DMRT like family A2(DMRTA2) | ENSG00000142700 | DMRTA2 | UP |
| mitogen-activated protein kinase kinase kinase 6(MAP3K6) | ENSG00000142733 | MAP3K6 | DOWN |
| ficolin 3(FCN3) | ENSG00000142748 | FCN3 | DOWN |
| cysteine rich angiogenic inducer 61(CYR61) | ENSG00000142871 | CYR61 | DOWN |
| kinesin family member 2C(KIF2C) | ENSG00000142945 | KIF2C | UP |
| cytochrome P450 family 4 subfamily B member 1(CYP4B1) | ENSG00000142973 | CYP4B1 | DOWN |
| transmembrane protein 61(TMEM61) | ENSG00000143001 | TMEM61 | UP |
| cadherin EGF LAG seven-pass G-type receptor 2(CELSR2) | ENSG00000143126 | CELSR2 | UP |
| dermatopontin(DPT) | ENSG00000143196 | DPT | DOWN |
| hemicentin 1(HMCN1) | ENSG00000143341 | HMCN1 | DOWN |
| ADAMTS like 4(ADAMTSL4) | ENSG00000143382 | ADAMTSL4 | DOWN |
| selenium binding protein 1(SELENBP1) | ENSG00000143416 | SELENBP1 | DOWN |
| ras homolog family member B(RHOB) | ENSG00000143878 | RHOB | DOWN |
| mal, T-cell differentiation protein like(MALL) | ENSG00000144063 | MALL | DOWN |
| cyclic nucleotide gated channel alpha 3(CNGA3) | ENSG00000144191 | CNGA3 | UP |
| unc-80 homolog, NALCN activator(UNC80) | ENSG00000144406 | UNC80 | UP |
| hes family bHLH transcription factor 6(HES6) | ENSG00000144485 | HES6 | UP |
| RNA binding motif single stranded interacting protein 3(RBMS3) | ENSG00000144642 | RBMS3 | DOWN |
| cysteine and serine rich nuclear protein 1(CSRNP1) | ENSG00000144655 | CSRNP1 | DOWN |
| NFKB inhibitor zeta(NFKBIZ) | ENSG00000144802 | NFKBIZ | DOWN |
| syntaxin binding protein 5 like(STXBP5L) | ENSG00000145087 | STXBP5L | UP |
| endothelin converting enzyme 2(ECE2) | ENSG00000145194 | ECE2 | UP |
| OCIA domain containing 2(OCIAD2) | ENSG00000145247 | OCIAD2 | UP |
| oncostatin M receptor(OSMR) | ENSG00000145623 | OSMR | DOWN |
| cysteine rich protein 3(CRIP3) | ENSG00000146215 | CRIP3 | UP |
| tau tubulin kinase 1(TTBK1) | ENSG00000146216 | TTBK1 | UP |
| Rho GTPase activating protein 18(ARHGAP18) | ENSG00000146376 | ARHGAP18 | DOWN |
| chromosome X open reading frame 36(CXorf36) | ENSG00000147113 | CXorf36 | DOWN |
| ST18, C2H2C-type zinc finger(ST18) | ENSG00000147488 | ST18 | UP |
| transforming acidic coiled-coil containing protein 1(TACC1) | ENSG00000147526 | TACC1 | DOWN |
| cyclin dependent kinase inhibitor 2A(CDKN2A) | ENSG00000147889 | CDKN2A | UP |
| stomatin(STOM) | ENSG00000148175 | STOM | DOWN |
| ankyrin repeat domain 1(ANKRD1) | ENSG00000148677 | ANKRD1 | DOWN |
| internexin neuronal intermediate filament protein alpha(INA) | ENSG00000148798 | INA | UP |
| inositol 1,4,5-trisphosphate receptor interacting protein(ITPRIP) | ENSG00000148841 | ITPRIP | DOWN |
| secretoglobin family 1A member 1(SCGB1A1) | ENSG00000149021 | SCGB1A1 | DOWN |
| serpin family G member 1(SERPING1) | ENSG00000149131 | SERPING1 | DOWN |
| dopamine receptor D2(DRD2) | ENSG00000149295 | DRD2 | UP |
| gamma-glutamyltransferase light chain 1(GGTLC1) | ENSG00000149435 | GGTLC1 | DOWN |
| checkpoint kinase 1(CHEK1) | ENSG00000149554 | CHEK1 | UP |
| endothelial cell adhesion molecule(ESAM) | ENSG00000149564 | ESAM | DOWN |
| cadherin 22(CDH22) | ENSG00000149654 | CDH22 | UP |
| family with sequence similarity 57 member B(FAM57B) | ENSG00000149926 | FAM57B | UP |
| C-type lectin domain family 1 member A(CLEC1A) | ENSG00000150048 | CLEC1A | DOWN |
| large tumor suppressor kinase 2(LATS2) | ENSG00000150457 | LATS2 | DOWN |
| cysteine rich transmembrane BMP regulator 1(CRIM1) | ENSG00000150938 | CRIM1 | DOWN |
| inositol 1,4,5-trisphosphate receptor type 1(ITPR1) | ENSG00000150995 | ITPR1 | DOWN |
| endothelin receptor type A(EDNRA) | ENSG00000151617 | EDNRA | DOWN |
| nuclear receptor subfamily 3 group C member 2(NR3C2) | ENSG00000151623 | NR3C2 | DOWN |
| WW and C2 domain containing 2(WWC2) | ENSG00000151718 | WWC2 | DOWN |
| acyl-CoA synthetase long-chain family member 1(ACSL1) | ENSG00000151726 | ACSL1 | DOWN |
| heat shock protein family B (small) member 8(HSPB8) | ENSG00000152137 | HSPB8 | DOWN |
| guanylate cyclase 1 soluble subunit alpha 2(GUCY1A2) | ENSG00000152402 | GUCY1A2 | DOWN |
| SPARC like 1(SPARCL1) | ENSG00000152583 | SPARCL1 | DOWN |
| gap junction protein alpha 1(GJA1) | ENSG00000152661 | GJA1 | DOWN |
| utrophin(UTRN) | ENSG00000152818 | UTRN | DOWN |
| RAB3C, member RAS oncogene family(RAB3C) | ENSG00000152932 | RAB3C | UP |
| neurensin 1(NRSN1) | ENSG00000152954 | NRSN1 | UP |
| pleckstrin homology and RhoGEF domain containing G4B(PLEKHG4B) | ENSG00000153404 | PLEKHG4B | UP |
| ankyrin repeat domain 29(ANKRD29) | ENSG00000154065 | ANKRD29 | DOWN |
| roundabout guidance receptor 4(ROBO4) | ENSG00000154133 | ROBO4 | DOWN |
| ABI family member 3 binding protein(ABI3BP) | ENSG00000154175 | ABI3BP | DOWN |
| cytochrome P450 family 4 subfamily Z member 2, pseudogene(CYP4Z2P) | ENSG00000154198 | CYP4Z2P | DOWN |
| ubiquitin C-terminal hydrolase L1(UCHL1) | ENSG00000154277 | UCHL1 | UP |
| ADAM metallopeptidase with thrombospondin type 1 motif 1(ADAMTS1) | ENSG00000154734 | ADAMTS1 | DOWN |
| FYVE, RhoGEF and PH domain containing 5(FGD5) | ENSG00000154783 | FGD5 | DOWN |
| contactin associated protein like 5(CNTNAP5) | ENSG00000155052 | CNTNAP5 | UP |
| V-set and immunoglobulin domain containing 4(VSIG4) | ENSG00000155659 | VSIG4 | DOWN |
| kinesin family member 5A(KIF5A) | ENSG00000155980 | KIF5A | UP |
| WNT inhibitory factor 1(WIF1) | ENSG00000156076 | WIF1 | DOWN |
| UDP-GlcNAc:betaGal beta-1,3-N-acetylglucosaminyltransferase 7(B3GNT7) | ENSG00000156966 | B3GNT7 | DOWN |
| TSC22 domain family member 3(TSC22D3) | ENSG00000157514 | TSC22D3 | DOWN |
| potassium voltage-gated channel subfamily J member 15(KCNJ15) | ENSG00000157551 | KCNJ15 | DOWN |
| solute carrier family 34 member 2(SLC34A2) | ENSG00000157765 | SLC34A2 | DOWN |
| dihydropyrimidinase like 5(DPYSL5) | ENSG00000157851 | DPYSL5 | UP |
| family with sequence similarity 46 member B(FAM46B) | ENSG00000158246 | FAM46B | DOWN |
| shroom family member 4(SHROOM4) | ENSG00000158352 | SHROOM4 | DOWN |
| adenosylhomocysteinase like 2(AHCYL2) | ENSG00000158467 | AHCYL2 | DOWN |
| intelectin 2(ITLN2) | ENSG00000158764 | ITLN2 | DOWN |
| nucleophosmin/nucleoplasmin 2(NPM2) | ENSG00000158806 | NPM2 | UP |
| CUGBP, Elav-like family member 3(CELF3) | ENSG00000159409 | CELF3 | UP |
| family with sequence similarity 131 member B(FAM131B) | ENSG00000159784 | FAM131B | UP |
| zyxin(ZYX) | ENSG00000159840 | ZYX | DOWN |
| C3 and PZP like, alpha-2-macroglobulin domain containing 8(CPAMD8) | ENSG00000160111 | CPAMD8 | DOWN |
| chromosome 21 open reading frame 58(C21orf58) | ENSG00000160298 | C21orf58 | UP |
| spectrin beta, non-erythrocytic 4(SPTBN4) | ENSG00000160460 | SPTBN4 | UP |
| BR serine/threonine kinase 1(BRSK1) | ENSG00000160469 | BRSK1 | UP |
| interleukin 6 receptor(IL6R) | ENSG00000160712 | IL6R | DOWN |
| cholinergic receptor nicotinic beta 2 subunit(CHRNB2) | ENSG00000160716 | CHRNB2 | UP |
| parathyroid hormone 1 receptor(PTH1R) | ENSG00000160801 | PTH1R | DOWN |
| fibroblast growth factor receptor 4(FGFR4) | ENSG00000160867 | FGFR4 | DOWN |
| RecQ like helicase 4(RECQL4) | ENSG00000160957 | RECQL4 | UP |
| CUGBP, Elav-like family member 5(CELF5) | ENSG00000161082 | CELF5 | UP |
| integrin subunit alpha 5(ITGA5) | ENSG00000161638 | ITGA5 | DOWN |
| C-X-C motif chemokine ligand 16(CXCL16) | ENSG00000161921 | CXCL16 | DOWN |
| B-cell CLL/lymphoma 6B(BCL6B) | ENSG00000161940 | BCL6B | DOWN |
| cyclin F(CCNF) | ENSG00000162063 | CCNF | UP |
| progestin and adipoQ receptor family member 4(PAQR4) | ENSG00000162073 | PAQR4 | UP |
| PDZK1 interacting protein 1(PDZK1IP1) | ENSG00000162366 | PDZK1IP1 | DOWN |
| TAL bHLH transcription factor 1, erythroid differentiation factor(TAL1) | ENSG00000162367 | TAL1 | DOWN |
| alkaline phosphatase, liver/bone/kidney(ALPL) | ENSG00000162551 | ALPL | DOWN |
| kelch domain containing 8A(KLHDC8A) | ENSG00000162873 | KLHDC8A | UP |
| polymeric immunoglobulin receptor(PIGR) | ENSG00000162896 | PIGR | DOWN |
| coiled-coil domain containing 141(CCDC141) | ENSG00000163492 | CCDC141 | DOWN |
| transforming growth factor beta receptor 2(TGFBR2) | ENSG00000163513 | TGFBR2 | DOWN |
| prickle planar cell polarity protein 2(PRICKLE2) | ENSG00000163637 | PRICKLE2 | DOWN |
| C-type lectin domain family 3 member B(CLEC3B) | ENSG00000163815 | CLEC3B | DOWN |
| Kruppel like factor 15(KLF15) | ENSG00000163884 | KLF15 | DOWN |
| calcium/calmodulin dependent protein kinase II inhibitor 2(CAMK2N2) | ENSG00000163888 | CAMK2N2 | UP |
| sphingomyelin synthase 2(SGMS2) | ENSG00000164023 | SGMS2 | DOWN |
| endomucin(EMCN) | ENSG00000164035 | EMCN | DOWN |
| hydroxyprostaglandin dehydrogenase 15-(NAD)(HPGD) | ENSG00000164120 | HPGD | DOWN |
| hedgehog interacting protein(HHIP) | ENSG00000164161 | HHIP | DOWN |
| solute carrier family 29 member 4(SLC29A4) | ENSG00000164638 | SLC29A4 | UP |
| DLC1 Rho GTPase activating protein(DLC1) | ENSG00000164741 | DLC1 | DOWN |
| adenylate cyclase 1(ADCY1) | ENSG00000164742 | ADCY1 | UP |
| MAM domain containing 2(MAMDC2) | ENSG00000165072 | MAMDC2 | DOWN |
| protease, serine 37(PRSS37) | ENSG00000165076 | PRSS37 | UP |
| sushi, von Willebrand factor type A, EGF and pentraxin domain containing 1(SVEP1) | ENSG00000165124 | SVEP1 | DOWN |
| fructose-bisphosphatase 1(FBP1) | ENSG00000165140 | FBP1 | DOWN |
| ring finger protein 183(RNF183) | ENSG00000165188 | RNF183 | UP |
| WNK lysine deficient protein kinase 2(WNK2) | ENSG00000165238 | WNK2 | UP |
| spindle and kinetochore associated complex subunit 3(SKA3) | ENSG00000165480 | SKA3 | UP |
| chromosome 10 open reading frame 10(C10orf10) | ENSG00000165507 | C10orf10 | DOWN |
| transmembrane protein 63C(TMEM63C) | ENSG00000165548 | TMEM63C | UP |
| neuroglobin(NGB) | ENSG00000165553 | NGB | UP |
| KIAA1462(KIAA1462) | ENSG00000165757 | KIAA1462 | DOWN |
| butyrophilin like 9(BTNL9) | ENSG00000165810 | BTNL9 | DOWN |
| E2F transcription factor 7(E2F7) | ENSG00000165891 | E2F7 | UP |
| transmembrane protein 100(TMEM100) | ENSG00000166292 | TMEM100 | DOWN |
| microfibrillar associated protein 4(MFAP4) | ENSG00000166482 | MFAP4 | DOWN |
| polo like kinase 1(PLK1) | ENSG00000166851 | PLK1 | UP |
| carbonic anhydrase 4(CA4) | ENSG00000167434 | CA4 | DOWN |
| SERTA domain containing 3(SERTAD3) | ENSG00000167565 | SERTAD3 | DOWN |
| transmembrane protein 145(TMEM145) | ENSG00000167619 | TMEM145 | UP |
| protein phosphatase 1 regulatory inhibitor subunit 14A(PPP1R14A) | ENSG00000167641 | PPP1R14A | DOWN |
| chromosome 19 open reading frame 33(C19orf33) | ENSG00000167644 | C19orf33 | DOWN |
| ATCAY, caytaxin(ATCAY) | ENSG00000167654 | ATCAY | UP |
| mannosyl (alpha-1,6-)-glycoprotein beta-1,6-N-acetyl-glucosaminyltransferase, isozyme B(MGAT5B) | ENSG00000167889 | MGAT5B | UP |
| ATP binding cassette subfamily A member 3(ABCA3) | ENSG00000167972 | ABCA3 | DOWN |
| G protein subunit gamma 4(GNG4) | ENSG00000168243 | GNG4 | UP |
| kinesin family member 5C(KIF5C) | ENSG00000168280 | KIF5C | UP |
| family with sequence similarity 107 member A(FAM107A) | ENSG00000168309 | FAM107A | DOWN |
| tenascin XB(TNXB) | ENSG00000168477 | TNXB | DOWN |
| leucine rich repeat LGI family member 3(LGI3) | ENSG00000168481 | LGI3 | DOWN |
| surfactant protein C(SFTPC) | ENSG00000168484 | SFTPC | DOWN |
| bone morphogenetic protein 1(BMP1) | ENSG00000168487 | BMP1 | DOWN |
| serum deprivation response(SDPR) | ENSG00000168497 | SDPR | DOWN |
| interleukin 7 receptor(IL7R) | ENSG00000168685 | IL7R | DOWN |
| atonal bHLH transcription factor 8(ATOH8) | ENSG00000168874 | ATOH8 | DOWN |
| surfactant protein B(SFTPB) | ENSG00000168878 | SFTPB | DOWN |
| vesicle associated membrane protein 5(VAMP5) | ENSG00000168899 | VAMP5 | DOWN |
| phospholipase A2 group IVF(PLA2G4F) | ENSG00000168907 | PLA2G4F | DOWN |
| collagen type IV alpha 3 chain(COL4A3) | ENSG00000169031 | COL4A3 | DOWN |
| adrenoceptor beta 2(ADRB2) | ENSG00000169252 | ADRB2 | DOWN |
| natriuretic peptide receptor 1(NPR1) | ENSG00000169418 | NPR1 | DOWN |
| chloride intracellular channel 3(CLIC3) | ENSG00000169583 | CLIC3 | DOWN |
| cytoskeleton associated protein 2 like(CKAP2L) | ENSG00000169607 | CKAP2L | UP |
| BUB1 mitotic checkpoint serine/threonine kinase(BUB1) | ENSG00000169679 | BUB1 | UP |
| LIM domain binding 2(LDB2) | ENSG00000169744 | LDB2 | DOWN |
| neuroligin 1(NLGN1) | ENSG00000169760 | NLGN1 | UP |
| myosin VIIB(MYO7B) | ENSG00000169994 | MYO7B | DOWN |
| solute carrier family 16 member 5(SLC16A5) | ENSG00000170190 | SLC16A5 | DOWN |
| cyclin dependent kinase 1(CDK1) | ENSG00000170312 | CDK1 | UP |
| Fos proto-oncogene, AP-1 transcription factor subunit(FOS) | ENSG00000170345 | FOS | DOWN |
| short chain dehydrogenase/reductase family 16C, member 5(SDR16C5) | ENSG00000170786 | SDR16C5 | DOWN |
| sphingosine-1-phosphate receptor 1(S1PR1) | ENSG00000170989 | S1PR1 | DOWN |
| formyl peptide receptor 1(FPR1) | ENSG00000171051 | FPR1 | DOWN |
| prostate and testis expressed 1(PATE1) | ENSG00000171053 | PATE1 | UP |
| GTPase, IMAP family member 8(GIMAP8) | ENSG00000171115 | GIMAP8 | DOWN |
| potassium voltage-gated channel modifier subfamily G member 3(KCNG3) | ENSG00000171126 | KCNG3 | UP |
| kinase suppressor of ras 2(KSR2) | ENSG00000171435 | KSR2 | UP |
| cyclin dependent kinase 5 regulatory subunit 2(CDK5R2) | ENSG00000171450 | CDK5R2 | UP |
| HOP homeobox(HOPX) | ENSG00000171476 | HOPX | DOWN |
| ribonucleotide reductase regulatory subunit M2(RRM2) | ENSG00000171848 | RRM2 | UP |
| aquaporin 4(AQP4) | ENSG00000171885 | AQP4 | DOWN |
| synaptopodin(SYNPO) | ENSG00000171992 | SYNPO | DOWN |
| laminin subunit beta 2(LAMB2) | ENSG00000172037 | LAMB2 | DOWN |
| calbindin 2(CALB2) | ENSG00000172137 | CALB2 | UP |
| Rho family GTPase 1(RND1) | ENSG00000172602 | RND1 | DOWN |
| EGF like domain multiple 7(EGFL7) | ENSG00000172889 | EGFL7 | DOWN |
| heparanase(HPSE) | ENSG00000173083 | HPSE | UP |
| multimerin 2(MMRN2) | ENSG00000173269 | MMRN2 | DOWN |
| tribbles pseudokinase 1(TRIB1) | ENSG00000173334 | TRIB1 | DOWN |
| complement C1q A chain(C1QA) | ENSG00000173372 | C1QA | DOWN |
| neuron derived neurotrophic factor(NDNF) | ENSG00000173376 | NDNF | DOWN |
| INSM transcriptional repressor 1(INSM1) | ENSG00000173404 | INSM1 | UP |
| protein tyrosine phosphatase, receptor type M(PTPRM) | ENSG00000173482 | PTPRM | DOWN |
| TNF receptor superfamily member 10d(TNFRSF10D) | ENSG00000173530 | TNFRSF10D | DOWN |
| heart development protein with EGF like domains 1(HEG1) | ENSG00000173706 | HEG1 | DOWN |
| potassium voltage-gated channel subfamily H member 6(KCNH6) | ENSG00000173826 | KCNH6 | UP |
| polo like kinase 3(PLK3) | ENSG00000173846 | PLK3 | DOWN |
| homeobox B2(HOXB2) | ENSG00000173917 | HOXB2 | UP |
| leucine rich repeat and Ig domain containing 2(LINGO2) | ENSG00000174482 | LINGO2 | UP |
| solute carrier organic anion transporter family member 2A1(SLCO2A1) | ENSG00000174640 | SLCO2A1 | DOWN |
| chromosome 4 open reading frame 32(C4orf32) | ENSG00000174749 | C4orf32 | DOWN |
| frizzled class receptor 4(FZD4) | ENSG00000174804 | FZD4 | DOWN |
| cornichon family AMPA receptor auxiliary protein 2(CNIH2) | ENSG00000174871 | CNIH2 | UP |
| ubiquitin conjugating enzyme E2 C(UBE2C) | ENSG00000175063 | UBE2C | UP |
| chromosome 1 open reading frame 127(C1orf127) | ENSG00000175262 | C1orf127 | UP |
| lipoprotein lipase(LPL) | ENSG00000175445 | LPL | DOWN |
| purinergic receptor P2Y2(P2RY2) | ENSG00000175591 | P2RY2 | DOWN |
| alpha-2-macroglobulin(A2M) | ENSG00000175899 | A2M | DOWN |
| forkhead box G1(FOXG1) | ENSG00000176165 | FOXG1 | UP |
| C-type lectin domain family 14 member A(CLEC14A) | ENSG00000176435 | CLEC14A | DOWN |
| cyclin dependent kinase 5 regulatory subunit 1(CDK5R1) | ENSG00000176749 | CDK5R1 | UP |
| transcription elongation regulator 1 like(TCERG1L) | ENSG00000176769 | TCERG1L | UP |
| NCK associated protein 5(NCKAP5) | ENSG00000176771 | NCKAP5 | DOWN |
| MIR7-3 host gene(MIR7-3HG) | ENSG00000176840 | MIR7-3HG | UP |
| SRY-box 11(SOX11) | ENSG00000176887 | SOX11 | UP |
| ribosomal modification protein rimK like family member A(RIMKLA) | ENSG00000177181 | RIMKLA | UP |
| polymerase I and transcript release factor(PTRF) | ENSG00000177469 | PTRF | DOWN |
| piggyBac transposable element derived 5(PGBD5) | ENSG00000177614 | PGBD5 | UP |
| serine/arginine repetitive matrix 3(SRRM3) | ENSG00000177679 | SRRM3 | UP |
| ALS2 C-terminal like(ALS2CL) | ENSG00000178038 | ALS2CL | DOWN |
| transmembrane protein 151B(TMEM151B) | ENSG00000178233 | TMEM151B | UP |
| thrombomodulin(THBD) | ENSG00000178726 | THBD | DOWN |
| GTPase, IMAP family member 7(GIMAP7) | ENSG00000179144 | GIMAP7 | DOWN |
| transmembrane protein 125(TMEM125) | ENSG00000179178 | TMEM125 | DOWN |
| arachidonate 15-lipoxygenase, type B(ALOX15B) | ENSG00000179593 | ALOX15B | DOWN |
| glutamate metabotropic receptor 8(GRM8) | ENSG00000179603 | GRM8 | UP |
| cadherin 5(CDH5) | ENSG00000179776 | CDH5 | DOWN |
| myeloid associated differentiation marker(MYADM) | ENSG00000179820 | MYADM | DOWN |
| neurexin 1(NRXN1) | ENSG00000179915 | NRXN1 | UP |
| histone cluster 1 H2B family member c(HIST1H2BC) | ENSG00000180596 | HIST1H2BC | UP |
| perforin 1(PRF1) | ENSG00000180644 | PRF1 | DOWN |
| G protein-coupled receptor 137C(GPR137C) | ENSG00000180998 | GPR137C | UP |
| rhotekin 2(RTKN2) | ENSG00000182010 | RTKN2 | DOWN |
| neurexophilin 4(NXPH4) | ENSG00000182379 | NXPH4 | UP |
| potassium voltage-gated channel subfamily B member 2(KCNB2) | ENSG00000182674 | KCNB2 | UP |
| chromosome 1 open reading frame 116(C1orf116) | ENSG00000182795 | C1orf116 | DOWN |
| SRY-box 1(SOX1) | ENSG00000182968 | SOX1 | UP |
| C2 calcium dependent domain containing 4C(C2CD4C) | ENSG00000183186 | C2CD4C | UP |
| ovochymase 2 (gene/pseudogene)(OVCH2) | ENSG00000183378 | OVCH2 | DOWN |
| SH2 domain containing 7(SH2D7) | ENSG00000183476 | SH2D7 | UP |
| mex-3 RNA binding family member B(MEX3B) | ENSG00000183496 | MEX3B | UP |
| poly(rC) binding protein 3(PCBP3) | ENSG00000183570 | PCBP3 | UP |
| gastrokine 2(GKN2) | ENSG00000183607 | GKN2 | DOWN |
| beta-1,3-galactosyltransferase 5(B3GALT5) | ENSG00000183778 | B3GALT5 | UP |
| family with sequence similarity 162 member B(FAM162B) | ENSG00000183807 | FAM162B | DOWN |
| zinc finger protein 730(ZNF730) | ENSG00000183850 | ZNF730 | UP |
| IQ motif containing GTPase activating protein 3(IQGAP3) | ENSG00000183856 | IQGAP3 | UP |
| transmembrane protease, serine 2(TMPRSS2) | ENSG00000184012 | TMPRSS2 | DOWN |
| claudin 5(CLDN5) | ENSG00000184113 | CLDN5 | DOWN |
| slit guidance ligand 3(SLIT3) | ENSG00000184347 | SLIT3 | DOWN |
| histone cluster 1 H1 family member b(HIST1H1B) | ENSG00000184357 | HIST1H1B | UP |
| colony stimulating factor 1(CSF1) | ENSG00000184371 | CSF1 | DOWN |
| potassium voltage-gated channel subfamily H member 7(KCNH7) | ENSG00000184611 | KCNH7 | UP |
| RALY RNA binding protein-like(RALYL) | ENSG00000184672 | RALYL | UP |
| lipocalin 12(LCN12) | ENSG00000184925 | LCN12 | UP |
| mannosidase endo-alpha like(MANEAL) | ENSG00000185090 | MANEAL | UP |
| surfactant protein A2(SFTPA2) | ENSG00000185303 | SFTPA2 | DOWN |
| mucin 1, cell surface associated(MUC1) | ENSG00000185499 | MUC1 | DOWN |
| ATPase H+/K+ transporting beta subunit(ATP4B) | ENSG00000186009 | ATP4B | UP |
| suppressor APC domain containing 2(SAPCD2) | ENSG00000186193 | SAPCD2 | UP |
| kallikrein related peptidase 12(KLK12) | ENSG00000186474 | KLK12 | UP |
| family with sequence similarity 183 member A(FAM183A) | ENSG00000186973 | FAM183A | UP |
| LY6/PLAUR domain containing 6(LYPD6) | ENSG00000187123 | LYPD6 | UP |
| sprouty RTK signaling antagonist 4(SPRY4) | ENSG00000187678 | SPRY4 | DOWN |
| platelet endothelial aggregation receptor 1(PEAR1) | ENSG00000187800 | PEAR1 | DOWN |
| deleted in malignant brain tumors 1(DMBT1) | ENSG00000187908 | DMBT1 | DOWN |
| delta/notch like EGF repeat containing(DNER) | ENSG00000187957 | DNER | UP |
| SH3 domain binding kinase 1(SBK1) | ENSG00000188322 | SBK1 | UP |
| H2A histone family member X(H2AFX) | ENSG00000188486 | H2AFX | UP |
| hemoglobin subunit alpha 2(HBA2) | ENSG00000188536 | HBA2 | DOWN |
| frizzled class receptor 9(FZD9) | ENSG00000188763 | FZD9 | UP |
| proline and arginine rich end leucine rich repeat protein(PRELP) | ENSG00000188783 | PRELP | DOWN |
| leucine rich repeat kinase 2(LRRK2) | ENSG00000188906 | LRRK2 | DOWN |
| family with sequence similarity 111 member B(FAM111B) | ENSG00000189057 | FAM111B | UP |
| monoamine oxidase A(MAOA) | ENSG00000189221 | MAOA | DOWN |
| paired box 5(PAX5) | ENSG00000196092 | PAX5 | UP |
| major histocompatibility complex, class II, DR beta 1(HLA-DRB1) | ENSG00000196126 | HLA-DRB1 | DOWN |
| myelin transcription factor 1(MYT1) | ENSG00000196132 | MYT1 | UP |
| S100 calcium binding protein A4(S100A4) | ENSG00000196154 | S100A4 | DOWN |
| FAT atypical cadherin 4(FAT4) | ENSG00000196159 | FAT4 | DOWN |
| zinc finger protein 681(ZNF681) | ENSG00000196172 | ZNF681 | UP |
| surfactant associated 2(SFTA2) | ENSG00000196260 | SFTA2 | DOWN |
| GTPase, IMAP family member 5(GIMAP5) | ENSG00000196329 | GIMAP5 | DOWN |
| CD55 molecule (Cromer blood group)(CD55) | ENSG00000196352 | CD55 | DOWN |
| ELAV like RNA binding protein 3(ELAVL3) | ENSG00000196361 | ELAVL3 | UP |
| histone cluster 1 H2A family member g(HIST1H2AG) | ENSG00000196787 | HIST1H2AG | UP |
| prostate and testis expressed 2(PATE2) | ENSG00000196844 | PATE2 | UP |
| solute carrier family 6 member 17(SLC6A17) | ENSG00000197106 | SLC6A17 | UP |
| serpin family A member 1(SERPINA1) | ENSG00000197249 | SERPINA1 | DOWN |
| oxoglutarate dehydrogenase-like(OGDHL) | ENSG00000197444 | OGDHL | UP |
| S100 calcium binding protein A10(S100A10) | ENSG00000197747 | S100A10 | DOWN |
| coiled-coil domain containing 69(CCDC69) | ENSG00000198624 | CCDC69 | DOWN |
| 3'-phosphoadenosine 5'-phosphosulfate synthase 2(PAPSS2) | ENSG00000198682 | PAPSS2 | DOWN |
| Rho guanine nucleotide exchange factor 15(ARHGEF15) | ENSG00000198844 | ARHGEF15 | DOWN |
| carboxylesterase 1(CES1) | ENSG00000198848 | CES1 | DOWN |
| G protein-coupled receptor kinase 5(GRK5) | ENSG00000198873 | GRK5 | DOWN |
| transglutaminase 2(TGM2) | ENSG00000198959 | TGM2 | DOWN |
| calpain 8(CAPN8) | ENSG00000203697 | CAPN8 | DOWN |
| zyg-11 family member A, cell cycle regulator(ZYG11A) | ENSG00000203995 | ZYG11A | UP |
| major histocompatibility complex, class II, DM alpha(HLA-DMA) | ENSG00000204257 | HLA-DMA | DOWN |
| major histocompatibility complex, class II, DR alpha(HLA-DRA) | ENSG00000204287 | HLA-DRA | DOWN |
| notch 4(NOTCH4) | ENSG00000204301 | NOTCH4 | DOWN |
| advanced glycosylation end-product specific receptor(AGER) | ENSG00000204305 | AGER | DOWN |
| family with sequence similarity 155 member A(FAM155A) | ENSG00000204442 | FAM155A | UP |
| major histocompatibility complex, class I, E(HLA-E) | ENSG00000204592 | HLA-E | DOWN |
| protocadherin alpha 9(PCDHA9) | ENSG00000204961 | PCDHA9 | UP |
| hemoglobin subunit alpha 1(HBA1) | ENSG00000206172 | HBA1 | DOWN |
| collagen type VI alpha 6 chain(COL6A6) | ENSG00000206384 | COL6A6 | DOWN |
| transmembrane protein 200C(TMEM200C) | ENSG00000206432 | TMEM200C | UP |
| vestigial like family member 3(VGLL3) | ENSG00000206538 | VGLL3 | DOWN |
| XK related 4(XKR4) | ENSG00000206579 | XKR4 | UP |
| glutathione peroxidase 3(GPX3) | ENSG00000211445 | GPX3 | DOWN |
| epithelial membrane protein 2(EMP2) | ENSG00000213853 | EMP2 | DOWN |
| neuronal pentraxin receptor(NPTXR) | ENSG00000221890 | NPTXR | UP |
| maternally expressed 9 (non-protein coding)(MEG9) | ENSG00000223403 | MEG9 | UP |
| surfactant associated 1, pseudogene(SFTA1P) | ENSG00000225383 | SFTA1P | DOWN |
| NLGN1 antisense RNA 1(NLGN1-AS1) | ENSG00000228213 | NLGN1-AS1 | UP |
| major histocompatibility complex, class II, DP alpha 1(HLA-DPA1) | ENSG00000231389 | HLA-DPA1 | DOWN |
| histone cluster 1 H2B family member n(HIST1H2BN) | ENSG00000233822 | HIST1H2BN | UP |
| DiGeorge syndrome critical region gene 5 (non-protein coding)(DGCR5) | ENSG00000237517 | DGCR5 | UP |
| aquaporin 1 (Colton blood group)(AQP1) | ENSG00000240583 | AQP1 | DOWN |
| hemoglobin subunit beta(HBB) | ENSG00000244734 | HBB | DOWN |
| uncharacterized LOC100652791(GS1-24F4.2) | ENSG00000245857 | GS1-24F4.2 | UP |
| endothelial cell surface expressed chemotaxis and apoptosis regulator(ECSCR) | ENSG00000249751 | ECSCR | DOWN |
| SH3 and multiple ankyrin repeat domains 3(SHANK3) | ENSG00000251322 | SHANK3 | DOWN |
| transmembrane protein 179(TMEM179) | ENSG00000258986 | TMEM179 | UP |
| XK related 7(XKR7) | ENSG00000260903 | XKR7 | UP |
| solute carrier family 6 member 14(SLC6A14) | ENSG00000268104 | SLC6A14 | DOWN |
| matrix metallopeptidase 28(MMP28) | ENSG00000271447 | MMP28 | DOWN |
| zinc finger and BTB domain containing 8B(ZBTB8B) | ENSG00000273274 | ZBTB8B | UP |
| ATP/GTP binding protein like 1(AGBL1) | ENSG00000273540 | AGBL1 | DOWN |
| histone cluster 1 H3 family member b(HIST1H3B) | ENSG00000274267 | HIST1H3B | UP |
| histone cluster 1 H3 family member i(HIST1H3I) | ENSG00000275379 | HIST1H3I | UP |
| uroplakin 3B(UPK3B) | ENSG00000276184 | UPK3B | DOWN |
| SRC kinase signaling inhibitor 1(SRCIN1) | ENSG00000277363 | SRCIN1 | UP |
| glycosylphosphatidylinositol anchored high density lipoprotein binding protein 1(GPIHBP1) | ENSG00000277494 | GPIHBP1 | DOWN |
| transcription elongation factor B subunit 3C like(TCEB3CL) | ENSG00000278674 | TCEB3CL | UP |
| histone cluster 1 H3 family member h(HIST1H3H) | ENSG00000278828 | HIST1H3H | UP |
|  |  | AC007740.1 | UP |
|  |  | CCDC64 | UP |
|  |  | SOGA2 | UP |
|  |  | AC018359.1 | UP |
|  |  | RP11-102F4.3 | UP |
|  |  | RP11-5A11.1 | UP |
|  |  | C8orf47 | UP |
|  |  | STMN1 | UP |
|  |  | 42250 | UP |
|  |  | RP11-923I11.1 | UP |
|  |  | HMGB1P41 | UP |
|  |  | RP11-229P13.23 | UP |
|  |  | TTC3-AS1 | UP |
|  |  | AC015933.2 | UP |
|  |  | RP11-715J22.2 | UP |
|  |  | CELSR3 | UP |
|  |  | PTCHD2 | UP |
|  |  | NRG1-IT1 | UP |
|  |  | FAM155A-IT1 | UP |
|  |  | BAI2 | UP |
|  |  | RP11-688I9.4 | UP |
|  |  | RP11-923I11.3 | UP |
|  |  | RP11-480I12.5 | UP |
|  |  | UBA52P6 | UP |
|  |  | RP11-672L10.2 | UP |
|  |  | RP11-715J22.4 | UP |
|  |  | RP5-1092A11.5 | UP |
|  |  | ZNF643 | UP |
|  |  | RP5-890O15.3 | UP |
|  |  | CTD-2231H16.1 | UP |
|  |  | AL592528.1 | UP |
|  |  | ACTBP7 | UP |
|  |  | RPS11P6 | UP |
|  |  | FAM123C | UP |
|  |  | RP11-290P14.2 | UP |
|  |  | AP000251.2 | UP |
|  |  | RP11-317N12.1 | UP |
|  |  | AC105402.4 | UP |
|  |  | AC012123.1 | UP |
|  |  | LL0XNC01-116E7.2 | UP |
|  |  | AC009113.1 | UP |
|  |  | RP11-680F20.10 | UP |
|  |  | C18orf56 | UP |
|  |  | AC012317.1 | UP |
|  |  | RP11-477I4.3 | UP |
|  |  | RP13-15E13.1 | UP |
|  |  | LL0XNC01-116E7.1 | UP |
|  |  | RP11-65D24.2 | UP |
|  |  | C2orf48 | UP |
|  |  | FAM201A | UP |
|  |  | RP11-1145L24.1 | UP |
|  |  | AC003102.3 | UP |
|  |  | RP11-175B12.2 | UP |
|  |  | LINC00354 | UP |
|  |  | RP11-73C9.1 | UP |
|  |  | RP11-629G13.1 | UP |
|  |  | C8orf12 | UP |
|  |  | RP11-342L8.2 | UP |
|  |  | AP000569.2 | UP |
|  |  | RP11-159H10.3 | UP |
|  |  | RP11-85O21.5 | UP |
|  |  | RP11-337L12.1 | UP |
|  |  | GAPDHP55 | UP |
|  |  | RP1-140A9.1 | UP |
|  |  | AC073236.3 | UP |
|  |  | CTD-2555A7.2 | UP |
|  |  | RP11-441F2.1 | UP |
|  |  | RP11-247C2.2 | UP |
|  |  | RP11-26M5.3 | UP |
|  |  | RP11-566K11.2 | UP |
|  |  | NRG1-IT2 | UP |
|  |  | RP11-551L14.6 | UP |
|  |  | NKX2-2-AS1 | UP |
|  |  | RP11-114H24.5 | DOWN |
|  |  | RP11-325F22.5 | DOWN |
|  |  | RP11-475O23.2 | DOWN |
|  |  | RP11-864I4.3 | DOWN |
|  |  | RP11-417N10.3 | DOWN |
|  |  | GPRC5A | DOWN |
|  |  | CTD-2531D15.4 | DOWN |
|  |  | MRC1L1 | DOWN |
|  |  | CTC-558O2.2 | DOWN |
|  |  | AC098617.2 | DOWN |
|  |  | RP11-286E11.1 | DOWN |
|  |  | RP11-664I21.6 | DOWN |
|  |  | RP11-702H23.6 | DOWN |
|  |  | GPR116 | DOWN |
|  |  | Z98256.1 | DOWN |
|  |  | FIGF | DOWN |
|  |  | RP3-510L9.1 | DOWN |
|  |  | RP1-127D3.4 | DOWN |
|  |  | RP11-446J8.1 | DOWN |
|  |  | RP11-613D13.8 | DOWN |
|  |  | CTD-2033D15.1 | DOWN |
|  |  | RPS12P5 | DOWN |
|  |  | CD97 | DOWN |
|  |  | GPR133 | DOWN |
|  |  | AC008440.5 | DOWN |
|  |  | RP11-76C10.5 | DOWN |
|  |  | PER1 | DOWN |
|  |  | AC003991.3 | DOWN |
|  |  | RP3-425P12.4 | DOWN |
|  |  | AP001189.4 | DOWN |
|  |  | STARD13-AS2 | DOWN |
|  |  | MIR22HG | DOWN |
|  |  | VWFP1 | DOWN |
|  |  | RP11-27M24.1 | DOWN |
|  |  | RP11-54A4.2 | DOWN |
|  |  | GCOM1 | DOWN |
|  |  | RP11-88L24.4 | DOWN |
|  |  | AC093110.3 | DOWN |
|  |  | RP11-433J22.2 | DOWN |
|  |  | RP11-420K8.1 | DOWN |
|  |  | RP11-664I21.5 | DOWN |
|  |  | RP3-449M8.3 | DOWN |
|  |  | RP11-815J21.3 | DOWN |
|  |  | RP11-46C24.8 | DOWN |
|  |  | RP3-340B19.5 | DOWN |
|  |  | RP11-264F23.3 | DOWN |
|  |  | ITGB6 | DOWN |
|  |  | RP11-290F20.3 | DOWN |
|  |  | RP11-27M24.3 | DOWN |
|  |  | GALNTL4 | DOWN |
|  |  | GAPDHP49 | DOWN |
|  |  | CTD-2562J17.7 | DOWN |
|  |  | SYNE1-AS1 | DOWN |
|  |  | RP11-27M24.2 | DOWN |
|  |  | RP11-588H23.3 | DOWN |
|  |  | AC058791.2 | DOWN |
|  |  | C11orf9 | DOWN |
|  |  | RP11-524D16__A.3 | DOWN |
|  |  | RP11-806O11.1 | DOWN |
|  |  | KIAA1274 | DOWN |
|  |  | CCDC48 | DOWN |
|  |  | RP11-61I13.3 | DOWN |
|  |  | RP11-392P7.6 | DOWN |
|  |  | CTC-558O2.1 | DOWN |
|  |  | RP5-977B1.11 | DOWN |
|  |  | RP11-415D17.3 | DOWN |
|  |  | AC006159.5 | DOWN |
|  |  | RP11-388M20.2 | DOWN |
|  |  | RP11-79H23.3 | DOWN |
|  |  | RP11-815J21.2 | DOWN |
|  |  | RP11-1024P17.1 | DOWN |
|  |  | LDLR | DOWN |
|  |  | CSDA | DOWN |
|  |  | PIEZO1 | DOWN |
|  |  | GPR126 | DOWN |
|  |  | RP11-124N14.3 | DOWN |
|  |  | RP11-506G7.1 | DOWN |
|  |  | CTD-2207P18.1 | DOWN |
|  |  | LINC00472 | DOWN |
|  |  | AKAP13 | DOWN |
|  |  | RP11-129B22.1 | DOWN |
|  |  | LINC00312 | DOWN |
|  |  | IMPDH1P10 | DOWN |
|  |  | TENC1 | DOWN |
|  |  | RP11-697K23.1 | DOWN |
|  |  | CMAHP | DOWN |
|  |  | NPC2 | DOWN |

| **Table S9: Differentially Expressed Genes in SINEC** | | | |
| --- | --- | --- | --- |
|  |  |  |  |
| **Name** | **ENSEMBL_ID** | **Gene Symbol** | **Expression** |
| Tissue factor pathway inhibitor (lipoprotein-associated coagulation inhibitor) | ENSG00000003436 | TFPI | DOWN |
| Nuclear receptor subfamily 1, group H, member 4 | ENSG00000012504 | NR1H4 | DOWN |
| Pleckstrin homology domain containing, family B (evectins) member 1 | ENSG00000021300 | PLEKHB1 | UP |
| Carbamoyl-phosphate synthase 1, mitochondrial | ENSG00000021826 | CPS1 | DOWN |
| Ras-related GTP binding D | ENSG00000025039 | RRAGD | UP |
| Nuclear receptor subfamily 1, group H, member 3 | ENSG00000025434 | NR1H3 | DOWN |
| ATPase, H+ transporting, lysosomal 50/57kDa, V1 subunit H | ENSG00000047249 | ATP6V1H | UP |
| R3H domain containing 1 | ENSG00000048991 | R3HDM1 | UP |
| Pleckstrin homology domain containing, family A member 5 | ENSG00000052126 | PLEKHA5 | UP |
| Potassium voltage-gated channel, subfamily H (eag-related), member 2 | ENSG00000055118 | KCNH2 | UP |
| Cyclin-dependent kinase 14 | ENSG00000058091 | CDK14 | UP |
| PMS1 postmeiotic segregation increased 1 (S. cerevisiae) | ENSG00000064933 | PMS1 | UP |
| Synaptosomal-associated protein, 91kDa | ENSG00000065609 | SNAP91 | UP |
| Tumor necrosis factor receptor superfamily, member 1A | ENSG00000067182 | TNFRSF1A | DOWN |
| Monoamine oxidase B | ENSG00000069535 | MAOB | DOWN |
| Inhibitor of kappa light polypeptide gene enhancer in B-cells, kinase complex-associated protein | ENSG00000070061 | IKBKAP | UP |
| Profilin 2 | ENSG00000070087 | PFN2 | UP |
| Asparagine synthetase (glutamine-hydrolyzing) | ENSG00000070669 | ASNS | UP |
| LIM and cysteine-rich domains 1 | ENSG00000071282 | LMCD1 | UP |
| ATPase, H+ transporting, lysosomal accessory protein 1 | ENSG00000071553 | ATP6AP1 | UP |
| Helicase-like transcription factor | ENSG00000071794 | HLTF | UP |
| Family with sequence similarity 50, member A | ENSG00000071859 | FAM50A | UP |
| Cadherin 19, type 2 | ENSG00000071991 | CDH19 | DOWN |
| Collapsin response mediator protein 1 | ENSG00000072832 | CRMP1 | UP |
| Nebulette | ENSG00000078114 | NEBL | UP |
| Solute carrier family 4 (sodium bicarbonate cotransporter), member 4 | ENSG00000080493 | SLC4A4 | DOWN |
| Apolipoprotein B | ENSG00000084674 | APOB | DOWN |
| CD59 molecule, complement regulatory protein | ENSG00000085063 | CD59 | UP |
| DDHD domain containing 2 | ENSG00000085788 | DDHD2 | UP |
| Hydroxysteroid (17-beta) dehydrogenase 2 | ENSG00000086696 | HSD17B2 | DOWN |
| Ubiquitin specific peptidase 48 | ENSG00000090686 | USP48 | UP |
| Neuronal cell adhesion molecule | ENSG00000091129 | NRCAM | UP |
| Regulator of G-protein signaling 17 | ENSG00000091844 | RGS17 | UP |
| Dihydropyrimidinase-like 2 | ENSG00000092964 | DPYSL2 | UP |
| SUN domain containing ossification factor | ENSG00000094975 | SUCO | UP |
| B-cell linker | ENSG00000095585 | BLNK | DOWN |
| Sorbin and SH3 domain containing 1 | ENSG00000095637 | SORBS1 | DOWN |
| EF-hand domain (C-terminal) containing 1 | ENSG00000096093 | EFHC1 | UP |
| Cold inducible RNA binding protein | ENSG00000099622 | CIRBP | UP |
| Heme oxygenase (decycling) 1 | ENSG00000100292 | HMOX1 | DOWN |
| Phosphoenolpyruvate carboxykinase 2 (mitochondrial) | ENSG00000100889 | PCK2 | DOWN |
| Ninein-like | ENSG00000101004 | NINL | UP |
| Zinc finger, MYND-type containing 8 | ENSG00000101040 | ZMYND8 | UP |
| Lipin 2 | ENSG00000101577 | LPIN2 | DOWN |
| Proprotein convertase subtilisin/kexin type 1 inhibitor | ENSG00000102109 | PCSK1N | UP |
| Ubiquitin specific peptidase 11 | ENSG00000102226 | USP11 | UP |
| NME/NM23 nucleoside diphosphate kinase 3 | ENSG00000103024 | NME3 | UP |
| Adaptor-related protein complex 3, beta 2 subunit | ENSG00000103723 | AP3B2 | UP |
| Cathepsin H | ENSG00000103811 | CTSH | DOWN |
| Secretogranin III | ENSG00000104112 | SCG3 | UP |
| Prostate tumor overexpressed 1 | ENSG00000104960 | PTOV1 | UP |
| Tubulin folding cofactor B | ENSG00000105254 | TBCB | UP |
| Amyloid beta (A4) precursor-like protein 1 | ENSG00000105290 | APLP1 | UP |
| Inositol-3-phosphate synthase 1 | ENSG00000105655 | ISYNA1 | UP |
| Syntaxin 1A (brain) | ENSG00000106089 | STX1A | UP |
| Solute carrier family 1 (neuronal/epithelial high affinity glutamate transporter, system Xag), member 1 | ENSG00000106688 | SLC1A1 | DOWN |
| Dynamin 1 | ENSG00000106976 | DNM1 | UP |
| Adenylate kinase 1 | ENSG00000106992 | AK1 | UP |
| Neural proliferation, differentiation and control, 1 | ENSG00000107281 | NPDC1 | UP |
| DnaJ (Hsp40) homolog, subfamily C, member 12 | ENSG00000108176 | DNAJC12 | UP |
| Cytochrome P450, family 2, subfamily C, polypeptide 18 | ENSG00000108242 | CYP2C18 | DOWN |
| RUN domain containing 3A | ENSG00000108309 | RUNDC3A | UP |
| LUC7-like 3 (S. cerevisiae) | ENSG00000108848 | LUC7L3 | UP |
| Carboxypeptidase E | ENSG00000109472 | CPE | UP |
| Membrane-spanning 4-domains, subfamily A, member 4A | ENSG00000110079 | MS4A4A | DOWN |
| Choline phosphotransferase 1 | ENSG00000111666 | CHPT1 | DOWN |
| Enolase 2 (gamma, neuronal) | ENSG00000111674 | ENO2 | UP |
| Lysophosphatidylcholine acyltransferase 3 | ENSG00000111684 | LPCAT3 | DOWN |
| C2 calcium-dependent domain containing 5 | ENSG00000111731 | C2CD5 | UP |
| Mannosidase, alpha, class 1A, member 1 | ENSG00000111885 | MAN1A1 | DOWN |
| WAS protein family, member 1 | ENSG00000112290 | WASF1 | UP |
| Vanin 1 | ENSG00000112299 | VNN1 | DOWN |
| Chromosome 3 open reading frame 14 | ENSG00000114405 | C3orf14 | UP |
| Arylacetamide deacetylase | ENSG00000114771 | AADAC | DOWN |
| Fanconi anemia, complementation group L | ENSG00000115392 | FANCL | UP |
| Rho family GTPase 3 | ENSG00000115963 | RND3 | DOWN |
| S100P binding protein | ENSG00000116497 | S100PBP | UP |
| DnaJ (Hsp40) homolog, subfamily C, member 6 | ENSG00000116675 | DNAJC6 | UP |
| Nuclear receptor subfamily 5, group A, member 2 | ENSG00000116833 | NR5A2 | DOWN |
| Acyl-CoA dehydrogenase, C-4 to C-12 straight chain | ENSG00000117054 | ACADM | DOWN |
| CD48 molecule | ENSG00000117091 | CD48 | DOWN |
| Solute carrier family 35 (UDP-N-acetylglucosamine (UDP-GlcNAc) transporter), member A3 | ENSG00000117620 | SLC35A3 | DOWN |
| Apolipoprotein A-I | ENSG00000118137 | APOA1 | DOWN |
| High mobility group nucleosomal binding domain 3 | ENSG00000118418 | HMGN3 | UP |
| ATP-binding cassette, sub-family G (WHITE), member 2 | ENSG00000118777 | ABCG2 | DOWN |
| Glutamic-oxaloacetic transaminase 1, soluble | ENSG00000120053 | GOT1 | DOWN |
| Nucleoporin 43kDa | ENSG00000120253 | NUP43 | UP |
| Tumor necrosis factor receptor superfamily, member 10b | ENSG00000120889 | TNFRSF10B | DOWN |
| Bromodomain adjacent to zinc finger domain, 2B | ENSG00000123636 | BAZ2B | UP |
| Potassium inwardly-rectifying channel, subfamily J, member 2 | ENSG00000123700 | KCNJ2 | UP |
| Obscurin-like 1 | ENSG00000124006 | OBSL1 | UP |
| Phosphoenolpyruvate carboxykinase 1 (soluble) | ENSG00000124253 | PCK1 | DOWN |
| Peptidase D | ENSG00000124299 | PEPD | DOWN |
| SRY (sex determining region Y)-box 4 | ENSG00000124766 | SOX4 | UP |
| Metallothionein 1G | ENSG00000125144 | MT1G | DOWN |
| Metallothionein 2A | ENSG00000125148 | MT2A | DOWN |
| NK2 homeobox 2 | ENSG00000125820 | NKX2-2 | UP |
| Mercaptopyruvate sulfurtransferase | ENSG00000128309 | MPST | DOWN |
| Thiosulfate sulfurtransferase (rhodanese) | ENSG00000128311 | TST | DOWN |
| Ribonuclease, RNase A family, 1 (pancreatic) | ENSG00000129538 | RNASE1 | DOWN |
| Fibroblast growth factor 13 | ENSG00000129682 | FGF13 | UP |
| Scaffold attachment factor B2 | ENSG00000130254 | SAFB2 | UP |
| Argininosuccinate synthase 1 | ENSG00000130707 | ASS1 | DOWN |
| Gse1 coiled-coil protein | ENSG00000131149 | GSE1 | UP |
| Microtubule-associated protein 1B | ENSG00000131711 | MAP1B | UP |
| KH domain containing, RNA binding, signal transduction associated 3 | ENSG00000131773 | KHDRBS3 | UP |
| RAP1 GTPase activating protein 2 | ENSG00000132359 | RAP1GAP2 | UP |
| Synaptosomal-associated protein, 25kDa | ENSG00000132639 | SNAP25 | UP |
| KIAA0907 | ENSG00000132680 | KIAA0907 | UP |
| Brain expressed, X-linked 1 | ENSG00000133169 | BEX1 | UP |
| Adenosine monophosphate deaminase 3 | ENSG00000133805 | AMPD3 | UP |
| Hydroxysteroid (17-beta) dehydrogenase 4 | ENSG00000133835 | HSD17B4 | DOWN |
| Abelson helper integration site 1 | ENSG00000135541 | AHI1 | UP |
| Secernin 1 | ENSG00000136193 | SCRN1 | UP |
| Cell death-inducing DFFA-like effector b | ENSG00000136305 | CIDEB | DOWN |
| Syntaxin binding protein 1 | ENSG00000136854 | STXBP1 | UP |
| Solute carrier family 31 (copper transporter), member 1 | ENSG00000136868 | SLC31A1 | DOWN |
| Aldolase B, fructose-bisphosphate | ENSG00000136872 | ALDOB | DOWN |
| Ubiquitin associated protein 2 | ENSG00000137073 | UBAP2 | UP |
| Aldehyde dehydrogenase 1 family, member B1 | ENSG00000137124 | ALDH1B1 | DOWN |
| Cap methyltransferase 1 | ENSG00000137200 | CMTR1 | UP |
| Ketohexokinase (fructokinase) | ENSG00000138030 | KHK | DOWN |
| Prolyl endopeptidase-like | ENSG00000138078 | PREPL | UP |
| Solute carrier family 3 (amino acid transporter heavy chain), member 1 | ENSG00000138079 | SLC3A1 | DOWN |
| Cytochrome P450, family 2, subfamily C, polypeptide 9 | ENSG00000138109 | CYP2C9 | DOWN |
| 3'-phosphoadenosine 5'-phosphosulfate synthase 1 | ENSG00000138801 | PAPSS1 | UP |
| Microsomal triglyceride transfer protein | ENSG00000138823 | MTTP | DOWN |
| Electron-transfer-flavoprotein, alpha polypeptide | ENSG00000140374 | ETFA | DOWN |
| V-erb-b2 avian erythroblastic leukemia viral oncogene homolog 2 | ENSG00000141736 | ERBB2 | DOWN |
| Dermatopontin | ENSG00000143196 | DPT | DOWN |
| S100 calcium binding protein A8 | ENSG00000143546 | S100A8 | DOWN |
| Centrosomal protein 170kDa | ENSG00000143702 | CEP170 | UP |
| Membrane bound O-acyltransferase domain containing 2 | ENSG00000143797 | MBOAT2 | UP |
| Poly (ADP-ribose) polymerase 1 | ENSG00000143799 | PARP1 | UP |
| Nuclear receptor subfamily 1, group I, member 2 | ENSG00000144852 | NR1I2 | DOWN |
| IQ motif containing GTPase activating protein 2 | ENSG00000145703 | IQGAP2 | DOWN |
| Peptidylglycine alpha-amidating monooxygenase | ENSG00000145730 | PAM | UP |
| Pleckstrin homology domain interacting protein | ENSG00000146247 | PHIP | UP |
| calcium/calmodulin dependent protein kinase II beta(CAMK2B) | ENSG00000058404 | CAMK2B | UP |
| Perilipin 2 | ENSG00000147872 | PLIN2 | DOWN |
| Eukaryotic translation initiation factor 4E binding protein 2 | ENSG00000148730 | EIF4EBP2 | DOWN |
| Gamma-glutamyltransferase light chain 1 | ENSG00000149435 | GGTLC1 | DOWN |
| SET binding protein 1 | ENSG00000152217 | SETBP1 | UP |
| Centrin, EF-hand protein, 3 | ENSG00000153140 | CETN3 | UP |
| Sodium channel, voltage-gated, type III, alpha subunit | ENSG00000153253 | SCN3A | UP |
| Protein tyrosine phosphatase, receptor type, N polypeptide 2 | ENSG00000155093 | PTPRN2 | UP |
| Solute carrier family 16 (monocarboxylate transporter), member 1 | ENSG00000155380 | SLC16A1 | DOWN |
| Guanine nucleotide binding protein (G protein), q polypeptide | ENSG00000156052 | GNAQ | UP |
| WD repeat domain 19 | ENSG00000157796 | WDR19 | UP |
| CD1d molecule | ENSG00000158473 | CD1D | DOWN |
| Synaptic vesicle glycoprotein 2A | ENSG00000159164 | SV2A | UP |
| Interleukin 6 receptor | ENSG00000160712 | IL6R | DOWN |
| Cytochrome P450, family 3, subfamily A, polypeptide 4 | ENSG00000160868 | CYP3A4 | DOWN |
| Acyl-CoA oxidase 1, palmitoyl | ENSG00000161533 | ACOX1 | DOWN |
| Aldo-keto reductase family 7, member A3 (aflatoxin aldehyde reductase) | ENSG00000162482 | AKR7A3 | DOWN |
| Chromosome 1 open reading frame 115 | ENSG00000162817 | C1orf115 | DOWN |
| Cathepsin S | ENSG00000163131 | CTSS | DOWN |
| Frizzled family receptor 5 | ENSG00000163251 | FZD5 | DOWN |
| Serpin peptidase inhibitor, clade I (neuroserpin), member 1 | ENSG00000163536 | SERPINI1 | UP |
| Cytoplasmic linker associated protein 2 | ENSG00000163539 | CLASP2 | UP |
| Protein tyrosine phosphatase, non-receptor type 13 (APO-1/CD95 (Fas)-associated phosphatase) | ENSG00000163629 | PTPN13 | UP |
| Deoxyribonuclease I-like 3 | ENSG00000163687 | DNASE1L3 | DOWN |
| Hydroxyprostaglandin dehydrogenase 15-(NAD) | ENSG00000164120 | HPGD | DOWN |
| Fructose-1,6-bisphosphatase 1 | ENSG00000165140 | FBP1 | DOWN |
| Crystallin, lambda 1 | ENSG00000165475 | CRYL1 | DOWN |
| Mitochondrial calcium uptake 2 | ENSG00000165487 | MICU2 | UP |
| KIAA0355 | ENSG00000166398 | KIAA0355 | UP |
| Coiled-coil domain containing 68 | ENSG00000166510 | CCDC68 | DOWN |
| Secretogranin V (7B2 protein) | ENSG00000166922 | SCG5 | UP |
| Acetyl-CoA acyltransferase 2 | ENSG00000167315 | ACAA2 | DOWN |
| ATP-binding cassette, sub-family A (ABC1), member 3 | ENSG00000167972 | ABCA3 | UP |
| Kinesin family member 5C | ENSG00000168280 | KIF5C | UP |
| PX domain containing 1 | ENSG00000168994 | PXDC1 | DOWN |
| Metallothionein 1E | ENSG00000169715 | MT1E | DOWN |
| Metastasis suppressor 1 | ENSG00000170873 | MTSS1 | UP |
| Chromosome 11 open reading frame 24 | ENSG00000171067 | C11orf24 | DOWN |
| ATPase, H+ transporting V0 subunit e2 | ENSG00000171130 | ATP6V0E2 | UP |
| Chromosome 9 open reading frame 16 | ENSG00000171159 | C9orf16 | UP |
| Potassium channel, subfamily K, member 3 | ENSG00000171303 | KCNK3 | UP |
| Electron-transferring-flavoprotein dehydrogenase | ENSG00000171503 | ETFDH | DOWN |
| Glycine amidinotransferase (L-arginine:glycine amidinotransferase) | ENSG00000171766 | GATM | DOWN |
| Secretogranin II | ENSG00000171951 | SCG2 | UP |
| Succinate-CoA ligase, GDP-forming, beta subunit | ENSG00000172340 | SUCLG2 | DOWN |
| Sphingomyelin phosphodiesterase, acid-like 3A | ENSG00000172594 | SMPDL3A | DOWN |
[truncated: 5,107 more chars]
