# Supplementary material for: RUNDC3A regulates SNAP25-mediated chemotherapy resistance by binding AKT in gastric neuroendocrine carcinoma (GNEC)
Source: Cell Death Discov. 2022 Jun 25;8:296. doi: 10.1038/s41420-022-01084-4 (PMC9233710; doi:10.1038/s41420-022-01084-4)
Supplement: Supplementary file 3 — Original Data File [file 41420_2022_1084_MOESM3_ESM.docx]

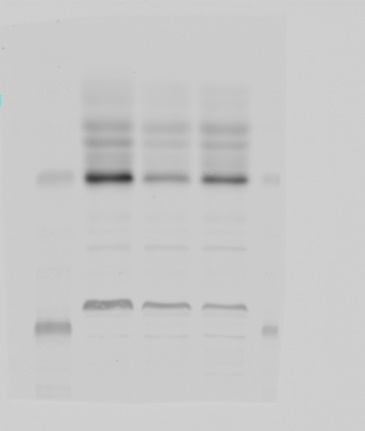
figure3 ECC12 SNAP25 knockdown effect


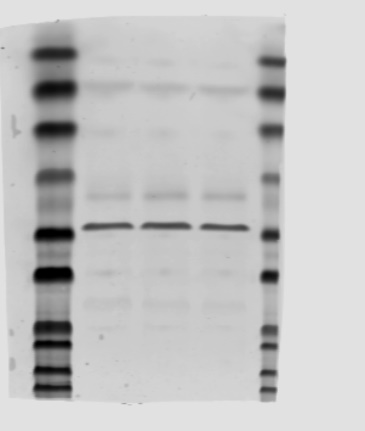
 figure3 ECC12 SNAP25 knockdown effect GAPDH


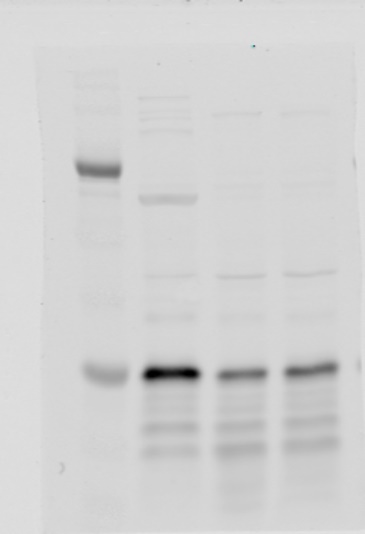
 figure3 ECC12 SNAP25 knockdown effect


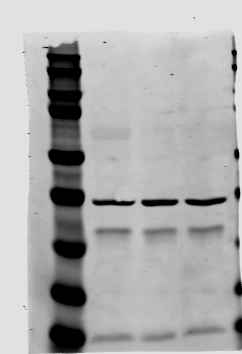
 figure3 ECC12 SNAP25 knockdown effect GAPDH


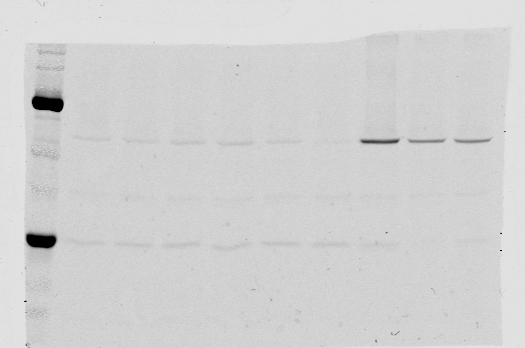
Figure5 total AKT in ECC12


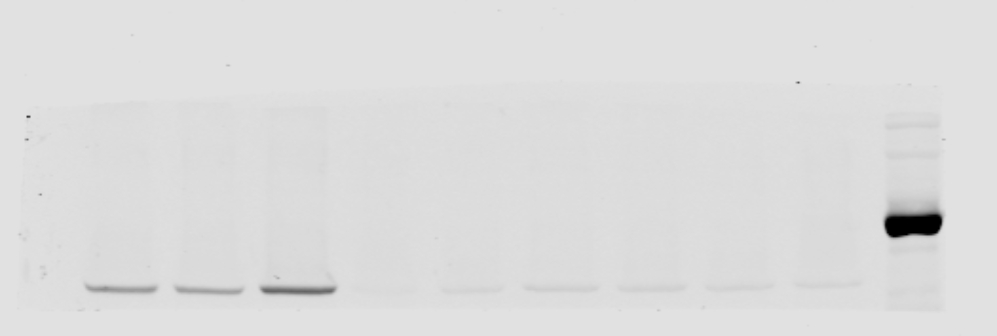
 Figure5 p-AKT in ECC12


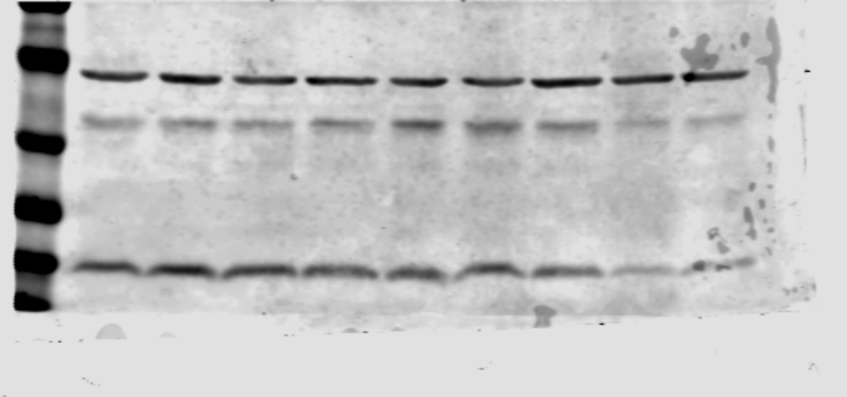
 Figure5 p-AKT in ECC12 GAPDH


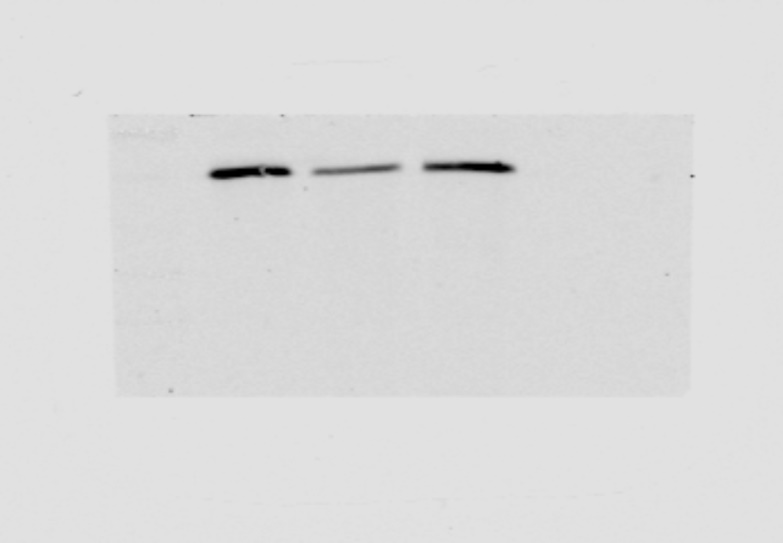
 Figure5 total AKT in ECC10


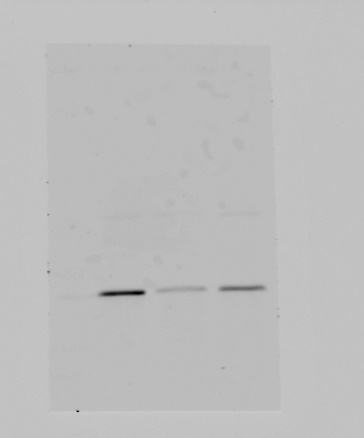
 Figure5 p-AKT in ECC10


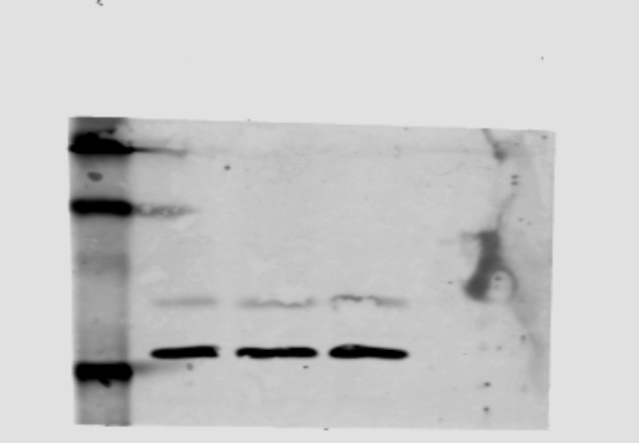
 Figure5 p-AKT in ECC10 GAPDH


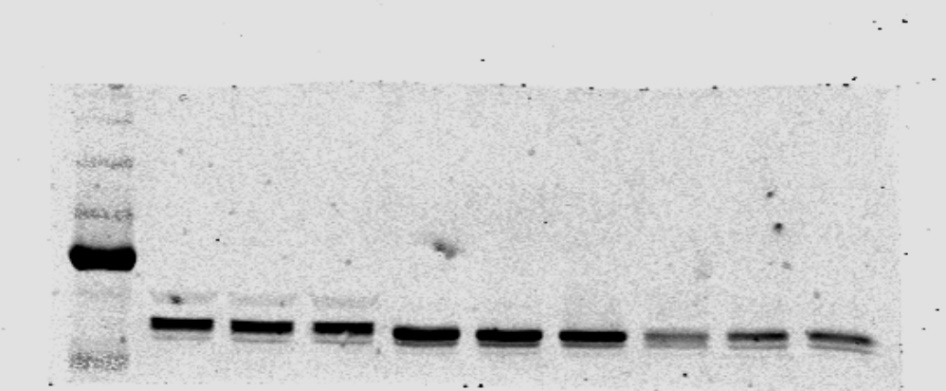
 Figure5 AKT in OE SNAP25 ECC12


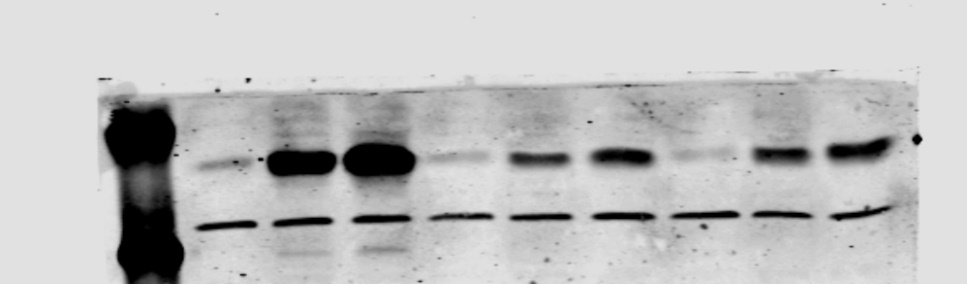
 Figure5 AKT in OE SNAP25 ECC12 GAPDH


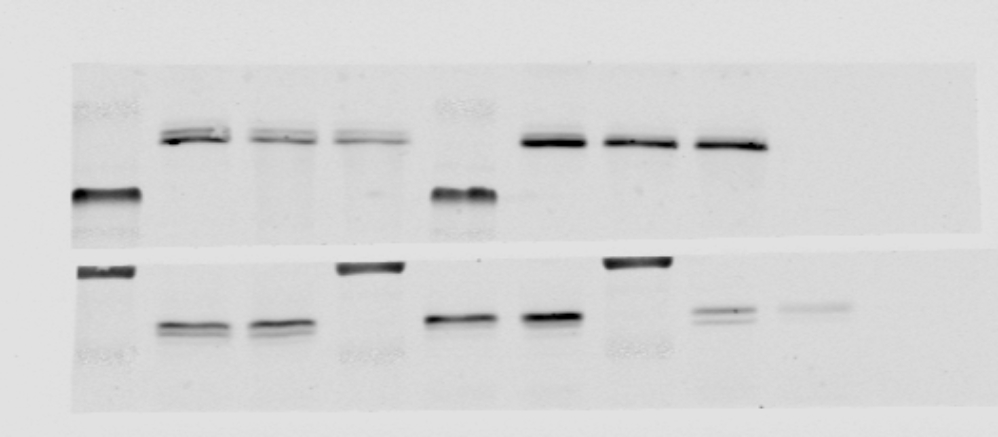
 Figure5 AKT in OE SNAP25 ECC10


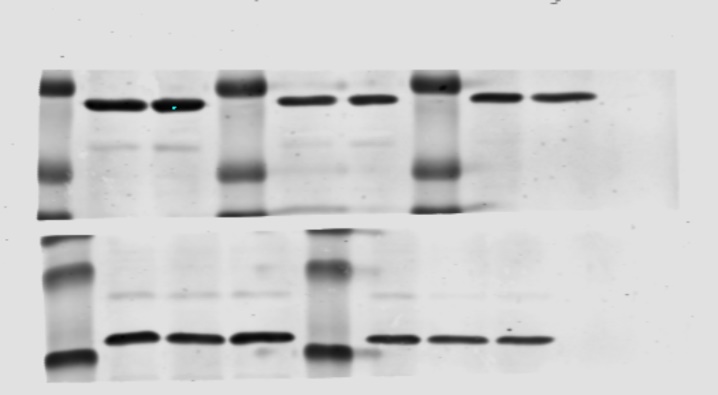
 Figure5 AKT in OE SNAP25 ECC10 GAPDH


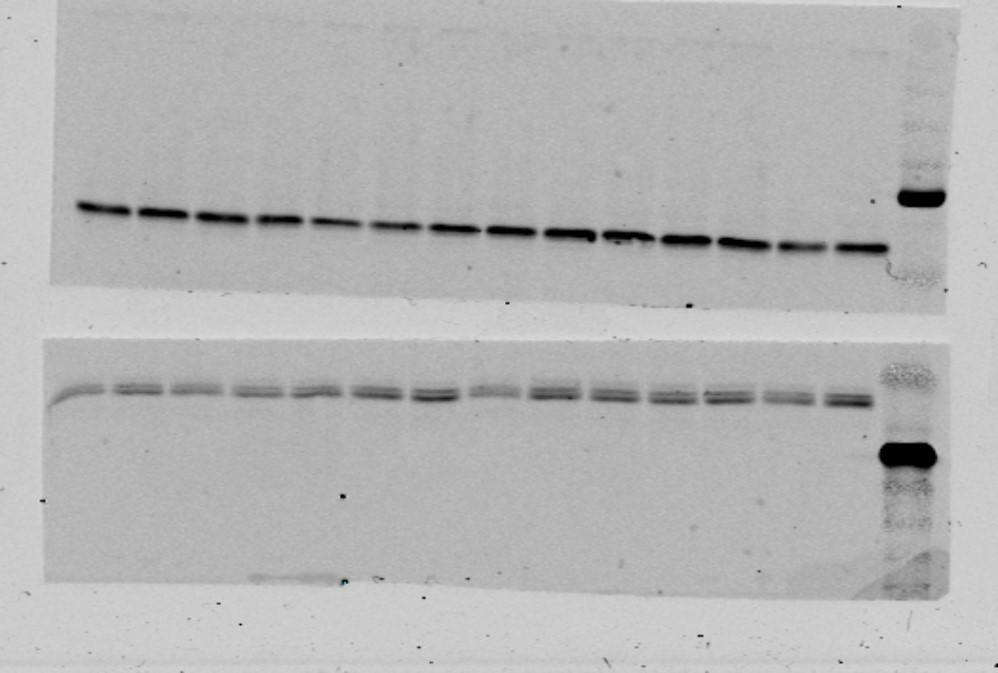
 Figure5 AKT half life in ECC12


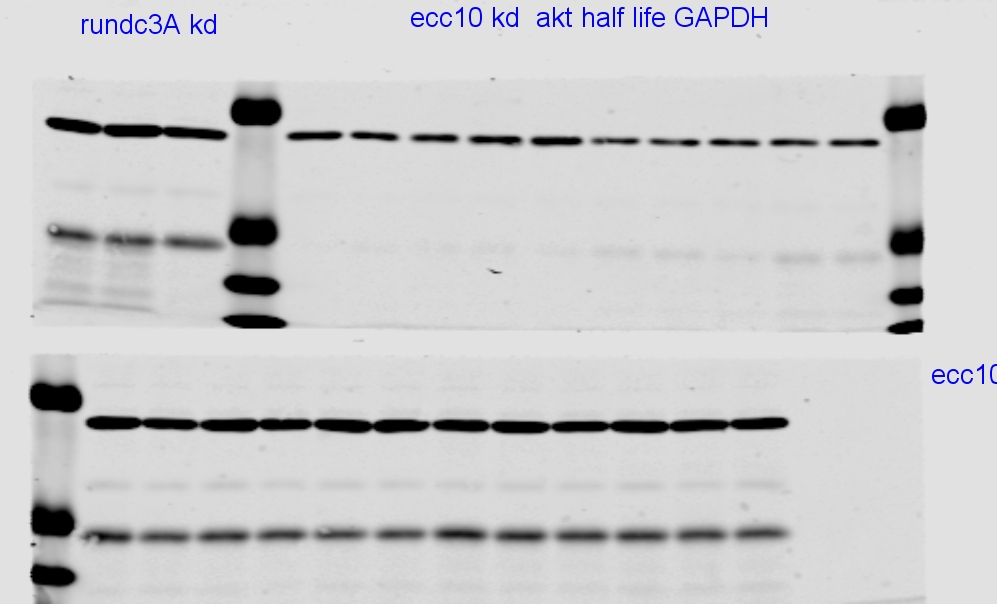
 Figure5 AKT half life GAPDH in ECC12


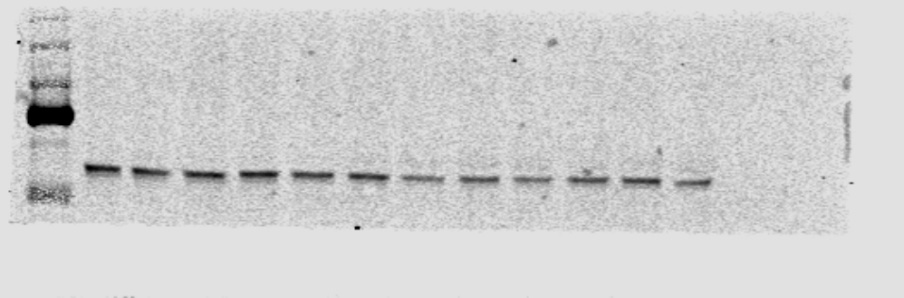
 Figure5 AKT half life in ECC10


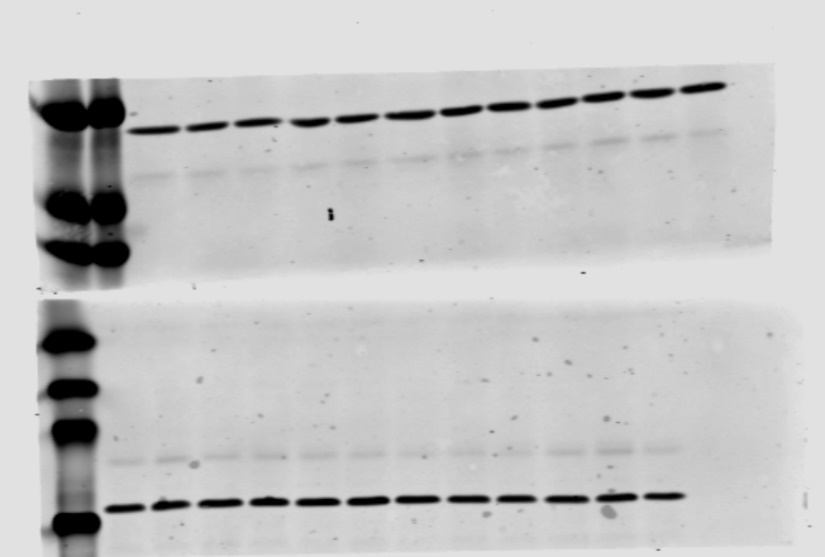
 Figure5 AKT half life GAPDH in ECC10


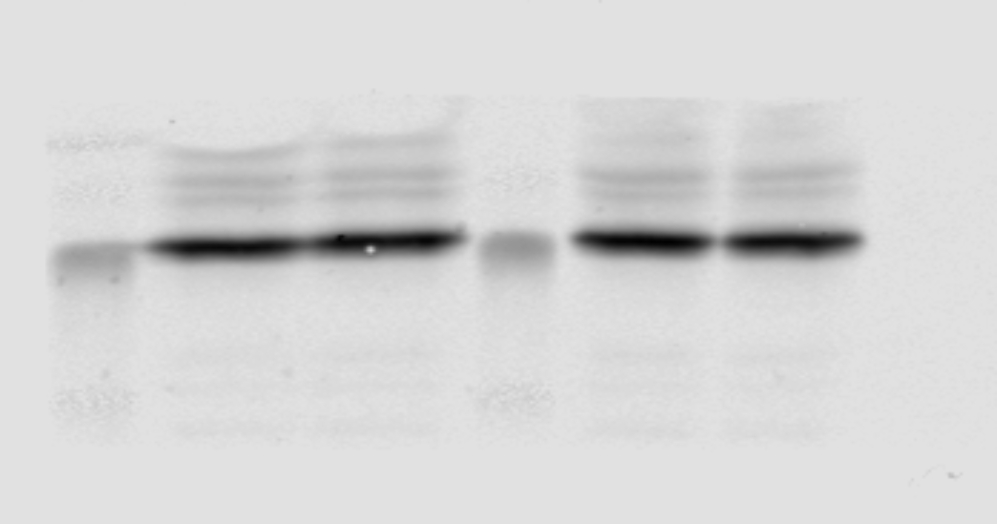
 Figure5I SNAP25 input


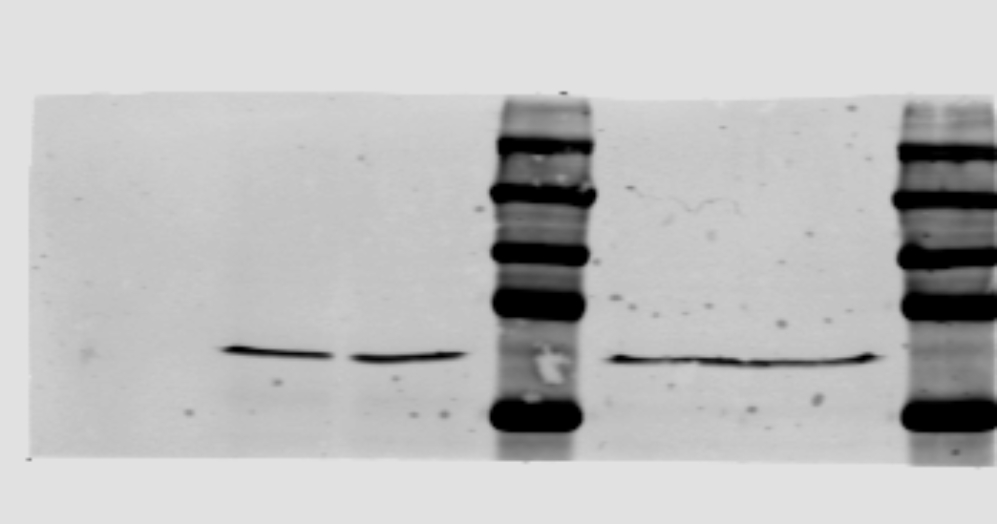
 Figure5I AKT input


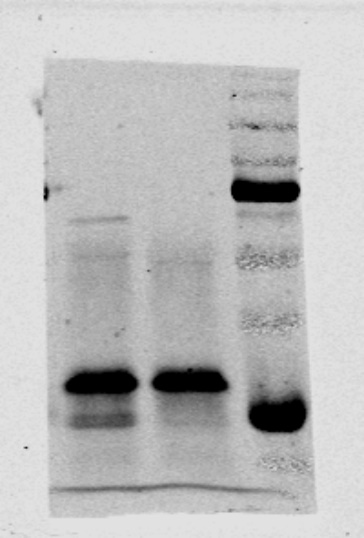
 Figure5I IB SNAP25


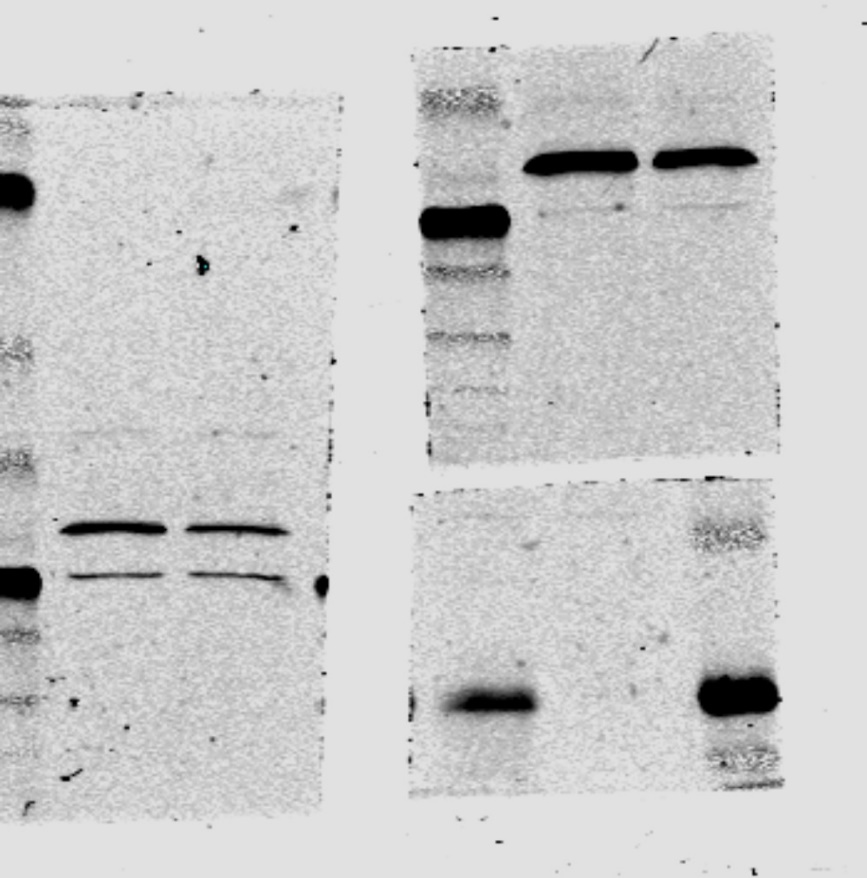
Figure 5J AKT input


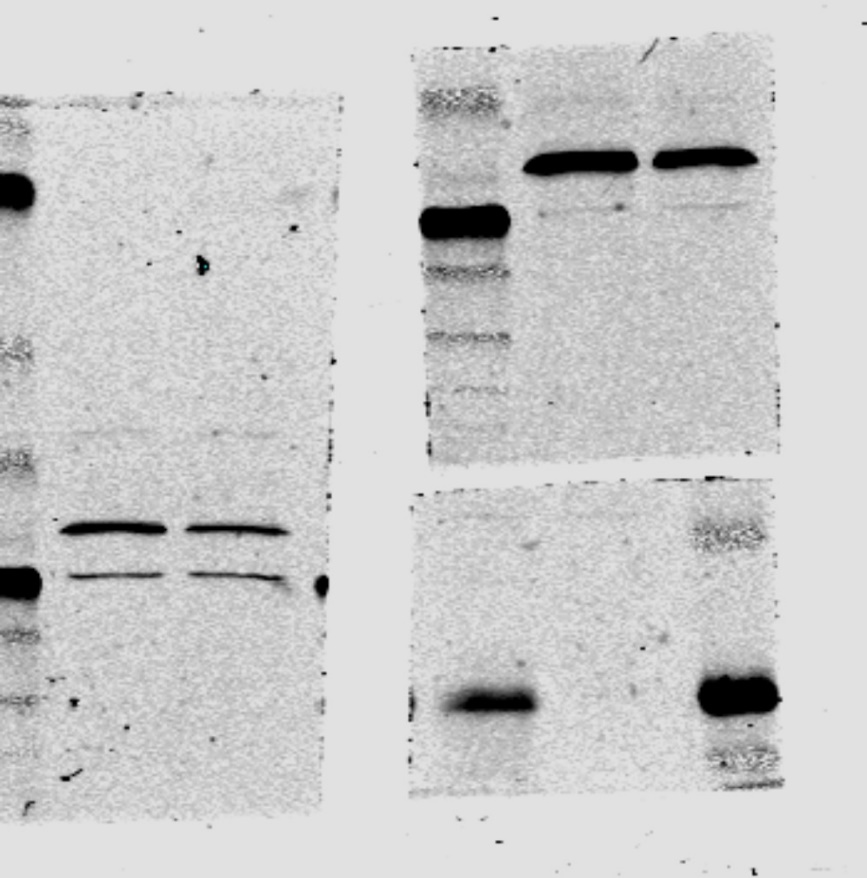
 Figure 5J SNAP25 input


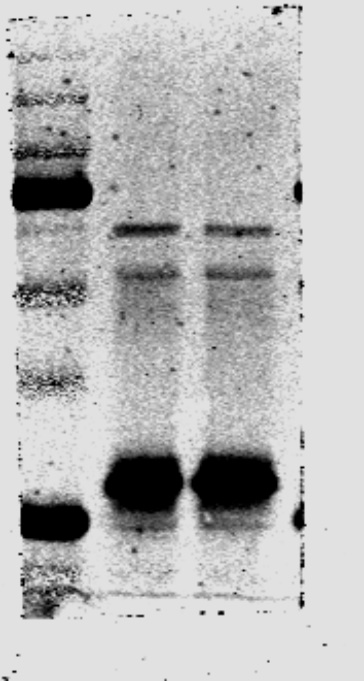
 Figure 5J IB Ubiquitin


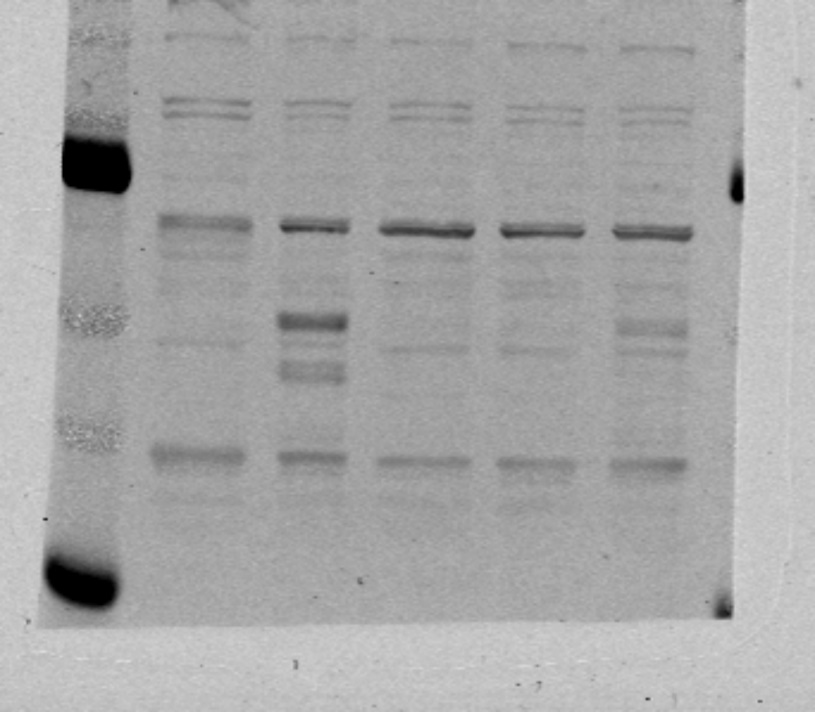
 Figure 8 RUNDC3A knockdown effect in ECC12


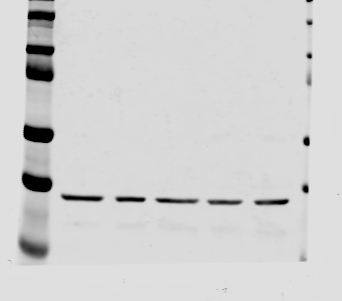
 Figure 8 RUNDC3A knockdown effect GAPDH in ECC12


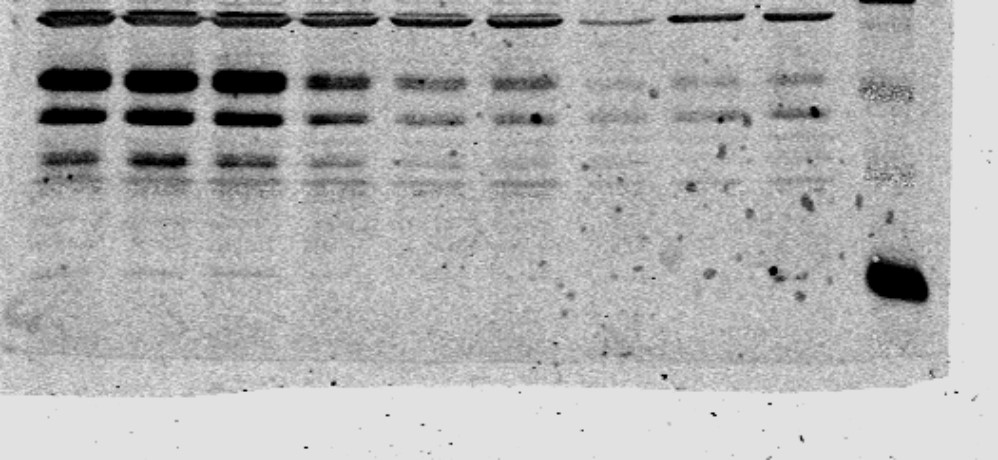
 Figure 8 RUNDC3A knockdown effect in ECC10


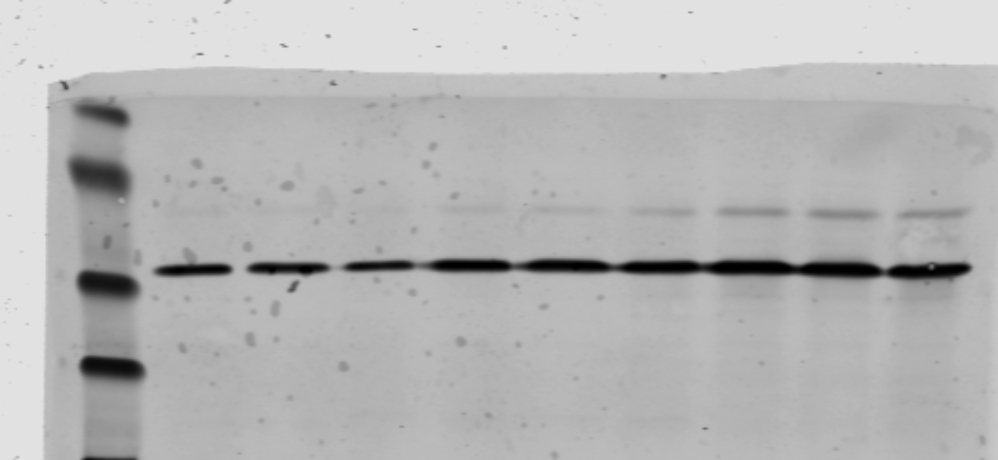
 Figure 8 RUNDC3A knockdown effect GAPDH in ECC10


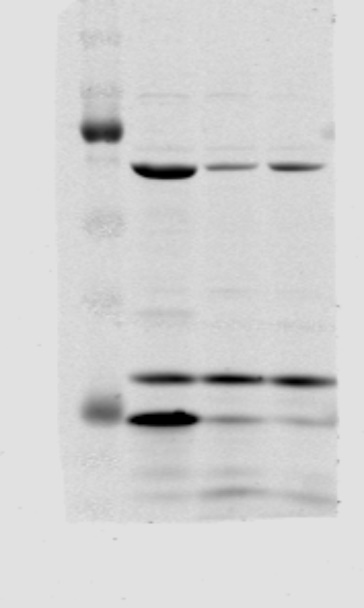

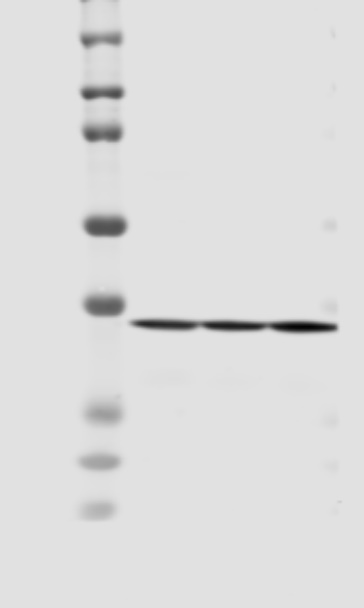
 Figure 8L


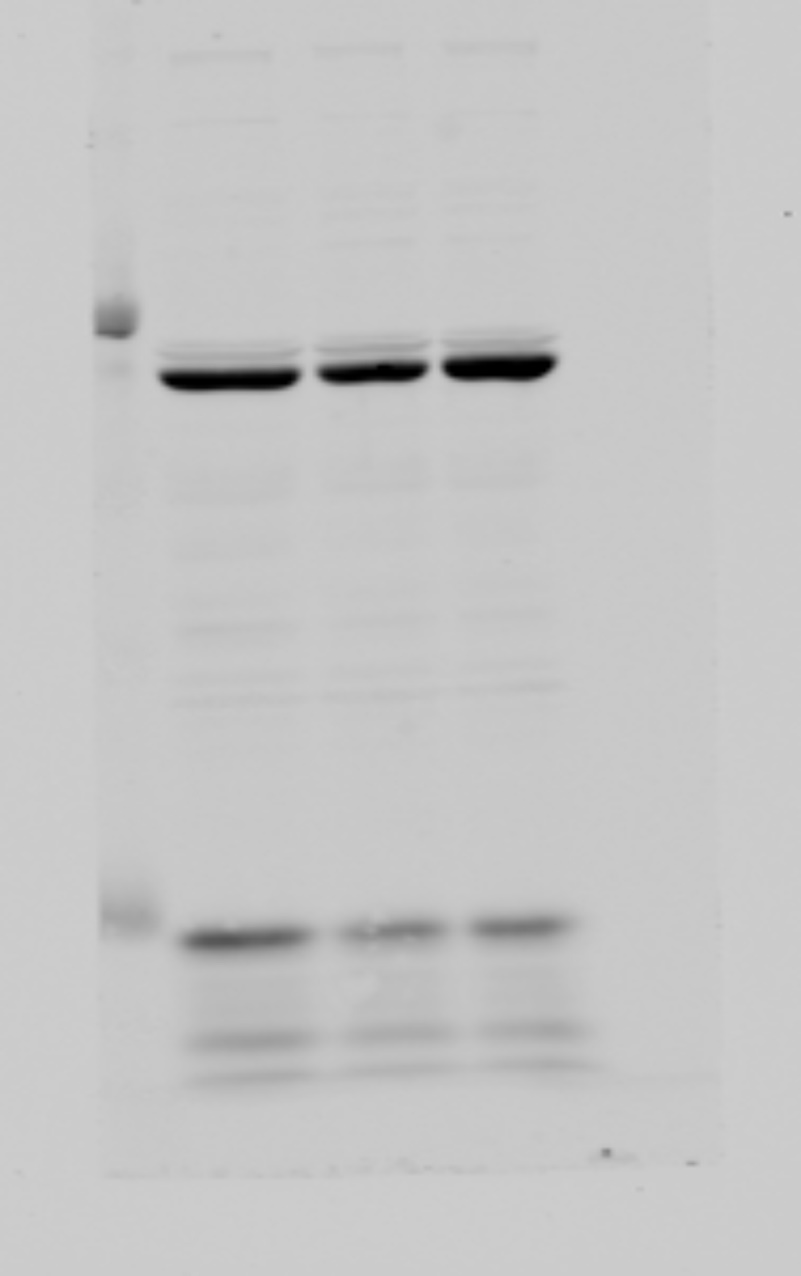

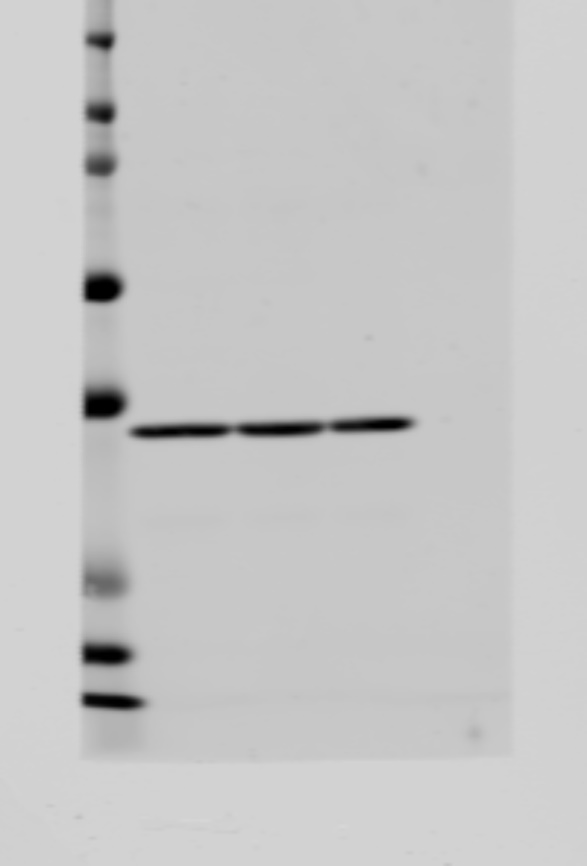
Figure 8M


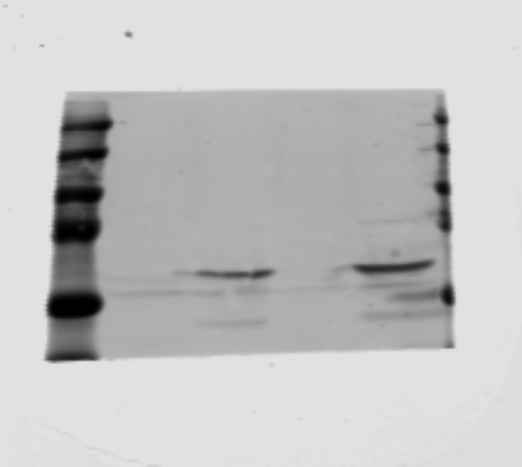

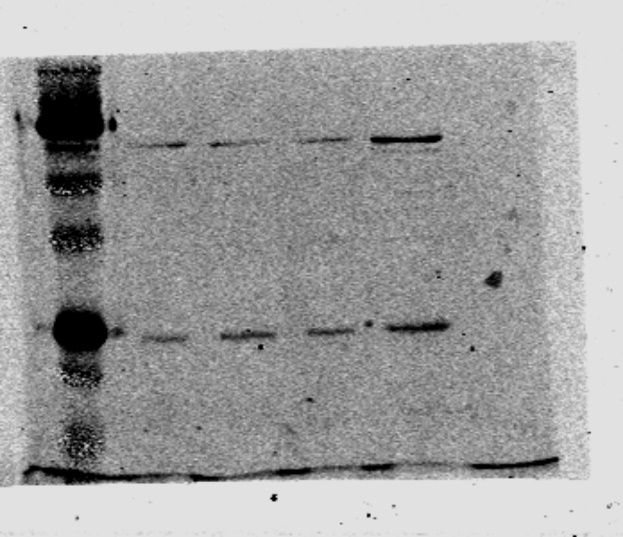

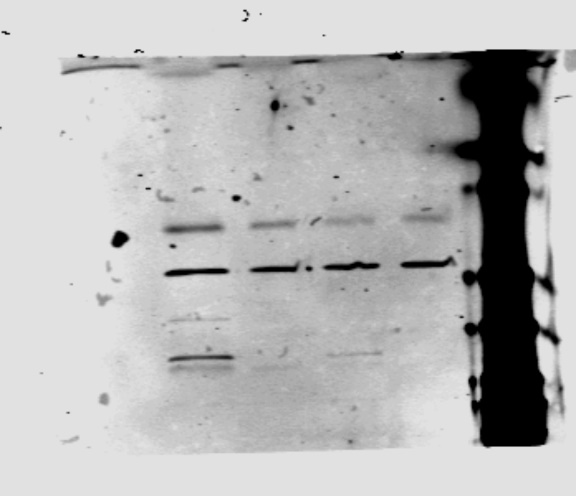
Figure 9N


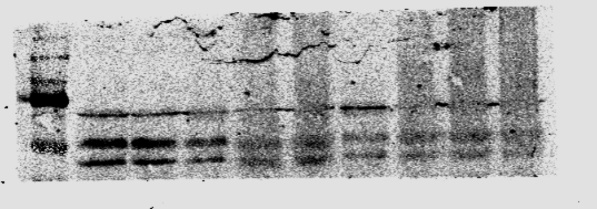

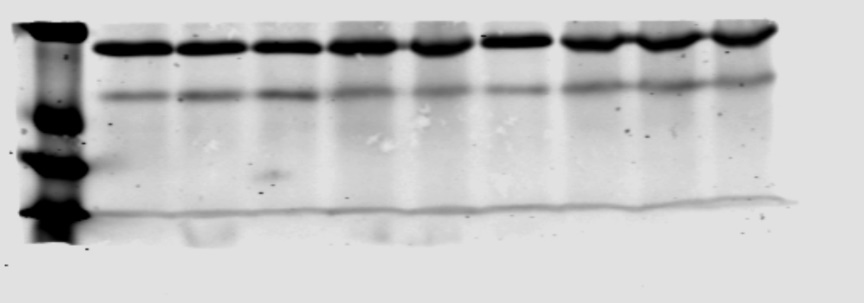


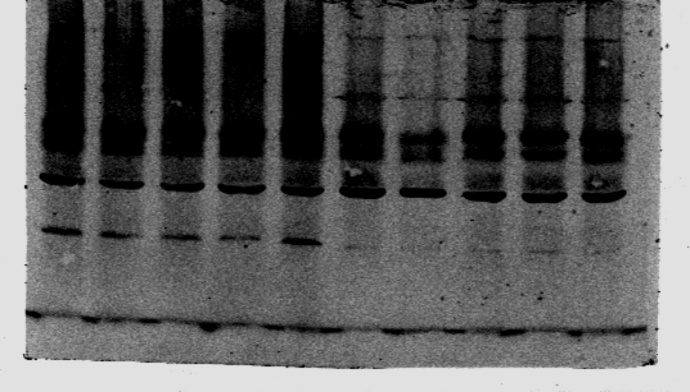
Figure 8O


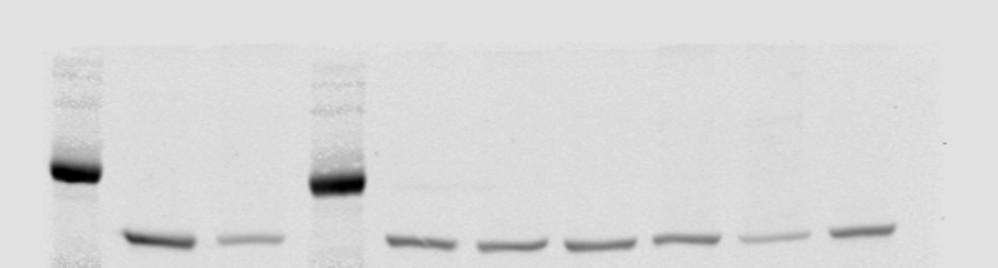

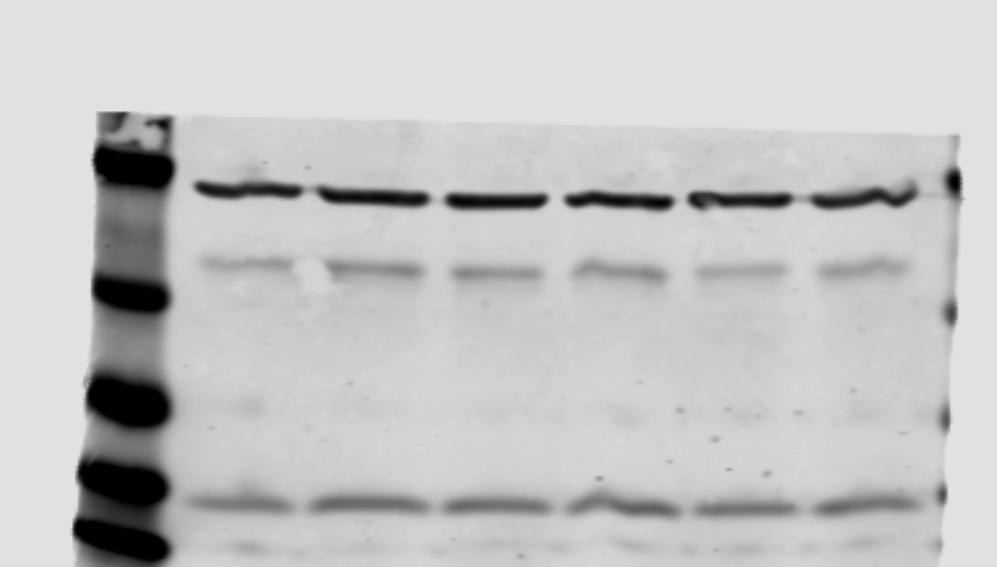

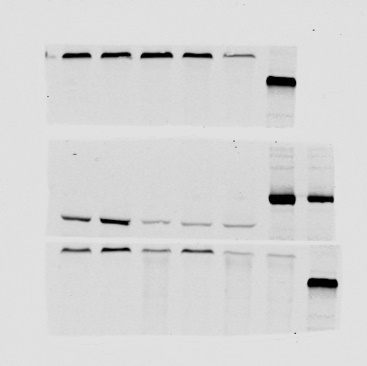

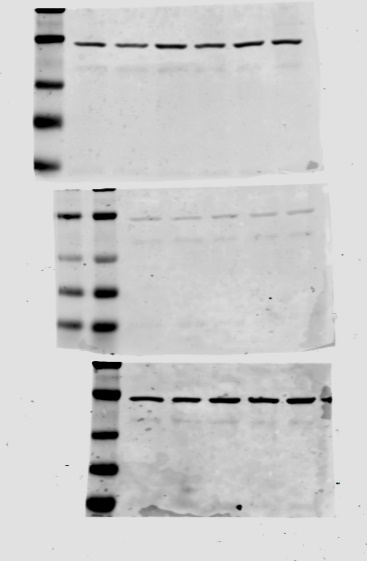
Supplmentary1


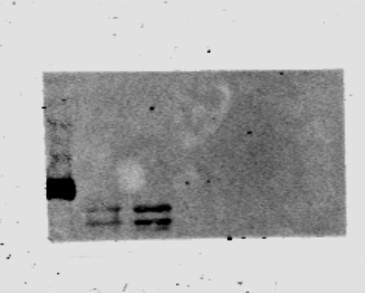

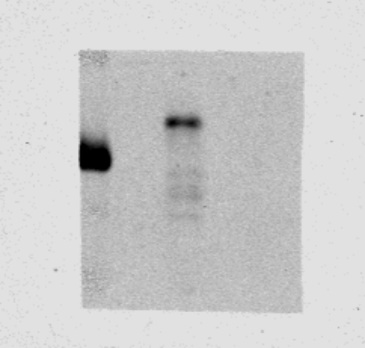

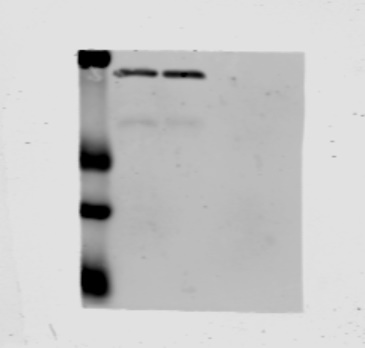


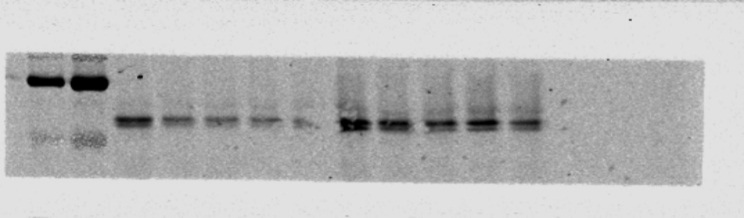

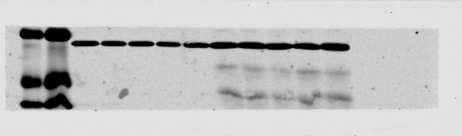
Supplemetary2


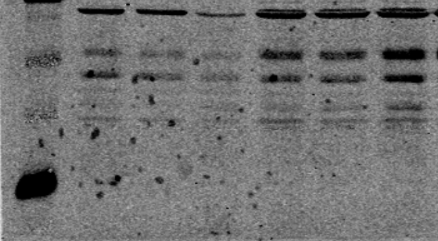


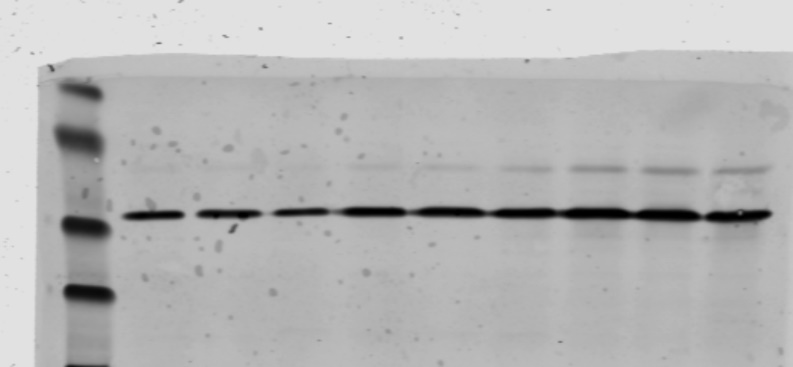


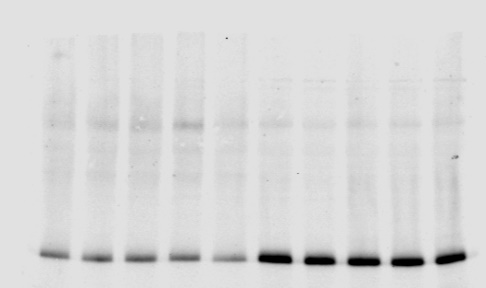

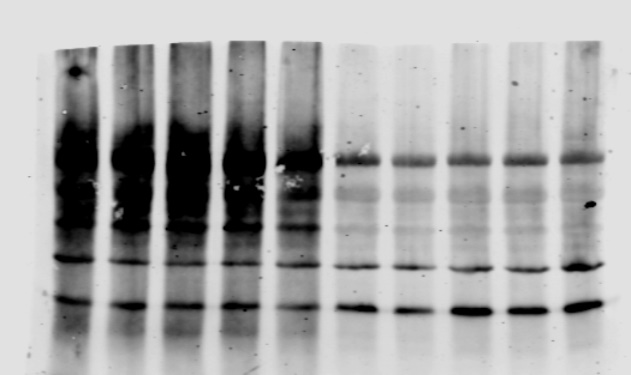

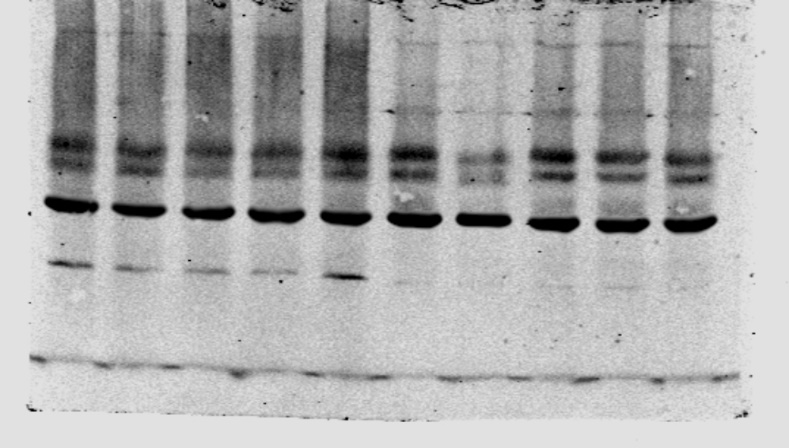
 Supplementary8
